# (SiFA)SeFe: A Hydrophilic Silicon-Based Fluoride Acceptor Enabling Versatile Peptidic Radiohybrid Tracers

**DOI:** 10.1021/acs.jmedchem.4c00924

**Published:** 2024-08-08

**Authors:** Sandra Deiser, Sebastian Fenzl, Victor König, Marike Drexler, Lydia M. Smith, Madeleine E. George, Roswitha Beck, Timothy H. Witney, Shigeyoshi Inoue, Angela Casini

**Affiliations:** Chair of Medicinal and Bioinorganic Chemistry, Department of Chemistry, School of Natural Sciences, Technical University of Munich, 85748 Garching b. München, Germany; Chair of Pharmaceutical Radiochemistry, Department of Chemistry, School of Natural Sciences, Technical University of Munich, 85748 Garching b. München, Germany; Chair of Pharmaceutical Radiochemistry, Department of Chemistry, School of Natural Sciences, Technical University of Munich, 85748 Garching b. München, Germany; Institute of Silicon Chemistry, Department of Chemistry, School of Natural Sciences, Technical University of Munich, 85748 Garching b. München, Germany; Chair of Pharmaceutical Radiochemistry, Department of Chemistry, School of Natural Sciences, Technical University of Munich, 85748 Garching b. München, Germany; Chair of Medicinal and Bioinorganic Chemistry, Department of Chemistry, School of Natural Sciences, Technical University of Munich, 85748 Garching b. München, Germany; Chair of Pharmaceutical Radiochemistry, Department of Chemistry, School of Natural Sciences, Technical University of Munich, 85748 Garching b. München, Germany; School of Biomedical Engineering and Imaging Sciences, King’s College London, London SE1 7EH, U.K; School of Biomedical Engineering and Imaging Sciences, King’s College London, London SE1 7EH, U.K; Chair of Pharmaceutical Radiochemistry, Department of Chemistry, School of Natural Sciences, Technical University of Munich, 85748 Garching b. München, Germany; School of Biomedical Engineering and Imaging Sciences, King’s College London, London SE1 7EH, U.K; Institute of Silicon Chemistry, Department of Chemistry, School of Natural Sciences, Technical University of Munich, 85748 Garching b. München, Germany; Chair of Medicinal and Bioinorganic Chemistry, Department of Chemistry, School of Natural Sciences, Technical University of Munich, 85748 Garching b. München, Germany; Chair of Pharmaceutical Radiochemistry, Department of Chemistry, School of Natural Sciences, Technical University of Munich, 85748 Garching b. München, Germany

## Abstract

The radiohybrid (rh) concept to design targeted (and chemically identical) radiotracers for imaging or radionuclide therapy of tumors has gained momentum. For this strategy, a new bifunctional Silicon-based Fluoride Acceptor (SiFA) moiety **(SiFA)SeFe** was synthesized, endowed with improved hydrophilicity and high versatility of integration into rh-compounds. Preliminary radiolabeling and stability studies under different conditions were conducted using model bioconjugate peptides. Further, three somatostatin receptor 2 (sstR2)-targeted rh-compounds (**(SiFA)SeFe-rhTATE1−3**, TATE = (Tyr^3^)-octreotate) were developed. Compound **(SiFA)SeFe-rhTATE3**, enables labeling with ^18^F for PET imaging or chelation of ^177^Lu for therapy. The rh-compounds possess comparable receptor binding affinity and *in vitro* performance as good as the clinically proven gold standards. SstR2-specificity was further shown for **(SiFA)SeFe-rhTATE2** using the chicken chorioallantoic membrane (CAM) model. The biodistribution of two compounds in mice showed high accumulation in tumors and excretion via the kidneys, demonstrating the clinical applicability of the **(SiFA)SeFe** moiety.

**Figure:**
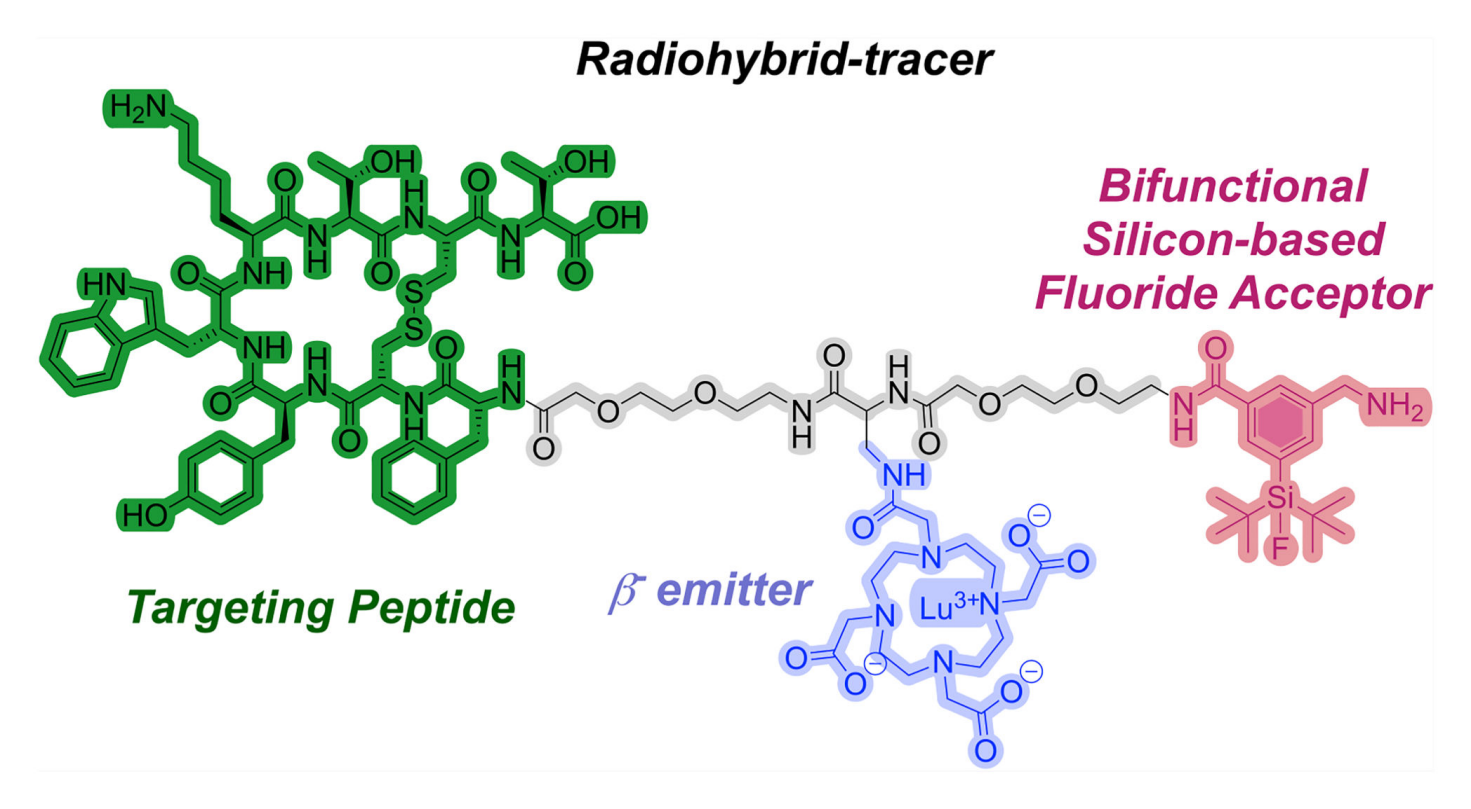

## Introduction

The term *theranostic* refers to a treatment strategy that combines therapeutic and diagnostic capabilities. This type of approach can be exploited clinically in various ways, including by imaging the biodistribution of the targeted drug, selecting patients to receive targeted therapies, and by visualizing and quantifying both the presence and engagement of the target to limit possible side-effects. This concept has been successfully attained in the area of pharmaceutical radiochemistry via different strategies, such as the incorporation of a radioisotope able to emit both *γ*-rays or positrons for imaging, and ionizing radiation (*β*^−^ and *α*) for therapy (“true theranostic”, e.g. ^177^Lu, ^64^Cu).^1–3^ As an alternative strategy, the use of isotopically matched pairs can be applied. Radiopharmaceuticals of this type contain either a therapeutic or a diagnostic radionuclide of the same element (e.g., ^64^Cu/^67^Cu and ^43^Sc/^47^Sc). Another way to realize a radio-theranostic compound is the so-called matched/mixed theranostic pair, whereby radionuclides of different elements can be incorporated in the same or a very similar compound.^4^ The matched/mixed pairs enable clinicians to select the radionuclides with the best chemical and physical properties for a specific task, and also allow for manifold possible combinations, provided that in either case the compound will feature very similar pharmacokinetic properties. An example of the latter strategy is offered by Zevalin, a monoclonal antibody featuring a chelator that can be labeled with ^90^Y (*β*^−^) and ^111^In (*γ*), respectively.^5^

**Figure 1:**
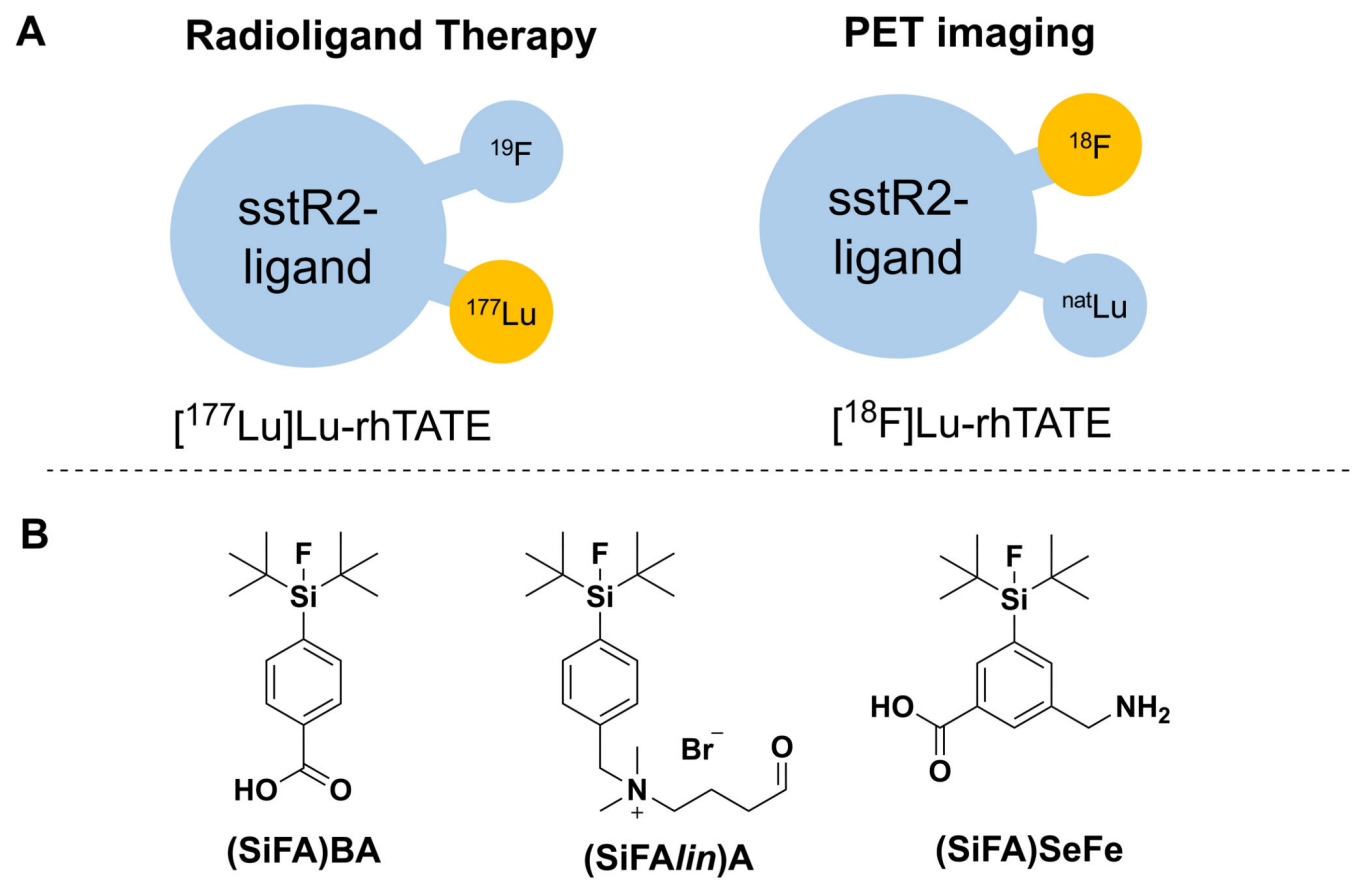
A) Schematic representation of the radiohybrid (rh) strategy targeted to sstR2 (somatostatine receptor 2). B) SiFA building blocks discussed in this study. Currently used SiFAs, the monofunctional **(SiFA)BA**^27^ (BA = benzoic acid) and **(SiFA*lin*)A**^24^ (A = Aldehyde) compounds, and the new bifunctional **(SiFA)SeFe**.

To achieve the matched/mixed pairs, another emerging design strategy is represented by the radiohybrid (rh) approach (Figure 1A), whereby a metal chelator is combined with an imaging modality such as a Silicon-based Fluoride Acceptor (SiFA) unit within a peptide targeted radiopharmaceutical.^6–8^ While the chelator is suitable for incorporating different radiometals, the SiFA can achieve ^18^F-fluorination under mild conditions to enable positron emission tomography (PET) imaging. It should be noted that ^18^F is the most used PET-nuclide, and its half-life (110 min), as well as low positron energy (maximum *β*^+^ energy = 635 keV), make it a close-to-ideal PET-isotope. However, direct ^18^F-labeling of peptides via nucleophilic aromatic substitution can be challenging due to the harsh reaction conditions required for the incorporation of [^18^F]fluoride. Other challenges include laborious and time-consuming labeling procedures and chemoselectivity aspects for the incorporation of ^18^F into peptides.^9^ In order to overcome these limitations, a variety of alternative ^18^F-labeling techniques have been investigated and assessed over the years, and the range of ^18^F-labeling has been extended from C−^18^F bond formation to the formation of ^18^F-bonds with heteroatoms, such as boron, aluminum, and eventually also silicon, through the use of SiFAs.^7,8,10^

The rh-concept has been successfully applied to achieve peptide-based radiotracers targeted to the prostate-specific membrane antigen (PSMA).^11,12^ Moreover, rh-tracers have been optimized to target the cholecystokinin-2 receptor (CCK-2R) in tumor models.^13–15^ The resulting compound can be radiolabeled with either ^18^F, while maintaining a non-radioactive metal in the chelator, or with radiometals (^68^Ga for PET imaging or ^177^Lu for *β*^−^ therapy, among others) while the SiFA moiety is nonradioactive. Eventually, a chemically identical pair of compounds (either ^19^F/radiometal or ^18^F/nonradioactive metal) is obtained which is endowed with identical pharmacokinetics for diagnostic and therapeutic applications (Figure 1A). Other explored radiohybrid approaches include the combination of radiometalation with ^125^I-labeling,^16^ and more recently with ^18^F-labeling via click chemistry^17^ or with organotrifluoroborate prosthetic groups.^18,19^

Despite the great potential of rh-compounds, a major challenge is the high lipophilic character of the SiFA moiety and its limited versatility of incorporation into a radiotracer scaffold (monofunctionalization) that can lead to an unfavorable slow hepatic excretion. The latter decreases the image quality and increases off-target radiation dose to the abdomen.^7,20^ Therefore, the need to further optimize SiFA building blocks remains of great importance. Previous approaches aiming at the reduction of lipophilicity of SiFAs introduced hydrophilic groups in the linking region; for example, the introduction of a carbohydrate component resulted in the promising fluorine-18 somatostatin receptor 2 (sstR2)-addressing ligand [^18^F]SiFA*lin*-TATE, featuring the SiFA synthon SiFA*lin* (N-(4-(di-*tert*-butylfluorosilyl)benzyl)-N,N-dimethyl-4-oxobutan-1-aminium) (Figure 1B).^21–25^ Recently, a potential clickable *Cyclo*SiFA prosthetic group based on an azasilole five-membered ring scaffold has been reported, which might be used in PET tracer development using Cu-catalyzed triazole formation, potentially enabling straightforward linkage to biomolecules on demand.^26^ However, the compound is still highly lipophilic and its implementation into radiotracer scaffolds needs to be further demonstrated.

Despite these important results, the introduction of classical SiFAs into targeted radiotracers, including rh-compounds, is still problematic and can lead to drawbacks such as diminished receptor binding affinity and the aforementioned high lipophilicity. Within this framework, we report on the design and synthesis of a bifunctional SiFA building block, namely **Fmoc-(SiFA)SeFe** (3-(((((9H-fluoren-9-yl)methoxy)-carbonyl)amino)methyl)-5-(di-*tert*-butylfluorosilyl)benzoic acid) (Figure 1B), which is endowed with higher hydrophilicity and can be incorporated into a radiopharmaceutical construct both terminally and bridging two moieties.

**Figure 2:**
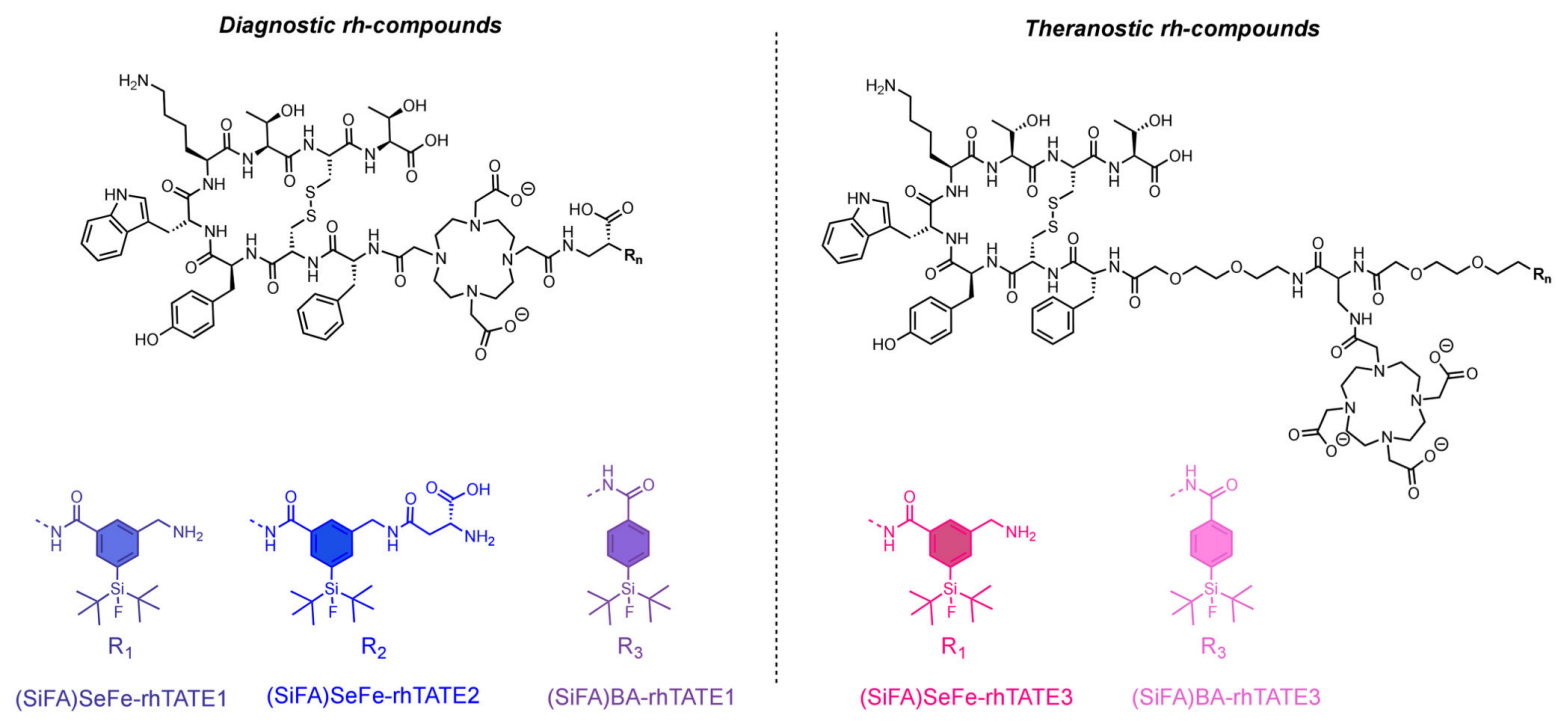
Structures of sstR2-targeting rh-compounds containing a SiFA building block diagnostic rh-compounds **(SiFA)SeFe-rhTATE1, (SiFA)SeFe-rhTATE2** and (SiFA)BA analogue **(SiFA)BA-rhTATE1** (left), as well as theranostic rh-compounds **(SiFA)SeFe-rhTATE3** and (SiFA)BA analogue **(SiFA)BA-rhTATE3** (right) are shown.

Initially, **(SiFA)SeFe** was conjugated to model peptides to assess its stability to defluorination under different conditions (i.e., physiological conditions, stability toward reverse isotopic exchange, and lutetium-labeling conditions). Further, to prove the practical application of the new SiFA building block, the synthesis of **(SiFA)SeFe**-containing radiohybrid tracers was performed based on the clinically most relevant sstR2-targeting octapeptide [^nat/68^Ga]Ga-DOTA-TATE (DOTA = 1,4,7,10-tetraazacyclododecane-1,4,7,10-tetraacetic acid, TATE = (Tyr^3^)-octreotate) for neuroendocrine tumors.^28^ In order to determine optimum radiolabeling conditions/biodistribution/etc., we have included the novel building block **(SiFA)SeFe** at different positions in a series of rh-constructs, using DOTA as a general chelator for different radiometals for both imaging or therapy.^29^ Thus, three compounds were obtained featuring the targeted sstR2 binding ligand TATE, a DOTA chelator (for Ga^3+^, Lu^3+^) and **(SiFA)SeFe**. The new SiFA group was initially inserted either in a terminal or bridging position - in between the TATE-DOTA scaffold and a negatively charged diamino propionic acid group - to obtain **(SiFA)SeFe-rhTATE1** and **(SiFA)SeFe-rhTATE2**, respectively, (Figure 2). It should be noted that these two compounds can enable purely diagnostic radiohybrids, for example based either on ^18^F or ^68^Ga, whereby the radionuclide can be selected depending on site specific availability, cost and suitable infrastructure. The diagnostic rh-concept has successfully reached FDA approval with [^18^F]-rhPSMA-7.3 (POSLUMA)^30^ for PET imaging of prostate cancer.

Most importantly, a theranostic compound was also synthesized, featuring a “branched” DOTA moiety, enabling the heptacoordination to lutetium-177 for therapy, and the **(SiFA)SeFe** in terminal position (**SiFA)SeFe-rhTATE3** (Figure 2). To gain further insights into the effects of the **(SiFA)SeFe** moiety on the chemico-physical properties of the resulting rh-construct, the compounds **[^nat^Ga]Ga-(SiFA)BA-rhTATE1** and **[^nat^Lu]Lu-(SiFA)BA-rhTATE3** featuring the classical (SiFA)BA (4-(di-*tert*-butylfluorosilyl)benzoic acid)^27^ group were also synthesized (Figure 2) and evaluated for comparison. The compounds were characterized by different methods, including multinuclear nuclear magnetic resonance spectroscopy (^1^H-, ^13^C-, ^19^F-, ^29^Si-NMR), RP-HPLC (reverse phase high-performance liquid chromatography) and ESI-MS (electrospray ionization mass spectrometry), and a protocol for radiolabeling with [^18^F]fluoride or [^177^Lu]lutetium has been developed.

Further, the *in vitro* properties of the gallium or lutetium complexed ligands, including sstR2 binding affinity, lip-ophilicity, human albumin binding, as well as stability in human serum were assessed. The obtained results have been discussed in comparison to the FDA approved benchmark [^68^Ga]Ga-DOTA-TATE and [^177^Lu]Lu-DOTA-TATE, and the well-known ^18^F-labeled SST-analogue for NETs [^19/18^F]-SiFA*lin*-TATE. One of the newly developed tracers, namely **[^18^F][^nat^Ga]Ga-(SiFA)SeFe-rhTATE2**, was studied *in ovo* in AR42J and U87 tumors engrafted on the chicken chorioallantoic membrane (CAM) model. This model is time- and cost-effective, and provides the possibility for high-throughput screening, while complying with the principles of the 3Rs (Replacement, Reduction, Refinement), allowing for fast and unsophisticated ligand screening and collection of preliminary data.^31,32^ Despite these advantages, the chick CAM is a non-mammalian complementary model to classical mouse xeno-grafts to assess compound’s pharmacokinetics and metabolism.^33,34^ Consequently, the biodistribution of two compounds representative of each family of diagnostic and theranostic rhtracers - **[^18^F][^nat^Ga]Ga-(SiFA)SeFe-rhTATE1** and **[^18^F]-[^nat^Lu]Lu-(SiFA)SeFe-rhTATE3** - were investigated *in vivo* in AR42J tumor-bearing CD1-nu/nu mice.

## Results and Discussion

### Synthesis of (SiFA)SeFe

**Scheme 1:**
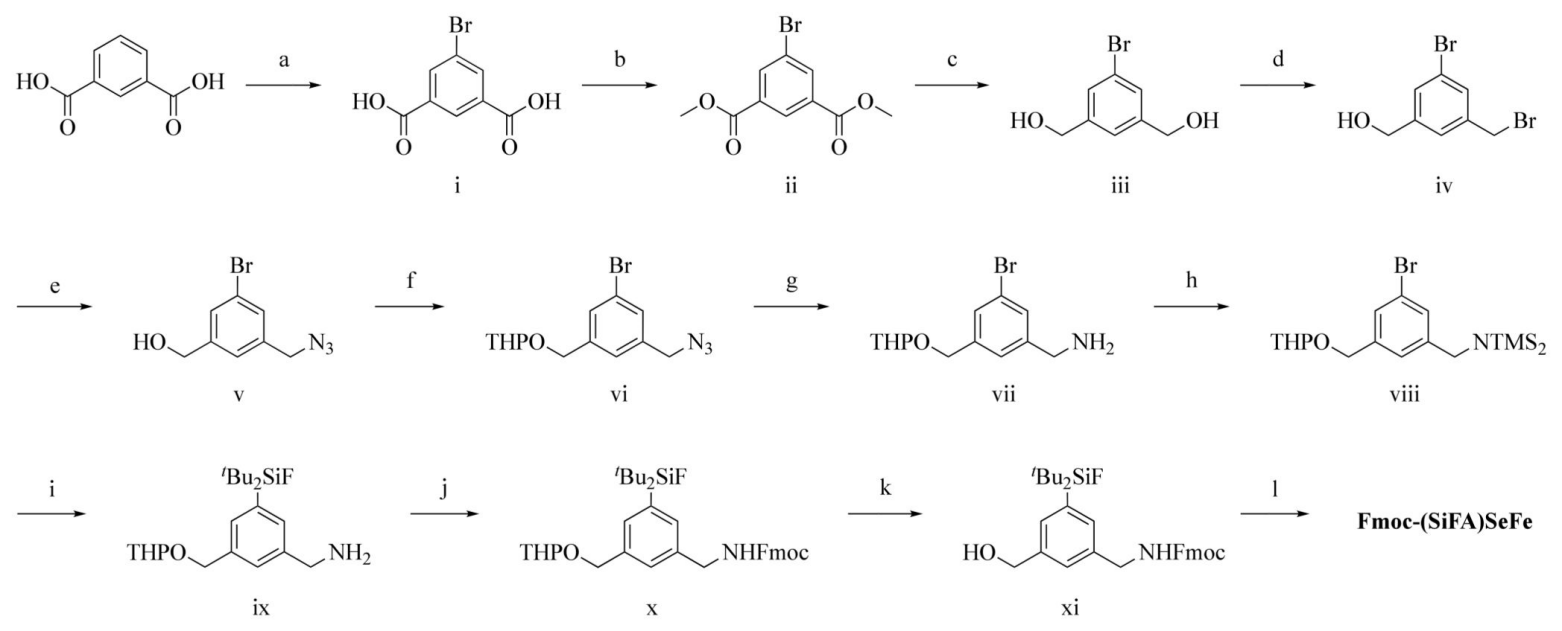
Synthesis of Fmoc-(SiFA)SeFe^*a*^ ^*a*^Reagents and conditions: a) Dibromantin (H_2_SO_4_), 60 °C, 3 h, 100%; b) MeOH, H_2_SO_4_, 70 °C, 16 h, 86.9%; c) LiAlH_4_ (THF), RT, 16 h, 70.8%; d) HBr (toluene), 60 °C, 16 h, 77.5%; e) NaN_3_ (acetone/H_2_O), 60 °C, 1 h, 100%; f) DHP, *p*-TsOH (DCM), RT, 1 h, 100%; g) PPh_3_ (THF/H_2_O), 70 °C, 1 h, 66.2%; h) TMSCl, TEA (DCM), RT, 16 h, 77.9%; (i) ^*t*^BuLi, ^*t*^Bu_2_SiF_2_, H_2_O (THF), RT, 16 h, 45.8%; j) FmocCl (THF/*i*PrOH), RT, 0.5 h, 42.7%; k) HCl (THF/MeOH), RT, 16 h, 50.8%; l) TEMPO, NaClO_2_, NaOCl (MeCN), 40 °C, 2 h, 76.6%.

The main challenge of the synthesis of the new SiFA was to achieve a trifunctional aromatic system. The crucial steps included introduction of an amine, introduction of the silicon moiety via umpolung with ^*t*^BuLi and oxidation to a carboxylic acid (Scheme 1, see Experimental section for details and characterization of the compounds, Figures S1−S15). In detail, starting from inexpensive isophthalic acid, 5-Bromo isophthalic acid **i** was obtained in quantitative yield. Further, esterification to dimethyl 5-bromoisophthalate (**ii**) was performed, followed by reduction (**iii**) and selective monobromination (**iv**). The bromoalcohol **iv** was reacted with sodium azide to get the azido-alcohol **v** in quantitative yields in 1 h followed by simple purification by extraction. Previous synthesis of similar compounds with a free benzylic alcohol and amine required elaborate purification, so to prevent this, the hydroxyl group was protected with tetrahydropyran (THP) to achieve **vi** prior amine formation via the Staudinger reduction with triphenyl-phosphine and water. The protected amino alcohol **vii** was obtained in good yield (71.4%). The following protection of the amine with trimethylsilyl chloride (TMSCl) showed no interference with the THP group and yielded **viii** (77.9%). Since there are no suitable protecting groups for carboxylic acids against ^*t*^BuLi, the oxidation of the respective alcohol had to occur after the introduction of ^*t*^Bu_2_SiF_2_. From previous attempts, it was also clear that a mild oxidation was necessary because of the susceptibility of the protected amine. The introduction of ^*t*^Bu_2_SiF_2_ onto **viii** was performed with ^*t*^BuLi, but the standard acidic aqueous workup was instead performed at pH 8−9 to prevent the cleavage of the THP-group. The free amine **ix** was obtained in moderate yield (45.8%). In order to oxidize the alcohol group, the amine had to be again protected. To this aim, the fluorenylmethoxycarbonyl (Fmoc) protecting group was chosen for future use in solid phase peptide synthesis (SPPS) to obtain the double protected compound **x** in moderate yield (42.7%). Following acidic deprotection of THP to obtain **xi** (33.5%), the oxidation with the mild (2,2,6,6-tetramethylpiperidin-1-yl)oxyl (TEMPO) reagent was chosen to get the desired product **Fmoc-(SiFA)SeFe** in good yield (69.8%). The product was purified by flash chromatography and obtained in 12 steps with an average yield of 74.9% per step. While **Fmoc-(SiFA)SeFe** can be synthesized in gram scale, the 12-steps cumulative yield is very poor (ca. 2−3%) and in-future, further optimization of the synthetic protocol is required. The compound was characterized by different analytical and spectroscopic methods including ^1^H-, ^13^C-, ^19^F- and ^29^Si NMR spectroscopy (Figure S1−S15) and high-resolution electrospray mass spectrometry (HR-ESI-MS) (Figure S16).

### Stability Studies with Model (SiFA)SeFe Bioconjugate Peptides

The **(SiFA)SeFe** moiety was initially conjugated to model peptides to assess its stability to defluorination depending on its positioning in the peptide sequence (terminal or bridged) and under different conditions (i.e., physiological conditions, stability toward reverse isotopic exchange, and lutetium-labeling conditions). Therefore, nine compounds were obtained featuring positively (L-Lysine) and negatively (L-Aspartic acid or L-Glutamic acid) charged or bulky-neutral (L-Tyrosine) amino acids attached directly or via a Glycine linker to **(SiFA)SeFe** (see Figures S17A). Specifically, three peptides had the **(SiFA)SeFe** group positioned at the terminus (compounds **1X_n_**, n = 1, 2, 3, Figure S17A), while the other six had it bridged via amide bond formation to either sides (**2X_n_** and **3X_n_**). For comparison, three model peptides containing the benchmark SiFA *para*-(SiFA)BA were also evaluated (compounds **4X_n_**, Figure S17A). See Figures S18−S188 for the full characterization. First, the radiochemical conversion (RCC) of the ^18^F-labeling was determined by Radio-TLC (Figure S17B, Table S1). With the exception of the terminal Gly-Asp bearing bioconjugate **1X_2_**, the RCCs and RCYs of the model **(SiFA)SeFe**-bioconjugates were lower than the *para*-(SiFA)BA ones (**4X_n_**); likely to be attributed to the *meta* position of the SiFA group. Additionally, the bridged model peptides (**2X_n_** and **3X_n_**) showed generally lower RCCs/RCYs than the terminal ones (**1X_n_**), likely due to their higher steric hindrance.

The stability of the model ^18^F-labeled bioconjugates was further investigated in PBS buffer (pH 7.4) at 37 °C over 2 h (Figure S17C, Table S2). Notably, the constructs bearing the negatively charged aspartate (compounds **1−3X_2_**) were the most stable within their respective series, and markedly more stable than the *para*-(SiFA)BA compounds (**4X_n_**). Next, the stability of the bioconjugates was evaluated in the conditions used for ^177^Lu-labeling (pH 5.5 and 90 °C) (Figure S17D, Table S2). Compared to the already stable benchmark *para*-(SiFA)BA, all **(SiFA)SeFe**-bioconjugates showed higher stability (up to 10-fold, 371% average increase). Based on these promising data, we prepared rh-constructs featuring the new **(SiFA)SeFe** moiety either in a terminal (**(SiFA)SeFe-rhTATE1** and **(SiFA)SeFe-rhTATE3**, Figure 2) or bridged position (**(SiFA)SeFe-rhTATE2**). Of note, the latter tracer was designed based on the observed beneficial effect of the negatively charged Gly-Asp group on both the RCC and stability of the model bioconjugates in physiological conditions.

### Synthesis of sstR2 Targeted (SiFA)SeFe rh-Compounds

For an initial proof-of-concept study, the rh-ligands **(SiFA)SeFe-rhTATE1**, (**SiFA)SeFe-rhTATE2** and **(SiFA)-SeFe-rhTATE3** were synthesized via standard Fmoc-SPPS strategy using a 2-chlorotrityl chloride (2-CTC) resin (see SI for details, Schemes S1−S2, and full characterization, Figures S189−S209). While **(SiFA)SeFe-rhTATE1/2** were envisaged for purely diagnostic purposes based on the high-affinity ligand [^nat/68^Ga]Ga-DOTA-TATE as the lead structure,^28^ compound **(SiFA)SeFe-rhTATE3** was designed for theranostic applications, being suitable for either ^18^F- or ^177^Lu-labeling. Moreover, the structures of **(SiFA)SeFe-rhTATE1** and **(SiFA)SeFe-rhTATE3** were designed to investigate the properties of the radiotracer when the new SiFA building block was used terminally. Instead, **(SiFA)SeFe-rhTATE2** was designed as representative scaffold for a bridged use of the **(SiFA)SeFe** building block, which provides advantages over the classical SiFA groups. To obtain a direct comparison with the commonly used monofunctional **(SiFA)BA** building block, which can only be used terminally, compounds **(SiFA)BA-rhTATE1** and **(SiFA)BA-rhTATE3** were also designed to be the structural analogues of **(SiFA)SeFe-rhTATE1** and **(SiFA)-SeFe-rhTATE3**, respectively.

For the purely diagnostic rh-compounds, the linker unit 2,3-diaminopropionic acid (Fmoc-D-Dap-O^*t*^Bu·HCl) was bridged via an amide bond to the free carboxylic acid group, which was located distal to DOTA-TATE, and then the respective SiFA building block was conjugated under standard conditions. After cleavage from the resin and removal of all protecting groups, the HPLC-purified precursors were obtained in overall yields of 2−3%. The selected amino acid linker Fmoc-D-Dap-O^*t*^Bu· HCl in the compounds **(SiFA)SeFe-rhTATE1/2** increased the distance from the binding motif and introduced a negative charge (deprotonated state), which has been shown in the aforementioned studies with model peptides to increase the stability of **(SiFA)SeFe**. Moreover, since **(SiFA)SeFe** can be used bridged, the amino acid Fmoc-D-aspartic acid-*α-tert*-butyl ester (Fmoc-D-Asp-O^*t*^Bu) was also coupled terminal in the case of **(SiFA)SeFe-rhTATE2**. In this way, an additional negative charge was introduced in the direct proximity of the **(SiFA)SeFe**, hoping for a positive effect on the overall lipophilicity.^21,35^ Further, [^nat^Ga]gallium incorporation was achieved as reported in the experimental section.

Regarding the synthesis of the theranostic compound **(SiFA)SeFe-rhTATE3**, the linker unit Fmoc-O_2_Oc-OH (8-amino-3,6-dioxaoctanoic acid) was incorporated between the pharmacophore TATE and the trivalent linker Fmoc-D-Dap(Dde)-OH. The polyethylene glycol-like linker Fmoc-O_2_Oc-OH from SiFA*lin*-TATE was selected to maintain good affinity to sstR2. Subsequently, the chelator DOTA was placed at the side chain of Fmoc-D-Dap(Dde)-OH to enable stable complexation of the therapeutic isotope by providing three carboxylic acid groups for coordination. After conjugation of another Fmoc-O_2_Oc-OH linker, **(SiFA)SeFe** was incorporated at a terminal position. Afterward, the incorporation of [^nat^Lu] lutetium was conducted (Figure S206).

### Radiolabeling

Radiolabeling of SiFA moieties with [^18^F]fluorine was carried out according to a slightly modified procedure from the literature.^36^ The ionic exchange reaction (IE) was achieved ≤10 min at RT. In detail, the required amount of fluoride-18 (0.2−2.0 GBq in [^18^O]H_2_O) was fixed on a Sep Pak Light (46 mg) Acell Plus QMA Carbonate cartridge and dried with 8 mL of DMSO (anhydrous). The loaded cartridge was then eluted with 150 *μ*L of NH_4_HCOO in DMSO (1 M) onto 30.0 *μ*L of the respective SiFA-conjugated peptide precursor in DMSO (1 mM, 30.0 nmol). After 10 min at RT, the reaction mixture was quenched with H_2_O (10 mL) (see experimental for details). After separation of free [^18^F]fluoride by solid phase extraction (SPE), the time for the whole labeling process was <30 min, demonstrating the fast and efficient ^18^F-fluorination of the new SiFA building block. Compared to the reference [^18^F]SiFA*lin*-TATE (RCC = 60%), the three sstR2-targeted ligands containing the **(SiFA)-SeFe** building block showed comparable RCC (54%, 60% and 63% respectively). In contrast, labeling of the **(SiFA)BA** building block showed lower RCCs (39%−54%, Table S3), indicating more efficient labeling of SiFA*lin* and (**SiFA)SeFe**. The RCYs were slightly reduced compared to [^18^F]SiFA*lin*-TATE (59%). Nevertheless, all **(SiFA)SeFe** derivatives showed satisfactory RCY in the range 36−47%. All ^18^F-compounds could be obtained in high radiochemical purities (RCP_HPLC_ = 94−99%, RCP_TLC_ = 98−99%, Table S3, and supplementary Figures S210−S225).

The reaction conditions for the ^177^Lu-labeling of **(SiFA)-SeFe-rhTATE3, (SiFA)BA-rhTATE3** and DOTA-TATE were optimized by varying the temperature, reaction time and ligand concentration, and eventually set to 70 °C for 5 min (1 nmol ligand) (Figure S226, S227, and S229). Under these conditions, very high RCYs and RCPs (radio-RP-HPLC) of 97% or higher, could be achieved, which were confirmed using TLC (≥99%) (see SI for details, Table S3). In comparison to other ^177^Lu-labeling methods, for example for PSMA- or CCK-addressing radioligands, which require reaction times of 20−30 min and temperatures of up to 90 °C to consume free [^177^Lu]lutetium(III),^36–38^ this represents a substantial improvement. This is important as free [^177^Lu]lutetium(III) results in bone accumulation, mimicking calcium(II)ion uptake and leading to unnecessary radiation exposure of nontarget tissue.^39,40^

### *In Vitro* Evaluation

**Figure 3:**
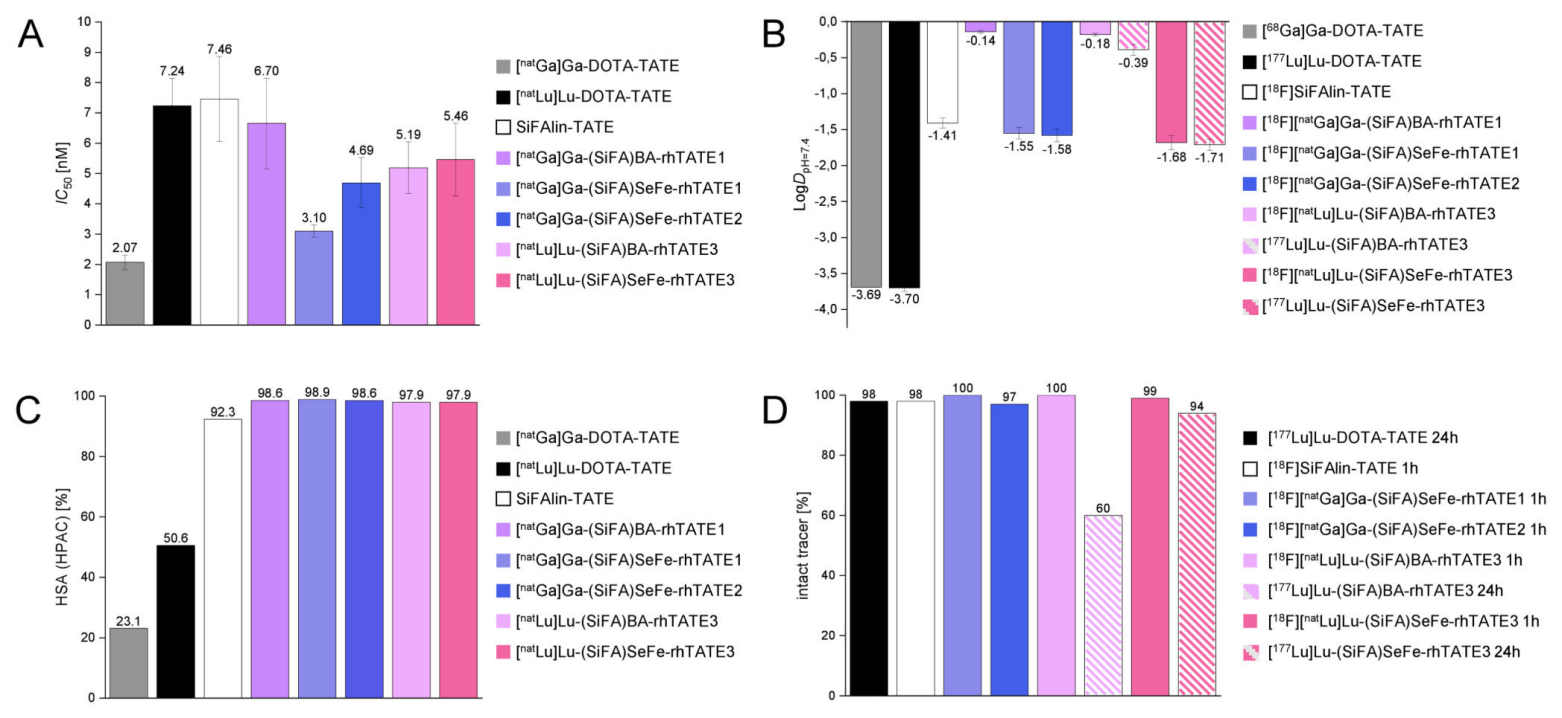
*In vitro* evaluation of the new rh-compounds. A) sstR2 receptor binding affinity (*IC*_50_) of the non-radiolabeled rh-compounds evaluated by competitive binding assay with [^125^I]I-TOC; B) lipophilicity (log*D*_pH=7.4_) of the ^18^F- or ^177^Lu-labeled compounds, C) human serum albumin binding (HSA) of the non-radiolabeled compounds assessed by the high performance affinity chromatography (HPAC) method; and D) stability of the radiolabeled compounds in comparison to the benchmarks ([^18^F]SiFA*lin*-TATE, [^nat/68^Ga]Ga-DOTA-TATE, and [^nat/177^Lu]Lu-DOTA-TATE) in human serum at 37 °C after 1 or 24 h.

The sstR2-addressing ligands were evaluated in *in vitro* experiments and compared with the clinical standards [^18^F]SiFA*lin*-TATE, [^68^Ga]Ga-DOTA-TATE and [^177^Lu]Lu-DOTA-TATE as well as the radiolabeled benchmark ligands **(SiFA)BA-rhTATE1** and **(SiFA)BA-rhTATE3**. The studies included determination of binding affinity to sstR2-expressing CHO_sst2_ cells (Chinese hamster ovary (CHO) cells stably transfected with human sstR2 (epitope-tagged at the *N*-terminal end)), lipophilicity, human serum albumin binding, and human serum stability (Figure 3, Table S4).

To determine the binding affinity toward sstR2, the half-maximal inhibitory concentration (*IC*_50_) was examined in a competitive binding assay using CHO_sst2_ cells, in which [^125^I]TOC was used as the competitor. Compared to the references [^nat^Ga]Ga-DOTA-TATE (*IC*_50_ = 2.07 ± 0.24 nM), [^nat^Lu]Lu-DOTA-TATE (*IC*_50_ = 7.24 ± 0.9 nM) and [^nat^F]SiFA*lin*-TATE (*IC*_50_ = 7.46 ± 1.40 nM), all model ligands showed comparable high affinities with *IC*_50_ values in the range of 3−5 nM, with **[^nat^Ga]Ga-(SiFA)SeFe-rhTATE1** being the closest to [^nat^Ga]Ga-DOTA-TATE (Figure 3A).

The lipophilicity of all compounds was determined as octanol-PBS partition coefficient at pH = 7.4 (log*D*_pH=7.4_) by the shake flask method. Compounds [^68^Ga]Ga-DOTA-TATE and [^177^Lu]Lu-DOTA-TATE exhibited the most hydrophilic character with a log*D*_pH=7.4_ value of −3.69^41^ and −3.70 ± 0.05, respectively (Figure 3B). All the **(SiFA)SeFe** containing ligands featured a high hydrophilicity (log*D*_pH=7.4_ = −1.55 to −1.71) comparable to [^18^F]SiFA*lin*-TATE (log*D*_pH=7.4_ = −1.41 ± 0.07), and were markedly more hydrophilic (ca. 11-fold) than the respective (SiFA)BA benchmarks^42^ (Figure 3B), demonstrating the advantage of using the new **(SiFA)SeFe** moiety. Considering the primarily renal excretion of [^18^F]-SiFA*lin*-TATE in patients,^22^ these data also point toward a similar behavior of the **(SiFA)SeFe** containing ligands *in vivo*.

High performance affinity chromatography (HPAC) was used to determine representative values for HSA binding of the ligands and their references, since it can influence the distribution and pharmacokinetics of radiopharmaceuticals.^43,44^ All the compounds **[^nat^Ga]Ga-(SiFA)SeFe-rhTATE1, [^nat^Ga]Ga-(SiFA)SeFe-rhTATE2, [^nat^Lu]Lu-(SiFA)SeFe-rhTATE3, [^nat^Ga]Ga-(SiFA)BA-rhTATE1** and **[^nat^Lu]Lu-(SiFA)BA-rhTATE3** showed an almost identical high binding to HSA of 98−99% (Figure 3C), and comparable to SiFA*lin*-TATE (92%). Markedly reduced HSA binding was observed for the more hydrophilic [^nat^Ga]Ga-DOTA-TATE and [^nat^Lu]Lu-DOTA-TATE (23% and 51%, respectively), as expected.

Stability studies in human serum were also performed by incubating the ^18^F-labeled ligands **[^18^F][^nat^Ga]Ga-(SiFA)-SeFe-rhTATE1, [^18^F][^nat^Ga]Ga-(SiFA)SeFe-rhTATE2, [^18^F][^nat^Lu]Lu-(SiFA)SeFe-rhTATE3** and **[^18^F][^nat^Lu]Lu-(SiFA)BA-rhTATE3** (for 1 h), as well as the ^177^Lu-labeled ligands **[^177^Lu]Lu-(SiFA)SeFe-rhTATE3** and **[^177^Lu]Lu-(SiFA)BA-rhTATE3** for 1 and 24 h at 37 °C (Figure 3D). Afterward, the samples were analyzed for the intact tracer by radio-RP-HPLC (see SI, Table S4). While **[^18^F][^nat^Ga]Ga-(SiFA)SeFe-rhTATE1** exhibited high stability with ≥99% intact tracer, **[^18^F][^nat^Ga]Ga-(SiFA)SeFe-rhTATE2** showed minor decomposition (97 ± 1.3% intact tracer) over 1 h. With regard to the diagnostic application, the ^18^F-labeled radio-hybrids **[^18^F][^nat^Lu]Lu-(SiFA)SeFe-rhTATE3** and **[^18^F]-[^nat^Lu]Lu-(SiFA)BA-rhTATE3**, as the diagnostic reference [^18^F]SiFA*lin*-TATE, showed no degradation after 1 h incubation in human serum (≥98% intact tracer^24^).

For the therapeutic applicability, the reference ligand [^177^Lu]Lu-DOTA-TATE featured high stability of 98 ± 3% intact tracer after 24 h of incubation. Interestingly, while **[^177^Lu]Lu-(SiFA)SeFe-rhTATE3** also showed high stability (94 ± 3.0%), the analogue compound **[^177^Lu]Lu-(SiFA)BA-rhTATE3** had reduced stability, with 60 ± 1.0% tracer intact after 24 h (see SI, Table S4). Based on the radio-RP-HPLC chromatograms, it can be concluded that decomplexation of [^177^Lu]lutetium(III) takes place. Moreover, another species at a slightly shorter retention time could be observed in the case of **[^177^Lu]Lu-(SiFA)BA-rhTATE3**, which could not be identified. Overall, the direct comparison between the radiohybrids shows the superiority of **(SiFA)SeFe** compared to **(SiFA)BA** in terms of stability.

### *In Ovo* Evaluation

**Figure 4:**
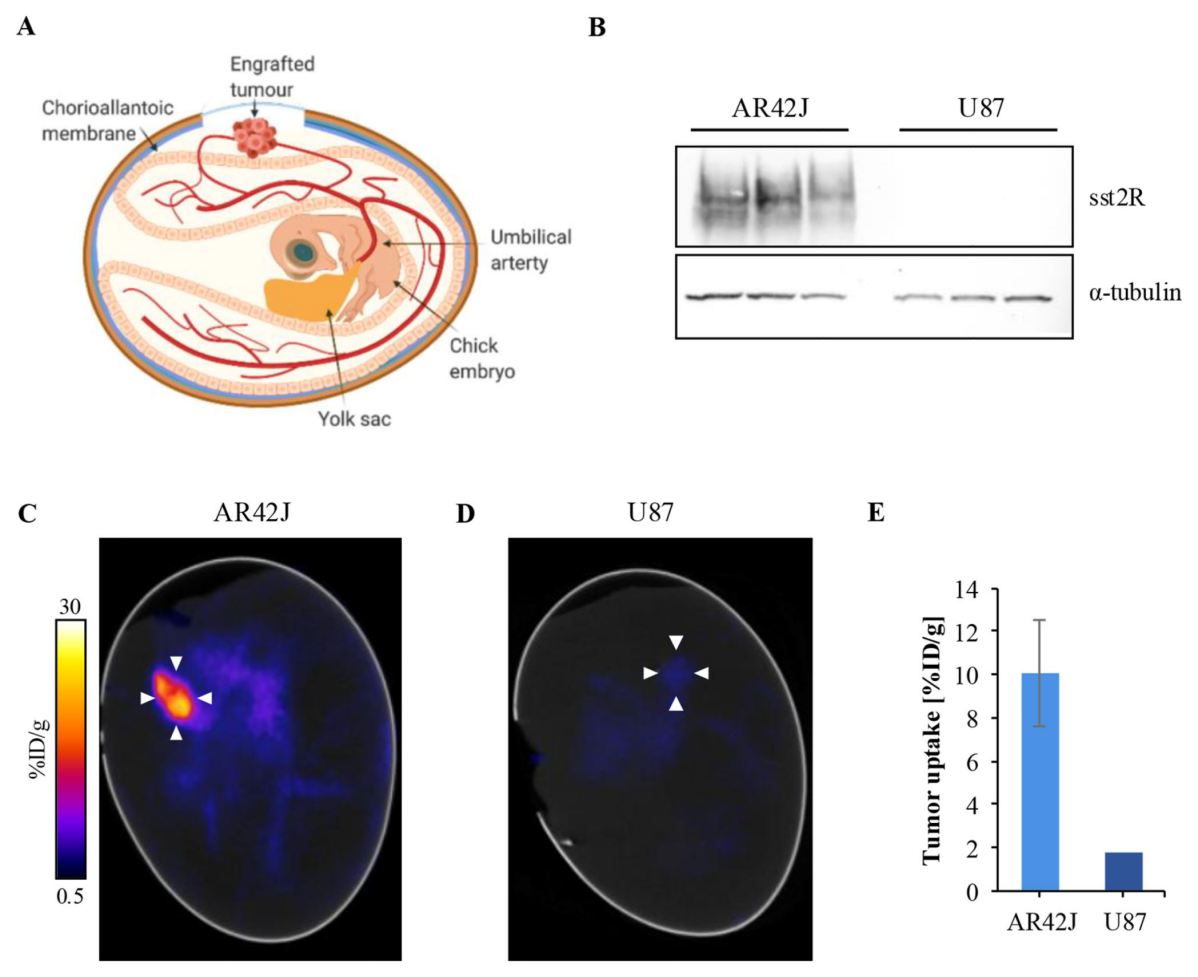
Specific *in ovo* uptake of [^18^F][^nat^Ga]Ga-(SiFA)SeFe-rhTATE2 in sstR2-expressing AR42J tumors in the chick CAM model. A) Schematic overview of the CAM model and associated tumor growth. B) sstR2 protein expression in AR42J and U87 cell lysates. Uncropped images of the gels are available in the Supporting Information (Figure S230). C) Representative *in ovo* PET/CT image of the **[^18^F][^nat^Ga]Ga-(SiFA)SeFe-rhTATE2** tracer uptake into sstR2-positive AR42J tumor-bearing chick CAM model 40−60 min p.i. (*n* = 4). White arrows indicate the tumor. D) Representative *in ovo* PET/CT image of the tracer uptake in a sstR2-negative U87 tumor chick CAM model 40−60 min p.i. (*n* = 1). White arrows indicate the tumor. E) Quantification of tracer uptake in AR42J and U87 tumors.

Recently, we have refined the chick CAM model (Figure 4A) for precision tumor imaging, providing images of similar quality to *in vivo* mouse xenografts.^34^ Here, as a proof of concept, we aimed to visualize the sstR2-specificity of one of the new tracers, **[^18^F][^nat^Ga]Ga-(SiFA)SeFe-rhTATE2**, *in ovo*. Besides the sstR2-expressing human pancreatic AR42J cells, the non-sstR2-expressing human glioblastoma U87 cell line was selected as negative control, with differences in sstR2 protein expression between the two cell lines confirmed by Western blot (Figure 4B). The final PET/CT image (1 h post injection (p.i.)) is shown in Figure 4C, depicting high tracer uptake in the AR42J engrafted tumor (10.1 ± 2.5 %ID/g). Furthermore, negligible uptake (ca. 1.8 %ID/g) into the U87 tumor was observed, further supporting the sstR2-specificity of **[^18^F][^nat^Ga]Ga-(SiFA)-SeFe-rhTATE2** (Figure 4D,E).

### *Ex Vivo* Biodistribution Studies

**Figure 5:**
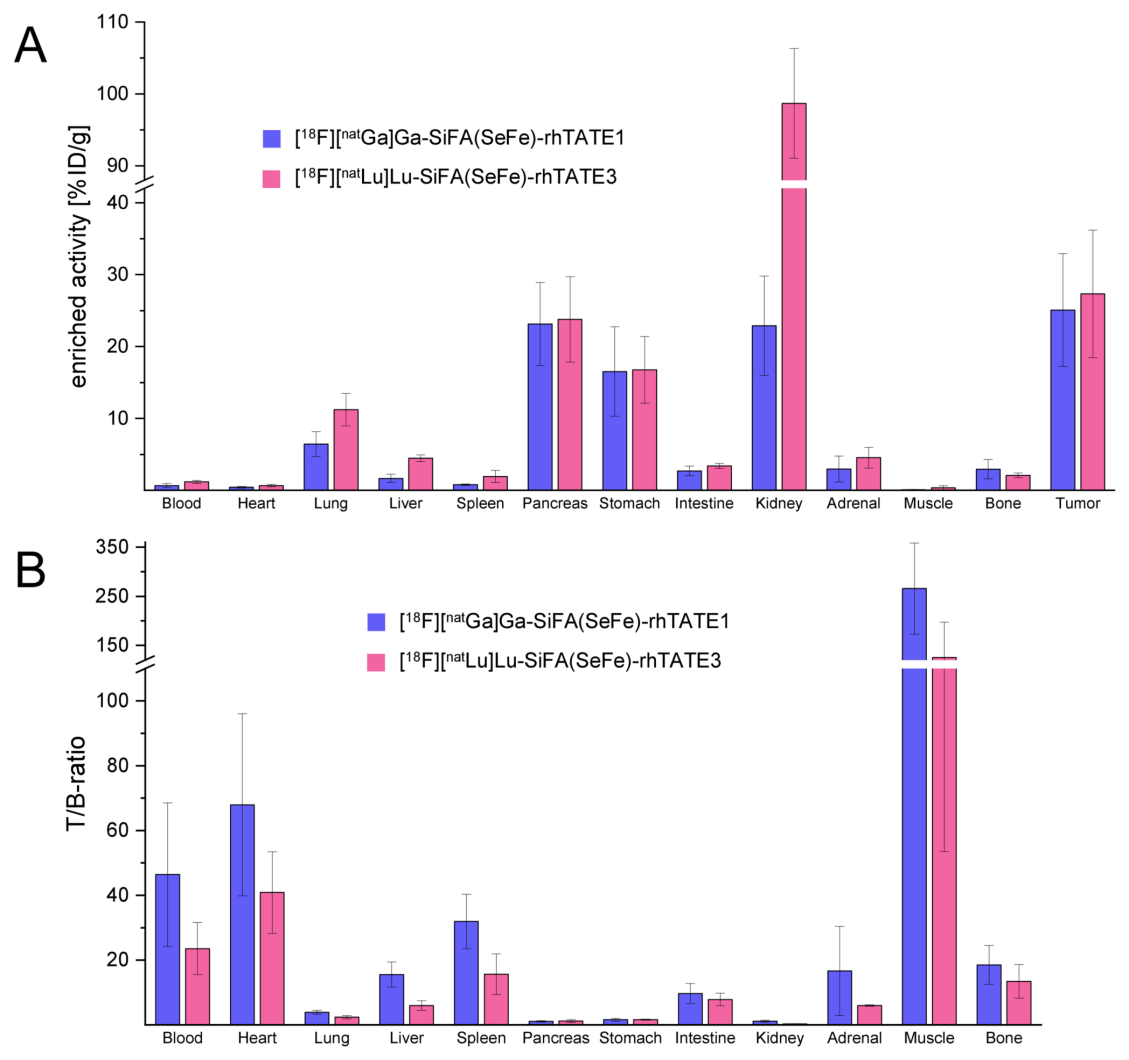
*In vivo* evaluation of [^18^F][^nat^Ga]Ga-(SiFA)SeFe-rhTATE1 and [^18^F][^nat^Lu]Lu-(SiFA)SeFe-rhTATE3. A) *Ex vivo* biodistribution and B) respective tumor to background (T/B) ratios of **[^18^F][^nat^Ga]Ga-(SiFA)SeFe-rhTATE1** and **[^18^F][^nat^Lu]Lu-(SiFA)SeFe-rhTATE3** (300 pmol per mouse) in selected organs after 1 h post injection (p.i.) in AR42J tumor-bearing CD1-nu/nu mice. Data are expressed as %ID/g, mean ± SD (*n* = 3). The exact values calculated for this diagram are given in the SI (Table S5−S6).

Further, *ex vivo* biodistribution studies in tumor-bearing mice were performed on two rh-compounds, **[^18^F][^nat^Ga]Ga-(SiFA)SeFe-rhTATE1** and **[^18^F][^nat^Lu]Lu-(SiFA)SeFe-rhTATE3**, representative of the diagnostic and theranostic families of compounds, respectively. The ^18^F-labeled compounds were assessed in AR42J tumor-bearing CD1-nu/nu mice after 1 h p.i. (Figure 5) (see SI for details, Table S5). After this time, radioactivity levels of 25.1 ± 7.8 %ID/g were measured for **[^18^F][^nat^Ga]Ga-(SiFA)SeFe-rhTATE1** in the AR42J-tumor, which were in the range of those reported for [^18^F]SiFA*lin*-TATE (18.5 ± 4.9 % ID/g)^24^ and for the gold standard [^68^Ga]Ga-DOTATATE (14.1 ± 4.8 %ID/g)^24^ in the same tumor model; whereas uptake in heart, liver, spleen, intestine, adrenal glands, muscle, and bones were low (0.10−2.96 %ID/g). Despite the determined HSA binding of approximately 99%, a beneficial low accumulation in the blood was also measured (0.67 ± 0.28 %ID/g). The low bone uptake also indicated high *in vivo* stability related to defluorination^45^ and is in line with the *in vitro* stability studies performed in human serum. Moderate uptake was seen in the lung (6.4 ± 1.7% ID/g), while pancreas (23.1 ± 5.8 %ID/g), stomach (16.5 ± 6.2 %ID/g) and kidney (22.9 ± 6.9 %ID/g) showed high accumulation of the tracer (Figure 5A). Almost no liver and high kidney accumulation indicated exclusive renal excretion. This is also attributable to the hydrophilic nature of **[^18^F][^nat^Ga]Ga-(SiFA)SeFe-rhTATE1** (log*D*_pH=7.4_ = 1.55 ± 0.08). Moreover, the high activity levels in the pancreas and stomach were expected due to the endogenous sstR2 expression in these organs.^41^ Although the lung, adrenal glands, and intestine of mice are also known to naturally express low levels of SSTR, they occur at lower densities and should have correspondingly lower accumulations of radioactivity.^41^ In the case of the lung in particular, specificity should be demonstrated in the future using competition studies. To assess the imaging quality of the radiotracer, the tumor-to-background (T/B) ratios were also analyzed (see SI, Figure 5B, Table S6). **[^18^F][^nat^Ga]Ga-(SiFA)SeFe-rhTATE1** showed high T/B ratios for blood, heart, adrenal glands and muscle. Overall, **[^18^F][^nat^Ga]Ga-(SiFA)SeFe-rhTATE1** has a desirable biodistribution, with excellent contrast for tumor imaging.

Concerning **[^18^F][^nat^Lu]Lu-(SiFA)SeFe-rhTATE3**, at 1 h p.i. the compound revealed the highest tumor uptake and low liver uptake (27.3 ± 8.9 and 4.5 ± 0.5 %ID/g, respectively), but very high kidney accumulation (98.9 ± 7.6 %ID/g), while activity levels in the blood and the bone were low (<2 %ID/g) (Figure 5A). The high kidney uptake could be explained by the presence of more positively charged residues.^46,47^ Although high kidney uptake can lead to harmful doses, similar uptake has been observed with the commonly used PSMA ligand PSMA I&T,^48^ which does not necessarily exclude **[^18^F]-[^nat^Lu]Lu-(SiFA)SeFe-rhTATE3** from possible use for clinical imaging. Based on the kidney-to-liver ratio, renal excretion was clearly favored and the low bone uptake confirmed good *in vivo* stability. Moderate radioactivity levels were found in the lung (11.2 ± 2.2 %ID/g), pancreas (23.8 ± 5.9 %ID/g), stomach (16.8 ± 4.6 %ID/g) and adrenal glands (4.5 ± 1.4 %ID/g). The T/B ratios of the theranostic rhTATE derivative (Figure 5B, Table S6) showed a comparable blood clearance with respect to SiFA*lin*-TATE.^24^ **[^18^F][^nat^Lu]Lu-(SiFA)SeFe-rhTATE3** showed high T/B ratios for the blood, heart, spleen, muscle and bone.^49^ Values for the liver, intestine and the adrenal glands were sufficiently low, while the lung, pancreas, stomach and the kidneys exhibited low tumor-to-background ratios as was seen with **[^18^F][^nat^Ga]Ga-(SiFA)SeFe-rhTATE1**.

## Conclusion

In summary, a novel hydrophilic and stably fluorinatable bifunctional SiFA building block (**(SiFA)SeFe**) was synthe-sized, which enables straightforward and versatile linkage to biomolecules (i.e., it can be inserted both terminally and bridged via amide bond formation) to achieve diagnostic (radiohybrid) tracers for PET imaging (^18^F or ^68^Ga labeling), and most importantly, theranostic rh-compounds (^18^F/^177^Lu). The stability of the new SiFA building block to defluorination under different conditions has been initially assessed by incorporating it in model peptides. The obtained results showed in some instances increased stability under physiological conditions, and markedly enhanced stability with respect to lutetium-labeling conditions, when compared to (SiFA)BA analogues.

Thus, as proof-of-concept of the potential of the new SiFA moiety, two diagnostic compounds (**(SiFA)SeFe-rhTATE1** and **(SiFA)SeFe-rhTATE2**) and a further theranostic compound (**(SiFA)SeFe-rhTATE3**) targeting sstR2 were synthesized and fully characterized. In addition, ^18^F and ^177^Lu-labeling protocols were optimized. The gallium or lutetium complexed rh-compounds showed promising *in vitro* results with respect to benchmark tracers. In detail, hydrophilicity was greatly enhanced (ca. 9−11 fold decreased log*D*_pH=7.4_ value) compared to (SiFA)BA rh-analogues and was comparable to the clinically established SiFA*lin*-TATE. Notably, the stability of **(SiFA)SeFe** rh-compounds in human serum was also very high, and in the case of **[^177^Lu]Lu-(SiFA)SeFe-rhTATE3** much higher than the (SiFA)BA analogue over 24 h.

The new rh-tracers also showed outstanding affinity (low nM range) toward sstR2 *in vitro*, comparable to that of the FDA approved [^68^Ga]Ga-DOTA-TATE. The sstR2-targeting ability of **[^18^F][^nat^Ga]Ga-(SiFA)SeFe-rhTATE2** was also successfully validated by PET *in ovo*. Further, the biodistribution of **[^18^F][^nat^Ga]Ga-(SiFA)SeFe-rhTATE1** and **[^18^F]-[^nat^Lu]Lu-(SiFA)SeFe-rhTATE3** was assessed in AR42J tumor-bearing CD1-nu/nu mice 1 h p.i.. *In vivo*, the compounds showed high tumor uptake (up to 27%ID/g) and favorable imaging properties. The stability toward defluorination observed in the model bioconjugates was confirmed by low bone uptake in mice. Overall, the application of the new **(SiFA)SeFe** building block for tumor imaging was successfully demonstrated. Future studies will include the incorporation of this moiety into rh-tracers addressing different targets, such as the chemokine receptor 4 (CXCR4) or the gastrin-releasing peptide receptor (GRPR), to further broaden the scope of peptide-based theranostics.^50^

## Experimental Section

### General

All reagents and solvents were purchased from commercial suppliers and used without further purification. Fluoride-18 in target water ([^18^O]H_2_O) was supplied by *Klinikum rechts der Isar* (Munich, Germany) and by *St Thomas’ Hospital* (London, UK), respectively. [^125^I]Sodium iodide in 40 mM sodium hydroxide solution was purchased from *Hartmann Analytic GmbH* (Braunschweig, Germany). ^1^H-NMR, ^13^C-NMR,^19^F-NMR and ^29^Si NMR spectra were obtained using a Bruker AV300/400/500 Ultra Shield (*Bruker Corporation*, Billerica, Massachusetts, USA). Chemical shifts are given in parts per million (ppm). Abbreviations for NMR multiplications are singlet (s), doublet (d), triplet (t), multiplet (m), and broad (b). The coupling constants *J* are given in Hz. ESI-MS spectra were recorded on an expression^L^ CMS mass spectrometer (*Advion Ltd*., Harlow, UK) with a quadrupole analyzer and an electron spray ionizer. Analytical and preparative RP-HPLC was carried out on *Shimadzu Corp*. Instruments (Kyoto, Japan) equipped with two LC-20AD gradient pumps, a CBM-20A communications module and a Smartline UV detector 2500 (*λ* = 220 nm, *λ* = 254 nm) from *Dr. Ing. Herbert Knauer GmbH* (Berlin, Germany). For analytical RP-HPLC a flow rate of 1.0 mL/min and for preparative RP-HPLC a flow rate of 10 mL/min was used. Quality controls of peptidic ligands were performed on a MultoKrom 100−5-C8 column (150 × 4.6 mm, 5 *μ*m particle size, *CS Chromatographie GmbH*, Langerwehe, Germany). Different gradients of A (H_2_O + 0.1% TFA) and B (MeCN + 5% H_2_O and 0.1% TFA) were used as eluents for all RP-HPLC operations. All compounds are >95% pure by HPLC analysis.

### Synthesis of Fmoc-(SiFA)SeFe

#### 5-Bromoisophthalic Acid (i)

For the aromatic bromination, 50.0 g isophthalic acid (312.2 mmol, 1.0 equiv) and 53.6 g 1,3-Dibrom-5,5-dimethyl-hydantoin (187.3 mmol, 0.6 equiv) were solved in 300 mL concentrated H_2_SO_4_ and stirred for 3 h at 60 °C. After cooling down to RT, the orange emulsion was poured into ice and 100 mL 1m HCl were added. The product was extracted with EtOAc (3 × 200 mL), the combined organic phases were washed with Brine (3 × 100 mL), dried with MgSO_4_ and the solvent was removed under reduced pressure. The product was obtained as a colorless solid in quantitative yield. ^1^H NMR (400 MHz, DMSO-*d*_6_): *δ* 8.41 (s, 1H), 8.24 (s, 2H).

#### Dimethyl 5-Bromoisophthalate (ii)

The esterification was performed by solving 5.0 g of i (20.5 mmol, 1.0 equiv) were in 82 mL MeOH and 4.1 mL H_2_SO_4_ and the solution was stirred at 70 °C for 16 h. After cooling to RT, the solvent was removed under reduced pressure and 100 mL H_2_O and 100 mL DCM were added. The crude product was extracted with DCM (3 × 100 mL) and the combined organic phases were washed with NaHCO_3_ and Brine. The solution was dried via MgSO_4_ and the solvent was removed under reduced pressure and after recrystallization from MeOH the product was obtained as colorless solid (3.89 g, 14.2 mmol, 86.9%).

^1^H-NMR: (400 MHz, Chloroform-*d*) *δ* 8.60 (s, 1H), 8.35 (s, 2H), 3.95 (s, 6 H).

^1^H-NMR: (400 MHz, DMSO-*d*_6_) *δ* 8.41 (s, 1H), 8.30 (s, 2H), 3.90 (s, 6H).

R_f_: 0.48 (10:1, CH/EA).

#### (5-Bromo-1,3-phenylene)dimethanol (iii)

To a stirring solution of 3.4 g LiAlH_4_ in 300 mL dry THF at 0 °C 24.4 g of ii (99.6 mmol, 1.0 equiv) solved in 100 mL dry THF were dropwise added. The slurry solution was stirred at RT for 16 h. The reaction was quenched by adding 400 mL H_2_O carefully. The solution was extracted with Et_2_O (3 × 200 mL), the combined organic phases were washed with brine and water, dried over MgSO_4_ and the solvent was removed under reduced pressure. After flash purification (CH/EA = 1:1 → 100% EA) the product was obtained as colorless needles (15.3 g, 70.5 mmol, 70.8%).

^1^H-NMR: (400 MHz, DMSO-*d*_6_) *δ* 7.35 (s, 2H), 7.24 (s, 1H), 5.31 (s, 2H), 4.48 (s, 4H).

R_f_: 0.56 (100% EA), 0.48 (1:10, CH/EA), 0.18 (1:1, CH/EA)

#### (3-Bromo-5-(bromomethyl)phenyl)methanol (iv)

To 300 mL toluene 40.0 g iii (184.3 mmol, 1.0 equiv) were added. Upon addition of 25.0 mL HBr (48 wt % in H_2_O, 221.2 mmol, 1.2 equiv), the solids dissolved and the solution was stirred o.n. at 60 °C. After cooling to RT, 100 mL NaHCO_3_ were added. The mixture was extracted with Et_2_O (3 × 200 mL), the combined organic phases were dried over MgSO_4_ and the solvent was removed under reduced pressure. After flash purification (100% CH → CH/EA = 1:1) the product was obtained as a colorless solid (40.0 g, 142.9 mmol, 77.5%).

^1^H-NMR: (400 MHz, Chloroform-*d*) *δ* 7.46 (s, 2H), 7.32 (s, 1H),

4.69 (s, 2H), 4.43 (d, *J* = 2.9 Hz, 2H). R_f_: 0.64 (1:1, CH/EA).

#### (3-(Azidomethyl)-5-bromophenyl)methanol (v)

To a solution of 26.04 g iv (92.9 mmol, 1.0 equiv) in 400 mL Aceton/H_2_O (*v/v* = 3:1) 12.07 g NaN_3_ (185.8 mmol, 2.0 equiv) was added and the solution was stirred for 1 h at 60 °C. After complete conversion the volume was reduced by removing the acetone under reduced pressure. The residual solution was extracted with EtOAc (3 × 200 mL) and the combined organic phases were washed with brine (1 × 100 mL) and H_2_O (1 × 200 mL). After drying over NaSO_4_, the solvent was removed under reduced pressure to obtain 22.5 g (92.9 mmol, 100%) of an orange liquid as product.

^1^H-NMR: (500 MHz, Chloroform-*d*) *δ* 7.50 (s, 1H), 7.39 (s, 1H), 7.25 (s, 1H), 4.70 (s, 2H), 4.33 (s, 2H).

R_f_: 0.63 (1:1, CH/EA).

#### 2-((3-(Azidomethyl)-5-bromobenzyl)oxy)tetrahydro-2H-pyran (vi)

A solution of 25.0 g v (103.3 mmol, 1.0 equiv) and 18.7 mL Dihydropyran (206.5 mmol, 2.0 equiv) in 300 mL DCM was cooled to 0 °C and 2.0 g *p*-toluenesulfonic acid (10.3 mmol, 0.1 equiv) was added. The solution was stirred at RT for 1 h. It was added 100 mL brine to the solution and it was extracted with Et_2_O (3 × 100 mL) dried over NaSO_4_ and the solvent and excessive dihydropyran was removed under reduced pressure to obtain the product as 33.7 g (103.3 mmol, 100%) of an orange oil.

^1^H-NMR: (500 MHz, Chloroform-*d*) *δ* 7.49 (s, 1H), 7.38 (s, 1H), 7.23 (s, 1H), 4.76 (d, *J* = 12.5 Hz, 1H), 4.70 (t, *J* = 3.5 Hz, 1H), 4.48 (d, *J* = 12.5 Hz, 1H), 4.33 (s, 2H), 3.60−3.51 (m, 2H), 1.92−1.71 (m, 6H).

R_f_: 0.78 (1:1, CH/EA).

#### (3-Bromo-5-(((tetrahydro-2H-pyran-2-yl)oxy)methyl)phenyl)-methanamine (vii)

In 300 mL of THF/H_2_O (*v/v* = 10/1) 30.2 g vi (92.7 mmol, 1.0 equiv) was solved. The solution was cooled to 0 °C and 29.2 g PPh_3_ (111.2 mmol, 1.2 equiv) was added slowly under gas development. The solution was stirred at 70 °C for 1 h. The solvent was removed under reduced pressure and 100 mL 1M NaOH was added to avoid ammonium ion formation. It was extracted with EtOAc (3 × 100 mL) dried over NaSO_4_ and the solvent was removed under reduced pressure. After 1 h the orange viscous oil solidified and the remaining triphenylphosphine oxide was filtered of by washing with pentane (20 × 50 mL). After removing the solvent under reduced pressure and purification via flash chromatography the product was obtained as 19.9 g (66.2 mmol, 71.4%) of an orange oil.

^1^H-NMR: (400 MHz, Chloroform-*d*) *δ* 7.39 (s, 2H), 7.22 (s, 1H), 4.74 (d, *J* = 12.3 Hz, 1H), 4.70 (m, 1H), 4.45 (d, *J* = 12.3 Hz, 1H), 3.85 (s, 1H), 3.59−3.39 (m, 1H), 1.93−1.62 (m, 6H).

R_f_: 0.40 (20:1, DCM/MeOH).

#### N-(3-Bromo-5-(((tetrahydro-2H-pyran-2-yl)oxy)methyl)benzyl)-1,1,1-trimethyl-N-(trimethylsilyl)silanamine (viii)

To a solution of 19.9 g vii (66.2 mmol, 1.0 equiv) in 400 mL dry DCM, 20.18 mL triethylamine (145.6 mmol, 2.2 equiv) was added. After cooling to 0 °C 16.8 mL trimethylsilyl chloride (132.3 mmol, 2.0 equiv) was added dropwise under precipitation of a colorless solid. The solution was stirred o.n. at RT. The solvent and the excessive TMSCl were removed under reduced pressure and the product was extracted with dry hexane (5 × 100 mL). After removing the solvent under reduced pressure the product was obtained as 22.9 g of an orange oil (51.5 mmol, 77.9%).

^1^H-NMR: (500 MHz, Chloroform-*d*) *δ* 7.31 (s, 1H), 7.30 (s, 1H), 7.19 (s, 1H), 4.74 (d, *J* = 12.4 Hz, 1H), 4.70 (t, *J* = 3.6 Hz, 1H), 4.44 (d, *J* = 12.4 Hz, 1H), 4.07 (s, 2H), 3.57−3.53 (m, 2H), 1.91−1.72 (m, 6H), 0.08 (s, 18H).

#### (3-(Di-tert-butylfluorosilyl)-5-(((tetrahydro-2H-pyran-2-yl)oxy)-methyl)phenyl)methanamine (ix)

For introduction of the silicon center 10.7 g viii (24.1 mmol, 1.0 equiv) were solved in 200 mL dry THF and 31.1 mL ^*t*^BuLi (53.0 mmol, 1.6 m in hexane, 2.2 equiv) was added dropwise at −78 °C. The solution was stirred for 15 min at −78 °C and was then added dropwise to a stirring solution of 5.9 mL ^*t*^Bu_2_SiF_2_ (26.5 mmol, 1.1 equiv) in 100 mL dry THF at −78 °C. The solution was stirred o.n. at RT before adding 200 mL brine and adjust the pH to 8−9 with NaOH. The organic solvent was removed under reduced pressure and it was extracted with Et_2_O (3 × 100 mL), dried over NaSO_4_ and the solvent was removed under reduced pressure. The crude product was purified via flash chromatography to obtain the product as 4.2 g (11.0 mmol, 45.8%) of a yellow solid.

^1^H-NMR: (400 MHz, Chloroform-*d*) *δ* 7.46 (s, 1H), 7.44 (s, 1H), 7.38 (s, 1H), 4.81 (d, *J* = 12.1 Hz, 1H), 4.75−4.67 (m, 1H), 4.52 (d, *J* = 10.8 Hz, 1H), 3.89 (s, 2H), 3.57−3.52 (m, 2H), 1.88−1.79 (m, 6H), 1.06 (s, 18H). R_f_: 0.80 (10:1, DCM/MeOH).

#### (9H-Fluoren-9-yl)methyl-(3-(di-tert-butylfluorosilyl)-5-(((tetrahy-dro-2H-pyran-2-yl)oxy)methyl)benzyl)carbamate (x)

For Fmoc protection 4.5 g ix (11.9 mmol, 1.0 equiv) was solved in 50 mL *i*PrOH and the pH was adjusted to pH 9 with triethylamine. At 0 °C a solution of 3.7 g fluorenylmethyloxycarbonyl chloride (14.3 mmol, 1.2 equiv) in 10 mL THF was added and stirred for 30 min at RT. The solvent was removed under reduced pressure and 50 mL brine was added. The solution was extracted with Et_2_O (3 × 100 mL), dried over NaSO_4_ and the solvent was removed under reduced pressure. The crude product was purified via flash chromatography to obtain the product as 3.1 g (5.1 mmol, 42.7%) of a yellow solid.

^1^H-NMR: (500 MHz, Chloroform-*d*) *δ* 7.77 (t, *J* = 7.7 Hz, 2H), 7.64 − 7.60 (m, 2H), 7.51 (s, 1H), 7.44 (s, 1H), 7.42 − 7.39 (m, 2H), 7.36 (s, 1H), 7.32 − 7.30 (m, 2H), 4.81 (d, *J* = 12.2 Hz, 1H), 4.72 − 4.69 (m, 1H), 4.52 (d, *J* = 12.4 Hz, 1H), 4.44 (s, 2H), 4.43 (s, 2H), 4.24 (t, *J* = 7.0 Hz, 1H), 3.57 − 3.46 (m, 2H), 1.90 − 1.65 (m, 6H), 1.05 (s, 18H).

R_f_: 0.22 (5:1, CH/EA).

#### (9H-Fluoren-9-yl)methyl-(3-(di-tert-butylfluorosilyl)-5-(hydroxymethyl)benzyl)carbamate (xi)

For THP deprotection 3.7 g of x (6.1 mmol, 1.0 equiv) was solved in 50 mL 1 m HCl and 50 mL MeOH. After stirring o.n. at RT, 50 mL NaHCO_3_ were added. The solution was extracted with DCM (3 × 100 mL), dried over Na_2_SO_4_ and the solvent was removed under reduced pressure. The crude product was purified via flash chromatography to obtain the product as 1.6 g (3.1 mmol, 50.8%) of a yellow solid.

^1^H-NMR: (500 MHz, Chloroform-*d*) *δ* 7.76 (d, *J* = 7.6 Hz, 2H), 7.60 (d, *J* = 7.5 Hz, 2H), 7.50 (s, 1H), 7.44 (s, 1H), 7.40 (t, *J* = 7.5 Hz, 2H), 7.37 (s, 1H), 7.31 (t, *J* = 7.4 Hz, 2H), 4.71 (s, 2H), 4.44 (m, 4H), 4.24 (t, *J* = 6.9 Hz, 1H), 1.05 (s, 18H).

R_f_: 0.58 (1:1, CH/EA).

#### 3-(((((9H-Fluoren-9-yl)methoxy)carbonyl)amino)methyl)-5-(ditert-butylfluorosilyl)benzoic Acid (Fmoc-(SiFA)SeFe)

For the oxidation from alcohol to acid 1.6 g of xi (3.1 mmol, 1.0 equiv) and 96.9 mg TEMPO (0.6 mmol, 0.2 equiv) were solved in 20 mL ACN. Then 10 mL of Phosphate buffer (pH 6.7) was added, heated to 40 °C and 552.0 mg NaClO_2_ (6.1 mmol, 2.0 equiv) solved in 5 mL H_2_O and 46.2 mg NaOCl (0.6 mmol, 6% in H_2_O ≙ 631.0 *μ*L, 0.2 equiv) were added simultaneously over 1 h. After stirring at 40 °C for 2 h the solution was extracted with Et_2_O (3 × 50 mL), dried over Na_2_SO_4_ and the solvent was removed under reduced pressure. The crude product was purified via flash chromatography (CH/EA + 0.1% AcOH) to obtain the product as 1.3 g (2.4 mmol, 76.6%) of a colorless solid.

^1^H-NMR: (500 MHz, Chloroform-*d*) *δ* 8.25 (s, 1H), 8.09 (s, 1H), 7.79 (s, 1H), 7.76 (d, *J* = 7.7 Hz, 2H), 7.60 (d, *J* = 7.4 Hz, 2H), 7.40 (t, *J* = 7.5 Hz, 2H), 7.31 (t, *J* = 7.3 Hz, 2H), 4.52−4.43 (m, 4H), 4.25 (t, *J* = 6.9 Hz, 1H), 1.06 (s, 18H).

^13^C-NMR (101 MHz, Chloroform-*d*): 170.80 (*C*OOH), 156.62 (N*C* O), 144.00 (*C*), 141.45 (*C*), 138.21 (*C*), 135.44 (*C*), 135.30 (*C*), 134.72 (*C*H), 130.32 (*C*H), 129.30 (*C*H), 127.85 (*C*H), 127.22 (*C*H), 125.16 (*C*H), 120.12 (*C*H), 69.56 (O*C*H_2_), 47.36 (*C*H), 31.34 (N*C*H_2_), 27.42 (*C*CH_3_), 20.42 (*C*H_3_).

^19^F-NMR (376 MHz, Chloroform-*d*): *δ* −188.18.

^29^Si-INEPT NMR (60 MHz, Chloroform-*d*): *δ* 13.54.

R_f_: 0.40 (1:1, CH/EA + 0.1% AcOH).

### General Section (GS) for Solid-Phase Peptide Synthesis Following the Fmoc Strategy (Fmoc-SPPS)

#### GS1: Loading of the 2-CTC Resin

The 2-CTC resin (2-chloro-tritylchlorid resin) (loading density: 1.6 mmol/g) is loaded with a Fmoc-protected amino acid (AA) using Fmoc-AA-OH (1.5 equiv) and DIPEA (*N,N*-Diisopropy-lethylamine) (1.5 equiv) in DMF in a 20 mL peptide syringe. After 15 min of preactivation at RT, another 3.0 equiv of DIPEA is added and the mixture is shaken at RT for 2 h. MeOH (1 mL/g resin) is added to the resin and shaken for 15 min (“capping”). Finally, the resin is washed five times each with DMF (5 mL), MeOH (5 mL) and DCM (5 mL). The loading density is calculated as follows:

(1) $$\text{loading}\;\text{density}\;l\left[\frac{\text{mmol}}{\text{g}}\right]=\frac{\left(m_{2}-m_{1}\right)\times 1000}{\left(M-M_{\text{HCl}}\right)\times m_{2}}$$

*m*_1_ = Mass of the dry uncoated resin [g]

*m*_2_ = Mass of the dry coated resin [g]

*M* = Molecular weight of the amino acid to be coupled [g/mol]

*M*_HCl_ = Molecular weight of HCl (36.46 g/mol)

#### GS2: Fmoc Deprotection

*N*-terminal Fmoc-protected amino acids or peptides bound to the resin are deprotected by adding 20% piperidine in DMF (5 mL) at RT. The deprotection reagent is added twice (1 × 5 min, 1 × 15 min). The resin is then washed with DMF (6x with 5 mL each).

#### GS3: Acetyl Deprotection

Acetyl deprotection is performed by dissolving 50 *μ*mol of the peptide in MeOH and adding NaOMe until the pH is 11−12. After 15 min, the reaction is stopped by adding TFA (pH = 2).

#### GS4: Dde Deprotection

*N*-terminal Dde-protected amino acids or peptides bound to the resin are deprotected by adding NH_3_OHCl (100 equiv) and Imidazole (75 equiv) in NMP/DCM (5/2, 5 mL) and shaken for 2 h. The resin is then washed with NMP (6 x with 5 mL each) and DMF (6 x with 5 mL each).

#### GS5: Peptide Coupling to the Resin

The loaded resin is swollen in NMP (*N*-methyl-2-pyrrolidone) for 30 min, washed six times with DMF (5 mL), and *N*-terminally Fmoc deprotected. Prior to coupling at the *C*-terminus of side-chain-protected Fmoc-AA−OH (1.5 equiv), preactivation is performed with TBTU (*N,N,N*′,*N*′-tetramethyluronium-tetrafluorborate) (1.5 equiv), HOAt (1-Hydroxy-7-azabenzotriazole) (1.5 equiv), and DIPEA (4.0 equiv) in 5 mL DMF at RT. After 10 min, the activated solution is added to the resin-bound peptide containing the free amine (2-CTC-AA-NH_2_) and shaken for 1.5 h at RT. The resin is then washed six times with DMF (5 mL) and, after Fmoc deprotection, washed another six times with DMF (5 mL). Thereafter, the next amino acid can be conjugated, or the resin is washed six times with DCM and dried in a desiccator.

#### GS6: Coupling of Fmoc-L-Asp(^t^Bu)-OH

For the coupling of Fmoc-L-Asp(^*t*^Bu)-OH to the resin-bound, *N*-terminally deprotected peptide (1.0 equiv), the peptide is first preactivated with a solution of TBTU (3.0 equiv), HOAt (3.0 equiv) and DIPEA (9.0 equiv) in DMF (3 mL) for 2 min. Then, Fmoc-L-Asp(^*t*^Bu)-OH (3.0 equiv) is added to the preactivated solution in 2 mL and shaken for 2 h at RT. The resin is then washed six times with DMF (5 mL each) and four times with DCM (5 mL each).

#### GS7: Coupling of Fmoc-Asn(Ac_3_AcNH-β-Glc)-OH

For the coupling of Fmoc-Asn(Ac_3_AcNH-*β*-Glc)-OH to the resin-bound, *N*-terminally deprotected peptide (1.0 equiv), the peptide is first preactivated with a solution of HATU (1.9 equiv), HOAt (1.9 equiv) and DIPEA (2.0 equiv) in DMF (3 mL) for 2 min. Then, Fmoc-Asn(Ac_3_AcNH-*β*-Glc)-OH (2.0 equiv) is added to the preactivated solution in 2 mL and shaken for 2 h at RT. The resin is then washed six times with DMF (5 mL each) and four times with DCM (5 mL each).

#### GS8: Coupling of Bis-Boc-amino-oxyacetic Acid

For the coupling of bis-Boc-amino-oxyacetic acid to the resin-bound, *N*-terminally deprotected peptide (1.0 equiv), the peptide is first preactivated with a solution of TBTU (1.9 equiv), HOAt (1.9 equiv) and DIPEA (2.0 equiv) in DMF (3 mL) for 2 min. Then, bis-Boc-amino-oxyacetic acid (2.0 equiv) is added to the preactivated solution in 2 mL and shaken for 2 h at RT. The resin is then washed six times with DMF (5 mL each) and four times with DCM (5 mL each).

#### GS9: Coupling of Fmoc-D-Dap-O^t^Bu·HCl

For the coupling of Fmoc-D-Dap-OtBu HCl to the resin-bound, *N*-terminally deprotected peptide (1.0 equiv), the peptide is first preactivated with a solution of TBTU (1.5 equiv), HOAt (1.5 equiv) and 2,4,6-Trimethylpyridine (5.0 equiv) in DMF (3 mL) for 2 min. Then, Fmoc-D-Dap-OtBu·HCl (1.5 equiv) is added to the preactivated solution in 2 mL and shaken for 2 h at RT. The resin is then washed six times with DMF (5 mL each) and four times with DCM (5 mL each).

#### GS10: Coupling of Fmoc-D-Dap(Dde)-OH

For the coupling of Fmoc-D-Dap(Dde)-OH to the resin-bound, *N*-terminally deprotected peptide (1.0 equiv), the peptide is first preactivated with a solution of TBTU (1.5 equiv), HOAt (1.5 equiv) and 2,4,6-Trimethylpyridine (5.0 equiv) in DMF (3 mL) for 2 min. Then, Fmoc-D-Dap(Dde)-OH (1.5 equiv) is added to the preactivated solution in 2 mL and shaken for 2 h at RT. The resin is then washed six times with DMF (5 mL each) and four times with DCM (5 mL each).

#### GS11: Coupling of DOTA(^t^Bu)_2_

For the coupling of *trans*-(di-*tert*-butyl)-1,4,7,10-tetraazacyclododecane-1,4,7,10-tetraacetic acid (DOTA(^*t*^Bu)_2_) to the resin bound *N*-terminally deprotected peptide (1.0 equiv), a solution of DOTA(^*t*^Bu)_2_ (3.0 equiv), HOAt (3.0 equiv), TBTU (3.0 equiv), and sym-collidine (11.0 equiv) is preactivated in DMF (5 mL) for 10 min. This solution is added to the resin-bound peptide and shaken overnight. Finally, the resin is washed six times with DMF (5 mL each) and four times with DCM (5 mL each).

#### GS12: Coupling of DOTA(^t^Bu)_3_

DOTA-tris(^*t*^Bu)ester (DOTA-(^*t*^Bu)_3_) (1.5 equiv), HATU (1.5 equiv), and HOAt (1.5 equiv), are dissolved in DMF (5 mL). DIPEA (4.5 equiv) is then added to the solution and left to preactivate for 15 min. The activated solution is added to the resin and reacted for 3 h at RT. Finally, the resin is washed six times with DMF (5 mL each).

#### GS13: Coupling of Fmoc-O_2_Oc-OH

For the coupling of 8-(9-Fluorenylmethyloxycarbonyl-amino)-3,6-dioxaoctanoic acid (Fmoc-O_2_Oc-OH) to the *N*-terminally deprotected peptide bound to the resin (1.0 equiv), a solution of Fmoc-O_2_Oc-OH (2.0 equiv), HATU (1.9 equiv), HOAt (1.9 equiv) and DIPEA (2.0 equiv) is preactivated in DMF (5 mL) for 15 min. This solution is added to the resin-bound peptide and shaken for 1.5 h at RT. Finally, the resin is washed six times with DMF (5 mL each).

#### GS14: Cleavage from the Resin with Removal of Acid Labile Protection Groups

The peptide bound to the resin is mixed with 5 mL of a mixture of TFA/TIPS/H_2_O (95/2.5/2.5) and agitated for 45 min at RT twice. The solution with the deprotected peptide is collected in a 50 mL round-bottom flask and the remaining resin is washed once with TFA and stirred overnight. The following day, the TFA is evaporated under nitrogen stream.

#### GS15: Cleavage from the Resin with Retention of Acid Labile Protective Groups

The peptide bound to the resin is mixed with 5 mL of a mixture of 2,2,2-Trifluorethanol (TFE)/DCM/AcOH (3/6/1) and agitated for 20 min at RT. The solution with the protected peptide is collected and evaporated under a nitrogen stream. After dissolving the residue in MeCN/H_2_O (1/1, *v/v*) the obtained protected product is analyzed by RP-HPLC and ESI-MS.

#### GS16: Lyophilization of Purified Peptide

After purification via RP-HPLC the solvent is removed under reduced pressure, the residue is solved in *tert*-Butanol/H_2_O (*v/v* = 1:1), frozen at −80 °C and lyophilized.

### Synthesis of Model (SiFA)SeFe-Bioconjugates

Model (SiFA)SeFe-bioconjugates were synthesized by coupling the first amino acid to the 2-CTC-resin (**GS1**), Fmoc deprotection (**GS2**), coupling of the second amino acid (**GS5**), Fmoc deprotection (**GS2**), coupling of Fmoc-(SiFA)SeFe (**GS5**), Fmoc deprotection (**GS2**) for 1X_n_. For 2X_n_ and 3X_n_ another amino acid coupling (**GS5**) and Fmoc deprotection was performed. The peptides were cleaved from the resin (**GS14**), purified via RP-HPLC and lyophilized (**GS16**).

(3-(aminomethyl)-5-(di-*tert*-butylfluorosilyl)benzoyl)glycyl-L-lysine (**H_2_N-(SiFA)SeFe-Gly-Lys-OH, 1X_1_**):

15.8% yield, **RP-HPLC** (10−60% MeCN/H_2_O with 0.1% TFA, *v/v*, 15 min, *λ* = 220 nm): *t*_R_ = 10.2 min.

**MS** (ESI positive): *m/z* calculated for H_2_N-(SiFA)SeFe-Gly-Lys-OH: 496.29, found: 497.3 [M + H^+^]^+^.

(3-(aminomethyl)-5-(di-*tert*-butylfluorosilyl)benzoyl)glycyl-L-as-partic acid (**H_2_N-(SiFA)SeFe-Gly-Asp-OH, 1X_2_**):

8.3% yield, **RP-HPLC** (10−60% MeCN/H_2_O with 0.1% TFA, *v/v*, 15 min, *λ* = 220 nm): *t*_R_ = 11.6 min.

**MS** (ESI positive): *m/z* calculated for H_2_N-(SiFA)SeFe-Gly-Asp-OH: 483.22, found: 484.6 [M + H^+^]^+^.

(3-(aminomethyl)-5-(di-*tert*-butylfluorosilyl)benzoyl)glycyl-L-tyrosine (**H_2_N-(SiFA)SeFe-Gly-Tyr-OH, 1X_3_**):

16.4% yield, **RP-HPLC** (10−60% MeCN/H_2_O with 0.1% TFA, *v/v*, 15 min, *λ* = 220 nm): *t*_R_ = 12.8 min.

**MS** (ESI positive): *m/z* calculated for H_2_N-(SiFA)SeFe-Gly-Tyr-OH: 531.26, found: 532.5 [M + H^+^]^+^.

(3-(di-*tert*-butylfluorosilyl)-5-(((S)-2,6-diaminohexanamido)-methyl)benzoyl)glycyl-L-lysine (**H_2_N-Lys-(SiFA)SeFe-Gly-Lys-OH, 2X_1_**):

13.3% yield, **RP-HPLC** (10−60% MeCN/H_2_O with 0.1% TFA, *v/v*, 15 min, *λ* = 220 nm): *t*_R_ = 9.5 min.

**MS** (ESI positive): *m/z* calculated for H_2_N-Lys-(SiFA)SeFe-Gly-Lys-OH: 624.38, found: 625.4 [M + H^+^]^+^.

(3-(di-*tert*-butylfluorosilyl)-5-(((S)-2,6-diaminohexanamido)-methyl)benzoyl)glycyl-L-aspartic acid (**H_2_N-Lys-(SiFA)SeFe-Gly-Asp-OH, 2X_2_**):

7.4% yield, **RP-HPLC** (10−60% MeCN/H_2_O with 0.1% TFA, *v/v*, 15 min, *λ* = 220 nm): *t*_R_ = 10.6 min.

**MS** (ESI positive): *m/z* calculated for H_2_N-Lys-(SiFA)SeFe-Gly-Asp-OH: 611.32, found: 612.2 [M + H^+^]^+^.

(3-(di-*tert*-butylfluorosilyl)-5-(((S)-2,6-diaminohexanamido)-methyl)benzoyl)glycyl-L-tyrosine (**H_2_N-Lys-(SiFA)SeFe-Gly-Tyr-OH, 2X_3_**):

12.6% yield, **RP-HPLC** (10−60% MeCN/H_2_O with 0.1% TFA, *v/v*, 15 min, *λ* = 220 nm): *t*_R_ = 11.4 min.

**MS** (ESI positive): *m/z* calculated for H_2_N-Lys-(SiFA)SeFe-Gly-Tyr-OH: 659.35, found: 660.3 [M + H^+^]^+^.

(3-(((S)-2-amino-4-carboxybutanamido)methyl)-5-(di-*tert*-butylfluorosilyl)benzoyl)glycyl-L-lysine (**H_2_N-Glu-(SiFA)SeFe-Gly-Lys-OH, 3X_1_**):

10.1% yield, **RP-HPLC** (10−60% MeCN/H_2_O with 0.1% TFA, *v/v*, 15 min, *λ* = 220 nm): *t*_R_ = 10.4 min.

**MS** (ESI positive): *m/z* calculated for H_2_N-Glu-(SiFA)SeFe-Gly-Lys-OH: 625.33, found: 626.3 [M + H^+^]^+^.

(3-(((S)-2-amino-4-carboxybutanamido)methyl)-5-(di-*tert*-butylfluorosilyl)benzoyl)glycyl-L-aspartic acid (**H_2_N-Glu-(SiFA)-SeFe-Gly-Asp-OH, 3X_2_**):

4.9% yield, **RP-HPLC** (10−60% MeCN/H_2_O with 0.1% TFA, *v/v*, 15 min, *λ* = 220 nm): *t*_R_ = 11.8 min.

**MS** (ESI positive): *m/z* calculated for **H_2_N-Glu**-(SiFA)SeFe-Gly-Asp-OH: 612.26, found: 613.2 [M + H^+^]^+^.

(S)-4-amino-5-((3-((2-(((S)-1-carboxy-2-(4-hydroxyphenyl)-ethyl)amino)-2-oxoethyl)carbamoyl)-5-(di-*tert*-butylfluorosilyl)-benzyl)amino)-5-oxopentanoic acid (**H_2_N-Glu-(SiFA)SeFe-Gly-Tyr-OH, 3X_3_**):

12.8% yield, **RP-HPLC** (10−60% MeCN/H_2_O with 0.1% TFA, *v/v*, 15 min, *λ* = 220 nm): *t*_R_ = 12.6 min.

**MS** (ESI positive): *m/z* calculated for H_2_N-Glu-(SiFA)SeFe-Gly-Tyr-OH: 660.30, found: 661.0 [M + H^+^]^+^.

### Synthesis of SST Binding Motif and SST-Ligands

#### H-TATE(PG)-2-CT

The synthesis of H-TATE(PG)-2-CT is carried out on the resin using the general working procedures (**GS**). 2-CTC resin is loaded with Fmoc-L-Thr(^*t*^Bu)-OH (**GS1**). This is followed by the coupling of Fmoc-L-Cys(Acm)-OH, Fmoc-L-Thr(^*t*^Bu)-OH, Fmoc-L-Lys(Boc)-OH, Fmoc-D-Trp(Boc)-OH, Fmoc-L-Tyr(^*t*^Bu)-OH, Fmoc-L-Cys(Acm)-OH and Fmoc-D-Phe-OH (**GS5**). Before the coupling of the next amino acid in each case, the *N*-terminus is Fmoc-deprotected (**GS2**). The final amino acid is only deprotected after the formation of the disulfide bridge.

#### Formation of the Disulfide Bridge

Fmoc-D-Phe-L-Cys(Acm)-L-Tyr(^*t*^Bu)-D-Trp(Boc)-L-Lys(Boc)-L-Thr(^*t*^Bu)-L-Cys(Acm)-L-Thr-(^*t*^Bu)-2-CT (1.0 equiv) is mixed with Tl(TFA)_3_ (4.0 equiv) and glycerol (4.0 equiv) in DMF (8 mL/g resin). After 1 h at room temperature, the solution is discarded and a fresh solution of the reaction solution is added to the resin for another 1 h at room temperature. The resin is then washed with DMF (6 × 5 mL/g resin). Test cleavage from the resin with retention of acid labile protective groups is used to verify the completeness of the cyclization (**GS15**). Characterization is investigated by analytical RP-HPLC and ESI-MS. After final Fmoc deprotection, the product, H-D-Phe-cyclo[L-Cys-L-Tyr(^*t*^Bu)-D-Trp(Boc)-L-Lys(Boc)-L-Thr(^*t*^Bu)-L-Cys]-L-Thr(^*t*^Bu)-2-CT is present bound to the resin.

**RP-HPLC** (10−90% MeCN/H_2_O with 0.1% TFA, *v/v*, 15 min, *λ* = 220 nm): *t*_R_ = 13.4 min.

**MS** (ESI positive): *m/z* calculated for H-TATE(PG)-OH: 1416.7; found: 1418.3 [M + H^+^]^+^.

#### SiFAlin-TATE

The synthesis of SiFAl*in*-TATE is carried out on resin using the general working procedures (**GS**). The resin-bound synthesis of H-TATE(PG)-2-CT is followed by the coupling of Fmoc-O_2_Oc-OH (**GS13**), Fmoc-L-Asp(O^*t*^Bu)-OH (**GS6**), Fmoc-L-Asp-(O^*t*^Bu)-OH (**GS6**), Fmoc-Asn(Ac_3_AcNH-*β*-Glc) OH (**GS7**) and *bis*-Boc-amino-oxyacetic acid (**GS8**). Before the coupling of the next amino acid in each case, the *N*-terminus is Fmoc-deprotected (**GS2**). After resin cleavage, removal of all protecting groups (**GS14**) and acetyl deprotection (**GS3**), purification is carried out by RP-HPLC (30−35% MeCN/H_2_O with 0.1% TFA, *v/v*, 20 min, *λ* = 220 nm).

#### Oxime Ligation

1.0 equiv TATE-O_2_OC-L-Asp-L-Asp-Asn-amino-oxy-acid and 4.0 equiv SiFA*lin* aldehyde in 400 *μ*L phosphate buffer/MeCN (1/1, *v/v*) are vesified with 4 M NaOH solution until a pH of pH = 4 is established. After 20 min the solution is diluted 1/1 with H_2_O (+0.1% TFA) and purified by RP-HPLC (20−45−60% MeCN/H_2_O with 0.1% TFA, *v/v*, 10−30 min, *λ* = 220 nm) and lyophilized (**GS16**). 3.41 mg (1.52 *μ*mol, 5%) were obtained in the form of a white solid.

**RP-HPLC** (10−60% MeCN/H_2_O with 0.1% TFA, *v/v*, 15 min, *λ* = 220 nm): *t*_R_ = 13.5 min.

**MS** (ESI positive): *m/z* calculated for SiFA*lin*-TATE: 2160.9, found: 1082.5 [M + 2H^+^]^2+^.

#### DOTA-TATE

The synthesis of DOTA-TATE is carried out on resin using the general working procedures (**GS**). The resin-bound synthesis of H-TATE(PG)-2-CT is followed by the coupling of DOTA(^*t*^Bu)_3_ (**GS12**). After resin cleavage, removal of all protecting groups (**GS14**), purification by RP-HPLC (15−40% MeCN/H_2_O with 0.1% TFA, *v/v*, 30 min, *λ* = 220 nm) and lyophilization (**GS16**), 1.11 mg (7.73 *μ*mol, 19%) is obtained in the form of a white solid.

**RP-HPLC** (10−60% MeCN/H_2_O with 0.1% TFA, *v/v*, 15 min, *λ* = 220 nm): *t*_R_ = 8.3 min.

**MS** (ESI positive): *m/z* calculated for DOTA-TATE: 1434.6, found: 718.2 [M + 2H^+^]^2+^, 479.5 [M + 3H^+^]^3+^.

#### (SiFA)SeFe-rhTATE1

The synthesis of (SiFA)SeFe-rhTATE1 is carried out on resin using the general working procedures (**GS**). The resin-bound synthesis of H-TATE(PG)-2-CT is followed by the coupling of DOTA(^*t*^Bu)_2_ (**GS11**), Fmoc-D-Dap-O^*t*^Bu·HCl (**GS9**) and Fmoc-(SiFA)SeFe-OH (**GS5**). Before the coupling of the next amino acid in each case, the *N*-terminus is Fmoc-deprotected (**GS2**). After resin cleavage, removal of all protecting groups (**GS14**), purification by RP-HPLC (30−50% MeCN/H_2_O with 0.1% TFA, *v/v*, 30 min, *λ* = 220 nm) and lyophilization (**GS16**), 1.28 mg (0.70 *μ*mol, 2%) is obtained in the form of a white solid.

**RP-HPLC** (10−60% MeCN/H_2_O with 0.1% TFA, *v/v*, 15 min, *λ* = 220 nm): *t*_R_ = 11.0 min.

**MS** (ESI positive): *m/z* calculated for (SiFA)SeFe-rhTATE1: 1813.8, found: 606.1 [M + 3H^+^]^3+^, 619.7 [M + H^+^ + 2Na^+^]^3+^, 1211.1 [2 M + 3H^+^]^3+^.

#### (SiFA)SeFe-rhTATE2

The synthesis of (SiFA)SeFe-rhTATE2 is carried out on resin using the general working procedures (**GS**). The resin-bound synthesis of H-TATE(PG)-2-CT is followed by the coupling of DOTA(^*t*^Bu)_2_ (**GS11**), Fmoc-D-Dap-O^*t*^Bu·HCl (**GS9**), Fmoc-(SiFA)SeFe−OH (**GS5**) and Fmoc-D-Asp-O^*t*^Bu (**GS5**). Before the coupling of the next amino acid in each case, the *N*-terminus is Fmoc-deprotected (**GS2**). After resin cleavage, removal of all protecting groups (**GS14**), purification by RP-HPLC (30−60% MeCN/H_2_O with 0.1% TFA, *v/v*, 30 min, *λ* = 220 nm) and lyophilization (**GS16**), 2.46 mg (1.28 *μ*mol, 3%) is obtained in the form of a white solid.

**RP-HPLC** (10−90% MeCN/H_2_O with 0.1% TFA, *v/v*, 15 min, *λ* = 220 nm): *t*_R_ = 8.2 min.

**MS** (ESI positive): *m/z* calculated for (SiFA)SeFe-rhTATE2: 1928.8, found: 644.0 [M + 3H^+^]^3+^, 965.7 [M + 2H^+^]^2+^, 1287.9 [2 M + 3H^+^]^3+^.

#### (SiFA)SeFe-rhTATE3

The synthesis of (SiFA)SeFe-rhTATE3 is carried out on resin using the general working procedures (**GS**). The resin-bound synthesis of H-TATE(PG)-2-CT is followed by the coupling of Fmoc-O_2_Oc-OH (**GS13**) and subsequent *N*-terminal Fmoc deprotection (**GS2**). Coupling of Fmoc-D-Dap(Dde)-OH (**GS10**) is followed by Dde deprotection (**GS4**) of the *N*-terminus. After that the couplings of DOTA(^*t*^Bu)_3_ (**GS12**), Fmoc-O_2_Oc-OH (**GS13**) and Fmoc-(SiFA)SeFe-OH (**GS5**) take place. Before the coupling of the next amino acid in each case, the *N*-terminus is Fmoc-deprotected (**GS2**). After resin cleavage, removal of all protecting groups (**GS14**), purification by RP-HPLC (30−45% MeCN/H_2_O with 0.1% TFA, *v/v*, 30 min, *λ* = 220 nm) and lyophilization (**GS16**), 2.82 mg (1.34 *μ*mol, 3%) is obtained in the form of a white solid.

**RP-HPLC** (10−60% MeCN/H_2_O with 0.1% TFA, *v/v*, 15 min, *λ* = 220 nm) for **(SiFA)SeFe-rhTATE3**: *t*_R_ = 11.4 min.

**MS** (ESI positive): *m/z* calculated for (SiFA)SeFe-rhTATE3: 2104.0, found: 1053.7 [M + 2H^+^]^2+^, 702.9 [M + 3H^+^]^3+^, 527.7 [M + 4H^+^]^4+^.

#### (SiFA)BA-rhTATE1

The synthesis of (SiFA)BA-rhTATE is carried out on resin using the general working procedures (**GS**). The resin-bound synthesis of H-TATE(PG)-2-CT is followed by the coupling of DOTA(^*t*^Bu)_2_ (**GS11**), Fmoc-D-Dap-O^*t*^Bu·HCl (**GS9**) and Fmoc-(SiFA)SeFe-OH (**GS5**). Before the coupling of the next amino acid in each case, the *N*-terminus is Fmoc-deprotected (**GS2**). After resin cleavage, removal of all protecting groups (**GS14**), purification by RP-HPLC (40−75% MeCN/H_2_O with 0.1% TFA, *v/v*, 20 min, *λ* = 220 nm) and lyophilization (**GS16**), 5.52 mg (3.09 *μ*mol, 8%) is obtained in the form of a white solid.

**RP-HPLC** (10−90% MeCN/H_2_O with 0.1% TFA, *v/v*, 15 min, *λ* = 220 nm): *t*_R_ = 9.7 min.

**MS** (ESI positive): *m/z* calculated for (SiFA)BA-rhTATE: 1784.8, found: 1786.8 [M + H^+^]^+^, 892.9 [M + 2H^+^]^2+^.

#### (SiFA)BA-rhTATE3

The synthesis of **(SiFA)BA-rhTATE3** is carried out on resin using the general working procedures (**GS**). The resin bound synthesis of H-TATE(PG)-2-CT is followed by the coupling of Fmoc-O_2_Oc-OH (**GS13**) and subsequent N-terminal Fmoc deprotection (**GS2**). Coupling of Fmoc-D-Dap(Dde)-OH (**GS10**) is followed by Dde deprotection (**GS4**) of the N-terminus. After that the couplings of DOTA(tBu)_3_ (**GS12**), Fmoc-O_2_Oc-OH (**GS13**) and Fmoc-(SiFA)BA-OH (**GS5**) take place. Before the coupling of the next amino acid in each case, the N-terminus is Fmoc-deprotected (**GS2**). After resin cleavage, removal of all protecting groups (**GS14**), purification by RP-HPLC (45−60% MeCN/H_2_O with 0.1% TFA, v/v, 30 min, *λ* = 220 nm) and lyophilization (**GS16**), 1.74 mg (0.84 *μ*mol, 2%) is obtained in the form of a white solid.

**RP-HPLC** (10−60% MeCN/H2O with 0.1% TFA, v/v, 15 min, *λ* = 220 nm) for **(SiFA)BA-rhTATE3**: t_R_ = 14.1 min.

**MS** (ESI positive): *m/z* calculated for (SiFA)BA-rhTATE3: 2074.9, found: 693.3 [M + 3H^+^]^3+^, 1039.0 [M + 2H^+^]^2+^.

#### [^nat^I]I-TOC

N-iodosuccinimide (NIS, 0.5 equiv) is added to the respective peptide solution [9 m_M_ in acetonitrile/water (1:1)]. After 5 min at room temperature, the solvent is removed and the ^nat^I-peptide is purified via RP-HPLC (15−50% MeCN/H_2_O with 0.1% TFA, *v/v*, 30 min, *λ* = 220 nm). 0.14 mg (0.12 *μ*mol, 27%) is obtained in the form of a white solid.

**RP-HPLC** (10−60% MeCN/H_2_O with 0.1% TFA, *v/v*, 15 min, *λ* = 220 nm): *t*_R_ = 9.2 min.

**MS** (ESI positive): *m/z* calculated for [^nat^I]I-TOC: 1160.3, found: 1162.0 [M + H^+^]^+^, 581.6 [M + 2H^+^]^2+^.

#### Complexation of DOTA Moieties with ^nat^Ga-Gallium

3.0 equiv of an aqueous Ga(NO_3_)_3_ solution (100 mM) and 1.0 equiv of the corresponding DOTA-conjugated peptide precursor (2 mM in DMSO) were added to a Protein LoBind Eppendorf tube and diluted with DMSO to a final concentration of 1 mM. After reacting at 70 °C for 1 h, quality control was carried out *vi*a analytical RP-HPLC and ESI-MS.

**DOTA-TATE: RP-HPLC** (10−60% MeCN/H_2_O with 0.1% TFA, *v/v*, 15 min, *λ* = 220 nm): *t*_R_ = 8.5 min.

**MS** (ESI positive): *m/z* calculated for [^nat^Ga]Ga-DOTA-TATE: 1500.5, found: 752.4 [M + 2H^+^]^2+^.

**(SiFA)SeFe-rhTATE1: RP-HPLC** (10−60% MeCN/H_2_O with 0.1% TFA, *v/v*, 15 min, *λ* = 220 nm): *t*_R_ = 11.1 min.

**MS** (ESI positive): *m/z* calculated for [^nat^Ga]Ga-(SiFA)SeFe-rhTATE1: 1880.7, found: 628.4 [M + 3H^+^]^3+^, 942.1 [M + 2H^+^]^2+^, 1255.7 [2M + 3H^+^]^3+^.

**(SiFA)SeFe-rhTATE2: RP-HPLC** (10−60% MeCN/H_2_O with 0.1% TFA, *v/v*, 15 min, *λ* = 220 nm): *t*_R_ = 11.7 min.

**MS** (ESI positive): *m/z* calculated for [^nat^Ga]Ga-(SiFA)SeFe-rhTATE2:1995.7, found: 666.2 [M + 3H^+^]^3+^, 999.1 [M + 2H^+^]^2+^, 1332.6 [2 M + 3H^+^]^3+^.

**(SiFA)BA-rhTATE1: RP-HPLC** (10−90% MeCN/H_2_O with 0.1% TFA, *v/v*, 15 min, *λ* = 220 nm): *t*_R_ = 10.1 min.

**MS** (ESI positive): *m/z* calculated for [^nat^Ga]Ga-(SiFA)BA-rhTATE1: 1852.2, found: 927.0 [M + 2H^+^]^2+^, 1235.3 [2M + 3H^+^]^3+^, 1853.2 [M + H^+^]^+^.

#### Complexation of DOTA Moieties with [^nat^Lu]Lutetium

For the incorporation of [^nat^Lu]lutetium, LuCl_3_ (20 mM in H_2_O, 3.0 equiv) was added to a 2 mM solution of the compound in DMSO and diluted to 1 mM by addition of DMSO. The obtained solution was incubated at 70 °C for 15 min.

**DOTA-TATE: RP-HPLC** (10−60% MeCN/H_2_O with 0.1% TFA, *v/v*, 15 min, *λ* = 220 nm) for [^nat^Lu]Lu-DOTA-TATE: *t*_R_ = 8.5 min.

**MS** (ESI positive): *m/z* calculated for [^nat^Lu]Lu-DOTA-TATE: 1606.5, found: 804.6 [M + 2H^+^]^2+^.

**(SiFA)SeFe-rhTATE3: RP-HPLC** (10−60% MeCN/H_2_O with 0.1% TFA, *v/v*, 15 min, *λ* = 220 nm) for [^nat^Lu]Lu-(SiFA)SeFe-rhTATE3: *t*_R_ = 11.9 min.

**MS** (ESI positive): *m/z* calculated for [^nat^Lu]Lu-(SiFA)SeFe-rhTATE3: 2275.9, found: 1139.4 [M + 2H^+^]^2+^, 759.9 [M + 3H^+^]^3+^. **(SiFA)BA-rhTATE3: RP-HPLC** (10−60% MeCN/H_2_O with % TFA, *v/v*, 15 min, *λ* = 220 nm) for [^nat^Lu]Lu-(SiFA)BA-rhTATE3: *t*_R_ = 15.1 min.

**MS** (ESI positive): *m/z* calculated for [^nat^Lu]Lu-(SiFA)BA-rhTATE3: 2246.8, found: 1124.7 [M + 2H^+^]^2+^.

**^177^Lu-Labeling**. For lutetium-177 labeling, the aq. [^177^Lu]LuCl_3_

(10 MBq) is added to 1 *μ*L (1 nmol) of the ligand (1 mM stock in DMSO), 10 *μ*L of a NaOAc buffer (pH = 4.5), 22 *μ*L 0.04 M HCl and the mixture reacted at 70 °C for 5 min.

**^18^F-Labeling Protocols**. *^18^F-labeling of Model (SiFA)SeFe-Bioconjugates*. Labeling of the model (SiFA)SeFe-bioconjugates with fluoride-18 was carried out via isotopic exchange reaction (IE). Therefore, the required amount of fluoride-18 (0.2−2.0 GBq in [^18^O]H_2_O) was fixed on a Sep Pak Light (46 mg) Acell Plus QMA Carbonate cartridge (preconditioned with 10 mL H_2_O) and dried with 8 mL of DMSO (anhydrous). The loaded cartridge was then eluted with 500 *μ*L of NH_4_HCOO in DMSO (1 M) into a Protein LoBind Eppendorf tube. To 9 *μ*L of the respective model bioconjugate in DMSO (1 mm, 9 nmol) 150 *μ*L of this eluate was added and kept for 5 min at RT. After the reaction an aliquot was analyzed via Radio-TLC (silica gel 60, mobile phase: MeCN/PBS (6/4, *v/v*) + 10 vol % 2 M NaOAc + 1 vol % TFA) for determining the RCC. The reaction mixture was quenched with H_2_O (10 mL) and the peptide was fixed on an Oasis HLB (30 mg) Light Cartridge (preconditioned with 10 mL EtOH and 10 mL H_2_O). The cartridge was washed with H_2_O (10 mL) and the peptide was eluted with 300 *μ*L of EtOH. Quality control of the radiolabeled bioconjugates was carried out via radio-RP-HPLC (10−60% B in 15 min).

#### ^18^F-Labeling of SSTR2-addressing Ligands

Labeling of SiFA moieties with fluoride-18 was carried out via isotopic exchange reaction (IE). Therefore, the required amount of fluoride-18 (0.2−2.0 GBq in [^18^O]H_2_O) was fixed on a Sep Pak Light (46 mg) Acell Plus QMA Carbonate cartridge (preconditioned with 10 mL H_2_O) and dried with 8 mL of DMSO (anhydrous). The loaded cartridge was then eluted with 150 *μ*L of NH_4_HCOO in DMSO (1 M) into a Protein LoBind Eppendorf tube, containing 30.0 *μ*L of the respective SiFA-conjugated peptide precursor in DMSO (1 mM, 30.0 nmol). After 10 min at RT, the reaction mixture was quenched with H_2_O (10 mL) and the peptide was fixed on an Oasis HLB (30 mg) Light Cartridge (preconditioned with 10 mL EtOH and 10 mL H_2_O). The cartridge was washed twice with H_2_O (10 mL) and the peptide was eluted with 300 *μ*L of EtOH/PBS (7/3, *v/v*). Quality control of the radiolabeled peptides was carried out via radio-RP-HPLC (10−60% B in 15 min) and radio-TLC (silica gel 60, mobile phase: MeCN/PBS (6/4, *v/v*) + 10 vol % 2 M NaOAc + 1 vol % TFA).

### Stability Studies of Fluorine-18 Labeled Model (SiFA)SeFe-Bioconjugates

#### Reverse Isotopic Exchange

To 10 *μ*L of the respective ^18^F-labeled (SiFA)SeFe-bioconjugate, a solution of 10 *μ*L 10 mM NaF (pH = 6.5) and 80 *μ*L H_2_O was added. The solution was kept at RT and for each time point (0, 30, 60, 90, 120 min) 10 *μ*L was analyzed via Radio-TLC. The half-life was calculated from the ratio between free fluorine-18 and labeled peptide.

#### Physiological Conditions

To a solution of 90 *μ*L aqueous K_2_CO_3_ buffer (pH 7.4) were added 10 *μ*L of the respective ^18^F-labeled (SiFA)SeFe-bioconjugate. The solution was kept at 37 °C and for each time point (0, 30, 60, 90, 120 min) 10 *μ*L were analyzed via Radio-TLC. The half-life was calculated from the ratio between free fluorine-18 and labeled peptide.

#### Lutetium Labeling Conditions

To a solution of 10 *μ*L aqueous sodium acetate buffer (pH 5.5) and 80 *μ*L 0.04 M HCl were added 10 *μ*L of the respective ^18^F-labeled (SiFA)SeFe-bioconjugate. The solution was kept at 90 °C and for each time point (0, 30, 60, 90, 120 min) 10 *μ*L were analyzed via Radio-TLC. The half-life was calculated from the ratio between free fluorine-18 and labeled peptide.

#### Lipophilicity (logD_pH=7.4_)

For the determination of the octanol-PBS partition coefficient (log*D*_pH=7.4_ values), 500 *μ*L of 1-octanol and 500 *μ*L of PBS were added to a 1.5 mL reaction tube (Eppendorf Tube) (n = 6). Thereafter, 1 MBq of each ^18^F-/^177^Lu-labeled compound was added and vortexed for 3 min at RT. After centrifugation (9.000 rpm, 5 min, RT), 200 *μ*L of each layer were taken separately and the activity was quantified by a *γ*-counter (*PerkinElmer* Inc. Langerwehe, Germany).

### Binding to Human Serum Albumin (HSA)

HSA binding studies were performed according to a previously published procedure, using RP-HPLC and HSA which is solid-phase fixed on a Chiralpak HSA column (50 × 3 mm, 5 *μ*m, H13 h-2433, *Daicel*, Tokio, Japan).^51^ A flow rate of 0.5 mL/min at RT was used. A freshly prepared 50 mM aqueous solution of NH_4_OAc (pH 6.9) was used as mobile phase A, and isopropanol (HPLC grade, *VWR*, Germany) was used as mobile phase B. A gradient of 100% A (0 to 3 min) followed by 80% A (3 to 40 min) was used for the experiments. Before the analysis of all compounds, the column was calibrated with nine reference substances having HSA binding known from the literature in the range of 13 to 99%.^51,52^ All compounds, were prepared in a 1/1 mixture (*v/v*) of isopropanol and a 50 mM aqueous solution of NH_4_OAc (pH 6.9) at a final concentration of 0.5 mg/mL. Nonlinear regression was performed using *OriginPro 2016G* software (North-ampton, United States).

### Iodine-125 Labeling of the Reference TOC for Cell Studies

For *in vitro* studies (*IC*_50_, n = 3), dissolve 50−150 *μ*g of TOC, in a 1.5 mL Eppendorf reaction tube (Protein LowBind), in 20 *μ*L of DMSO and add 280 *μ*L of TRIS buffer (25 mM TRIS-HCl, 0.4 mM NaCl, pH = 7.5). The solution is transferred to a reaction tube (1.5 mL, Protein LowBind) coated with Iodogen (150 *μ*g) and 5.00 *μ*L (10− 20 MBq) [^125^I]NaI solution (74 TBq, 40 mM NaOH, *HARTMANN ANALYTIC GmbH* (Braunschweig, Germany)) is added. After 15 min at RT, the reaction is stopped by separation from the oxidant (Iodogen). The crude product [^125^I]I-TOC is purified by analytical RP-HPLC [(20−40% in 15 min): *t*_R_ = 5.1 min] and 10 vol % sodium ascorbate solution (100 mM in H_2_O, radiolysis quencher) is added to the resulting product solution. The concentration of [^125^I]I-TOC is determined volumetrically by transferring the entire solution to a new vessel (20 mL reaction vessel) and the amount of [^125^I]I-TOC contained is measured using an activimeter. Using the specific activity of the commercially purchased [^125^I]NaI solution, the amount of substance concentration is determined (eqs 2, 3). The product obtained has a radiochemical yield of RCY (radio-RP-HPLC) = 42.9% and a radiochemical purity of RCP (radio-RP-HPLC) = 100%. Characterization of [^125^I]I-TOC was performed by co-injection of [^nat^I]I-TOC using a radio-RP-HPLC. [^125^I]I-TOC is stored at −4 °C and can be used for up to 3 weeks.

Radio-RP-HPLC (20−50% MeCN/H_2_O with 0.1% TFA, *v/v*, 20 min): *t*_R_ = 5.1 min.

(2) [I125]cTOC=A([I125]TOC)74MBqnmol×V([I125]TOC)

(3) V1nM[I125]TOC=1nm×1300μLc[I125]TOC[I125]cTOC=concentrationof[I125]I-TOC[M]A[I125]I-TOC=Specificactivityof[I125]I-TOC[Bq/mol]V[I125]I-TOC=Volumeof[I125]I-TOC[L]V1nM[I125]TOC=Volumeof1nM[I125]I-TOC[L]

### Cell Culture Maintenance

The adherent sstR2-transfected CHO_sst2_ cells (Chinese hamster ovary (CHO) cells, stably transfected with human sstR2 (epitope-tagged at the *N*-terminal end) and kindly provided by Dr. Jenny Koenig, (University of Cambridge, Cambridge, United Kingdom) were cultured in DMEM/F12 GlutaMax medium (plus 10% FBS *v/v*) at 37 °C in a humidified 5% CO_2_ atmosphere. To ensure uniform cell growth, cells were passaged at approximately 80% confluence (2−4 days). The spent medium is removed and the remaining cell lawn washed with PBS (10 mL, 37 °C). By treatment with trypsin/EDTA (5 mL, 5 min, 37 °C) at 37 °C, the cells were detached and suspended adding 5 mL DMEM/F12 GlutaMax medium (plus 10% FBS *v/v*). The suspension was centrifuged (1.300 rpm, 3 min, RT) and the cell pellet resuspended in fresh DMEM/F12 GlutaMax medium (20 mL, plus 10% TCS *v/v*, 37 °C). A portion of the suspension was transferred to new culture flasks and the volume was adjusted to 25 mL with DMEM/F12 GlutaMax medium (plus 10% FBS *v/v*).

AR42J cells (*CLS GmbH*, Eppelheim, Germany and *Sigma-Aldrich*, Gillingham, UK) were cultivated in RPMI medium (10% FBS + 2.5 vol% L-Gln solution (200 mM) + 1 vol% MEM nonessential amino acid solution, *v/v*) at 37 °C in a humidified 5% CO_2_ atmosphere. To ensure uniform cell growth, they were passaged at approximately 80% confluence (2−4 days). The medium was removed and the remaining cell lawn washed with PBS (6 mL, 37 °C). By treatment with EDTA (0.1%) in PBS (5 mL, 5 min, 37 °C), the cells were detached and suspended in 5 mL RPMI medium (10% FBS + 2.5 vol% L-Gln solution (200 mM) + 1 vol% MEM nonessential amino acid solution, *v/v*). The suspension was centrifuged (1.300 rpm, 3 min, RT) and the cell pellet resuspended in fresh RPMI medium (10% FBS + 2.5 vol% L-Gln solution (200 mM) + 1 vol% MEM nonessential amino acid solution, *v/v*). A portion of the suspension was transferred to new culture flasks and the volume adjusted to 25 mL with RPMI medium (10% FBS + 2.5 vol% L-Gln solution (200 mM) + 1 vol% MEM nonessential amino acid solution, *v/v*).

U87 cells (*ATCC*, Teddington, UK) were grown in DMEM medium (plus 10% FBS *v/v*) at 37 °C in a humidified 5% CO_2_ atmosphere. To ensure uniform cell growth, they were passaged at approximately 80% confluence (3−4 days). The spent medium was removed and the remaining cell lawn washed with PBS (10 mL, 37 °C). By treatment with trypsin/EDTA (5 mL, 5 min, 37 °C) at 37 °C, the cells were detached and suspended in 20 mL DMEM medium (plus 10% FBS *v/v*). The suspension was centrifuged (1.300 × *g*, 3 min, RT) and the cell pellet resuspended in fresh DMEM medium (6 mL, plus 10% FBS *v/v*, 37 °C). A portion of the suspension was transferred to new culture flasks and the volume adjusted to 25 mL with DMEM medium (plus 10% FBS *v/v*). Cell density was checked regularly in all cases using an inverted microscope.

#### Receptor Affinity Determination

*In vitro* competition studies were performed on CHO_sst2_ cells (Chinese hamster ovary (CHO) cells stably transfected with human sstR2 (epitope-tagged at the *N*-terminal end), provided by Dr. Jenny Koenig, University of Cambridge, Cambridge, United Kingdom), which were seeded (24-well plates, 1.0 × 10^5^ cells/well, DMEM/F12 GlutaMax plus 10% FCS) and incubated at 37 °C for 24 ± 2 h before the experiment. On the day of the experiment, the DMEM/F12 GlutaMax medium (plus 10% FCS) was removed and each well was washed with 300 *μ*L of HBSS (supplemented with 1 vol % of bovine serum albumin, = HBSA). After the addition of 200 *μ*L of HBSA, 25 *μ*L/well of HBSA (control, n = 3) or the respective ligand in concentrations ranging from 10^−10^ to 10^−4^ M (n = 3) was added. Subsequently, 25 *μ*L of the radiolabeled reference [^125^I]TOC (1 nM in HBSA) was added to each well. After incubation at RT for 1 h, the supernatant was removed, washed with ice-cold PBS (300 *μ*L), and the washing solutions were combined with the supernatants. The cells were lysed by adding NaOH (300 *μ*L, 1 M). The cell lysate is removed after incubation at RT for 20 min and washed with NaOH (300 *μ*L, 1 M), while both NaOH-containing fractions were combined. Subsequently, the activities of both the supernatant and the lysate were measured separately in a *γ*-counter and the *IC*_50_ value was calculated using GraphPad Prism software (*GraphPad Prism 4.0 Software Inc*., La Jolla, California, USA).

### Stability Studies in Human Serum

5 MBq of the respective ^18^F-/^177^Lu-labeled compound was added to 200 *μ*L of human serum (from a healthy volunteer) and incubated at 37 °C for 1 h. After the addition of 50 vol % of cold ethanol and 150 vol % of cold MeCN, centrifugation was performed at 13.000 rpm for 20 min. The supernatant was decanted and centrifuged at 13.000 rpm for 10 min in a centrifuge tube with a 0.45 *μ*m cellulose acetate filter. Approximately MBq of the remaining filtrate was injected into RP-HPLC and the number of intact radioligand was quantified.

### Western Blotting

Western Blots were carried out using an iBind Flex system (*invitrogen*) for primary and secondary antibody immunoblotting. For cell lysate collection, 10 mL of AR42J cells (6.05 × 10^5^ cells/mL) in RPMI media (*ThermoFisher*) and 10 mL of U87 cells (1.21 × 10^6^ cells/mL) in DMEM (*Sigma Life Sciences*) were seeded each in a 10 cm dish 1 day prior to harvesting and incubated at 37 °C (5% CO_2_). During lysate preparation, the dish and the buffers were kept on ice. Media was removed from the dish and the cells were washed with PBS (3 × 5 mL, *Sigma Life Sciences*). The cells were lysed with 400 *μ*L of Pierce RIPA buffer (*Thermo Scientif ic*) containing 4 *μ*L of Halt Protease and Phosphatase Inhibitor Cocktail (100 ×) (*Thermo Scientif ic*) and the collected lysates centrifuged at 21.130 × *g* at 4 °C for 10 min (*eppendorf* Centrifuge 5424 R). After cell debris removal, the supernatants were stored at −80 °C until further use. Three biological repeats were performed for each cell line.

### *In Ovo* Evaluation

All *in ovo* experiments were performed at King’s College London using fertilized Dekalb white or brown eggs (*Henry Stewart & co. Ltd*., UK) according to established procedures.^34^ Before use, the eggs were incubated for up to 14 days at 12−14 °C in a wine cooler (*Haller*) with humidified atmosphere. To engraft tumors onto the chick CAM, eggs were cleaned with Brinsea disinfectant (100 ×) and moved to an incubator (*Brinsea*) where they were kept at 38.7 °C and 48% humidity. The first day of incubation at this temperature was classified embryonic day 0 (E0). The incubator trays were slowly tilted from one side to the other until E3 to loosen the CAM from the eggshell. On E3, eggs were removed from the incubator for window cutting. The eggs were rolled to prevent the CAM sticking to the shell and placed on a cushioned holder. Then, they were pierced at the wide base where the air cell is located and approximately 5 mL of albumin was removed through the hole using a syringe with a 19G needle, which was then resealed with scotch magic tape. Next, a square of tape was placed onto the egg surface and four rectangularly arranged holes were punched into the shell through the tape. A rectangular window (1 × 2 cm) was made with a sharp dissection scissors by carefully cutting 3 sides of a rectangle into the shell using the holes for orientation and without damaging the inner shell membrane. The window was sealed with tape and the eggs placed in the incubator again until E7, the day of CAM implantation. AR42J and U87 cells were maintained as described previously and cell culture media was replenished 24 h prior to harvesting. On the day of inoculations (E7), cells were harvested, resuspended in media and aliquots with 3 × 10^6^ cells were prepared. The aliquots were centrifuged for 3 min at 500 × *g*, 4 °C in a Centrifuge 5424 R (*eppendorf*) and stored on ice. Meanwhile, Matrigel Matrix Basement Membrane (*Corning*) was defrosted on ice. The eggs were removed from the incubator, placed on an egg holder and the windows were opened to locate the CAM. After dabbing the CAM dry with a sterile lens tissue, a suspension of the cell pellet in 20 *μ*L of Matrigel was pipetted onto the CAM. Then, the eggs were resealed with tape, labeled accordingly and placed in the incubator for another 7 days. On E14 the eggs were removed from the incubator and placed on a cushioned holder. The shell window was enlarged to allow for direct injection of the radiotracer. A CAM vein was cannulated using a pulled glass needle, and 90 *μ*L of a 1 mg/mL solution of the anesthetic medetomidine (*Virbac*) was pipetted on to the surface of the CAM. Eggs were left for 15 min at RT before receiving an intravenous bolus injection of ~3 MB of the labeled radiotracer on the imaging bed (<150 *μ*L), followed by 50 *μ*L PBS (*Sigma Life Science*). After 60 min a static PET scan was acquired using a *Mediso* NanoScan PET/CT system (1−5 coincidence mode; 3D reconstruc-tion; CT attenuation corrected; scatter corrected). The eggs were kept at 37 °C throughout the scan and the embryos were humanely euthanized afterward. CT images were obtained for attenuation correction (180 projections; semicircular acquisition; 50 kVp; 300 ms exposure time). The acquired PET data was reconstructed (Tera-Tomo 3D reconstructed algorithm; 4 iterations; 6 subjects; 400−600 keV; 0.3 mm^3^ voxel size) and VivoQuant software (v2.5, *Invicro Ltd*..) was used to analyze the reconstructed images. Regions of interest (ROIs) were drawn manually using the PET signal.

### *Ex Vivo* Biodistribution Studies

Animal experiments were performed by certified personnel following a previously published method.^36^ Experiments were performed in agreement with the general animal welfare regulations in Germany (German Animal Welfare Act, as published on May 18, 2006, as amended by Article 280 of June 19, 2020, permit no. ROB-55.2-2532.Vet_02-18-109 by the *General Directorate of Upper Bavaria*) and institutional guidelines for the care and use of animals. Specifically, female CD1-nu/nu mice aged 5−6 weeks (*Charles River Laboratories International Inc*., Sulzfeld, Germany) were acclimated in the in-house animal facility for 1 week prior to inoculation. Tumor xenografts were generated using AR42J cells (7.0 × 10^6^ cells per 200 *μ*L) suspended in a 1/1 mixture (*v/v*) of RPMI 1640 medium and Cultrex Basement Membrane Matrix Type 3 (*Trevigen*, Gaithersburg, MD, USA). This suspension was inoculated subcutaneously onto the right shoulder and animals were used when tumor volume was >100 mm^3^ (1−2 week after inoculation). Exclusion criteria for animals from an experiment were either weight loss greater than 20%, tumor size greater than 1500 mm^3^, tumor ulceration, respiratory distress, or behavioral change. None of these criteria applied to any of the animals from the trial. No randomized or blinded approach was used in the allocation of the experiments. Health status is SPF according to the FELASA recommendation. Biodistribution studies (n = 3) were performed after 1 h p.i.. For all ^18^F-labeled compounds, approximately 2−3 MBq (300 pmol) were administered intravenously. Mice were sacrificed at 1 h after injection, and radioactivity measurements of tissue samples were performed using WIZARD 2480 automatic *γ*-counter. Collected data were statistically analyzed using Excel (*Microsoft Corporation*, Redmond, WA, USA) and OriginPro software (version 9.7) from *OriginLab Corporation* (Northampton, MA, USA).

## Supplementary Material

Supplementary data

Supplementary information

## Abbreviations

### Abbreviations

CAM: chorioallantoic membrane
2-CTC: 2-Chlorotrityl chloride
DCM: dichloromethane
DIPEA: *N,N*-diisopropyle-thylamine
DMEM: Dulbecco’s modified Eagle’s medium
DMF: dimethylformamide
DMSO: dimethyl sulfoxide
DOTA(^*t*^Bu)_3_: 1,4,7,10-Tetraazacyclododecane-1,4,7,10-tetra-acetic acid 1,4,7-tri-*tert*-butyl
DOTA(^*t*^Bu)_2_: *trans*-(di-*tert*-butyl)-1,4,7,10-tetraazacyclododecane-1,4,7,10-tetraacetic acid
ESI-MS: electrospray ionization mass spectrometry
Fmoc: fluorenylmethoxycarbonyl
GS: general section
HATU: hexafluorophosphate azabenzotriazole tetramethyl uranium
HOAt: 1-Hydroxy-7-azabenzotriazole
HR-ESI-MS: high-resolution electrospray mass spectrometry
HSA: human serum albumin
IE: isotopic exchange
*IC*_50_: half maximal inhibitory concentration
log*D*_pH=7.4_: octanol−PBS partition coefficient
MeCN: acetonitrile
MeOH: methanol
NMP: 1-Methylpyrrolidin-2-one
O_2_Oc: 8-Amino-3,6-dioxaoctanoic acid
PBS: phosphate-buffered saline
RCC: radiochemical conversion
RCP: radiochemical purity
RCY: radiochemical yield
RP-HPLC: reverse phase high performance liquid chromatography
RT: room temperature
SPPS: solid-phase peptide synthesis
SST: somatostatin
sstR2: somatostatine receptor subtype 2
*t*_R_: retention time
TATE: (Tyr^3^)-octreotate
TLC: thin-layer chromatography
TOC: (Tyr^3^)-octreotide
