# Supplementary material for: (SiFA)SeFe: A Hydrophilic Silicon-Based Fluoride Acceptor Enabling Versatile Peptidic Radiohybrid Tracers
Source: J Med Chem. Author manuscript; Available in PMC 2024 Aug 27. (PMC11345769; doi:10.1021/acs.jmedchem.4c00924)
Supplement: Supplementary information [file EMS198000-supplement-Supplementary_information.pdf]

Supporting Information Available

(SiFA)SeFe: A Hydrophilic Silicon-based Fluoride Acceptor Enabling Versatile Peptidic Radiohybrid Tracers

Sandra Deiser [a,b,§], Sebastian Fenzl [b,c,§], Victor König [b], Marike Drexler [a,b], Lydia M. Smith [d], Madeleine E. George,[d] Roswitha Beck [b] Timothy H. Witney [d], Shigeyoshi Inoue*[,c] and Angela Casini*[,a,b]

[a] Mrs. S. Deiser, Ms. M. Drexler, Prof. Dr. A. Casini
Chair of Medicinal and Bioinorganic chemistry, Department of Chemistry, School of Natural Sciences
Technical University of Munich
Lichtenbergstr. 4, 85748 Garching b. München, Germany
E-mail: angela.casini@tum.de

[b] Mr. V. König, Dr. R. Beck
Chair of Pharmaceutical Radiochemistry, Department of Chemistry, School of Natural Sciences
Technical University of Munich
Walther-Meißner-Str. 3, 85748 Garching b. München, Germany

[c] Mr. S. Fenzl, Prof. Dr. S. Inoue
Institute of Silicon Chemistry, Department of Chemistry, School of Natural Sciences
Technical University of Munich
Lichtenbergstr. 4, 85748 Garching b. München, Germany
E-mail: s.inoue@tum.de

[d] Dr. L.M. Smith, Ms. M.E. George, Dr. T. Witney
School of Biomedical Engineering and Imaging Sciences
King's College London
St Thomas' Hospital, London SE1 7EH London, UK

Contents

1. Characterization of the Fmoc-(SiFA)SeFe and precursors ... 3
   1.1. NMR-spectrometry ... 3
   1.2. Mass Spectrometry ... 17
2. Characterization and evaluation of the model (SiFA)SeFe-bioconjugates. ... 18
   2.1. RP-HPLC chromatograms ... 19
   2.2. Mass Spectrometry ... 27
   2.3. Radiochemical characterization ... 31
   2.4. Radiochemical conversion ... 40
   2.5. Stability under lutetium labeling conditions ... 45
   2.6. Stability towards physiological conditions ... 91
3. Characterization of the radiohybrid tracers and their benchmark compounds. ... 114
   3.1. Synthesis of SST binding motif and SST-ligands ... 114
   3.2. RP-HPLC analysis ... 116
   3.3. Mass Spectrometry ... 121
   3.4. Radiochemical characterization ... 126
   3.5. Radio-TLC sstR2 peptides ... 131
   3.6. Optimization of the ^{177}Lu-labeling ... 136
4. Western Blots for sstR2 expression ... 136
5. Supplementary Tables ... 138

1. Characterization of the Fmoc-(SiFA)SeFe and precursors

1.1. NMR-spectrometry

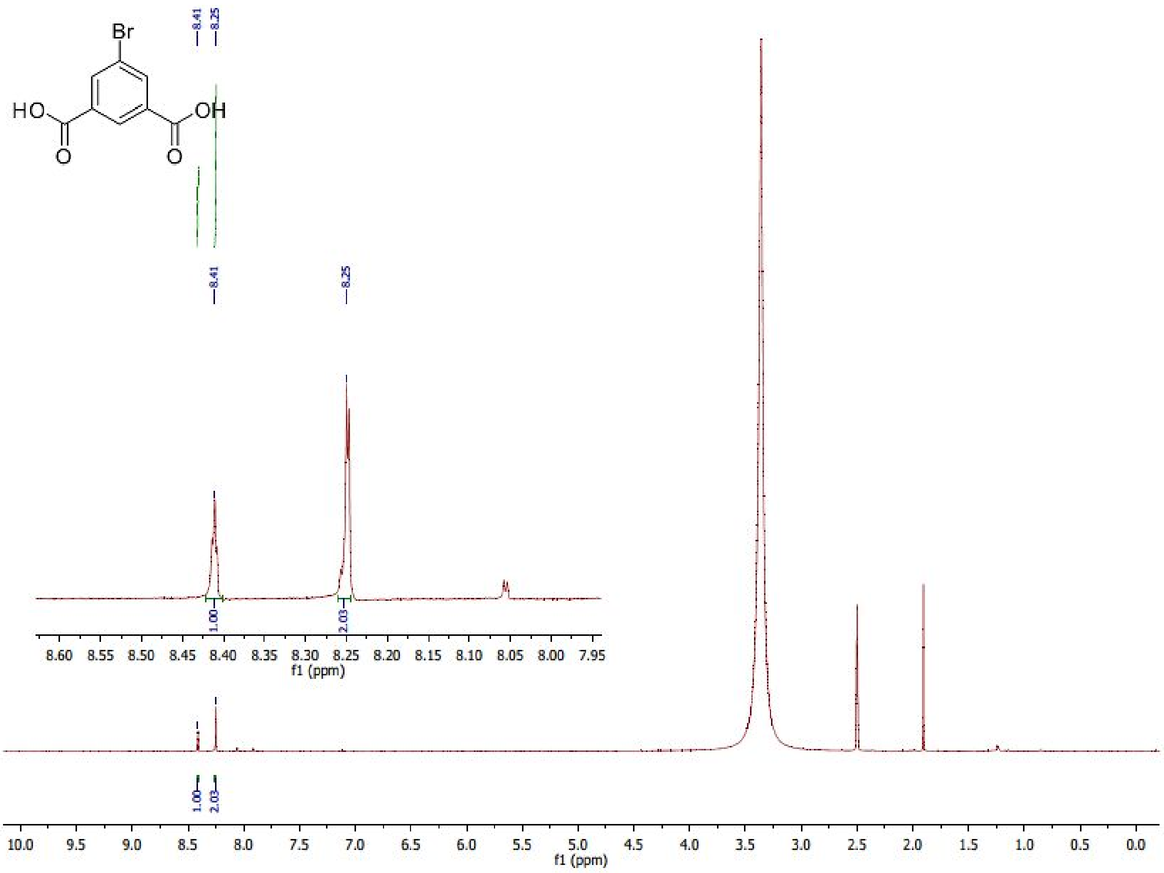

Figure S1: ^{1}H-NMR (400 MHz, DMSO-d_6) spectrum of 5-bromoisophthalic acid (i).

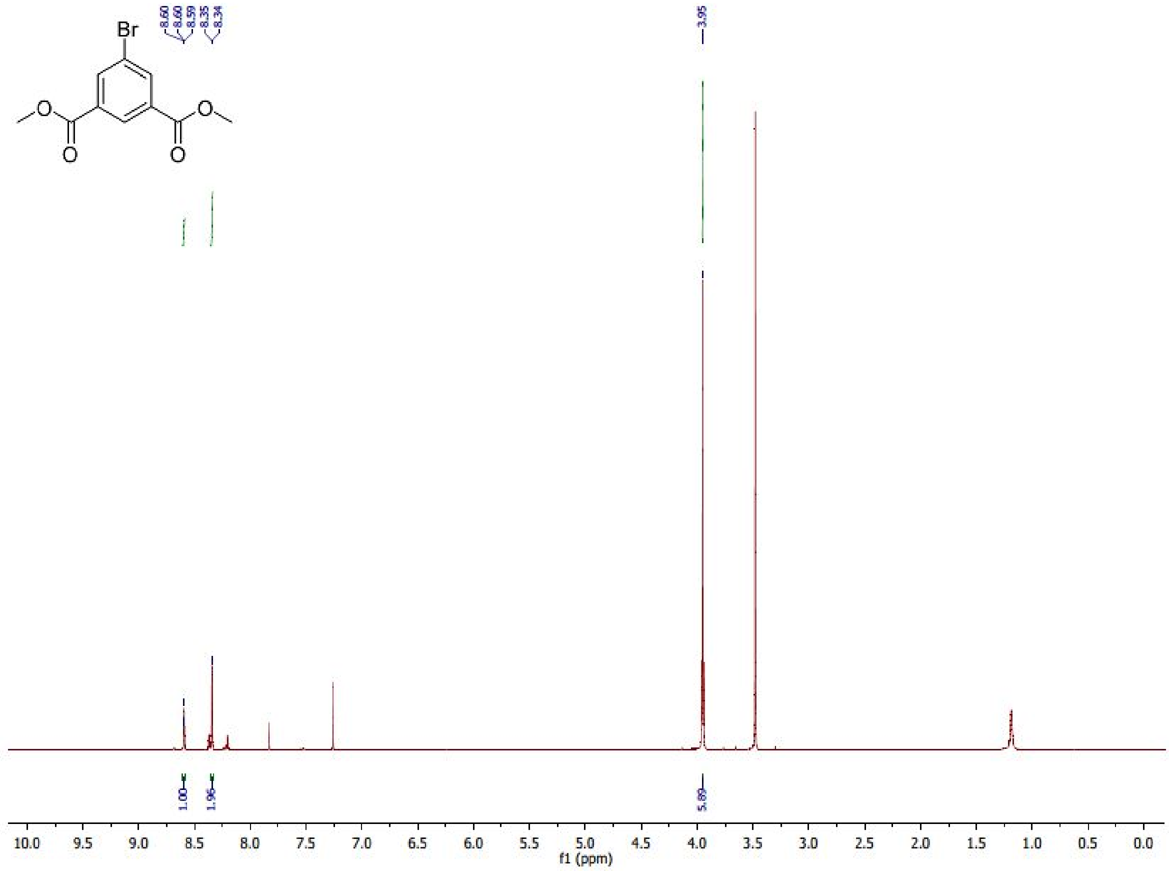

Figure S2: ^{1}H NMR (400 MHz, Chloroform-*d*) spectrum of Dimethyl 5-bromoisophthalate (ii).

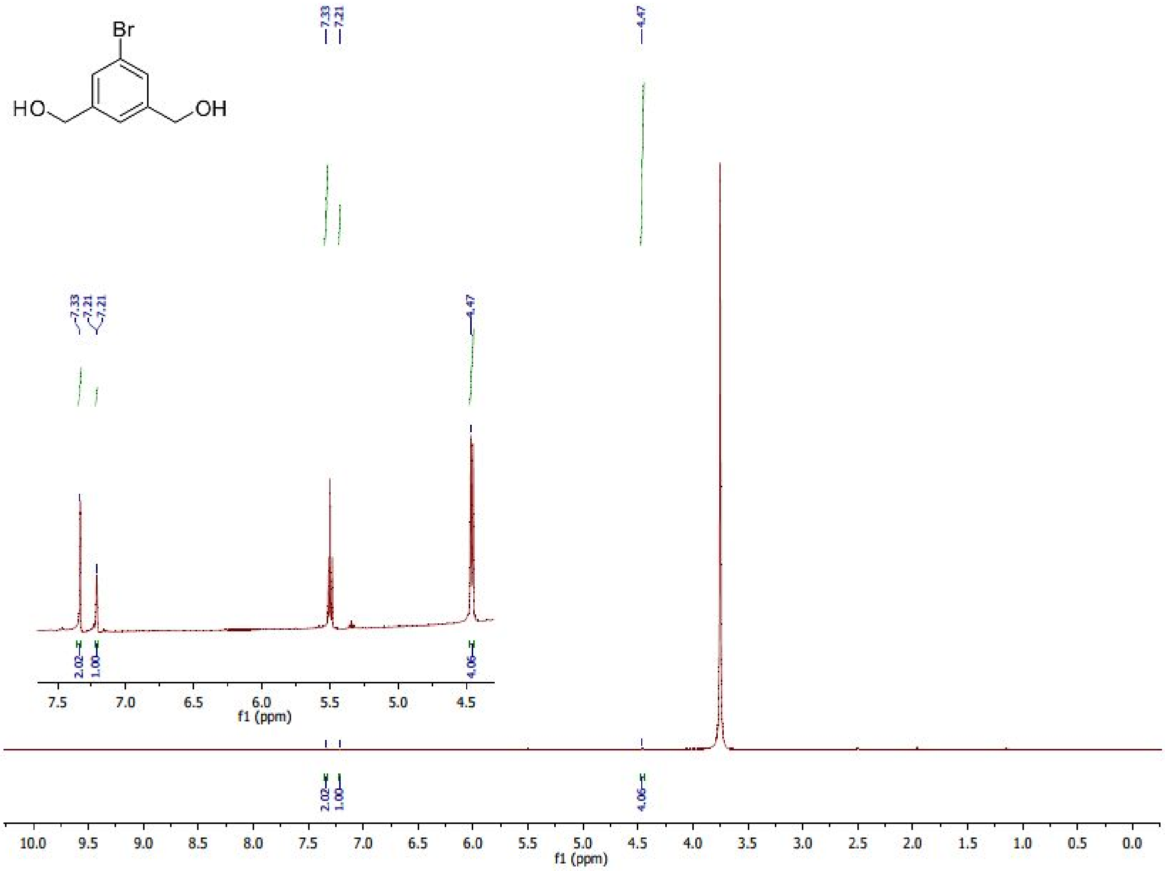

Figure S3: ^{1}H-NMR (400 MHz, DMSO-d_6) spectrum of (5-Bromo-1,3-phenylene)dimethanol (iii).

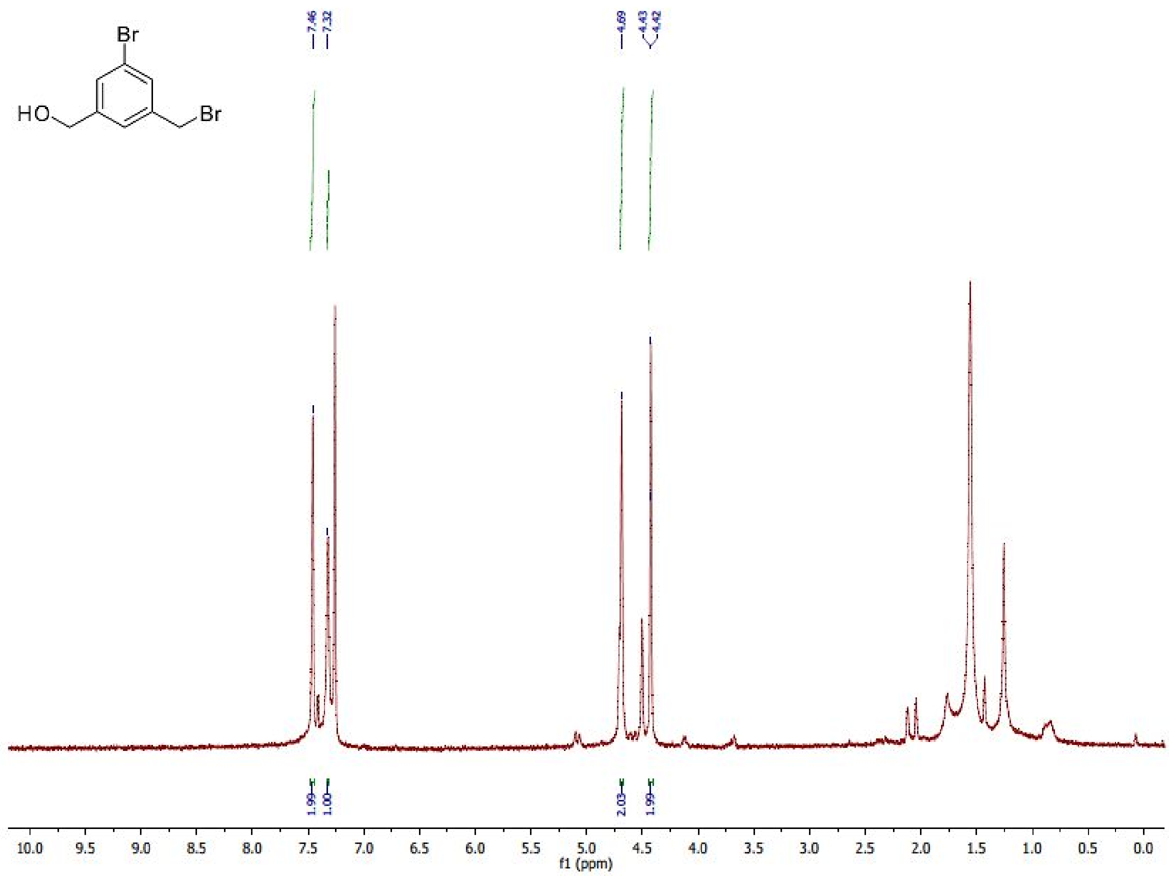

Figure S4: ^{1}H-NMR (400 MHz, Chloroform-*d*) spectrum of (3-bromo-5-(bromomethyl)phenyl)methanol (iv).

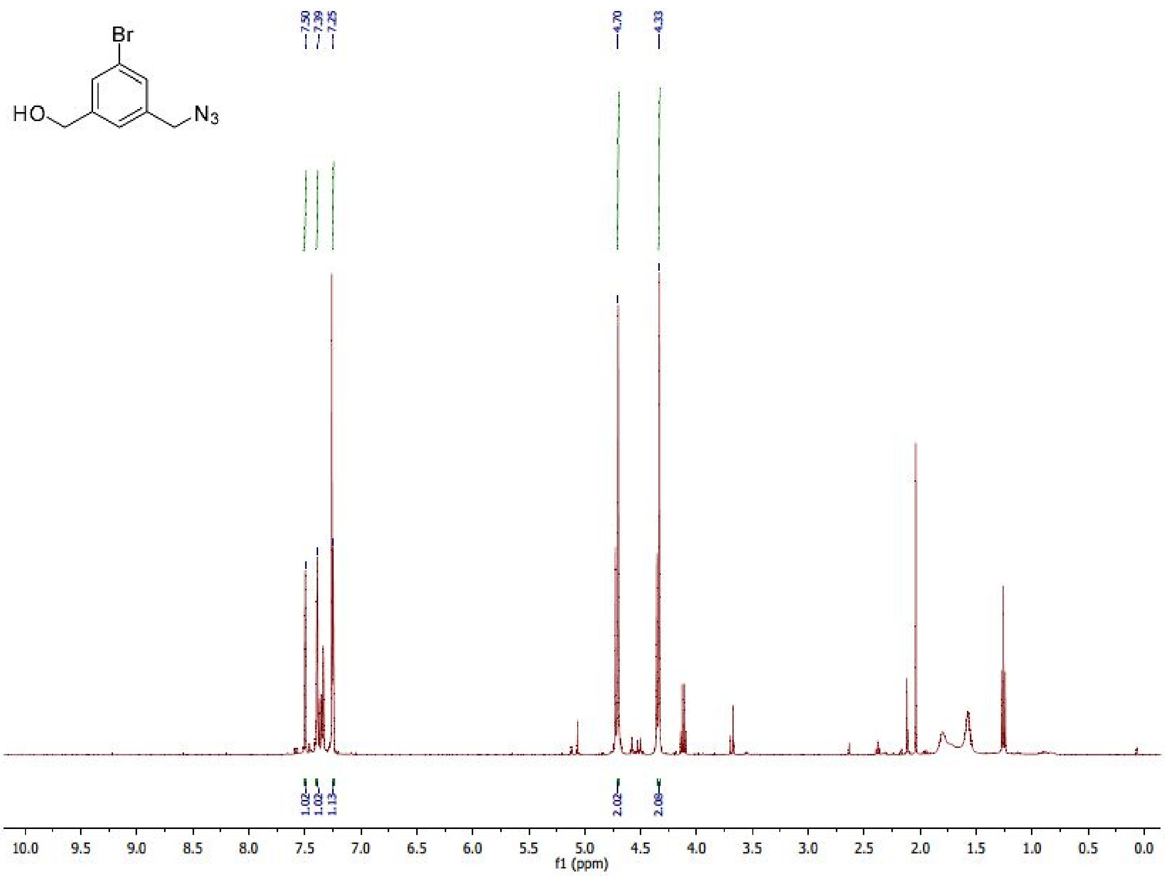

Figure S5: ^{1}H-NMR (500 MHz, Chloroform-*d*) spectrum of (3-(azidomethyl)-5-bromophenyl)methanol (v).

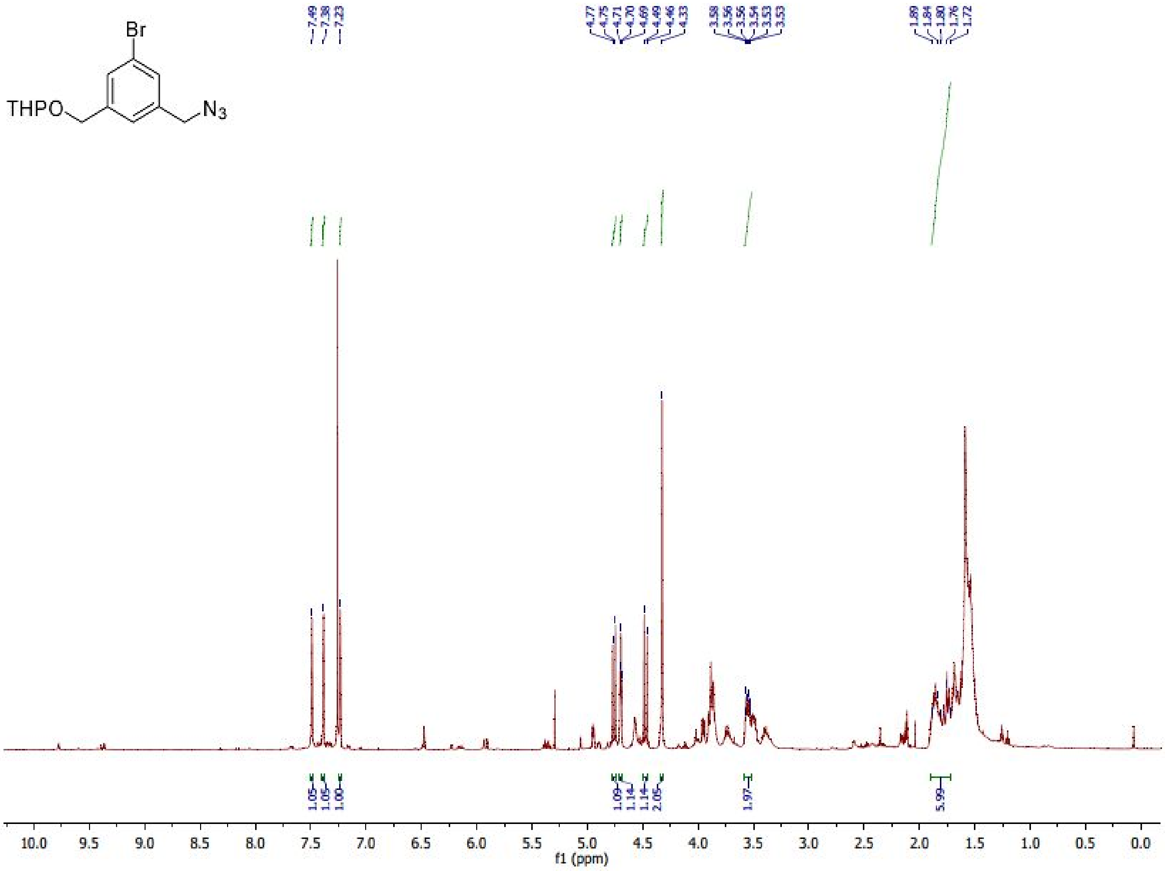

Figure S6: ^{1}H-NMR (500 MHz, Chloroform-*d*) spectrum of 2-((3-(azidomethyl)-5-bromobenzyl)oxy)tetrahydro-2H-pyran (vi).

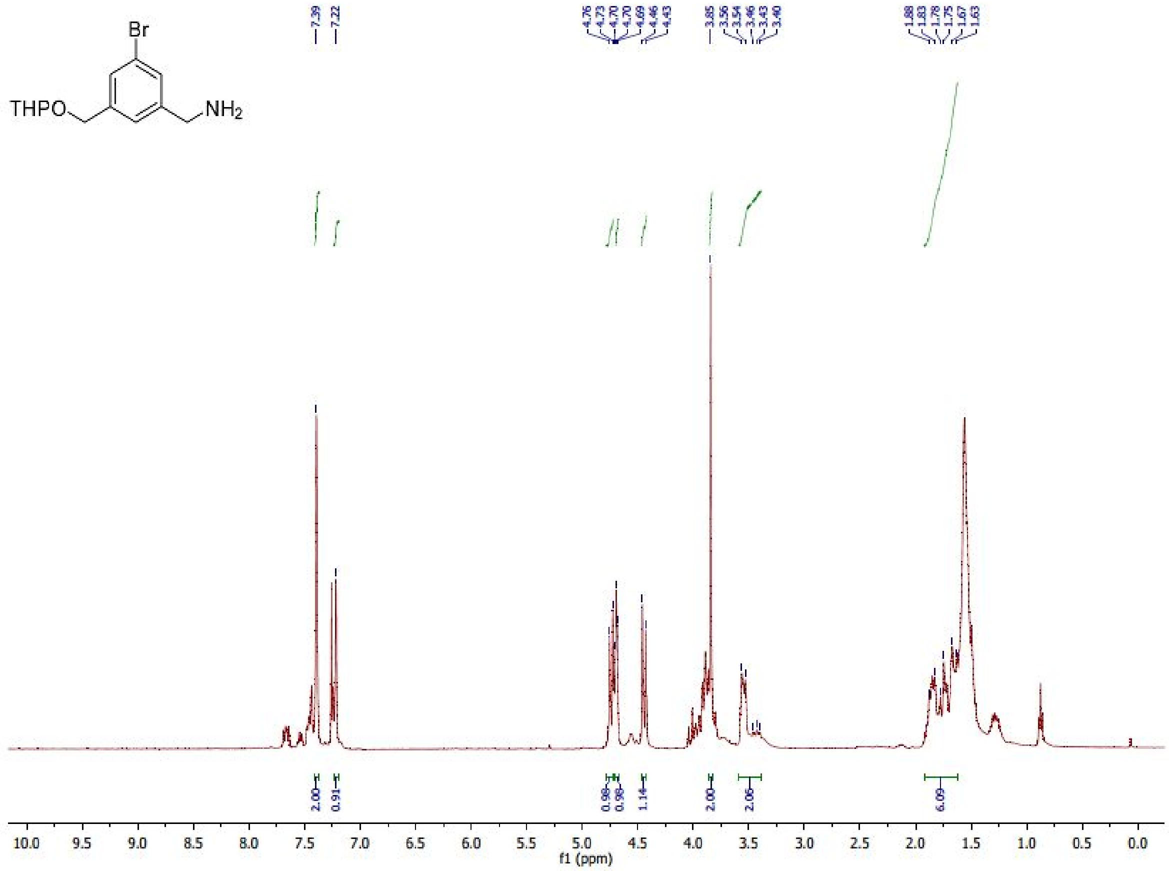

Figure S7: ^{1}H-NMR (400 MHz, Chloroform-*d*) spectrum of (3-Bromo-5-(((tetrahydro-2H-pyran-2-yl)oxy)methyl)phenyl)methanamine (vii).

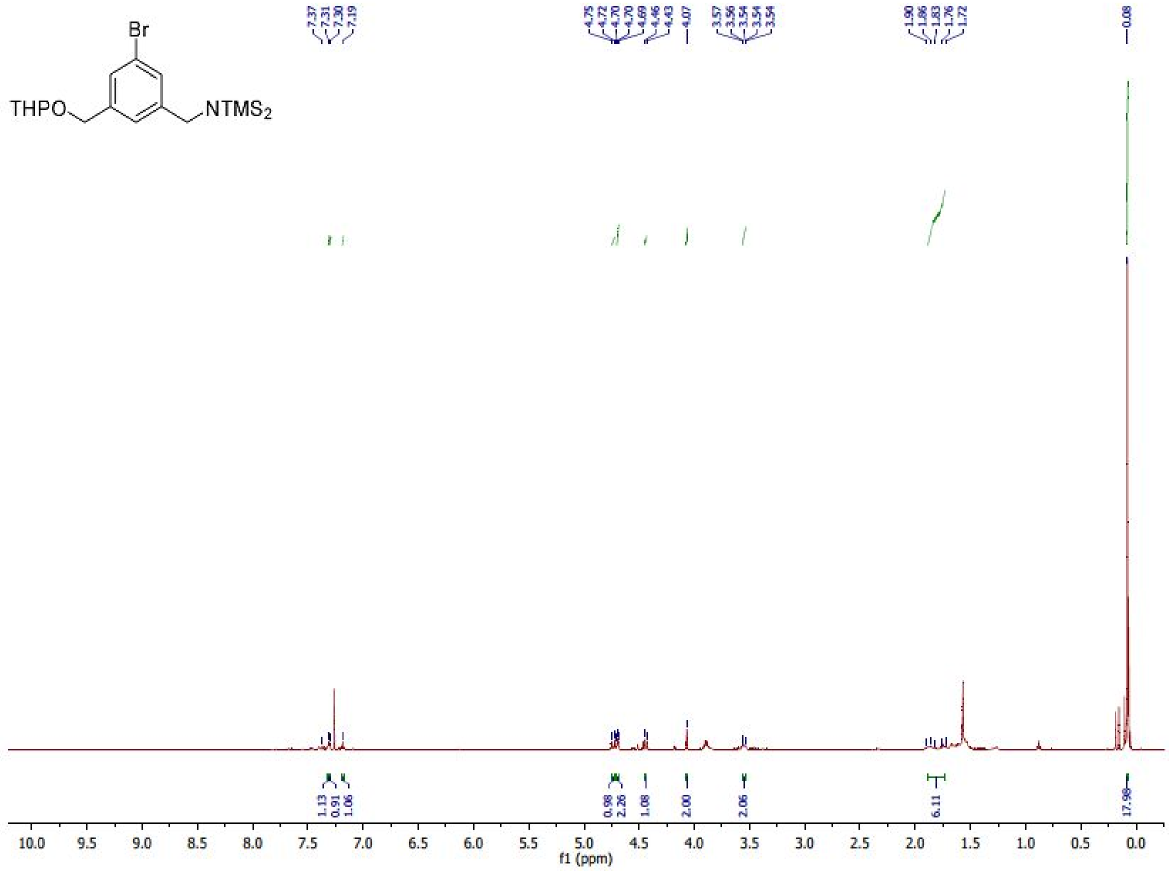

Figure S8: ¹H-NMR (500 MHz, Chloroform-*d*) spectrum of N-(3-Bromo-5-(((tetrahydro-2H-pyran-2-yl)oxy)methyl)benzyl)-1,1,1-trimethyl-N-(trimethylsilyl)silanamine (viii).

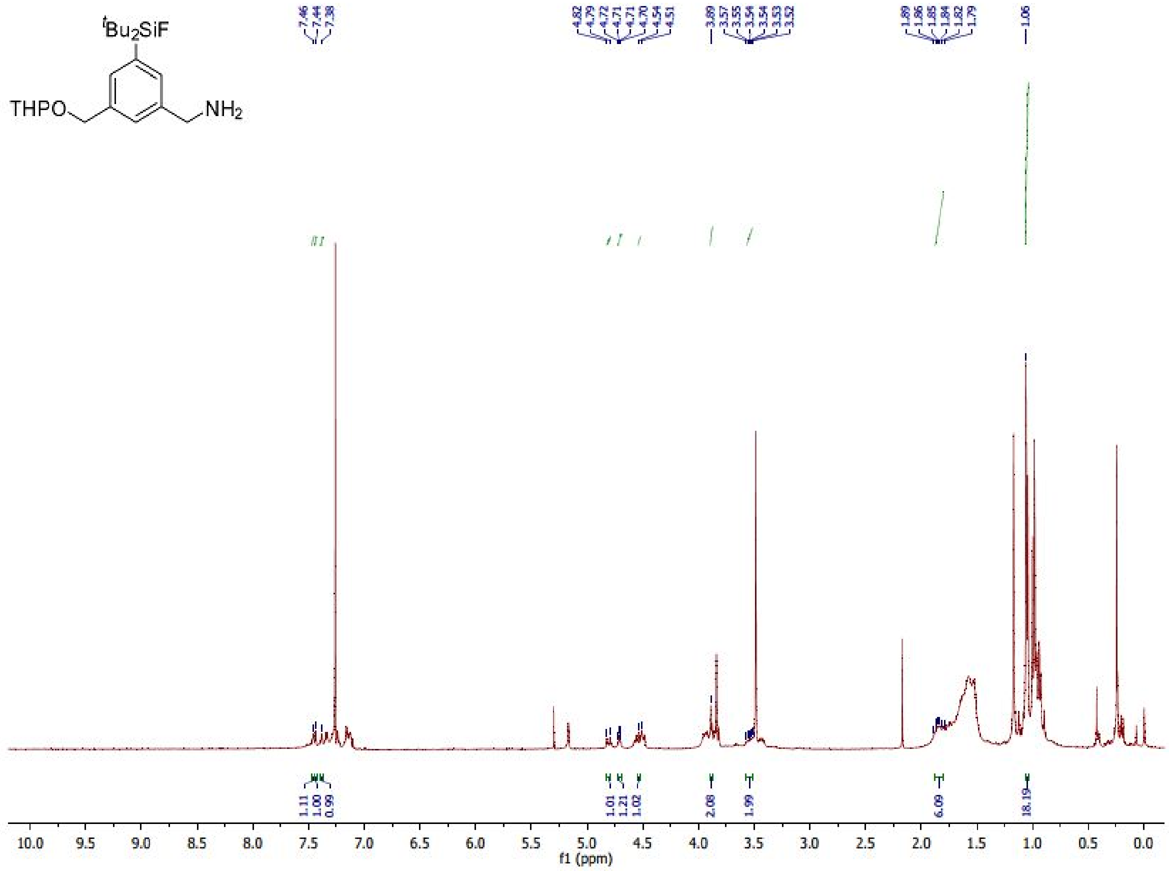

Figure S9: ^{1}H-NMR (400 MHz, Chloroform-*d*) spectrum of (3-(Di-tert-butylfluorosilyl)-5-(((tetrahydro-2H-pyran-2-yl)oxy)methyl)phenyl)methanamine (ix).

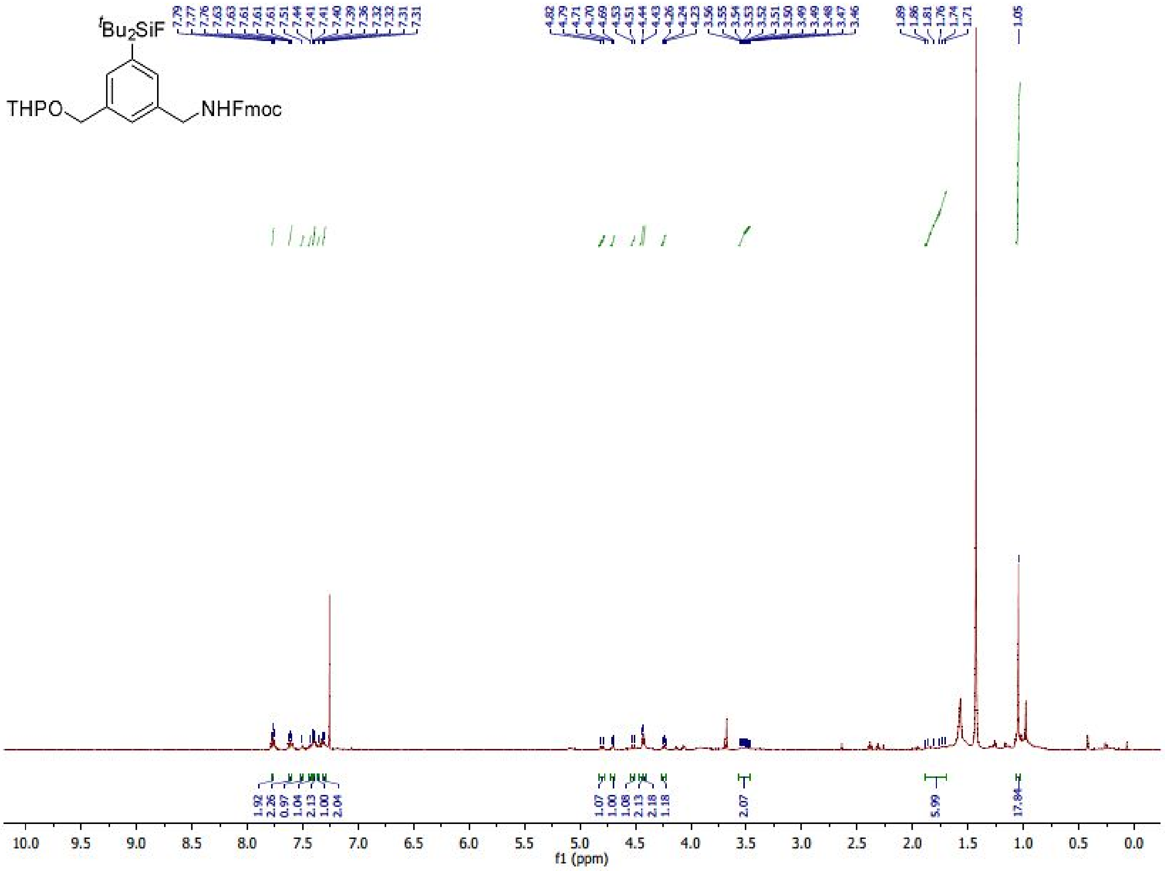

Figure S10: ^{1}H-NMR (500 MHz, Chloroform-*d*) spectrum of (9H-Fluoren-9-yl)methyl-(3-(di-tert-butylfluorosilyl)-5-(((tetrahydro-2H-pyran-2-yl)oxy)methyl)benzyl)carbamate (x).

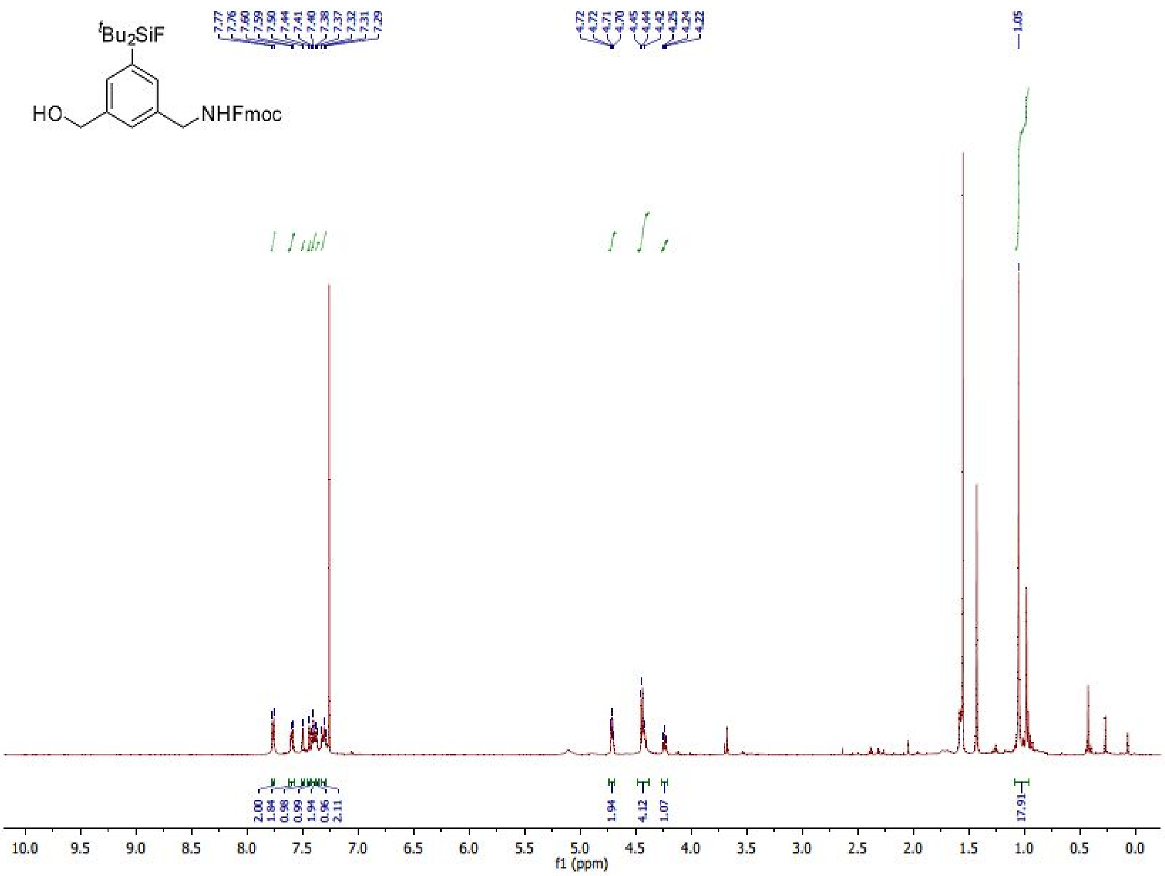

Figure S11: ^{1}H-NMR (500 MHz, Chloroform-*d*) spectrum of (9H-Fluoren-9-yl)methyl-(3-(di-tert-butylfluorosilyl)-5-(hydroxymethyl)benzyl)carbamate (xi).

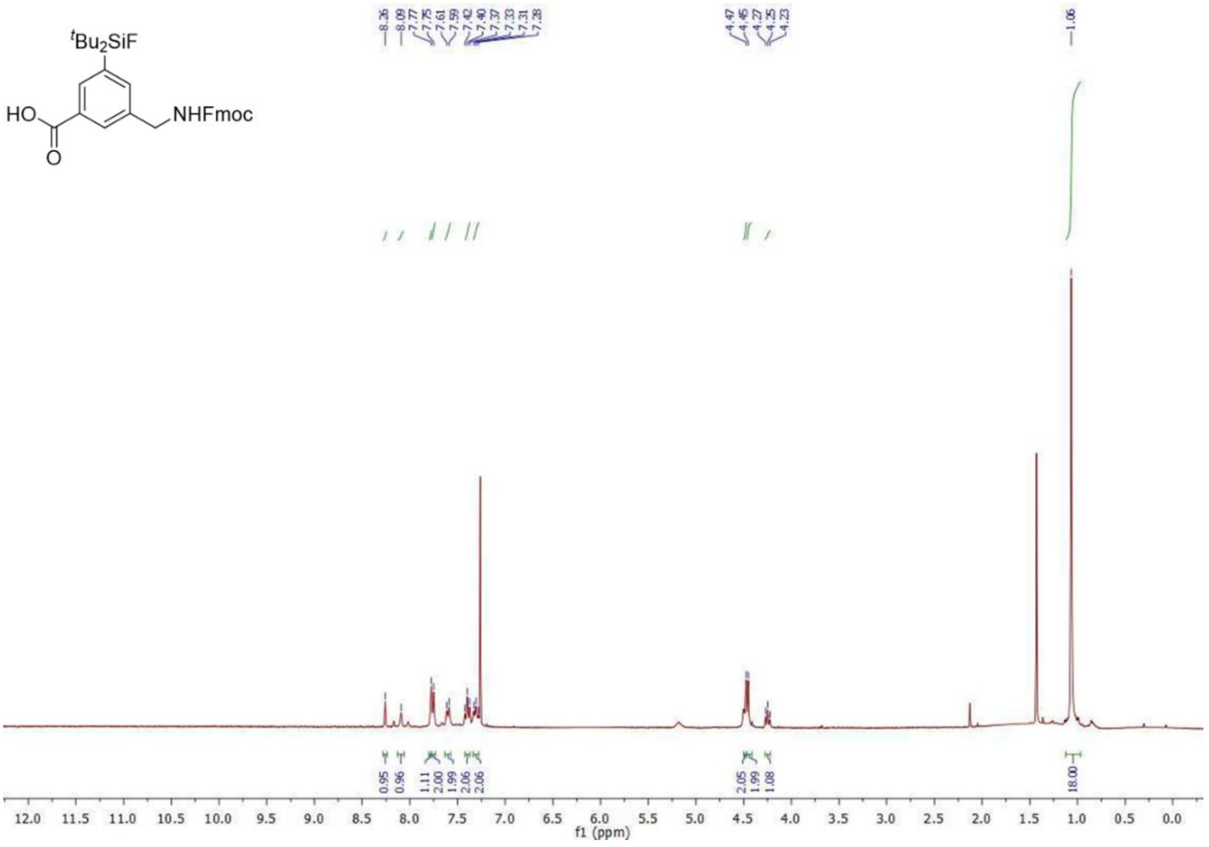

Figure S12: ^{1}H-NMR (500 MHz, Chloroform-*d*) spectrum of 3-(((((9H-Fluoren-9-yl)methoxy)carbonyl)amino)methyl)-5-(di-tert-butylfluorosilyl)benzoic acid (**Fmoc-(SiFA)SeFe**).

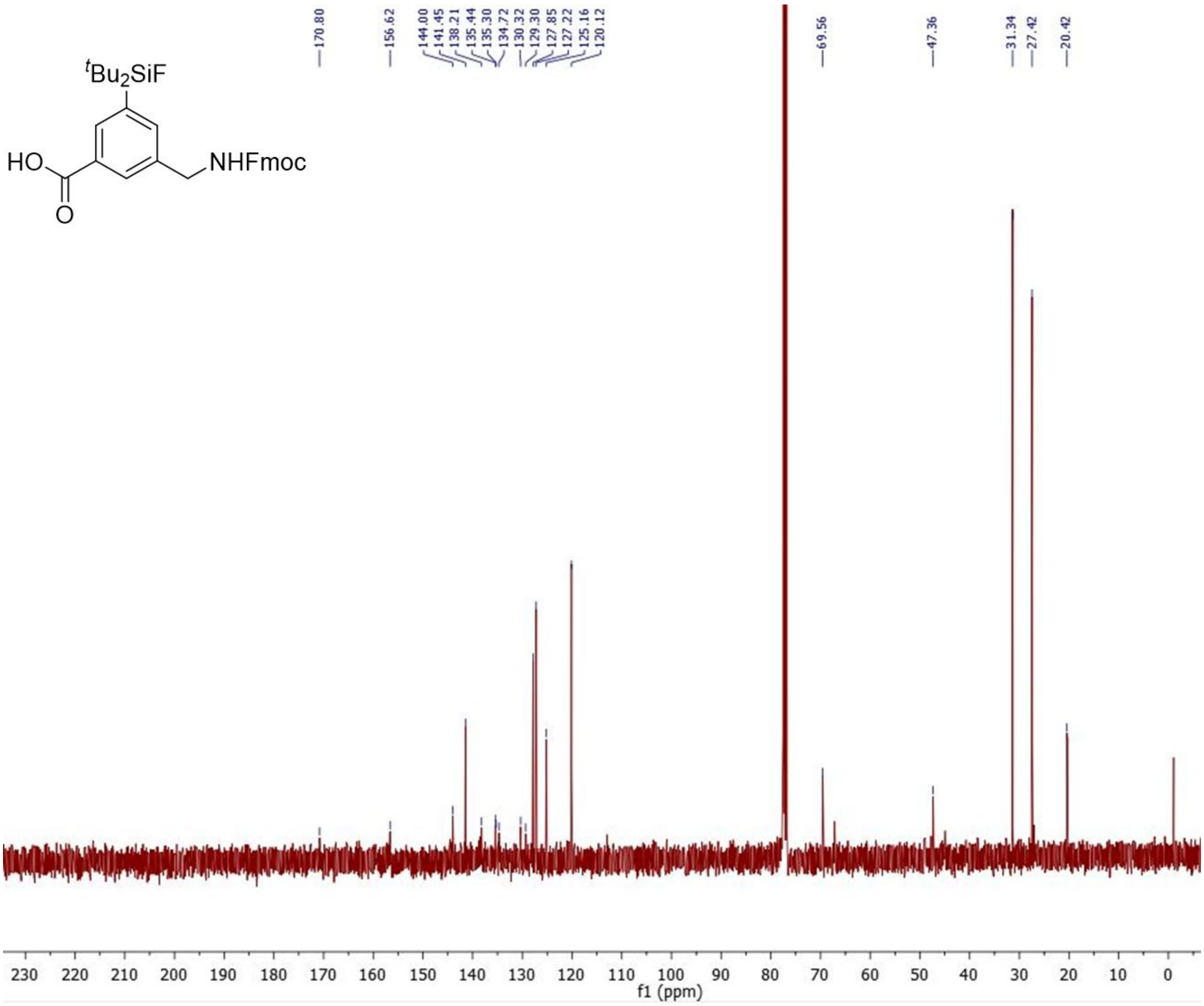

Figure S13: ^{13}C-NMR (101 MHz, Chloroform-*d*) spectrum of 3-(((((9H-Fluoren-9-yl)methoxy)carbonyl)amino)methyl)-5-(di-tert-butylfluorosilyl)benzoic acid (**Fmoc-(SiFA)SeFe**).

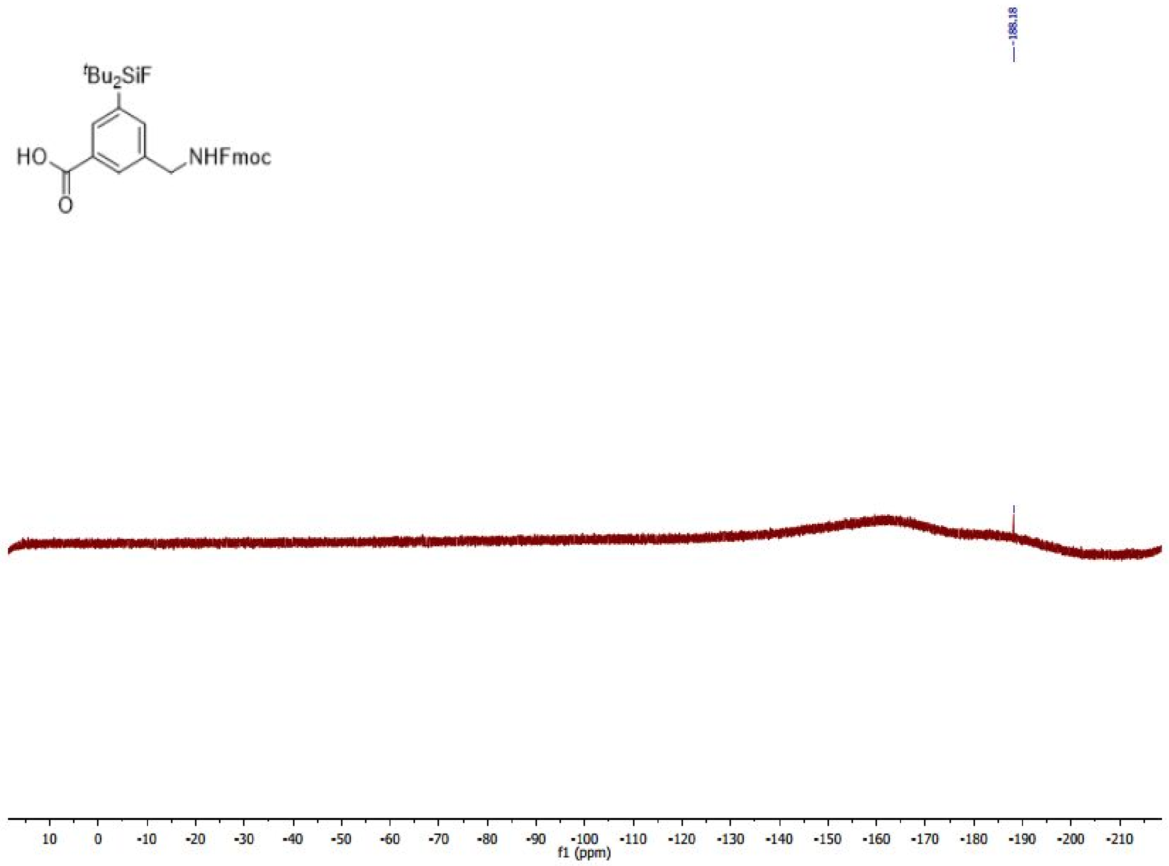

Figure S14: ^{19}F-NMR (376 MHz, Chloroform-*d*) spectrum of 3-(((((9H-Fluoren-9-yl)methoxy)carbonyl)amino)methyl)-5-(di-tert-butylfluorosilyl)benzoic acid (**Fmoc-(SiFA)SeFe**).

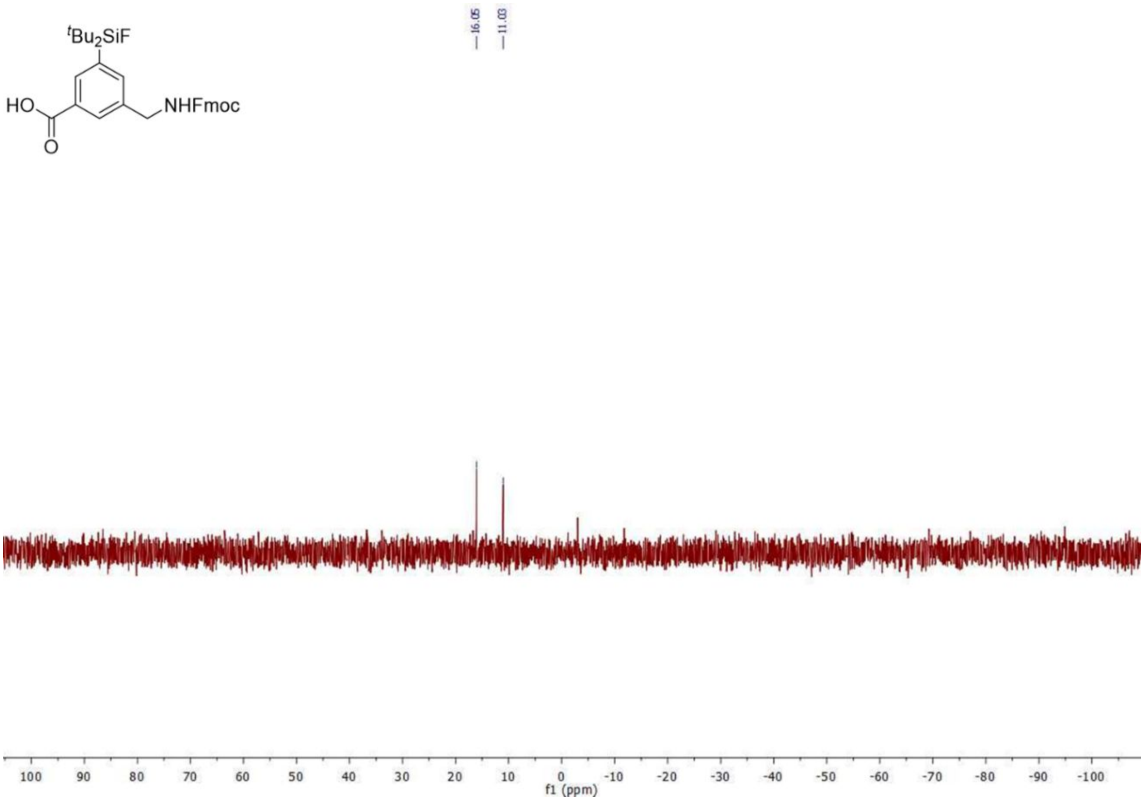

Figure S15: ^{29}Si-NMR (60 MHz, Chloroform-*d*) spectrum of 3-(((((9H-Fluoren-9-yl)methoxy)carbonyl)amino)methyl)-5-(di-tert-butylfluorosilyl)benzoic acid (**Fmoc-(SiFA)SeFe**).

1.2. Mass Spectrometry

Fmoc-(SiFA)SeFe

- $[2M+H^+]^+$ = 1067.4864 (calc. 1067.4866)
- $[M+Na^+]^+$ = 556.2287 (calc. 556.2290)

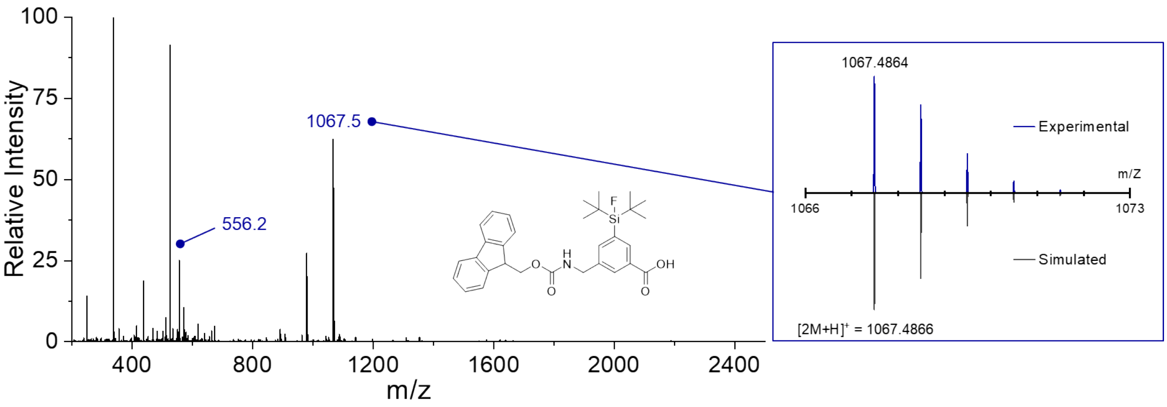

Figure S16: HR-ESI-MS of 3-(((((9H-Fluoren-9-yl)methoxy)carbonyl)amino)methyl)-5-(di-tert-butylfluorosilyl)benzoic acid (**Fmoc-(SiFA)SeFe**).

2. Characterization and evaluation of the model (SiFA)SeFe-bioconjugates.

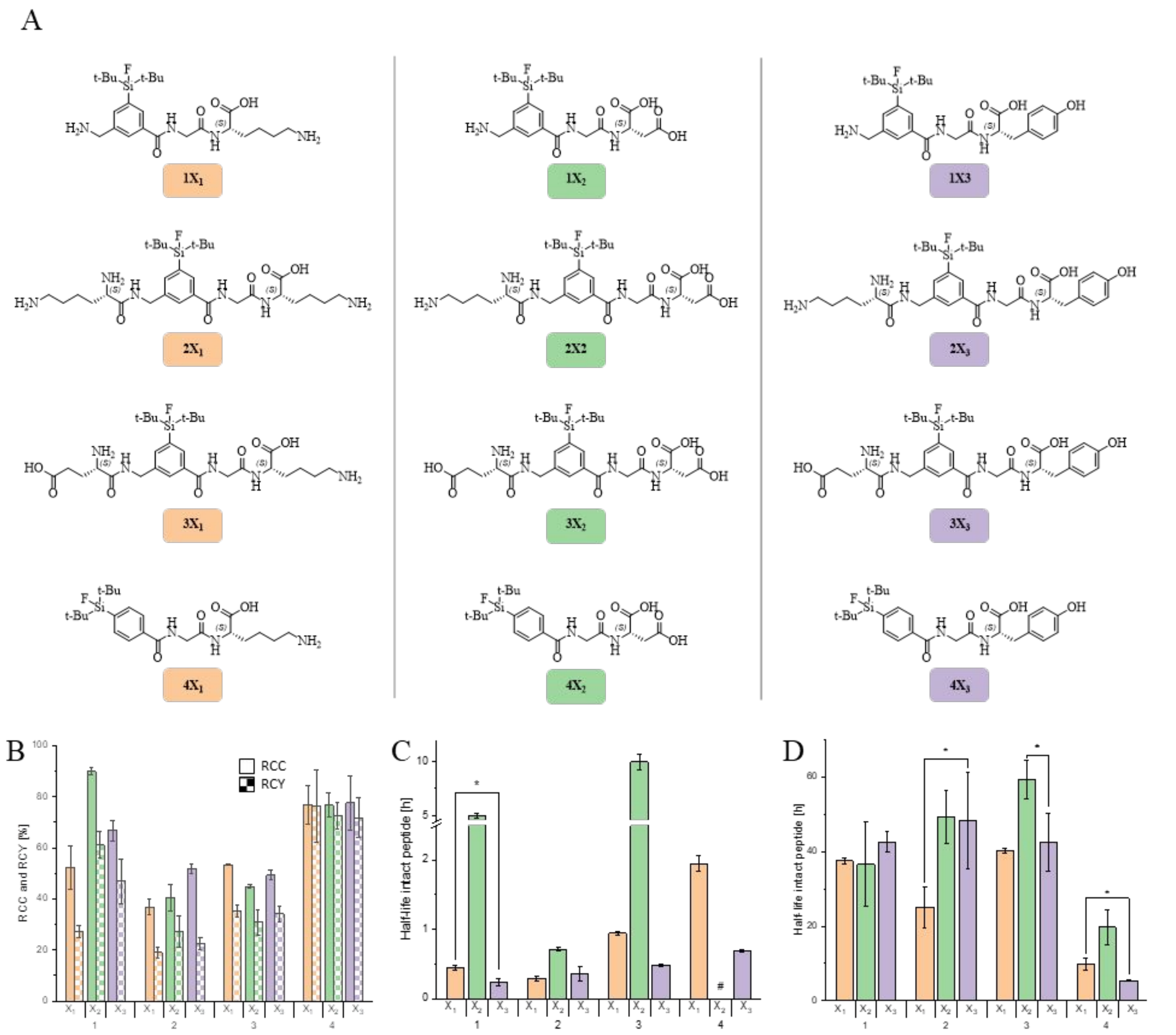

Figure S17: (A) Structures of the model (SiFA)SeFe bioconjugates. Results of the stability studies of model (SiFA)SeFe bioconjugates in comparison to *para*-(SiFA)BA model bioconjugates: (B) Results of ^{18}F-labeling expressed in terms of radiochemical conversions (RCCs) and radiochemical yields (RCYs); (C) stability of the ^{18}F-labeled bioconjugates under physiological conditions - pH 7.4, 37 °C, samples taken at t = 0, 30, 60, 90 and 120 min. The half-life was calculated from the ratio between free fluorine-18 and labeled peptide; (D) stability under lutetium labeling conditions of the ^{18}F-labeled bioconjugates - pH 5.5, 90 °C, samples taken at t = 0, 30, 60, 90 and 120 min. The half-life was calculated from the ratio between free fluorine-18 and labeled peptide. *$p \leq 0.05$; # Half-lives not given because of unprecise regression (R^2 << 90%) with stabilities of > 95% intact MP after 2 h.

2.1. RP-HPLC chromatograms

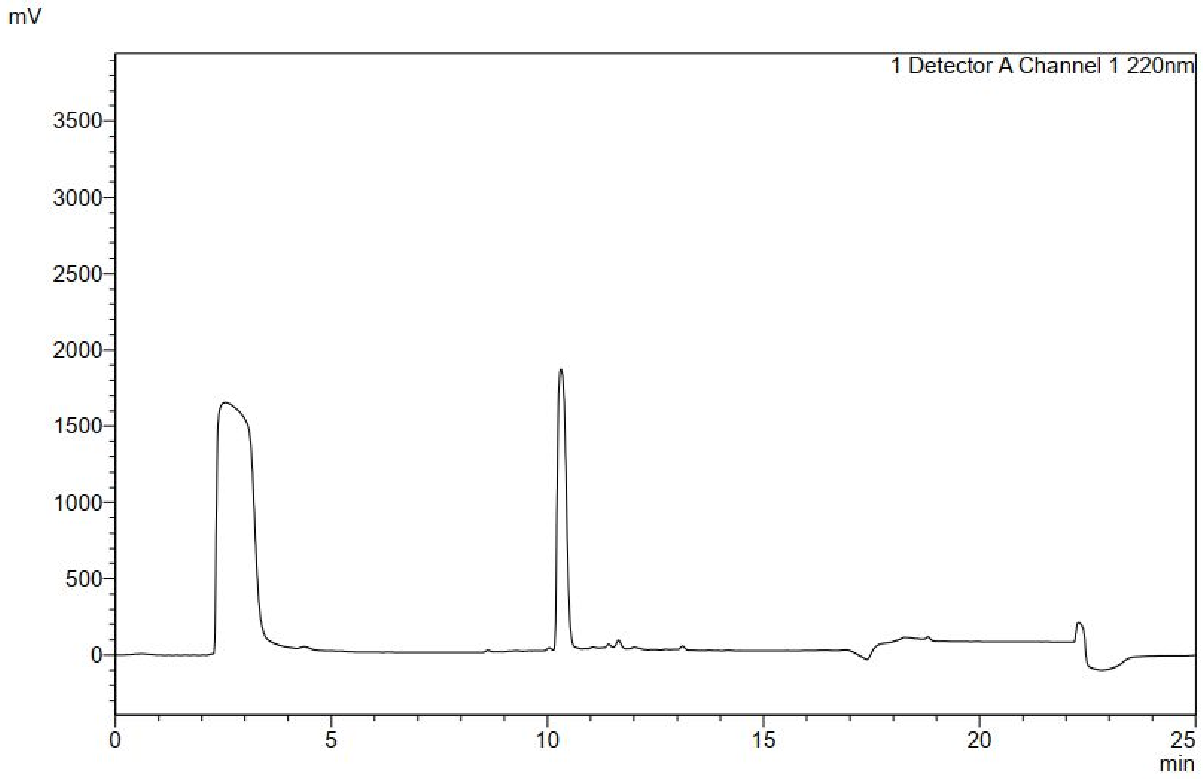

Figure S18: RP-HPLC chromatogram of the reaction control of H_2N-(SiFA)SeFe-Gly-Lys-OH ($1X_1$) using the analytical control method 10-60% B (15 min, λ = 220 nm, MultoKrom® 100-5 C18-column (125 × 4.6 mm, 5 µm particle size, *CS Chromatographie GmbH*, t_R = 10.2 min).

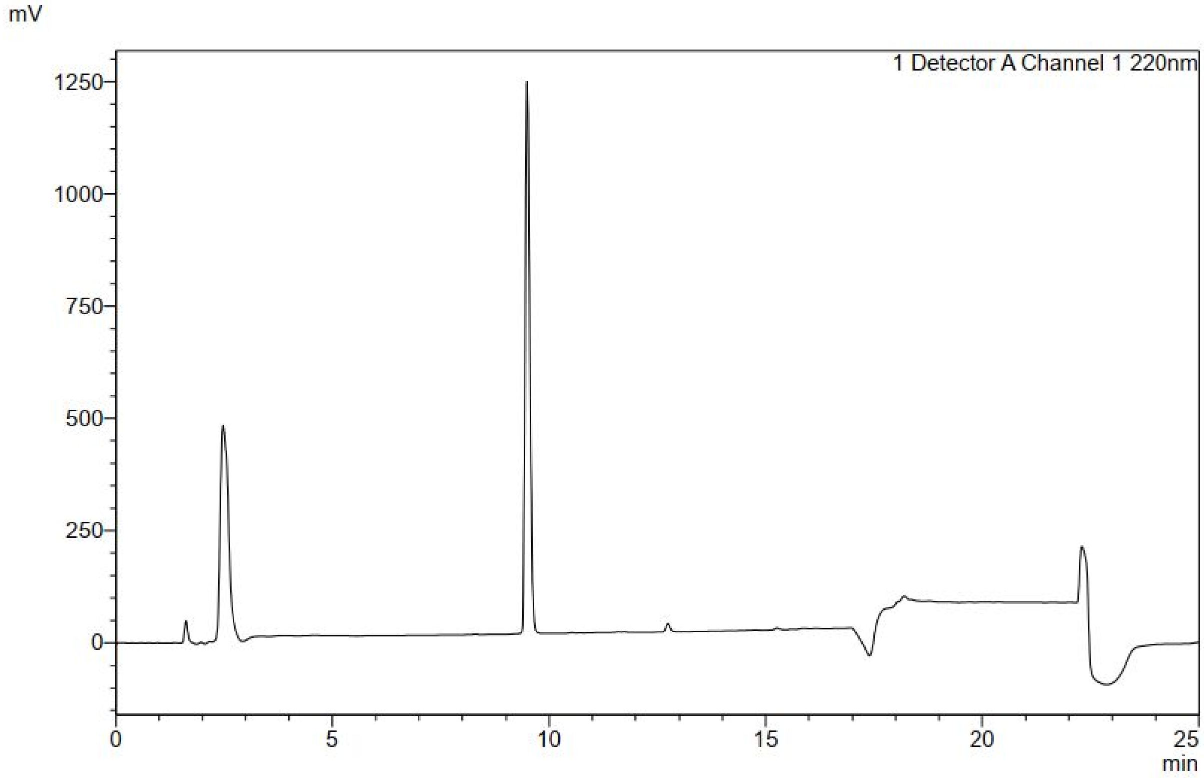

Figure S19: RP-HPLC chromatogram of the reaction control of H_2N-Lys-(SiFA)SeFe-Gly-Lys-OH ($2X_1$) using the analytical control method 10-60% B (15 min, $\lambda = 220$ nm, MultoKrom® 100-5 C18-column (125 × 4.6 mm, 5 µm particle size, *CS Chromatographie GmbH*, $t_R = 9.5$ min).

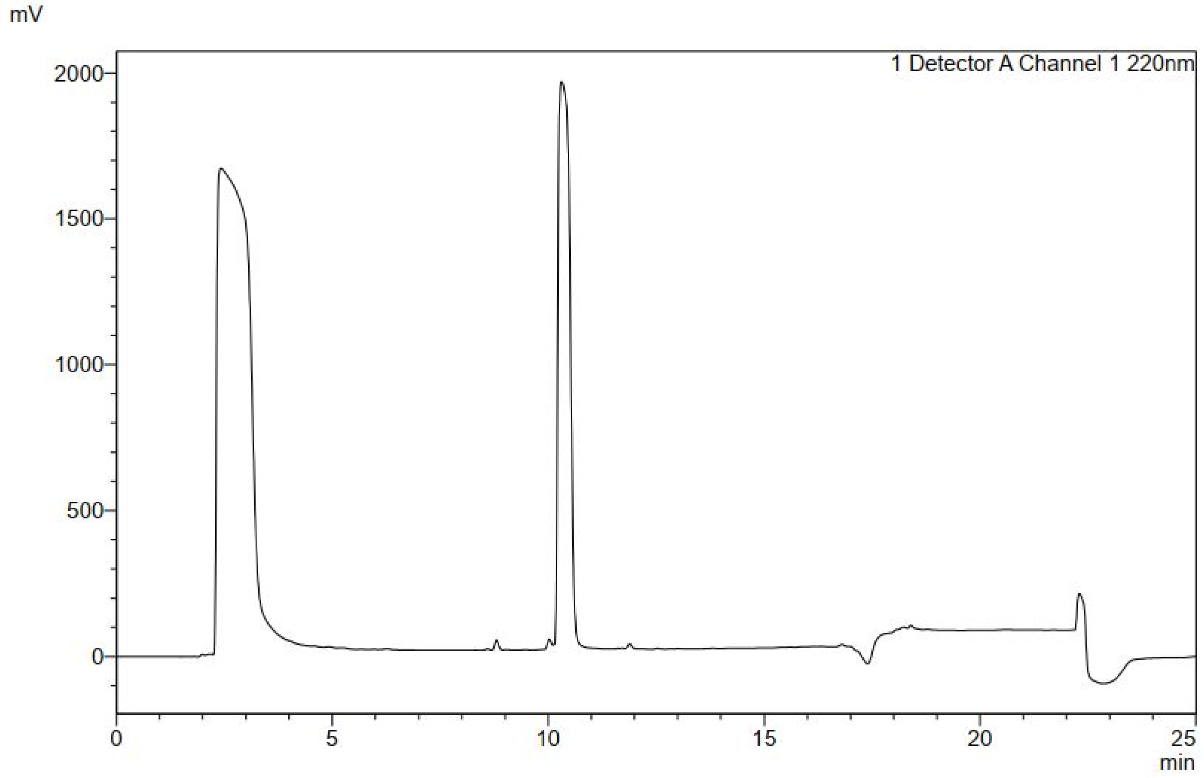

Figure S20: RP-HPLC chromatogram of the reaction control of H_2N-Glu-(SiFA)SeFe-Gly-Lys-OH ($3X_1$) using the analytical control method 10-60% B (15 min, λ = 220 nm, MultoKrom® 100-5 C18-column (125 × 4.6 mm, 5 µm particle size, *CS Chromatographie GmbH*, t_R = 10.4 min).

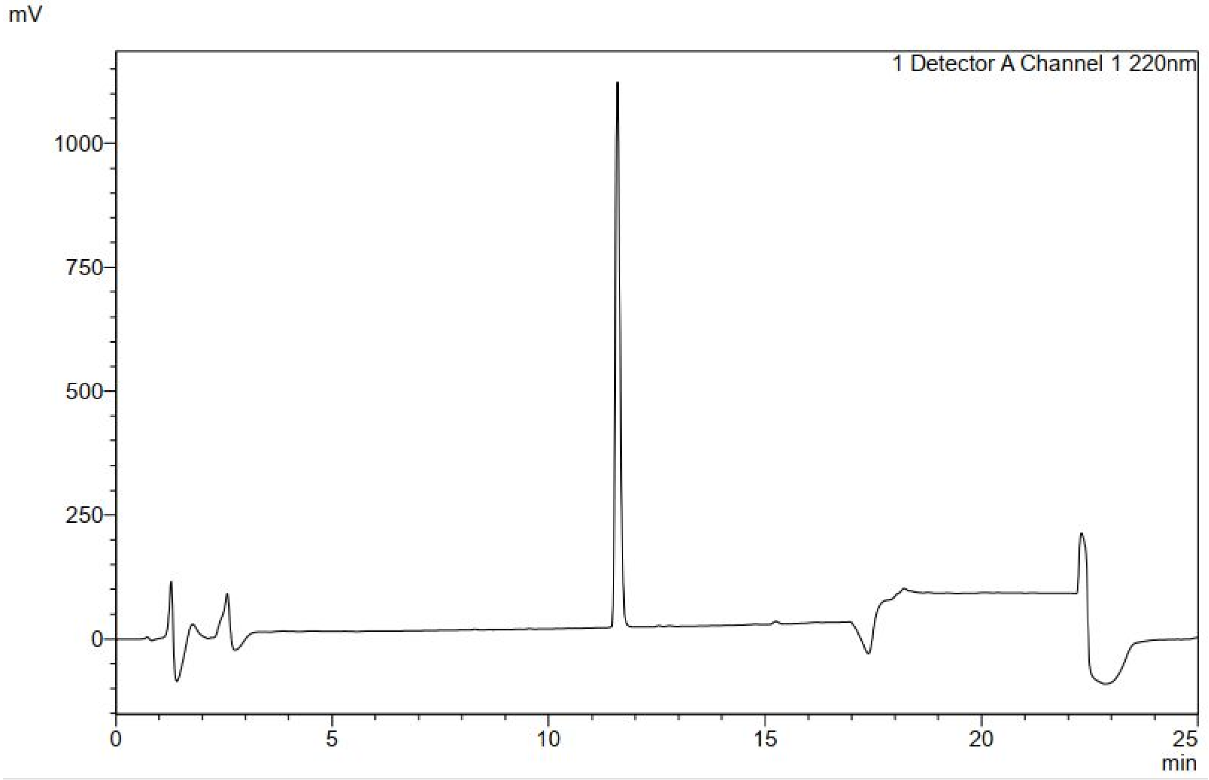

Figure S21: RP-HPLC chromatogram of the reaction control of H_2N-(SiFA)SeFe-Gly-Asp-OH ($1X_2$) using the analytical control method 10-60% B (15 min, λ = 220 nm, MultoKrom® 100-5 C18-column (125 × 4.6 mm, 5 µm particle size, *CS Chromatographie GmbH*, t_R = 11.6 min).

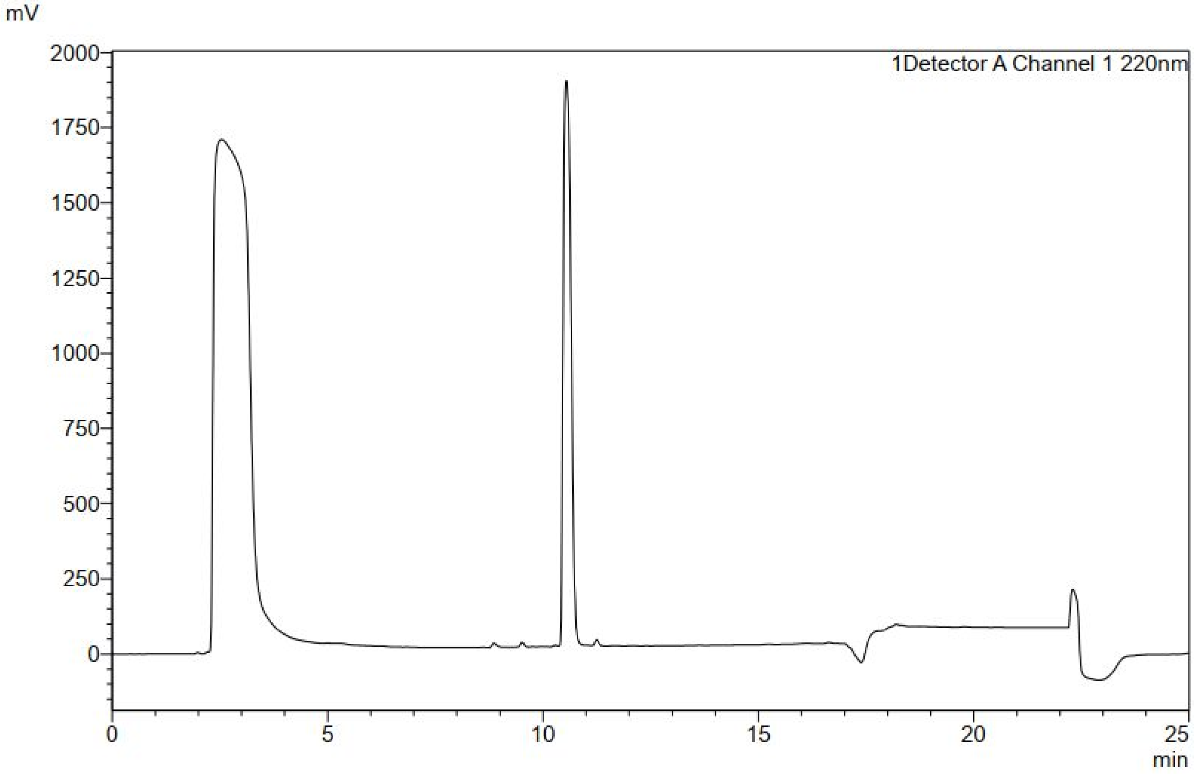

Figure S22: RP-HPLC chromatogram of the reaction control of H_2N-Lys-(SiFA)SeFe-Gly-Asp-OH ($2X_2$) using the analytical control method 10-60% B (15 min, λ = 220 nm, MultoKrom® 100-5 C18-column (125 × 4.6 mm, 5 µm particle size, *CS Chromatographie GmbH*, t_R = 10.6 min).

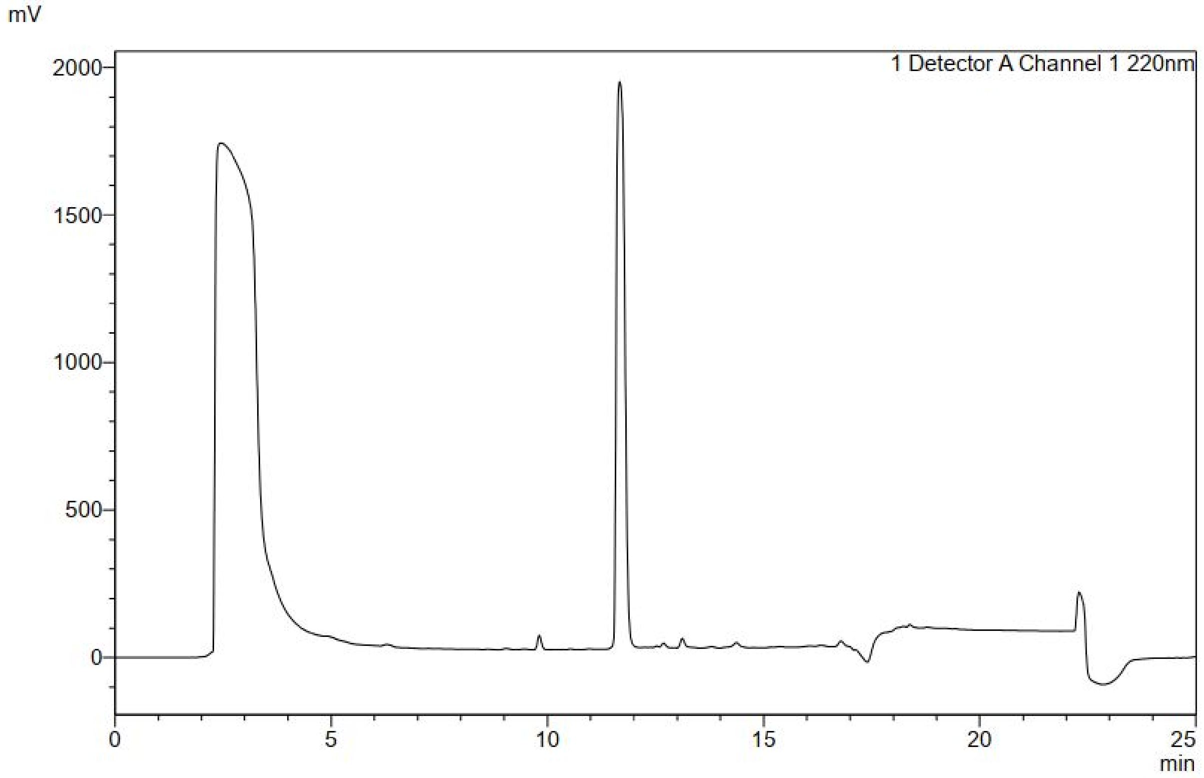

Figure S23: RP-HPLC chromatogram of the reaction control of H_2N-Glu-(SiFA)SeFe-Gly-Asp-OH ($3X_2$) using the analytical control method 10-60% B (15 min, $\lambda = 220$ nm, MultoKrom® 100-5 C18-column (125 × 4.6 mm, 5 µm particle size, *CS Chromatographie GmbH*, $t_R = 11.8$ min).

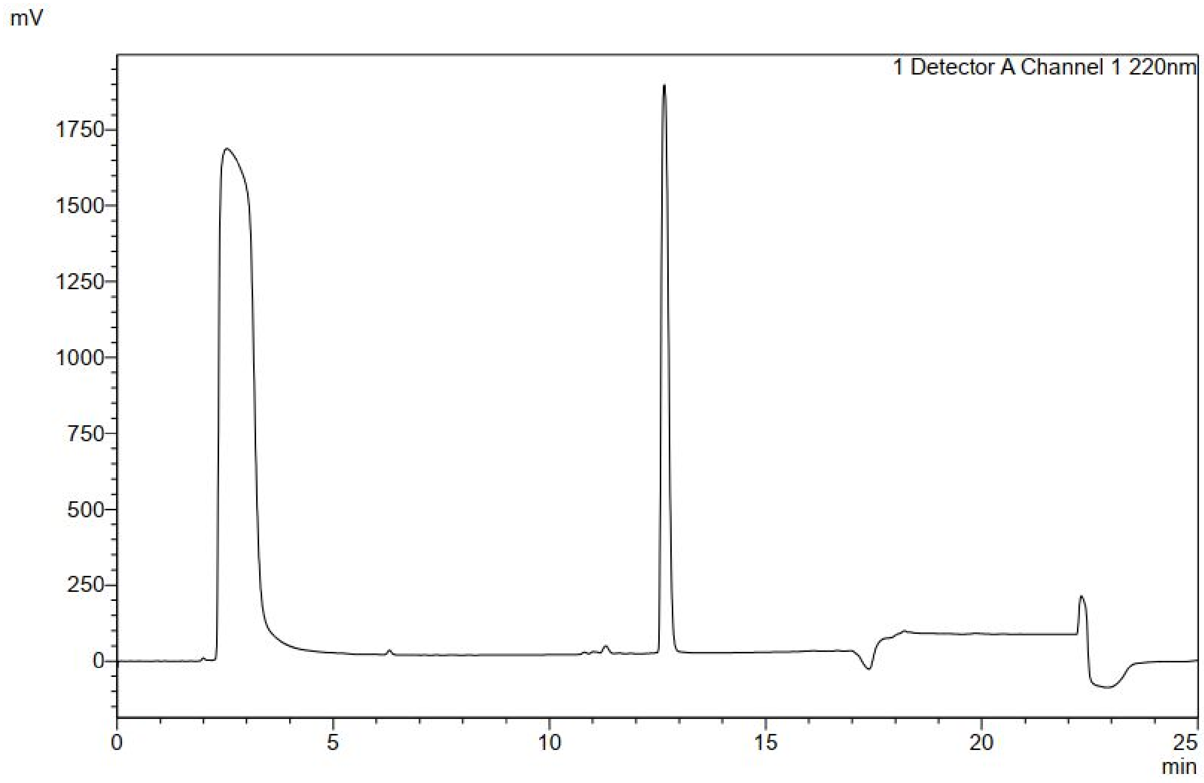

Figure S24: RP-HPLC chromatogram of the reaction control of H_2N-(SiFA)SeFe-Gly-Tyr-OH ($1X_3$) using the analytical control method 10-60% B (15 min, λ = 220 nm, MultoKrom® 100-5 C18-column (125 × 4.6 mm, 5 µm particle size, *CS Chromatographie GmbH*, t_R = 12.8 min).

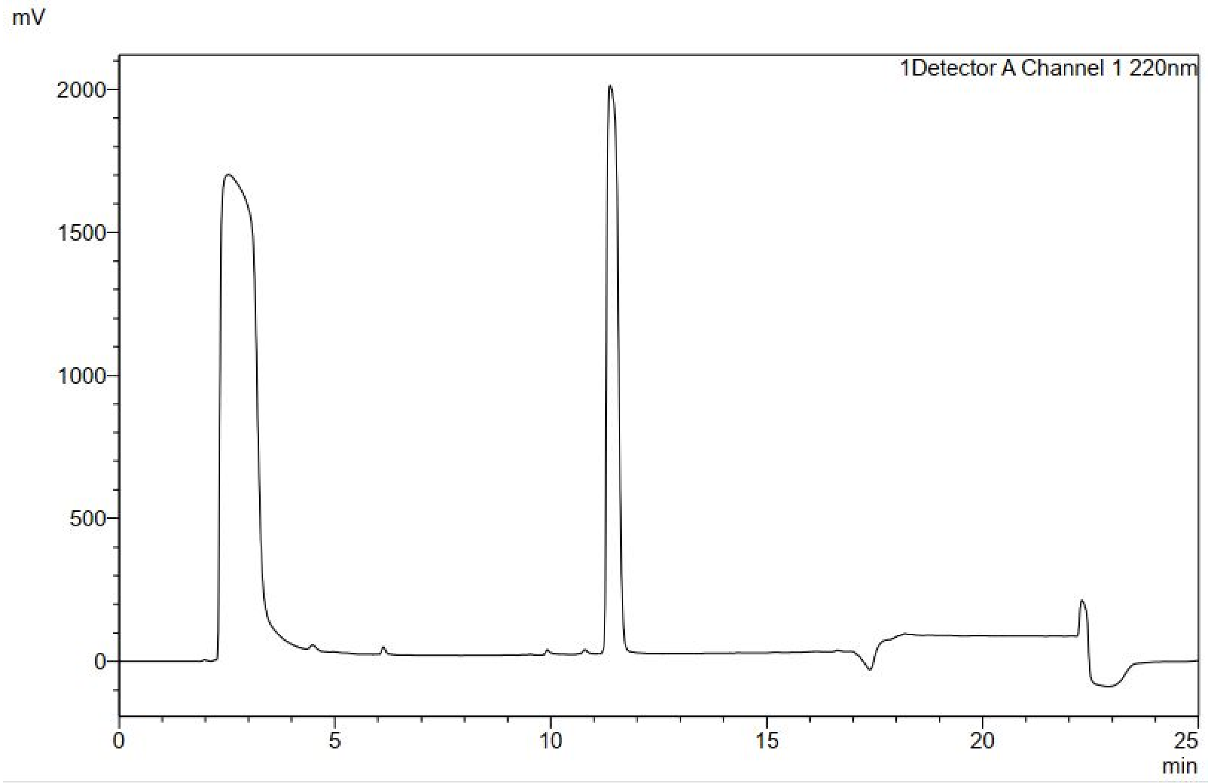

Figure S25: RP-HPLC chromatogram of the reaction control of H_2N-Lys-(SiFA)SeFe-Gly-Tyr-OH ($2X_3$) using the analytical control method 10-60% B (15 min, $\lambda = 220$ nm, MultoKrom® 100-5 C18-column (125 × 4.6 mm, 5 µm particle size, *CS Chromatographie GmbH*, $t_R = 11.4$ min).

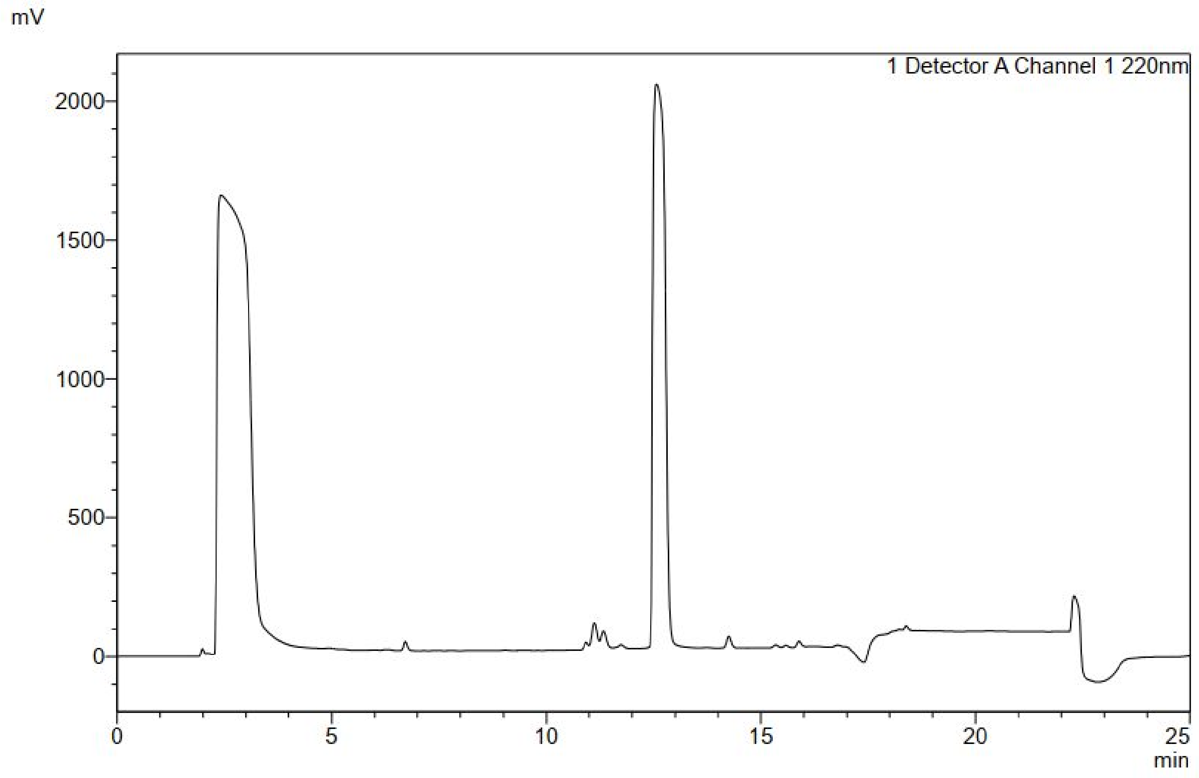

Figure S26: RP-HPLC chromatogram of the reaction control of H_2N-Glu-(SiFA)SeFe-Gly-Tyr-OH ($3X_3$) using the analytical control method 10-60% B (15 min, $\lambda = 220$ nm, MultoKrom® 100-5 C18-column (125 × 4.6 mm, 5 µm particle size, *CS Chromatographie GmbH*, $t_R = 12.6$ min).

2.2. Mass Spectrometry

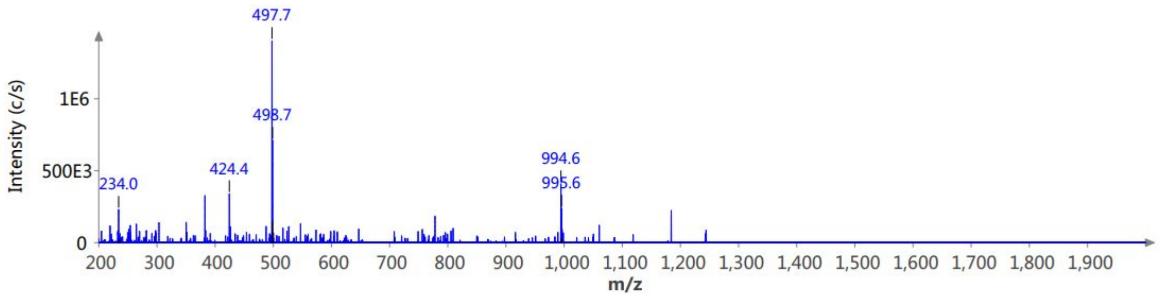

Figure S27: ESI+-mass spectrum of H_2N-(SiFA)SeFe-Gly-Lys-OH ($1X_1$).

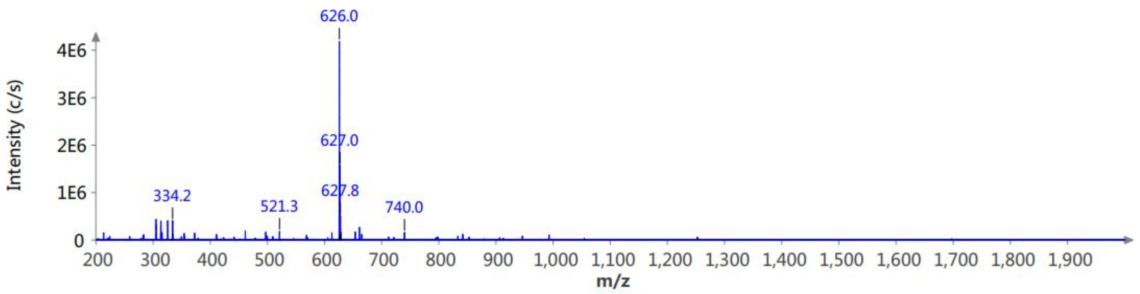

Figure S28: ESI$^+$-mass spectrum of H_2N-Lys-(SiFA)SeFe-Gly-Lys-OH ($2X_1$).

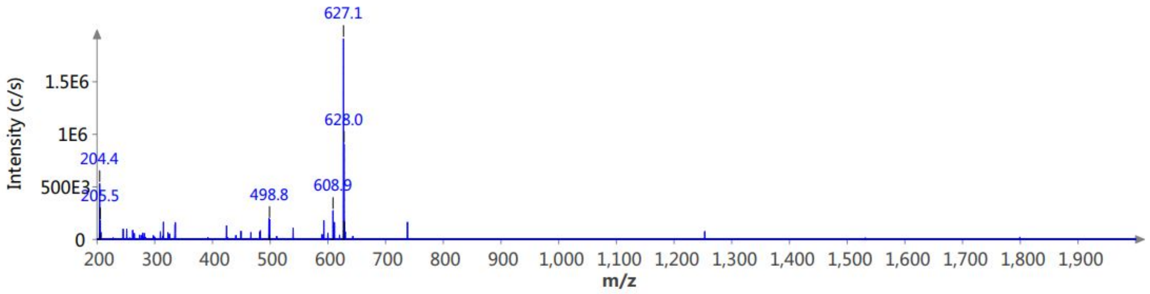

Figure S29: ESI$^+$-mass spectrum of H_2N-Glu-(SiFA)SeFe-Gly-Lys-OH ($3X_1$).

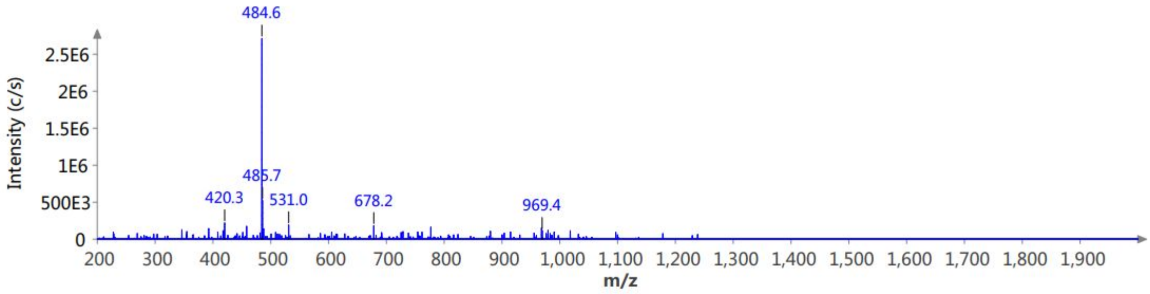

Figure S30: ESI$^+$-mass spectrum of H_2N-(SiFA)SeFe-Gly-Asp-OH ($1X_2$).

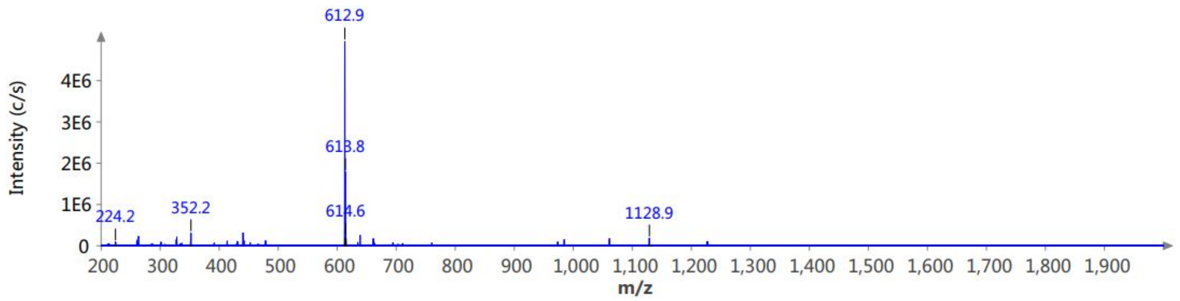

Figure S31: ESI^{+}-mass spectrum of H_2N-Lys-(SiFA)SeFe-Gly-Asp-OH ($2X_2$).

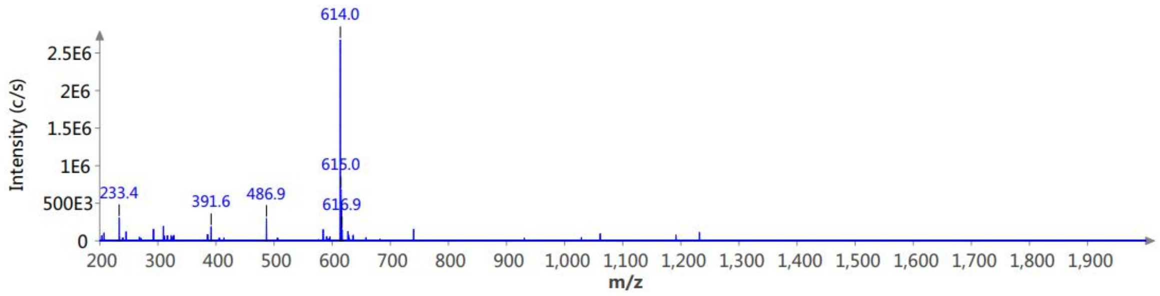

Figure S32: ESI^{+}-mass spectrum of H_2N-Glu-(SiFA)SeFe-Gly-Asp-OH ($3X_2$).

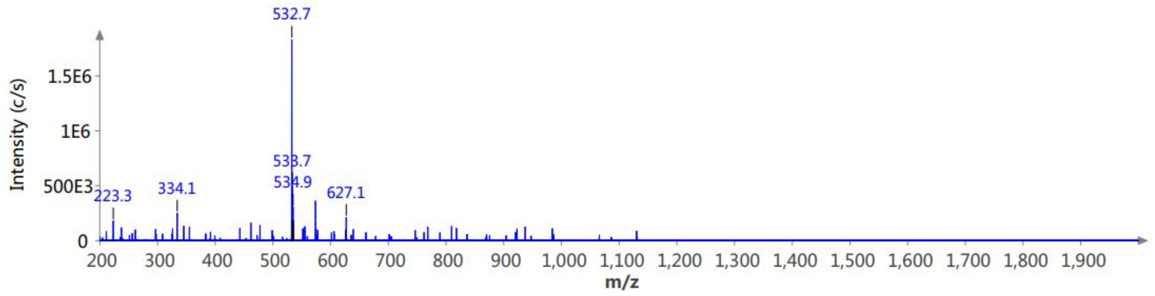

Figure S33: ESI^{+}-mass spectrum of H_2N-(SiFA)SeFe-Gly-Tyr-OH ($1X_3$).

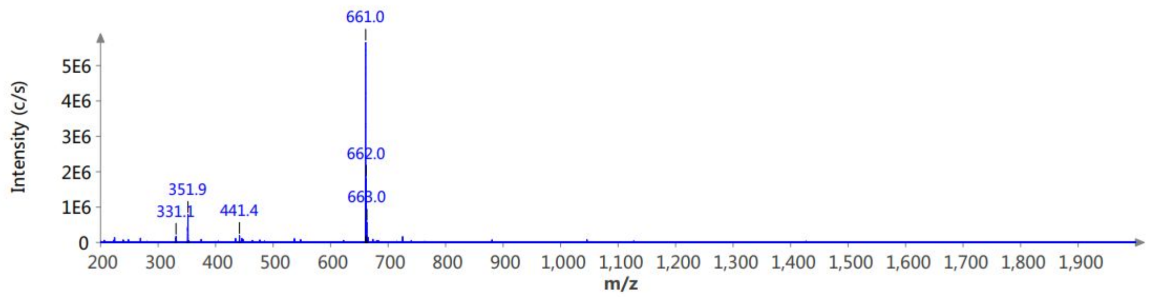

Figure S34: ESI$^+$-mass spectrum of H_2N-Lys-(SiFA)SeFe-Gly-Tyr-OH ($2X_3$).

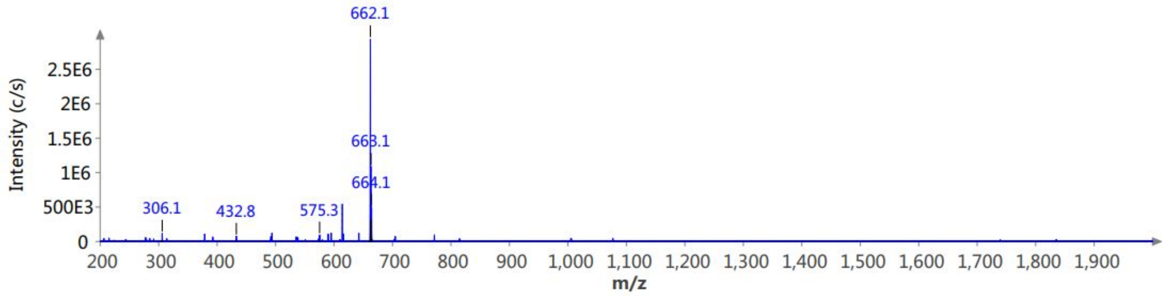

Figure S35: ESI$^+$-mass spectrum of H_2N-Glu-(SiFA)SeFe-Gly-Tyr-OH ($3X_3$).

2.3. Radiochemical characterization

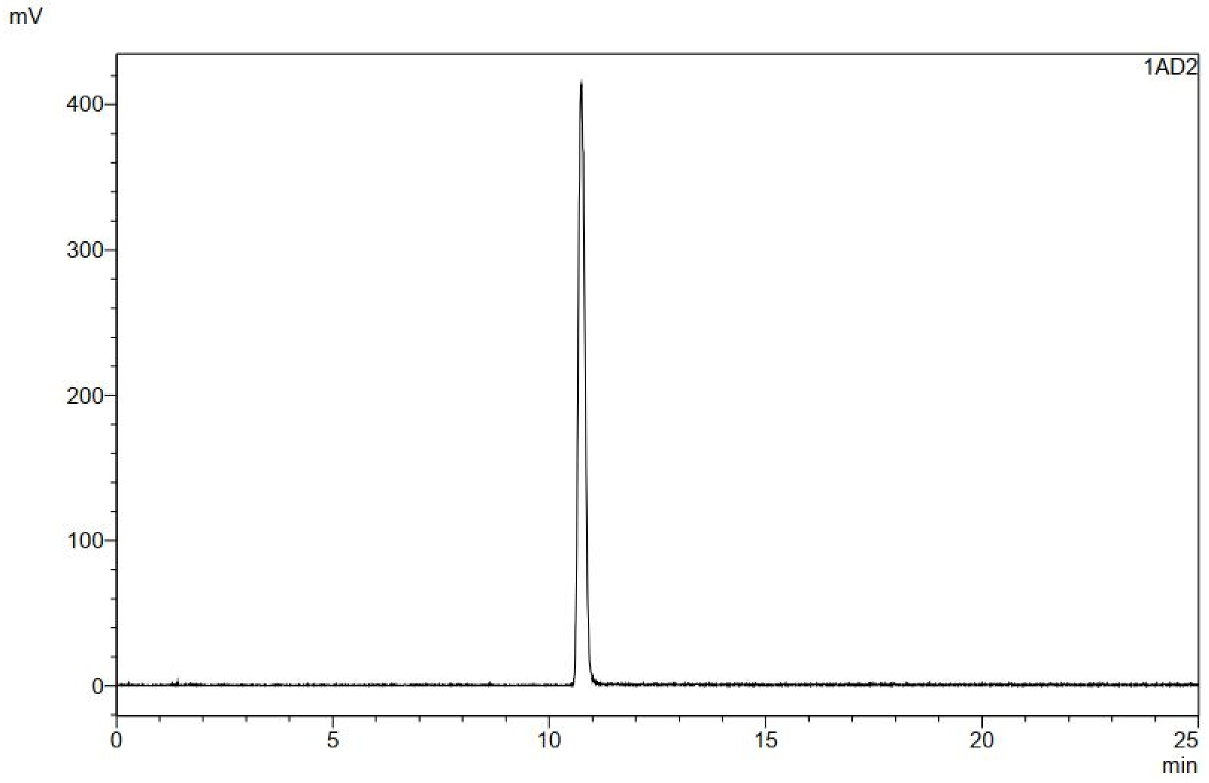

Figure S36: Radio-RP-HPLC chromatogram of [^{18}F]H_2N-(SiFA)SeFe-Gly-Lys-OH ($1X_1$) using the analytical control method 10-60% B (15 min, MultoKrom® 100-5 C18-column (125 × 4.6 mm, 5 µm particle size, CS Chromatographie GmbH), t_R = 10.7 min).

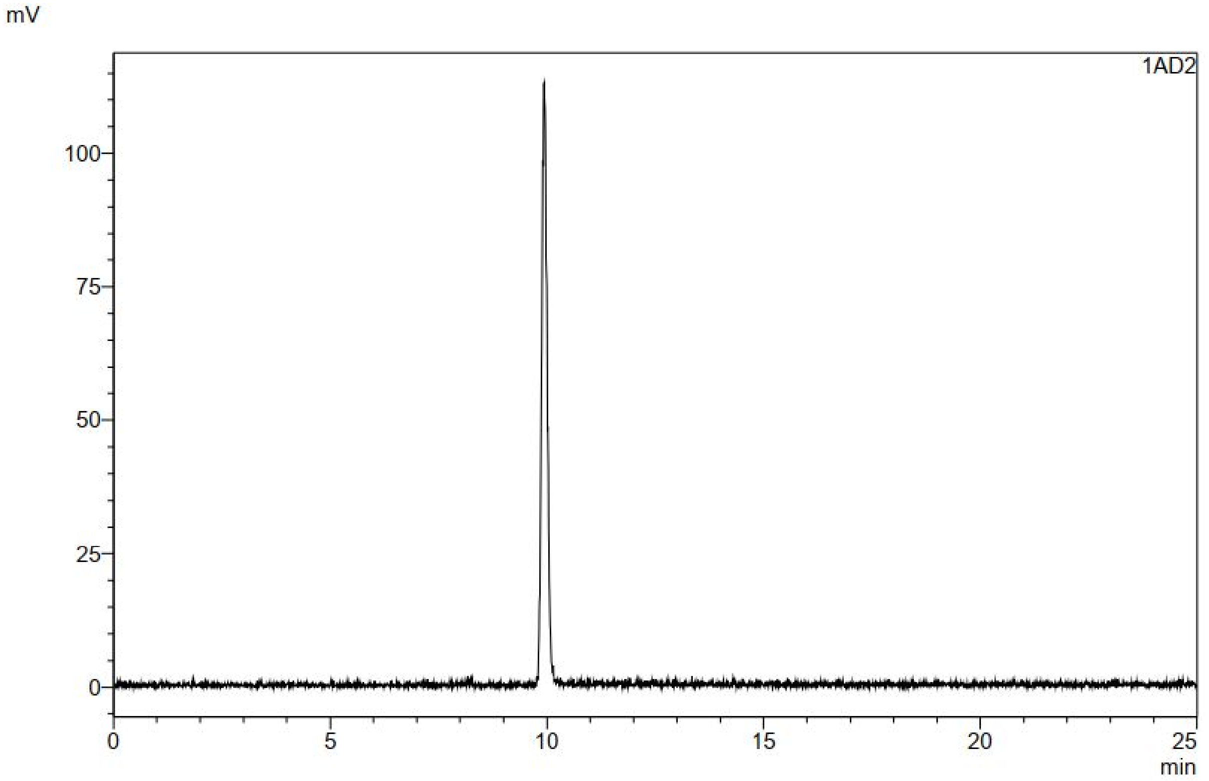

Figure S37: Radio-RP-HPLC chromatogram of [^{18}F]H_2N-Lys-(SiFA)SeFe-Gly-Lys-OH (2X_1) using the analytical control method 10-60% B (15 min, MultoKrom® 100-5 C18-column (125 × 4.6 mm, 5 µm particle size, CS Chromatographie GmbH), t_R = 9.9 min).

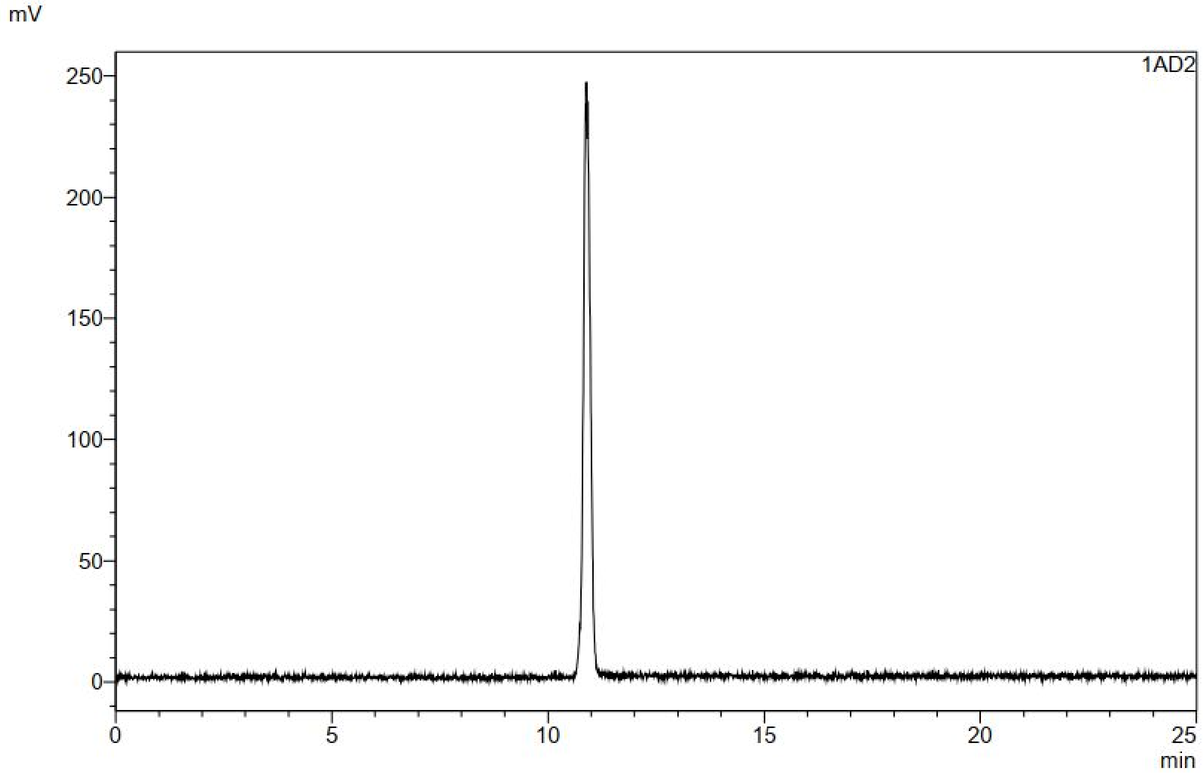

Figure S38: Radio-RP-HPLC chromatogram of [^{18}F]H_2N-Glu-(SiFA)SeFe-Gly-Lys-OH ($3X_1$) using the analytical control method 10-60% B (15 min, MultoKrom® 100-5 C18-column (125 × 4.6 mm, 5 µm particle size, CS Chromatographie GmbH), t_R = 10.9 min).

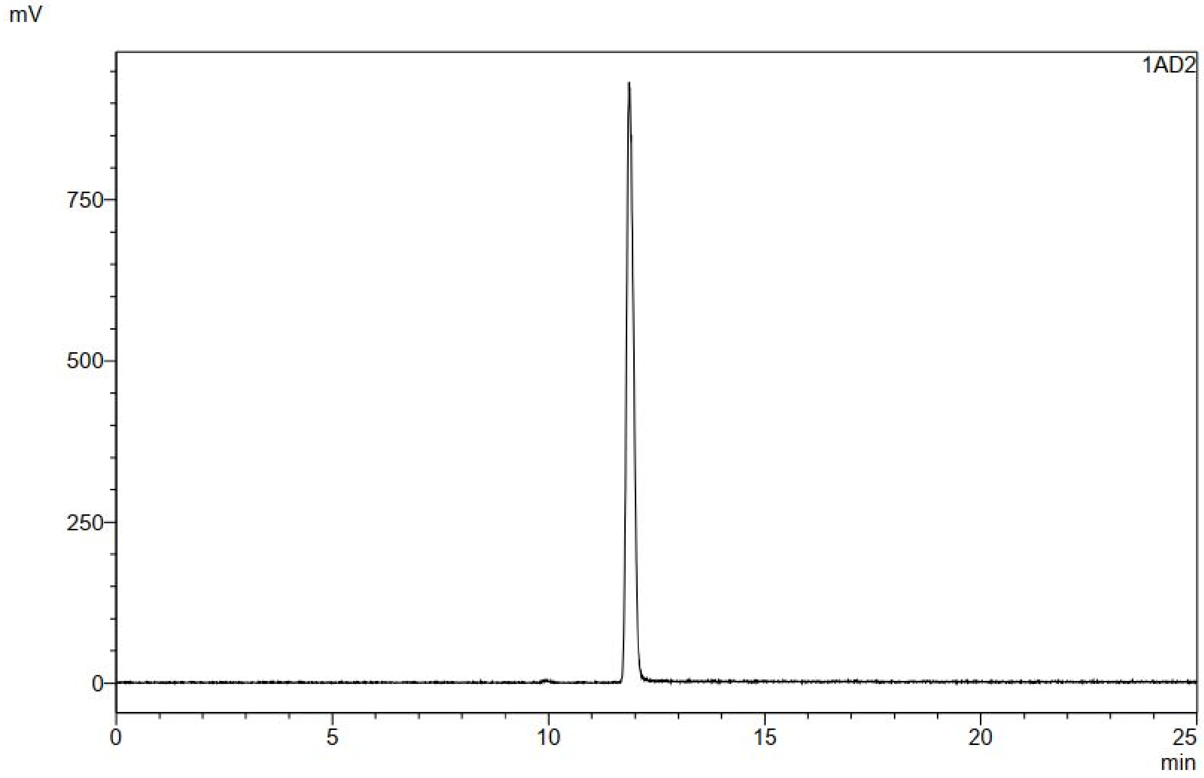

Figure S39: Radio-RP-HPLC chromatogram of [^{18}F]H_2N-(SiFA)SeFe-Gly-Asp-OH (1X_2) using the analytical control method 10-60% B (15 min, MultoKrom® 100-5 C18-column (125 × 4.6 mm, 5 µm particle size, CS Chromatographie GmbH), t_R = 11.9 min).

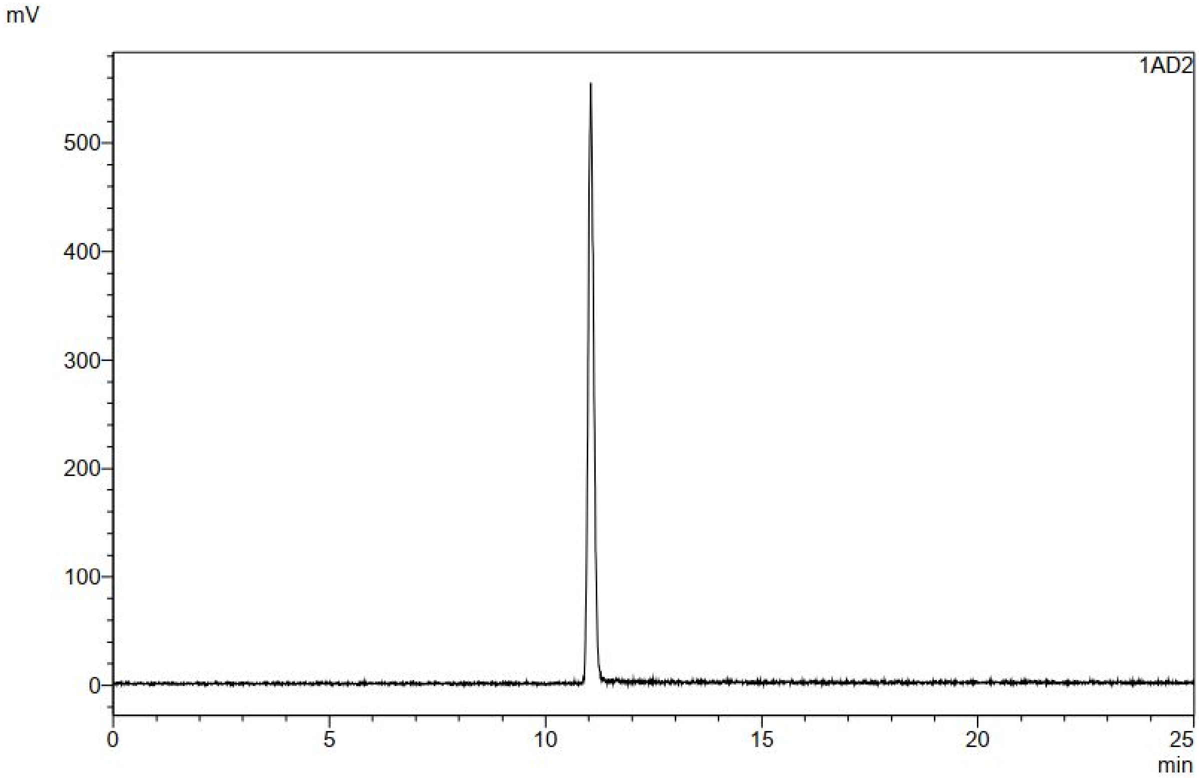

Figure S40: Radio-RP-HPLC chromatogram of [^{18}F]H_2N-Lys-(SiFA)SeFe-Gly-Asp-OH (2X_2) using the analytical control method 10-60% B (15 min, MultoKrom® 100-5 C18-column (125 × 4.6 mm, 5 µm particle size, CS Chromatographie GmbH), t_R = 11.0 min).

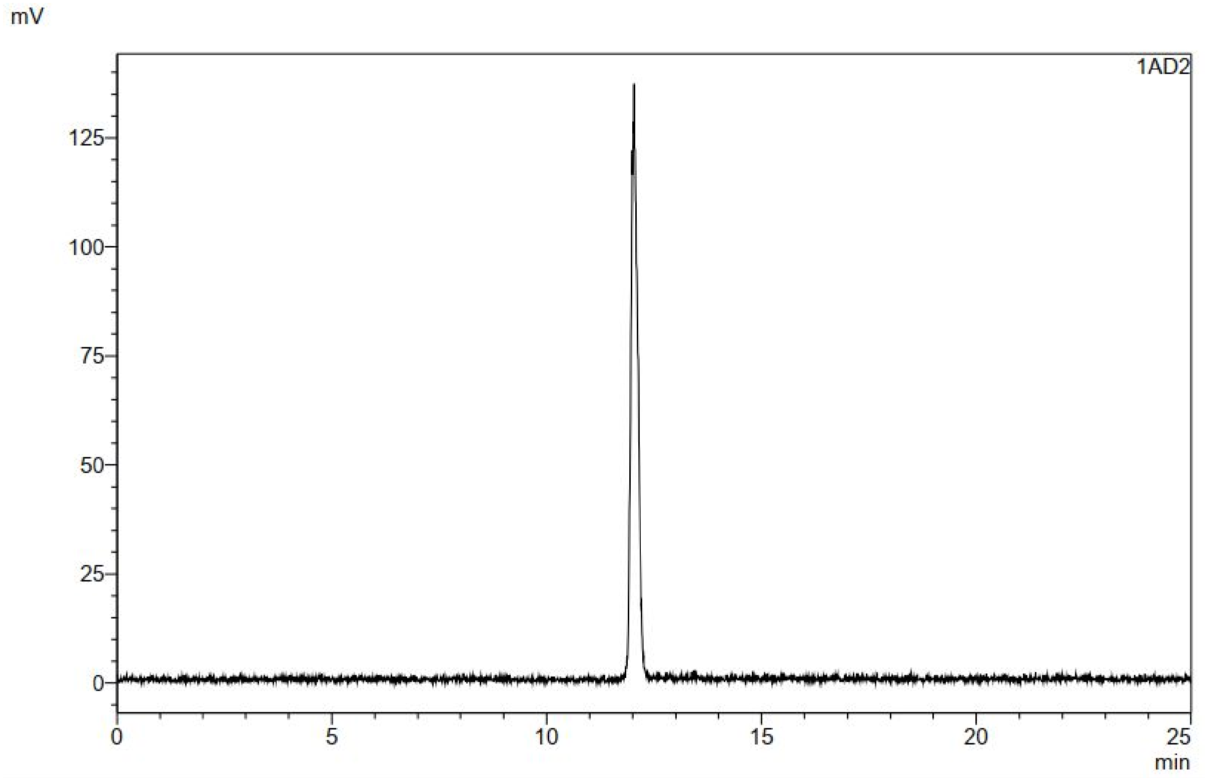

Figure S41: Radio-RP-HPLC chromatogram of [^{18}F]H_2N-Glu-(SiFA)SeFe-Gly-Asp-OH (3X_2) using the analytical control method 10-60% B (15 min, MultoKrom® 100-5 C18-column (125 × 4.6 mm, 5 µm particle size, CS Chromatographie GmbH), t_R = 12.0 min).

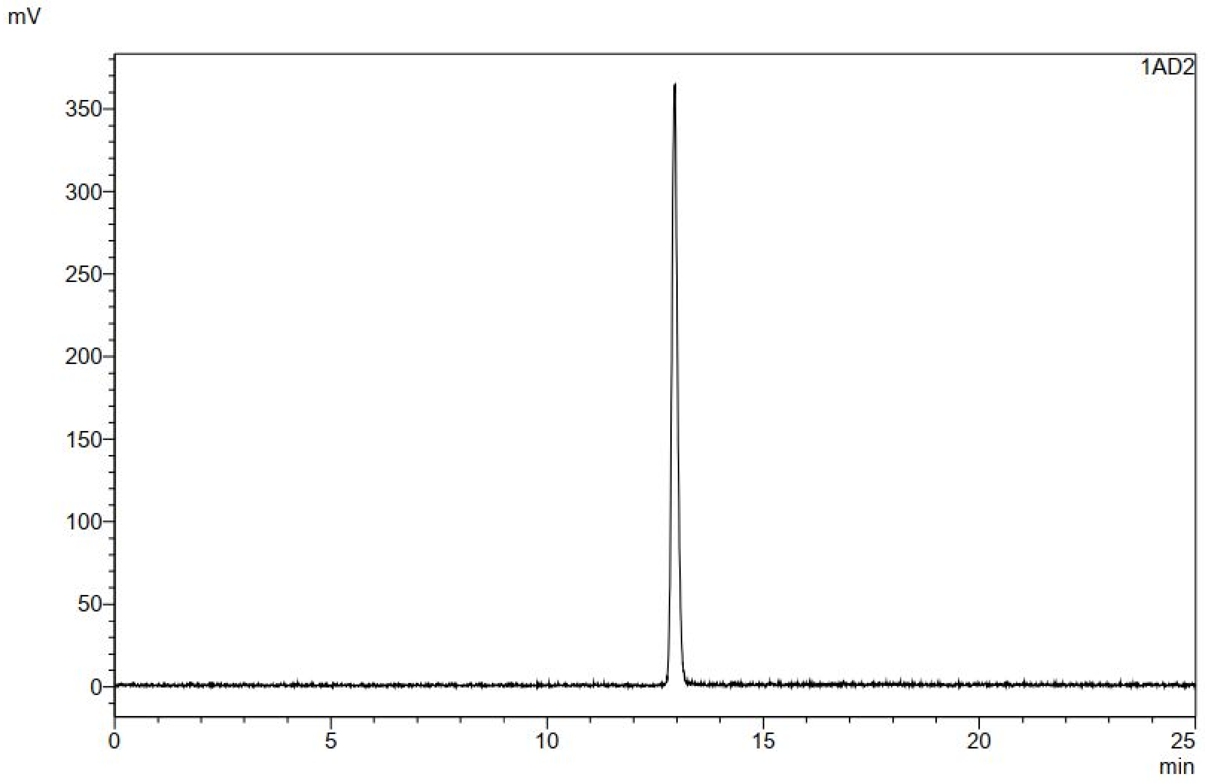

Figure S42: Radio-RP-HPLC chromatogram of [^{18}F]H_2N-(SiFA)SeFe-Gly-Tyr-OH (1X_3) using the analytical control method 10-60% B (15 min, MultoKrom® 100-5 C18-column (125 × 4.6 mm, 5 µm particle size, CS Chromatographie GmbH), t_R = 13.0 min).

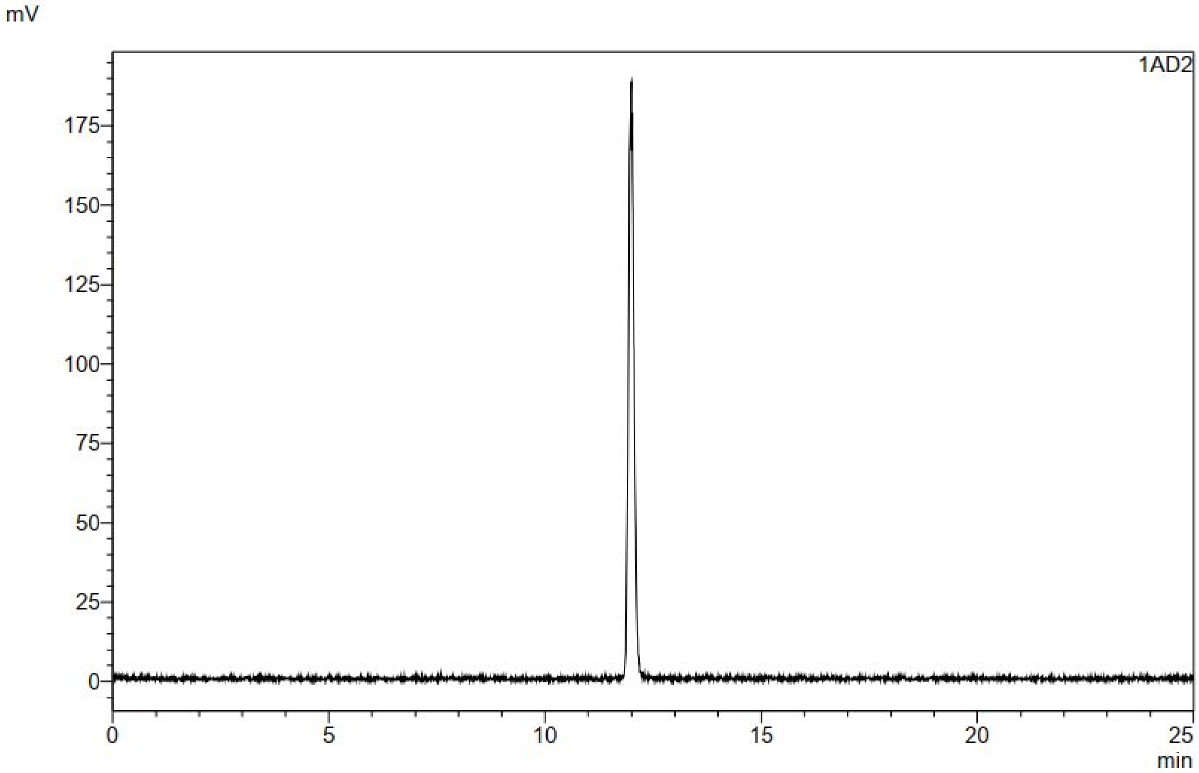

Figure S43: Radio-RP-HPLC chromatogram of [^{18}F]H_2N-Lys-(SiFA)SeFe-Gly-Tyr-OH (2X_3) using the analytical control method 10-60% B (15 min, MultoKrom® 100-5 C18-column (125 × 4.6 mm, 5 µm particle size, CS Chromatographie GmbH), t_R = 12.0 min).

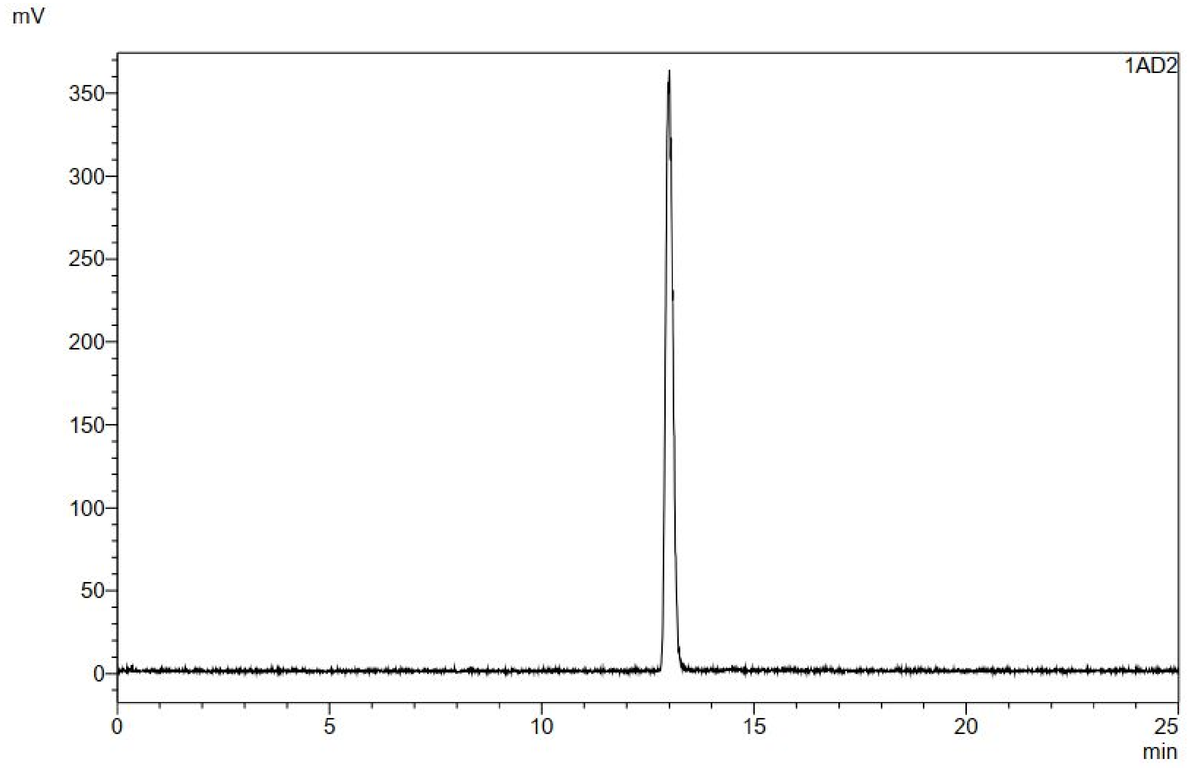

Figure S44: Radio-RP-HPLC chromatogram of [^{18}F]H_2N-Glu-(SiFA)SeFe-Gly-Tyr-OH (3X_3) using the analytical control method 10-60% B (15 min, MultoKrom® 100-5 C18-column (125 × 4.6 mm, 5 µm particle size, CS Chromatographie GmbH), t_R = 13.0 min).

2.4. Radiochemical conversion

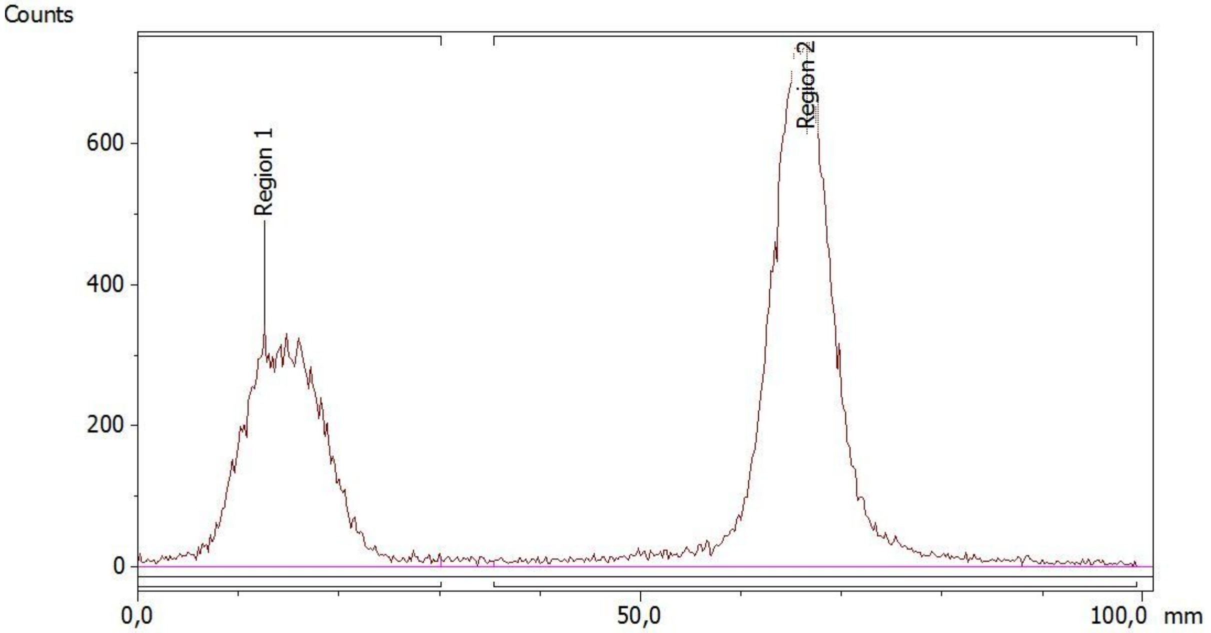

Figure S45: Radio-TLC chromatogram of [^{18}F]H_2N-(SiFA)SeFe-Gly-Lys-OH ($1X_1$) before purification for determination of the RCC (flow agent: 60% MeCN/ 40% PBS (6/4 *v*/*v*) with 10% NaOAc in H_2O (2 M) and 1% TFA, stationary phase: TLC Silica gel 60 F254 from *Merck Millipore*).

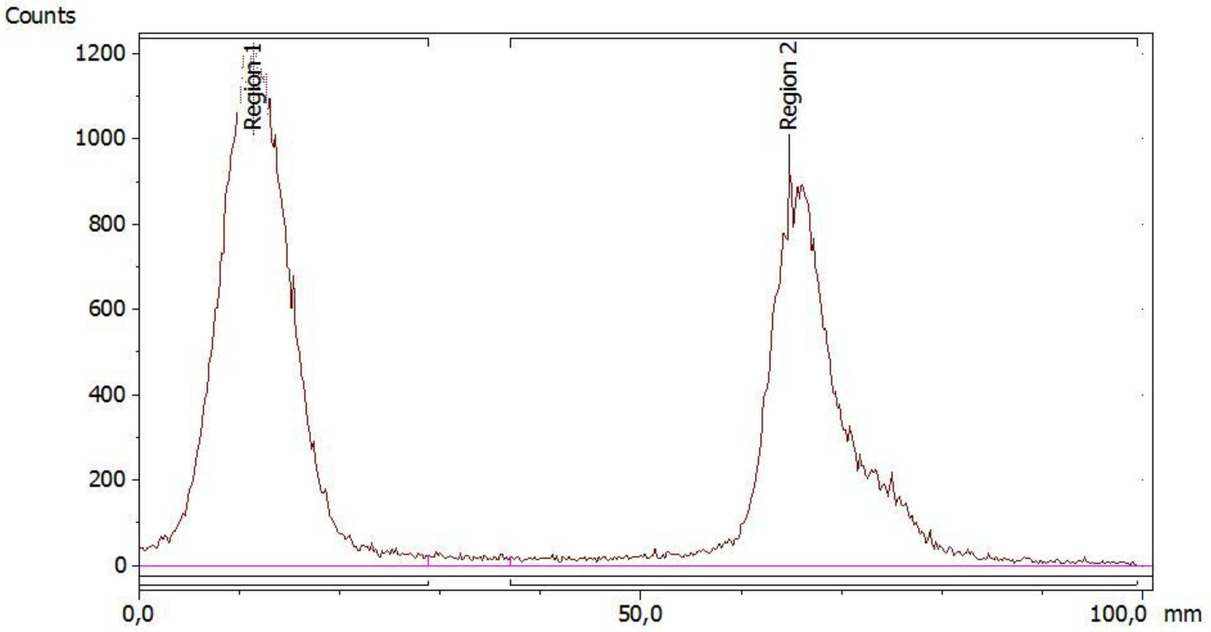

Figure S46: Radio-TLC chromatogram of [^{18}F]H_2N-Lys-(SiFA)SeFe-Gly-Lys-OH ($2X_1$) before purification for determination of the RCC (flow agent: 60% MeCN/ 40% PBS (6/4 *v*/*v*) with 10% NaOAc in H_2O (2 M) and 1% TFA, stationary phase: TLC Silica gel 60 F254 from *Merck Millipore*).

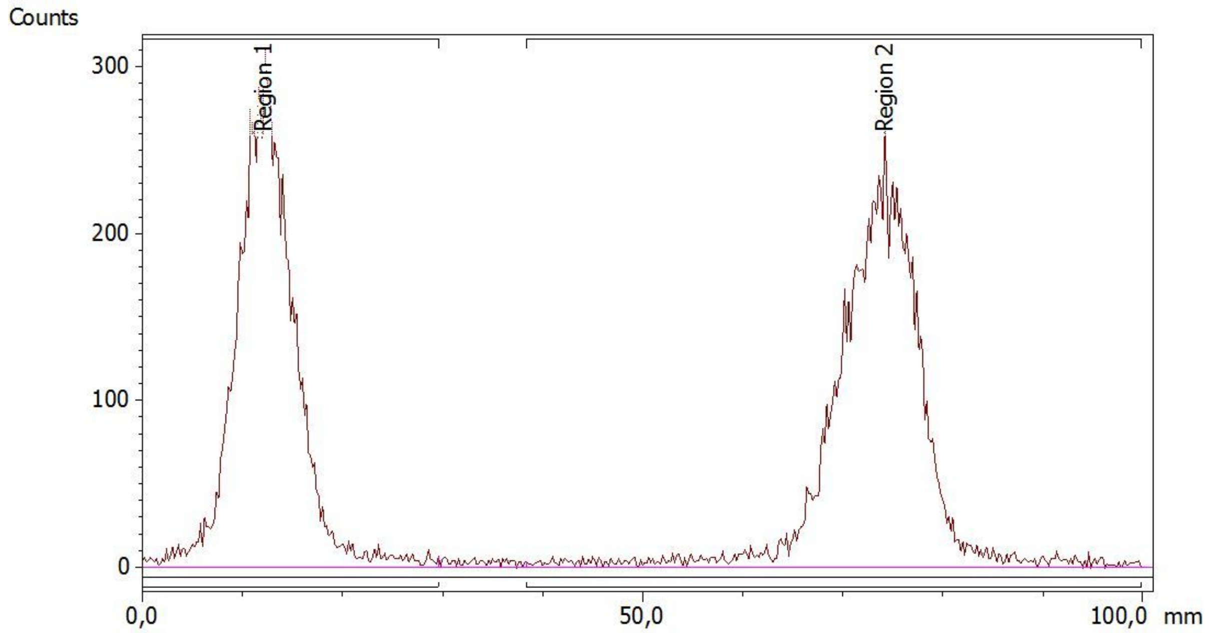

Figure S47: Radio-TLC chromatogram of [^{18}F]H_2N-Glu-(SiFA)SeFe-Gly-Lys-OH ($3X_1$) before purification for determination of the RCC (flow agent: 60% MeCN/ 40% PBS (6/4 *v*/*v*) with 10% NaOAc in H_2O (2 M) and 1% TFA, stationary phase: TLC Silica gel 60 F254 from *Merck Millipore*).

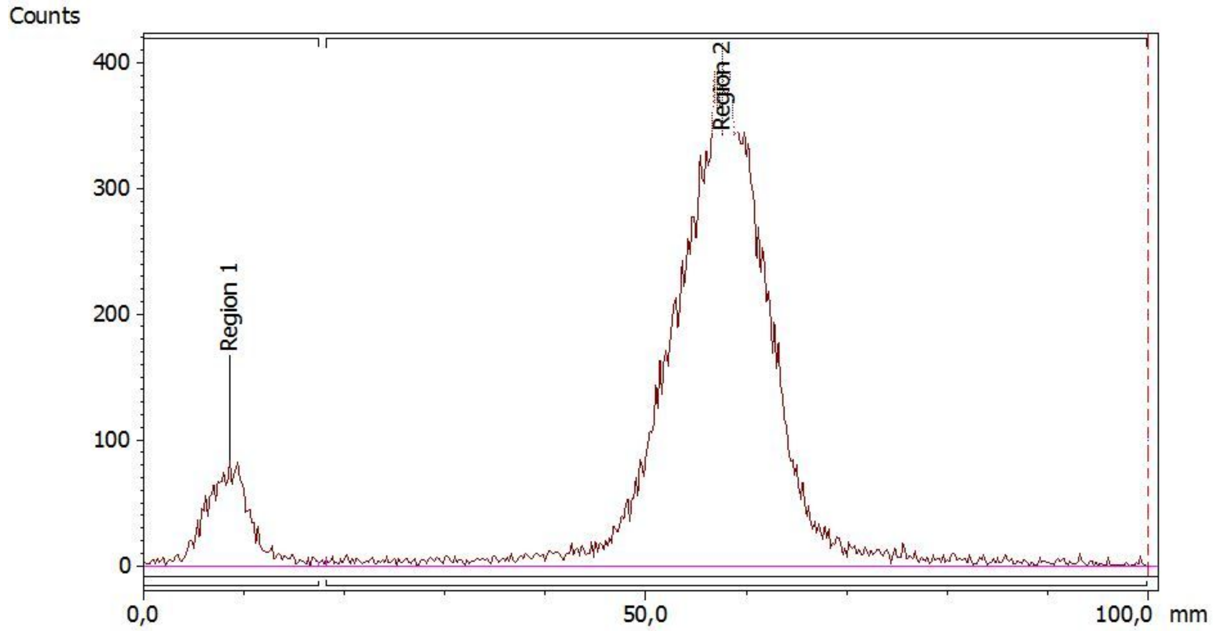

Figure S48: Radio-TLC chromatogram of [^{18}F]H_2N-(SiFA)SeFe-Gly-Asp-OH ($1X_2$) before purification for determination of the RCC (flow agent: 60% MeCN/ 40% PBS (6/4 *v*/*v*) with 10% NaOAc in H_2O (2 M) and 1% TFA, stationary phase: TLC Silica gel 60 F254 from *Merck Millipore*).

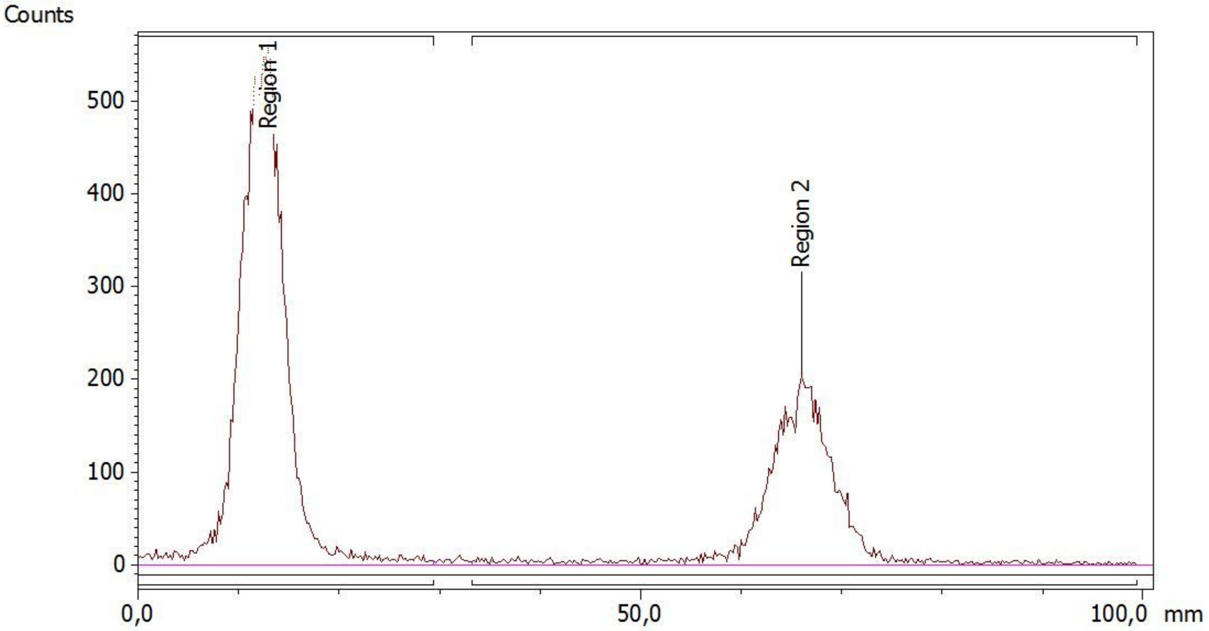

Figure S49: Radio-TLC chromatogram of [^{18}F]H_2N-Lys-(SiFA)SeFe-Gly-Asp-OH (2X2) before purification for determination of the RCC (flow agent: 60% MeCN/ 40% PBS (6/4 *v*/*v*) with 10% NaOAc in H_2O (2 M) and 1% TFA, stationary phase: TLC Silica gel 60 F254 from *Merck Millipore*).

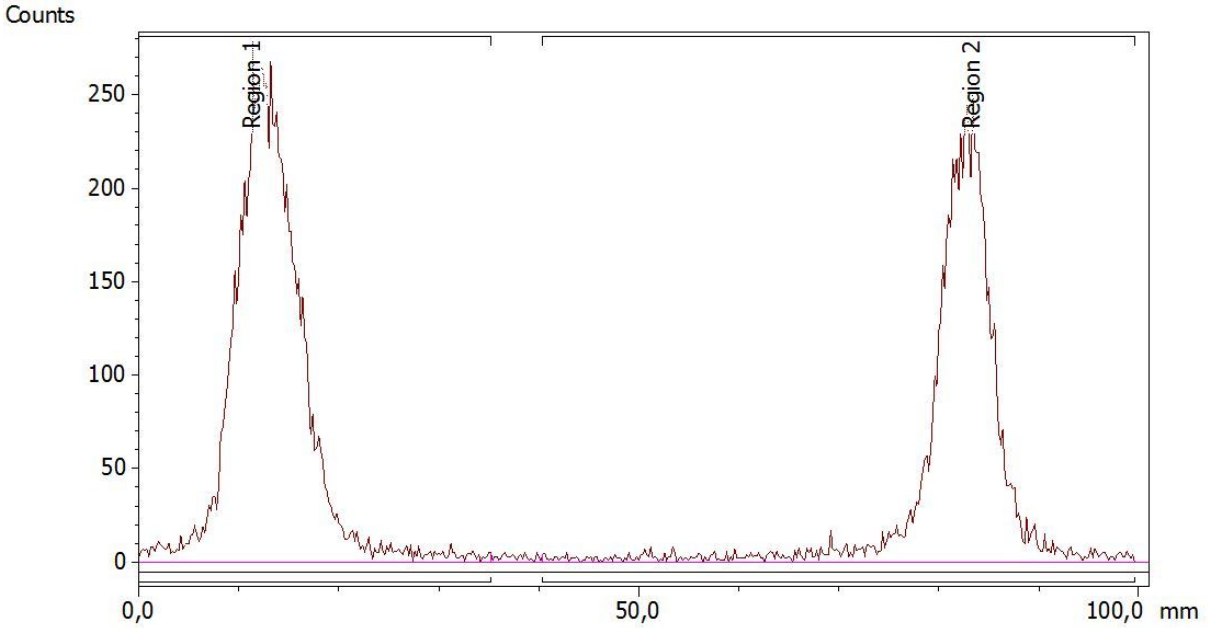

Figure S50: Radio-TLC chromatogram of [^{18}F]H_2N-Glu-(SiFA)SeFe-Gly-Asp-OH ($3X_2$) before purification for determination of the RCC (flow agent: 60% MeCN/ 40% PBS (6/4 *v*/*v*) with 10% NaOAc in H_2O (2 M) and 1% TFA, stationary phase: TLC Silica gel 60 F254 from *Merck Millipore*).

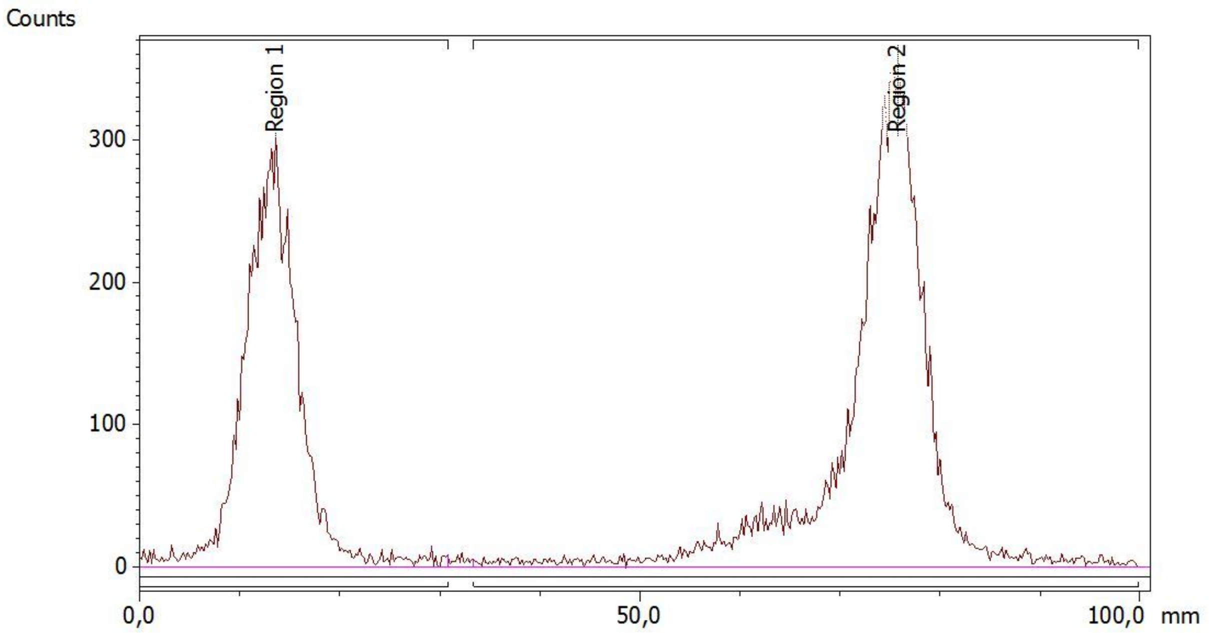

Figure S51*:* Radio-TLC chromatogram of [^{18}F]H_2N-(SiFA)SeFe-Gly-Tyr-OH (1X_3) before purification for determination of the RCC (flow agent: 60% MeCN/ 40% PBS (6/4 *v*/*v*) with 10% NaOAc in H_2O (2 M) and 1% TFA, stationary phase: TLC Silica gel 60 F254 from *Merck Millipore*).

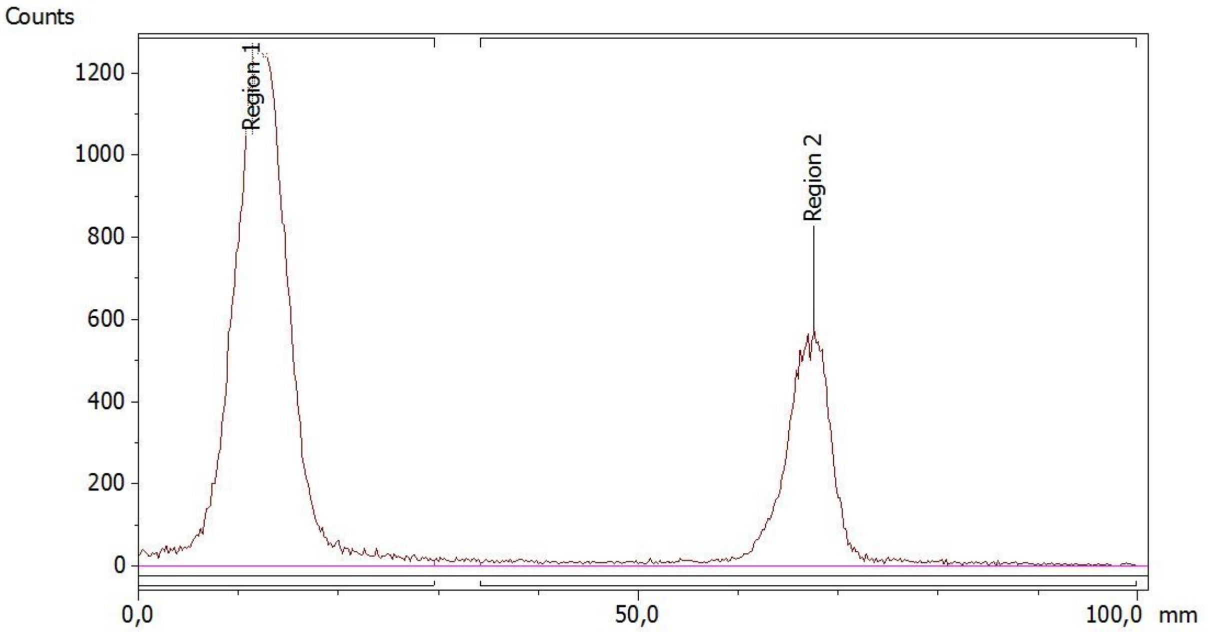

Figure S52*:* Radio-TLC chromatogram of [^{18}F]H_2N-Lys-(SiFA)SeFe-Gly-Tyr-OH (2X_3) before purification for determination of the RCC (flow agent: 60% MeCN/ 40% PBS (6/4 *v*/*v*) with 10% NaOAc in H_2O (2 M) and 1% TFA, stationary phase: TLC Silica gel 60 F254 from *Merck Millipore*).

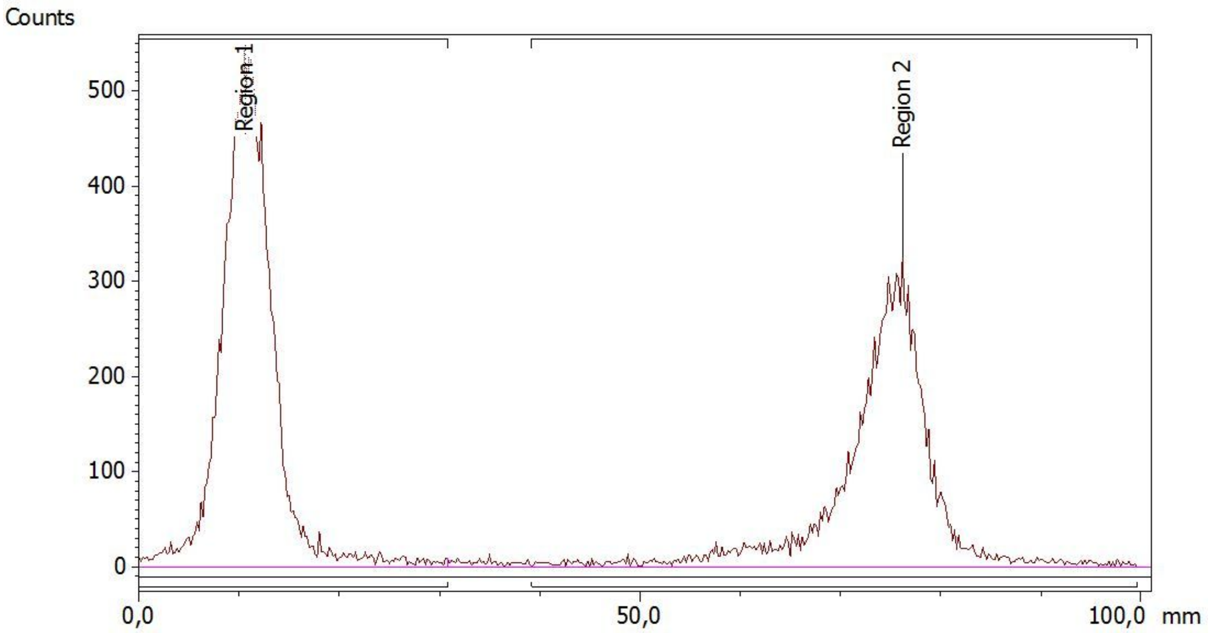

Figure S53*:* Radio-TLC chromatogram of [^{18}F]H_2N-Glu-(SiFA)SeFe-Gly-Tyr-OH ($3X_3$) before purification for determination of the RCC (flow agent: 60% MeCN/ 40% PBS (6/4 *v*/*v*) with 10% NaOAc in H_2O (2 M) and 1% TFA, stationary phase: TLC Silica gel 60 F254 from *Merck Millipore*).

2.5. Stability under lutetium labeling conditions

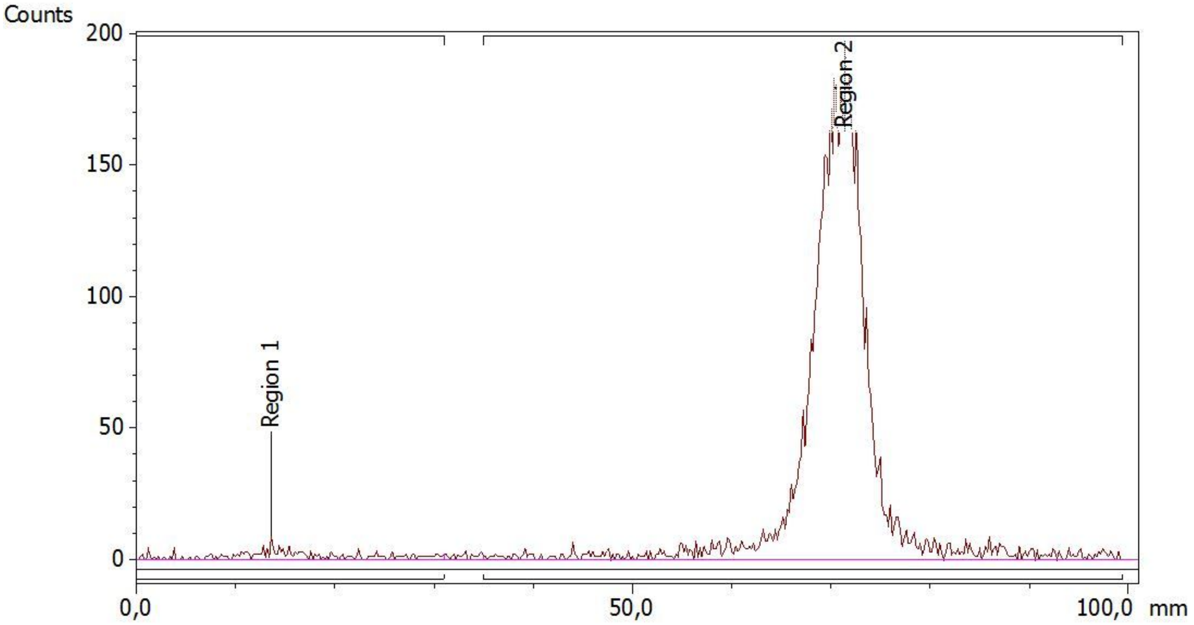

Figure S54: Radio-TLC chromatogram of [^{18}F]H_2N-(SiFA)SeFe-Gly-Lys-OH (1X_1) at pH 5.5 and 90 °C, t = 0 min (flow agent: 60% MeCN/ 40% PBS (6/4 v/v) with 10% NaOAc in H_2O (2 M) and 1% TFA, stationary phase: TLC Silica gel 60 F254 from Merck Millipore).

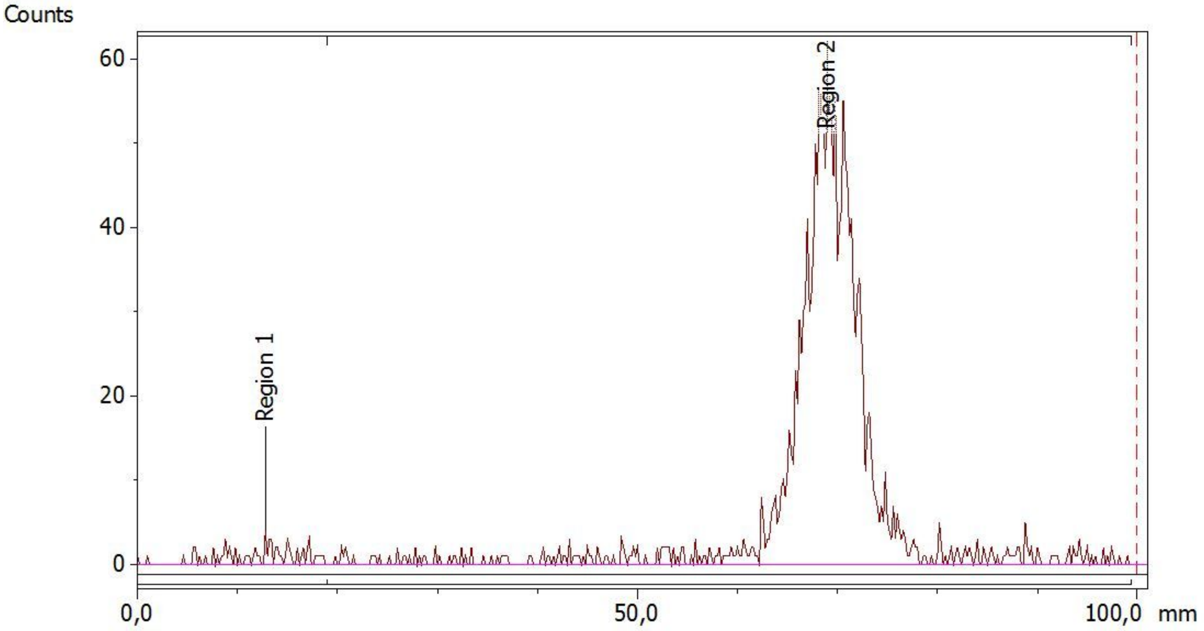

Figure S55: Radio-TLC chromatogram of [^{18}F]H_2N-(SiFA)SeFe-Gly-Lys-OH (1X_1) at pH 5.5 and

90 °C, t = 30 min (flow agent: 60% MeCN/ 40% PBS (6/4 v/v) with 10% NaOAc in H_2O (2 M) and 1% TFA, stationary phase: TLC Silica gel 60 F254 from Merck Millipore).

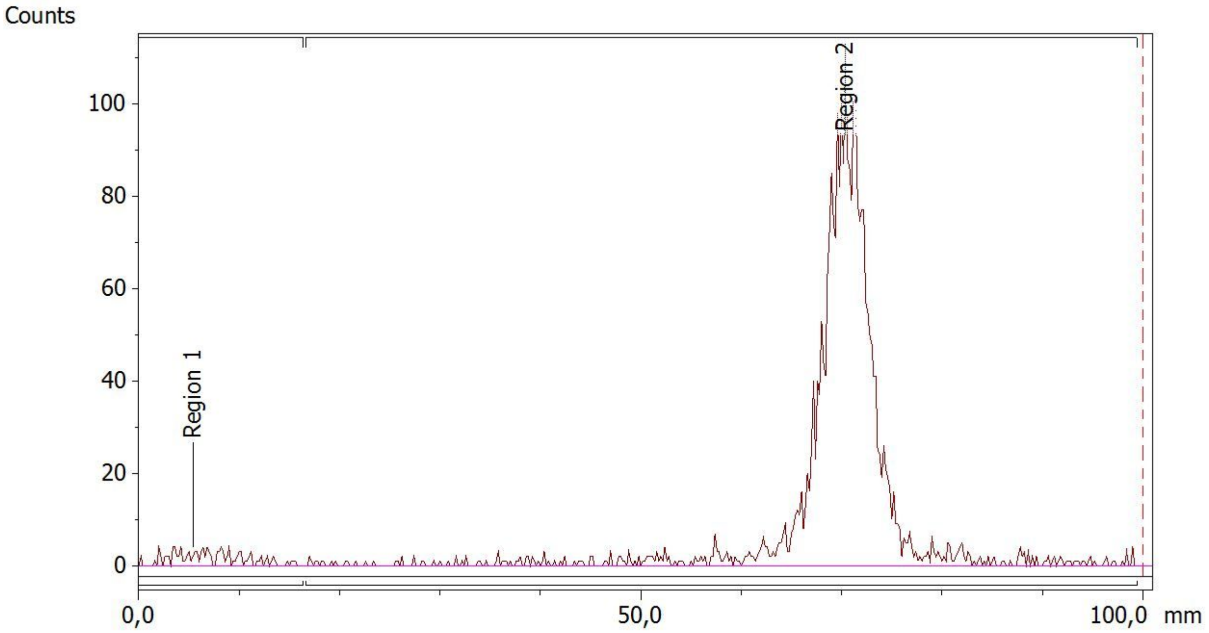

Figure S56: Radio-TLC chromatogram of [^{18}F]H_2N-(SiFA)SeFe-Gly-Lys-OH ($1X_1$) at pH 5.5 and 90 °C, t = 60 min (flow agent: 60% MeCN/ 40% PBS (6/4 v/v) with 10% NaOAc in H_2O (2 M) and 1% TFA, stationary phase: TLC Silica gel 60 F254 from Merck Millipore).

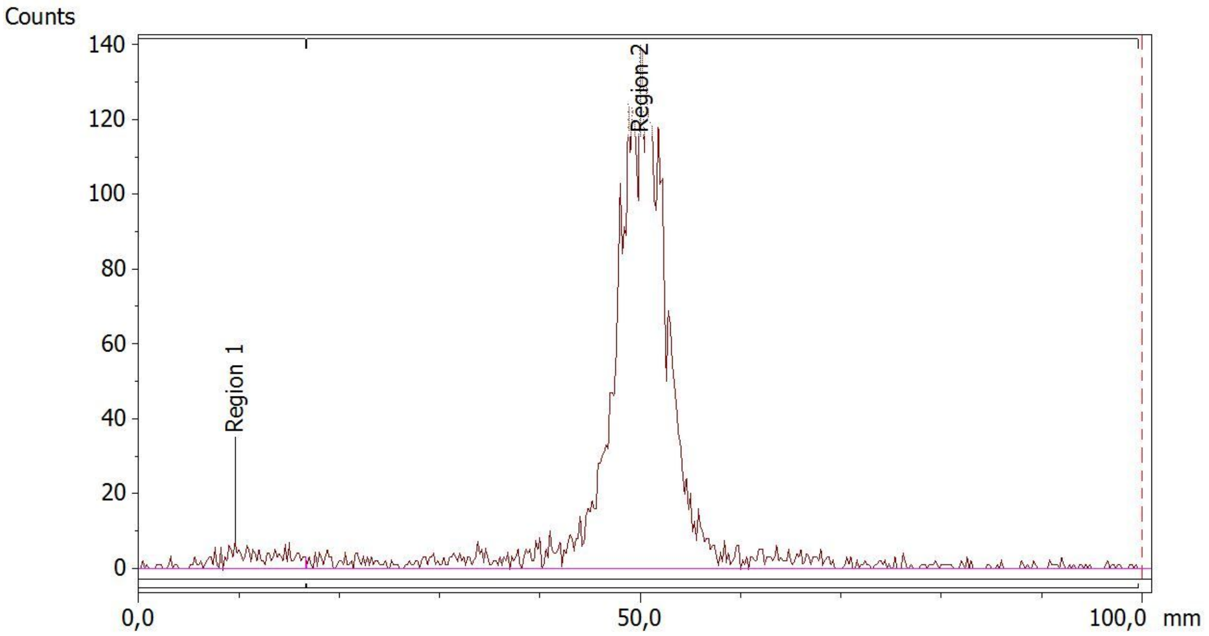

Figure S57: Radio-TLC chromatogram of [^{18}F]H_2N-(SiFA)SeFe-Gly-Lys-OH ($1X_1$) at pH 5.5 and

90 °C, t = 90 min (flow agent: 60% MeCN/ 40% PBS (6/4 v/v) with 10% NaOAc in H_2O (2 M) and 1% TFA, stationary phase: TLC Silica gel 60 F254 from Merck Millipore).

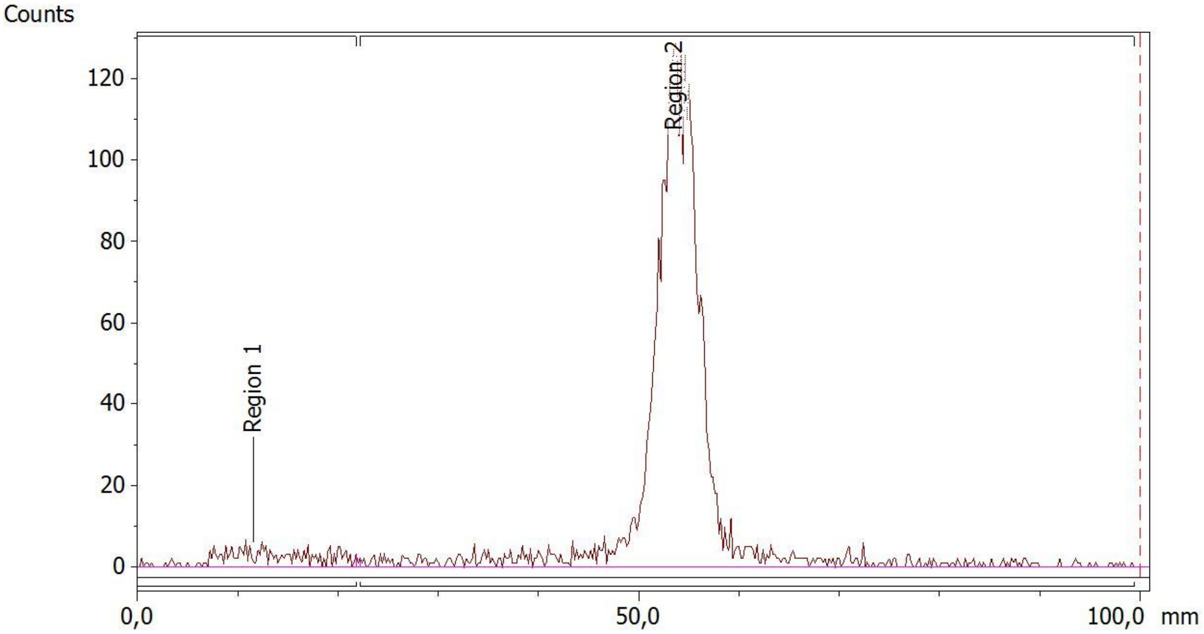

Figure S58: Radio-TLC chromatogram of [^{18}F]H_2N-(SiFA)SeFe-Gly-Lys-OH ($1X_1$) at pH 5.5 and 90 °C, t = 120 min (flow agent: 60% MeCN/ 40% PBS (6/4 v/v) with 10% NaOAc in H_2O (2 M) and 1% TFA, stationary phase: TLC Silica gel 60 F254 from Merck Millipore).

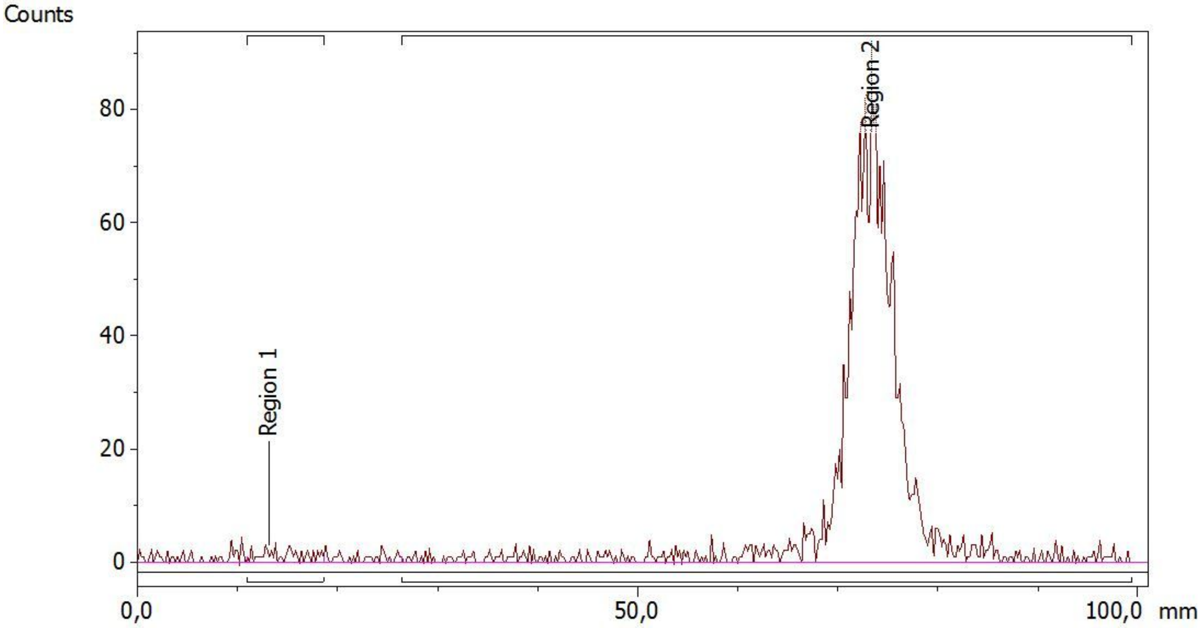

Figure S59: Radio-TLC chromatogram of [^{18}F]H_2N-Lys-(SiFA)SeFe-Gly-Lys-OH ($2X_1$) at pH 5.5 and

90°C, t = 0 min (flow agent: 60% MeCN/ 40% PBS (6/4 v/v) with 10% NaOAc in H_2O (2 M) and 1% TFA, stationary phase: TLC Silica gel 60 F254 from Merck Millipore).

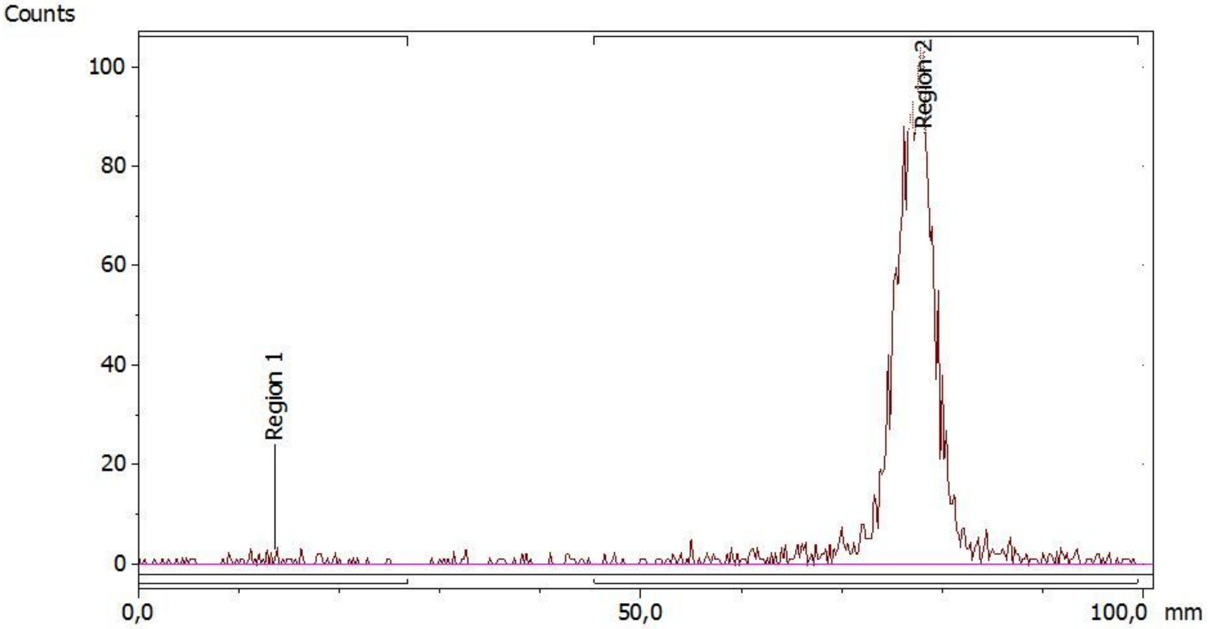

Figure S60: Radio-TLC chromatogram of [^{18}F]H_2N-Lys-(SiFA)SeFe-Gly-Lys-OH ($2X_1$) at pH 5.5 and 90 °C, t = 30 min (flow agent: 60% MeCN/ 40% PBS (6/4 v/v) with 10% NaOAc in H_2O (2 M) and 1% TFA, stationary phase: TLC Silica gel 60 F254 from Merck Millipore).

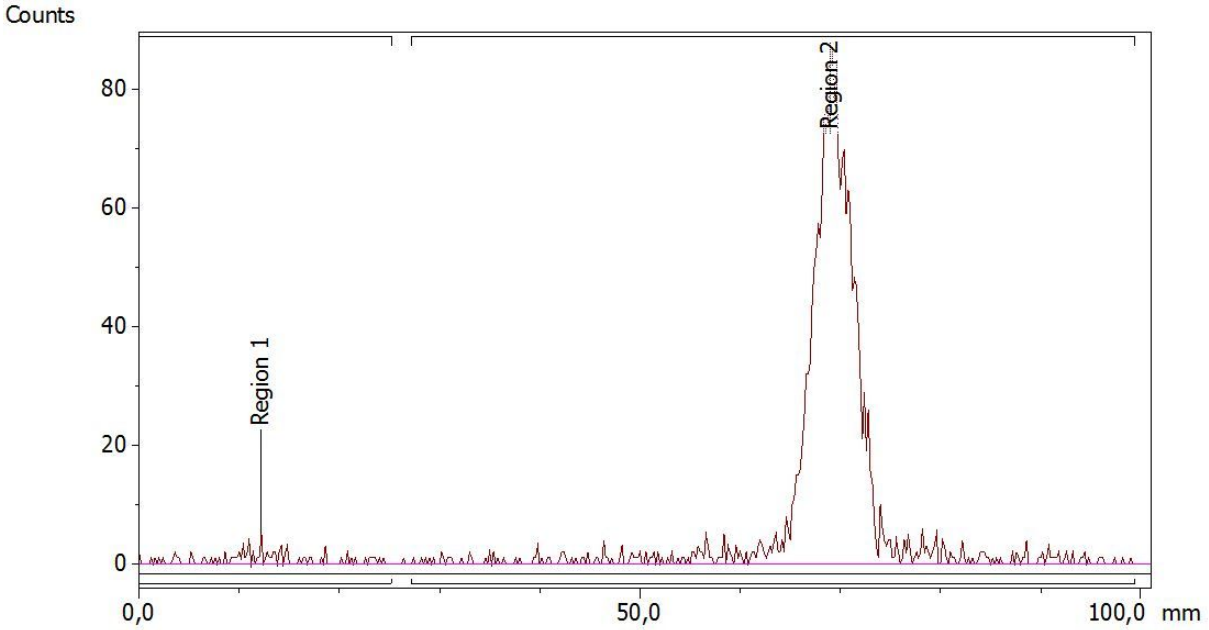

Figure S61: Radio-TLC chromatogram of [^{18}F]H_2N-Lys-(SiFA)SeFe-Gly-Lys-OH ($2X_1$) at pH 5.5 and

90°C, t = 60 min (flow agent: 60% MeCN/ 40% PBS (6/4 v/v) with 10% NaOAc in H_2O (2 M) and 1% TFA, stationary phase: TLC Silica gel 60 F254 from Merck Millipore).

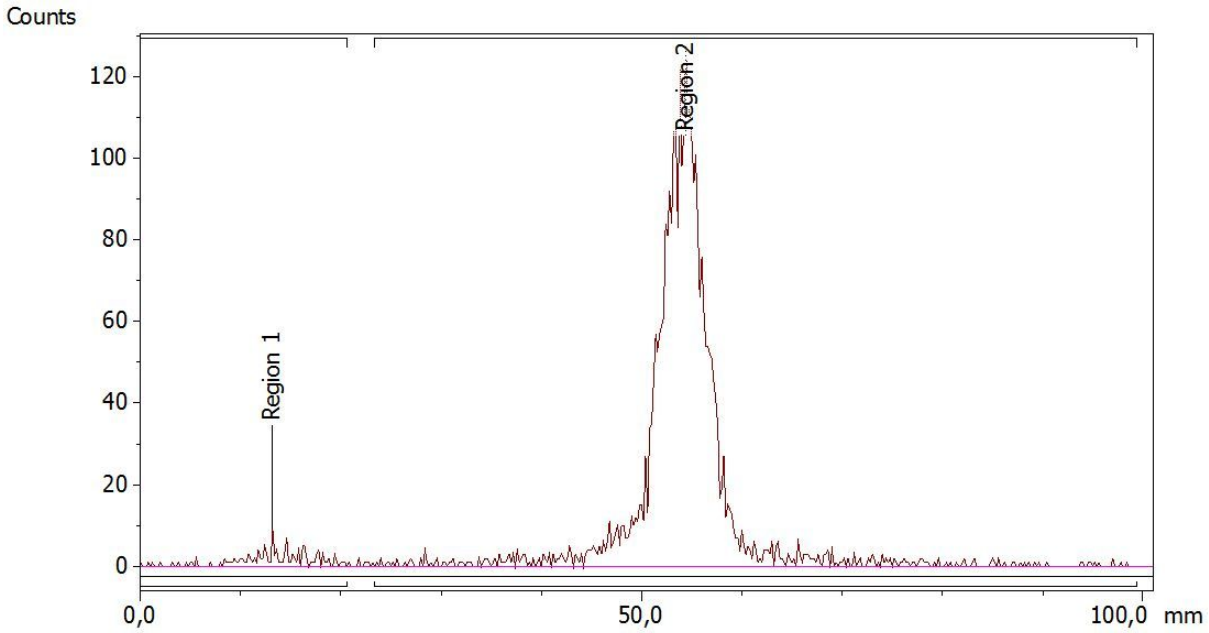

Figure S62: Radio-TLC chromatogram of [^{18}F]H_2N-Lys-(SiFA)SeFe-Gly-Lys-OH ($2X_1$) at pH 5.5 and 90°C, t = 90 min (flow agent: 60% MeCN/ 40% PBS (6/4 v/v) with 10% NaOAc in H_2O (2 M) and 1% TFA, stationary phase: TLC Silica gel 60 F254 from Merck Millipore).

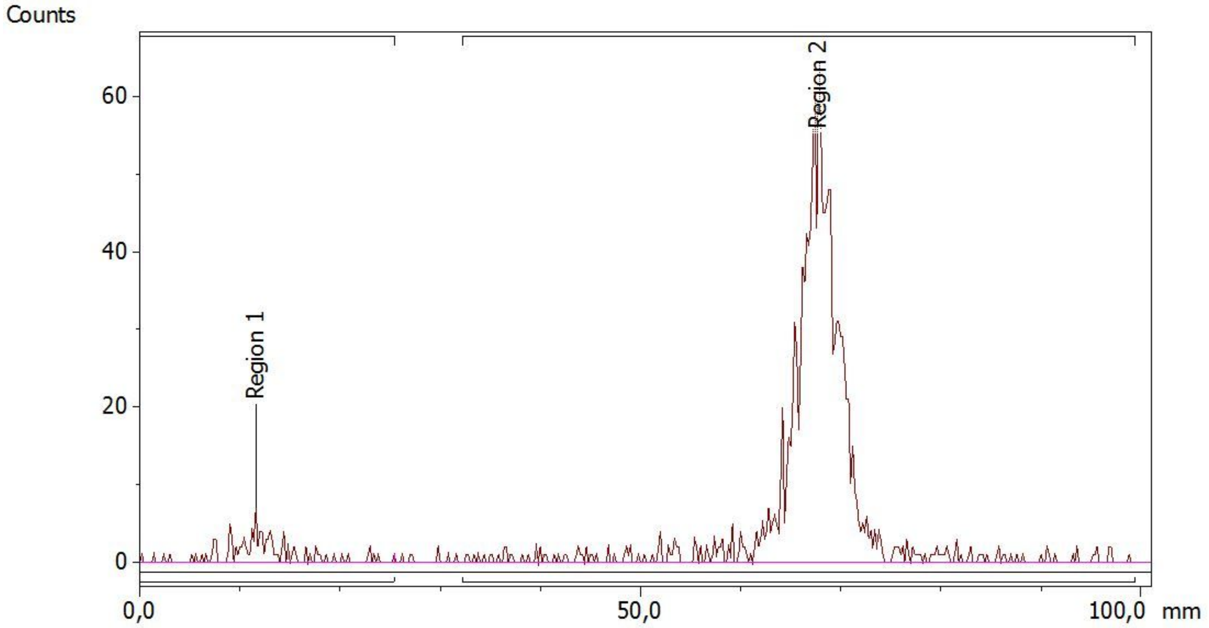

Figure S63: Radio-TLC chromatogram of [^{18}F]H$_2$N-Lys-(SiFA)SeFe-Gly-Lys-OH (2X$_1$) at pH 5.5 and 90°C, t = 120 min (flow agent: 60% MeCN/ 40% PBS (6/4 v/v) with 10% NaOAc in H$_2$O (2 M) and 1% TFA, stationary phase: TLC Silica gel 60 F254 from Merck Millipore).

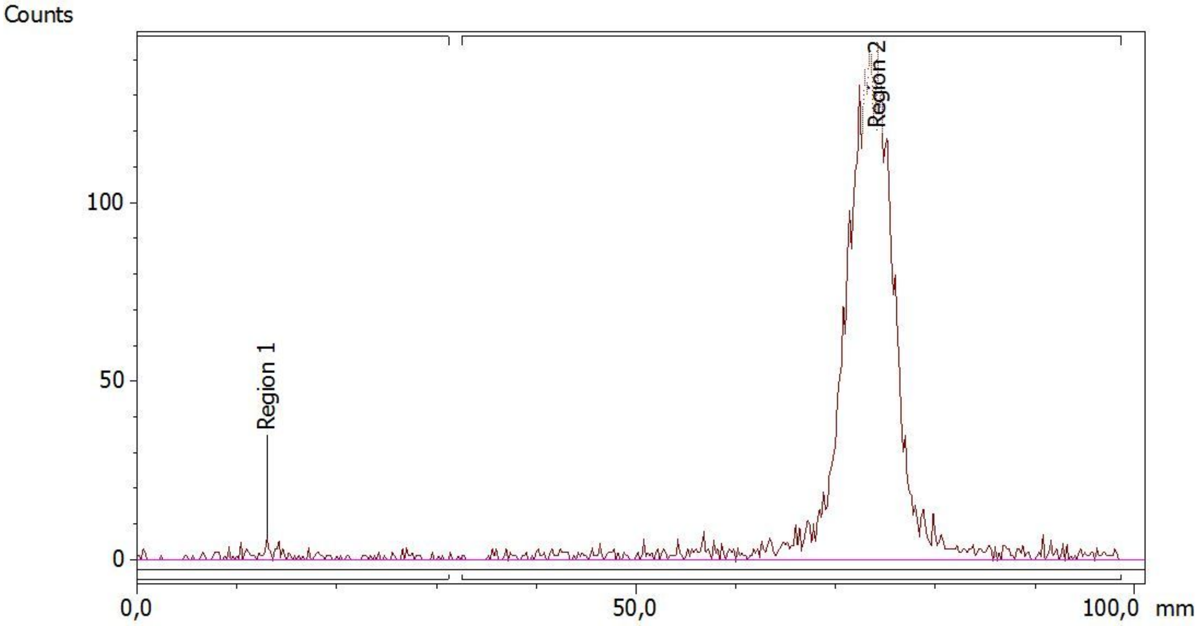

Figure S64: Radio-TLC chromatogram of [^{18}F]H$_2$N-Glu-(SiFA)SeFe-Gly-Lys-OH (3X$_1$) at pH 5.5 and 90°C, t = 0 min (flow agent: 60% MeCN/ 40% PBS (6/4 v/v) with 10% NaOAc in H$_2$O (2 M) and 1% TFA, stationary phase: TLC Silica gel 60 F254 from Merck Millipore).

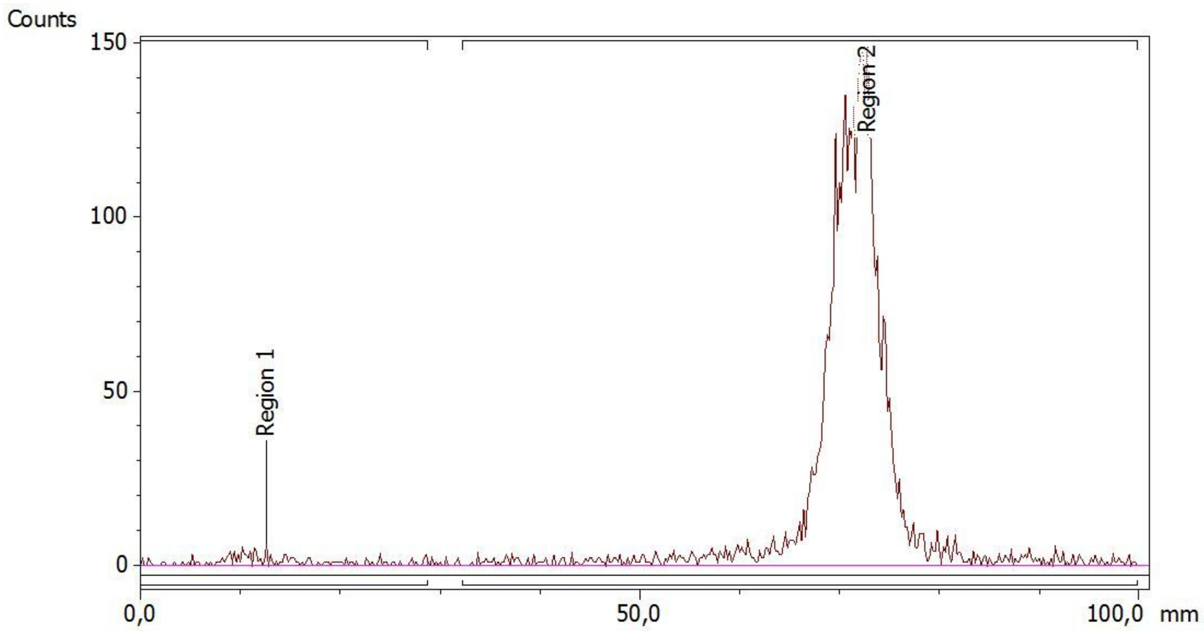

Figure S65: Radio-TLC chromatogram of [^{18}F]H_2N-Glu-(SiFA)SeFe-Gly-Lys-OH (3X_1) at pH 5.5 and 90°C, t = 30 min (flow agent: 60% MeCN/ 40% PBS (6/4 v/v) with 10% NaOAc in H_2O (2 M) and 1% TFA, stationary phase: TLC Silica gel 60 F254 from Merck Millipore).

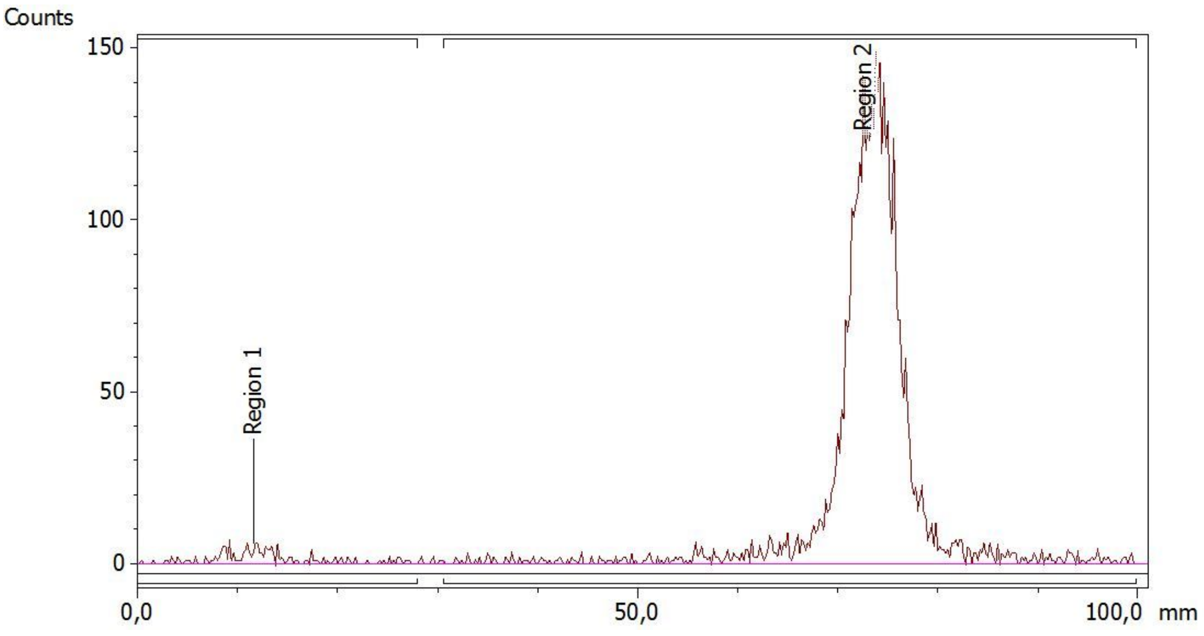

Figure S66: Radio-TLC chromatogram of [^{18}F]H_2N-Glu-(SiFA)SeFe-Gly-Lys-OH (3X_1) at pH 5.5 and 90 °C, t = 60 min (flow agent: 60% MeCN/ 40% PBS (6/4 v/v) with 10% NaOAc in H_2O (2 M) and 1% TFA, stationary phase: TLC Silica gel 60 F254 from Merck Millipore).

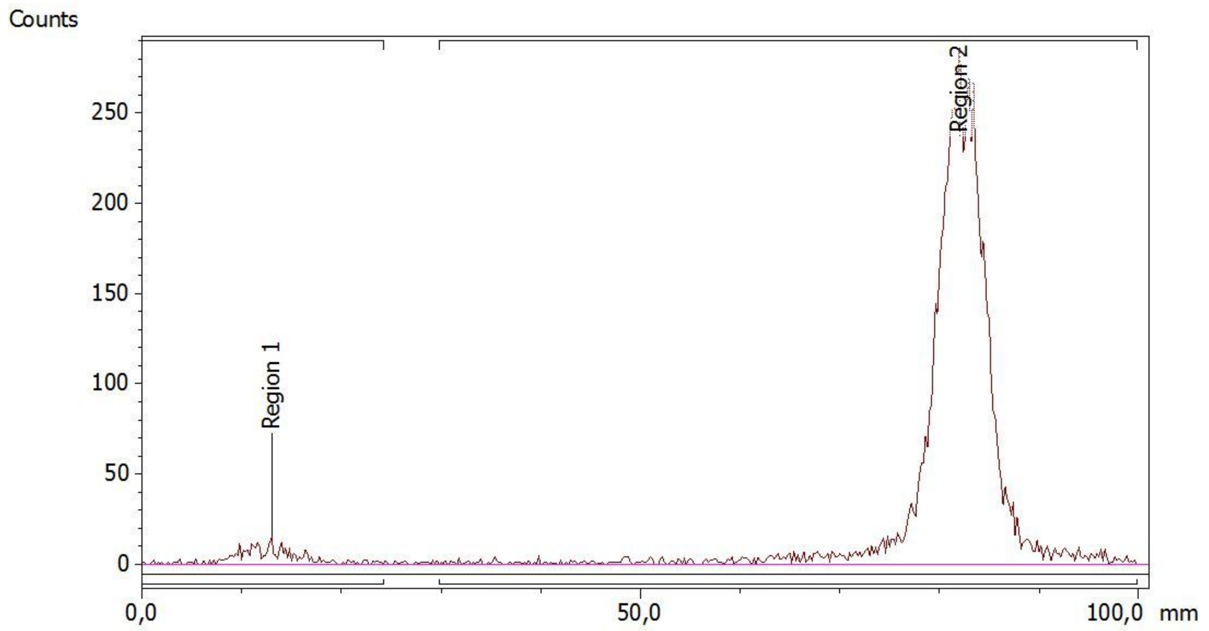

Figure S67: Radio-TLC chromatogram of [^{18}F]H_2N-Glu-(SiFA)SeFe-Gly-Lys-OH (3X_1) at pH 5.5 and 90°C, t = 90 min (flow agent: 60% MeCN/ 40% PBS (6/4 v/v) with 10% NaOAc in H_2O (2 M) and 1% TFA, stationary phase: TLC Silica gel 60 F254 from Merck Millipore).

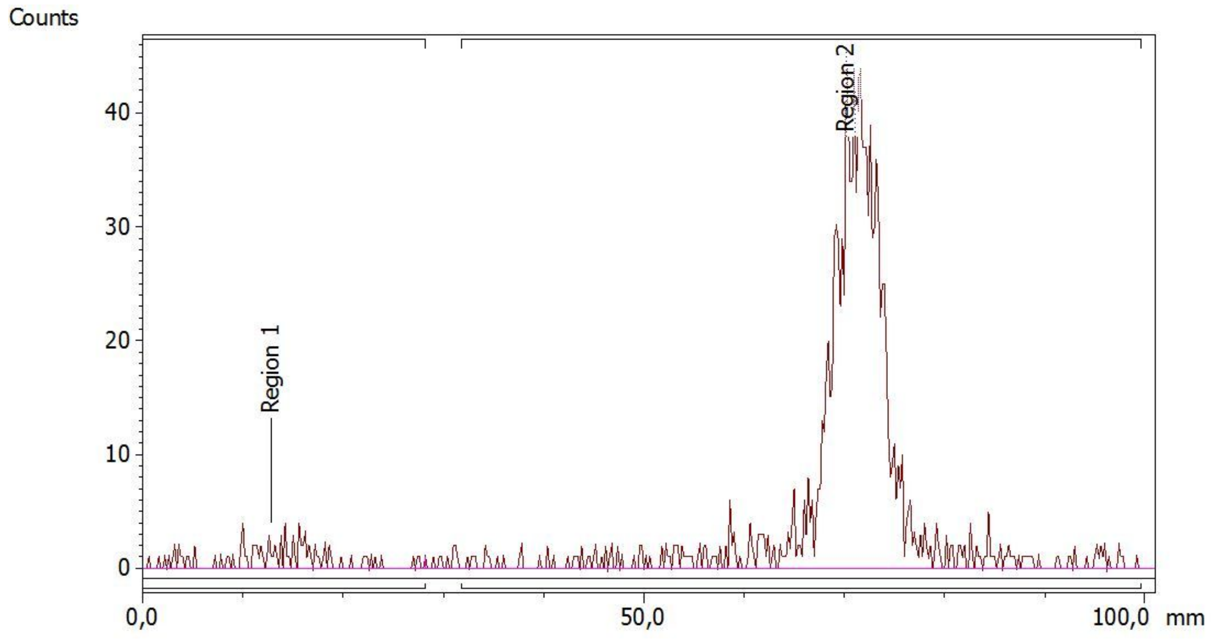

Figure S68: Radio-TLC chromatogram of [^{18}F]H_2N-Glu-(SiFA)SeFe-Gly-Lys-OH (3X_1) at pH 5.5 and 90°C, t = 120 min (flow agent: 60% MeCN/ 40% PBS (6/4 v/v) with 10% NaOAc in H_2O (2 M) and 1% TFA, stationary phase: TLC Silica gel 60 F254 from Merck Millipore).

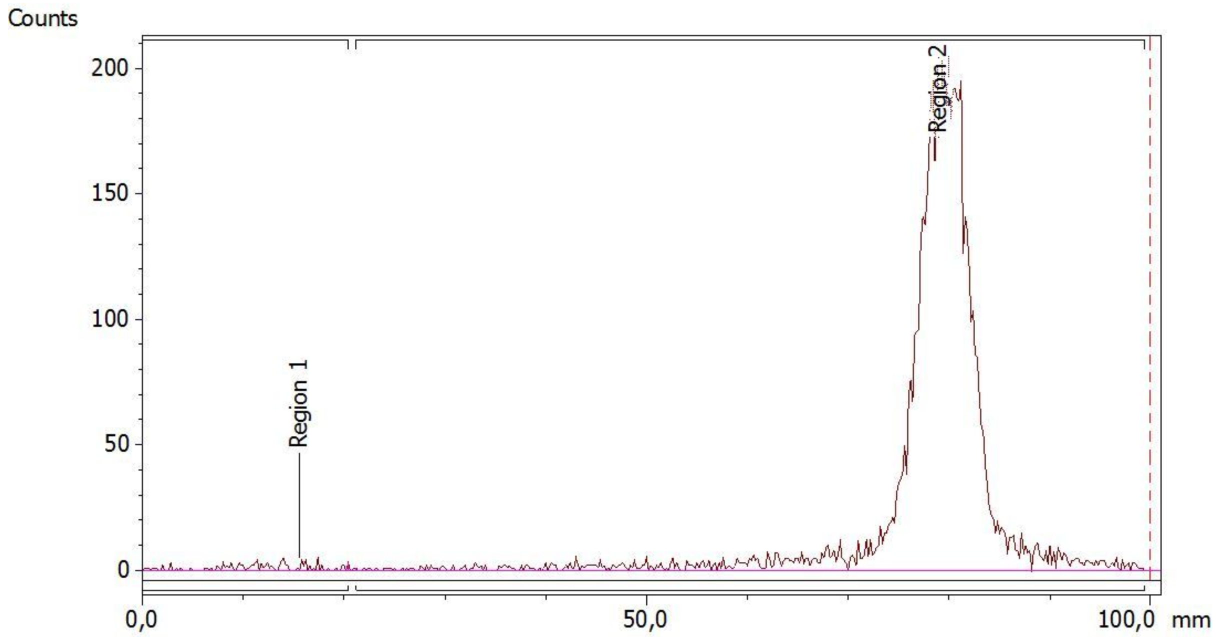

Figure S69: Radio-TLC chromatogram of [^{18}F]H_2N-(SiFA)SeFe-Gly-Asp-OH (1X_2) at pH 5.5 and 90°C, t = 0 min (flow agent: 60% MeCN/ 40% PBS (6/4 v/v) with 10% NaOAc in H_2O (2 M) and 1% TFA, stationary phase: TLC Silica gel 60 F254 from Merck Millipore).

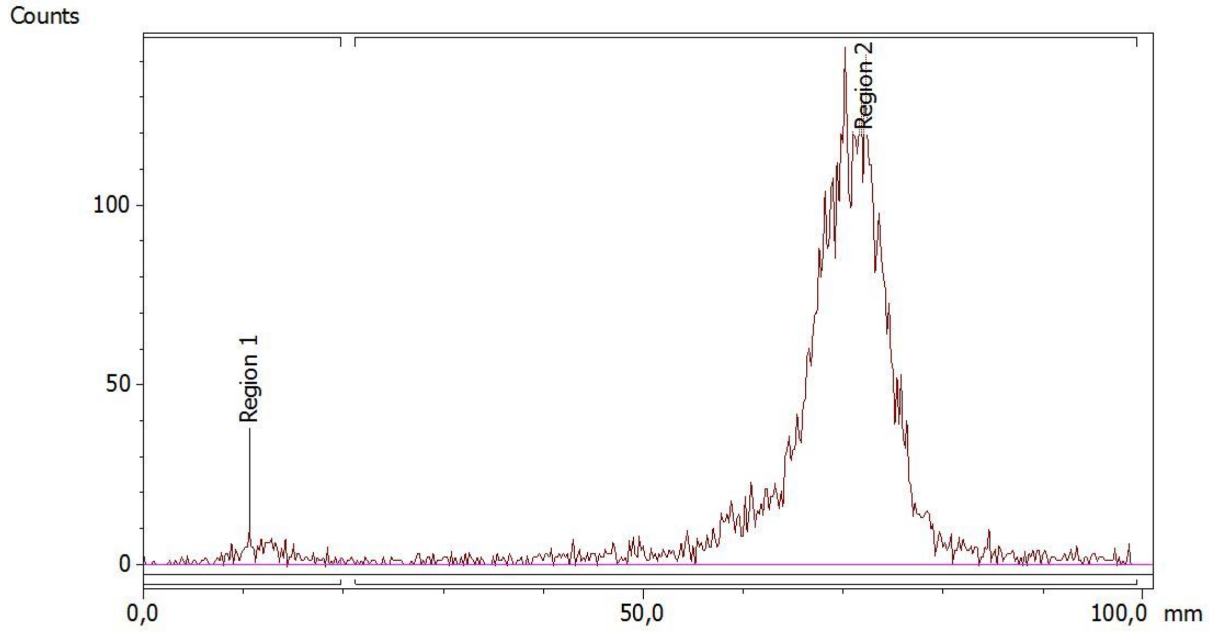

Figure S70: Radio-TLC chromatogram of [^{18}F]H_2N-(SiFA)SeFe-Gly-Asp-OH (1X_2) at pH 5.5 and 90°C, t = 30 min (flow agent: 60% MeCN/ 40% PBS (6/4 v/v) with 10% NaOAc in H_2O (2 M) and 1% TFA, stationary phase: TLC Silica gel 60 F254 from Merck Millipore).

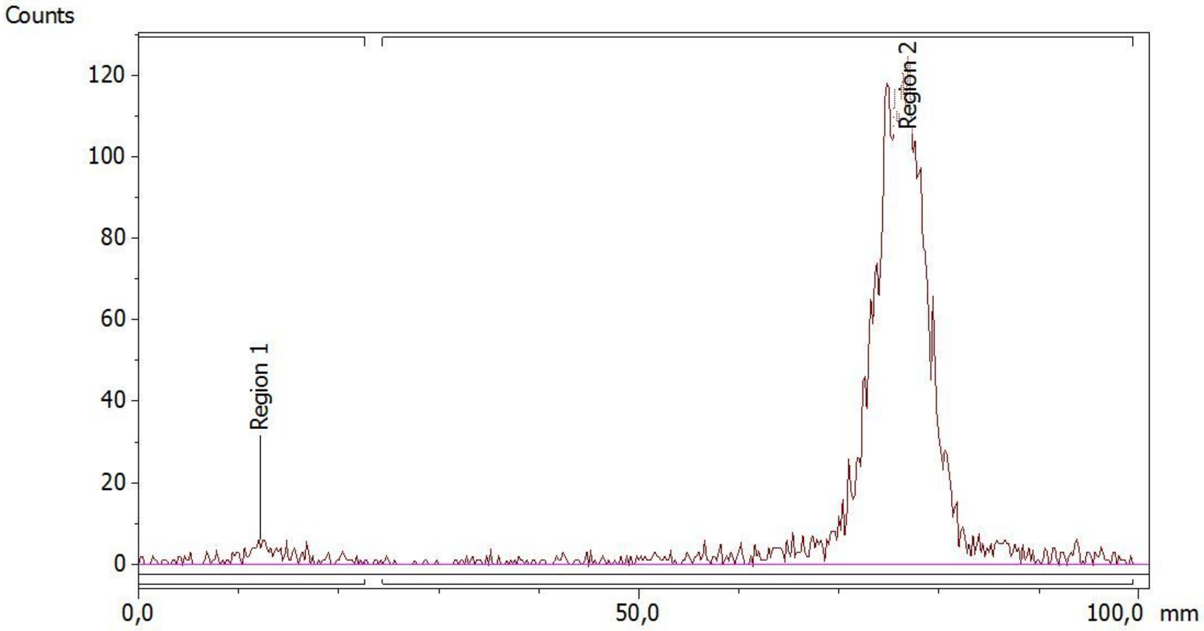

Figure S71: Radio-TLC chromatogram of [^{18}F]H_2N-(SiFA)SeFe-Gly-Asp-OH (1X_2) at pH 5.5 and 90°C, t = 60 min (flow agent: 60% MeCN/ 40% PBS (6/4 v/v) with 10% NaOAc in H_2O (2 M) and 1% TFA, stationary phase: TLC Silica gel 60 F254 from Merck Millipore).

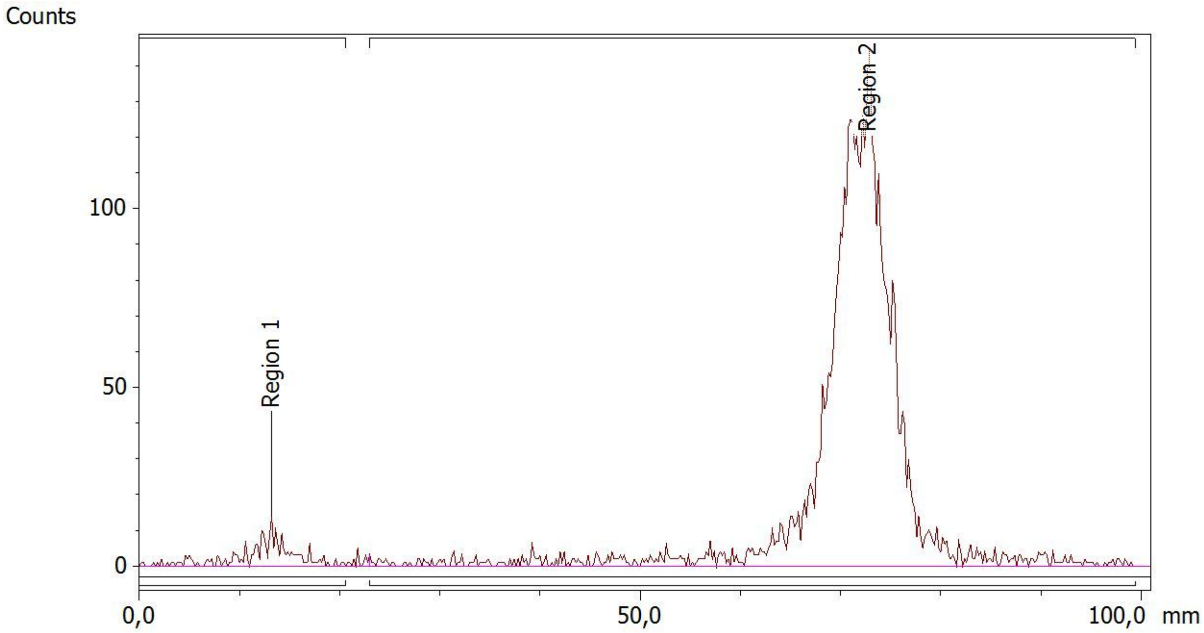

Figure S72: Radio-TLC chromatogram of [^{18}F]H_2N-(SiFA)SeFe-Gly-Asp-OH (1X_2) at pH 5.5 and 90°C, t = 90 min (flow agent: 60% MeCN/ 40% PBS (6/4 v/v) with 10% NaOAc in H_2O (2 M) and 1% TFA, stationary phase: TLC Silica gel 60 F254 from Merck Millipore).

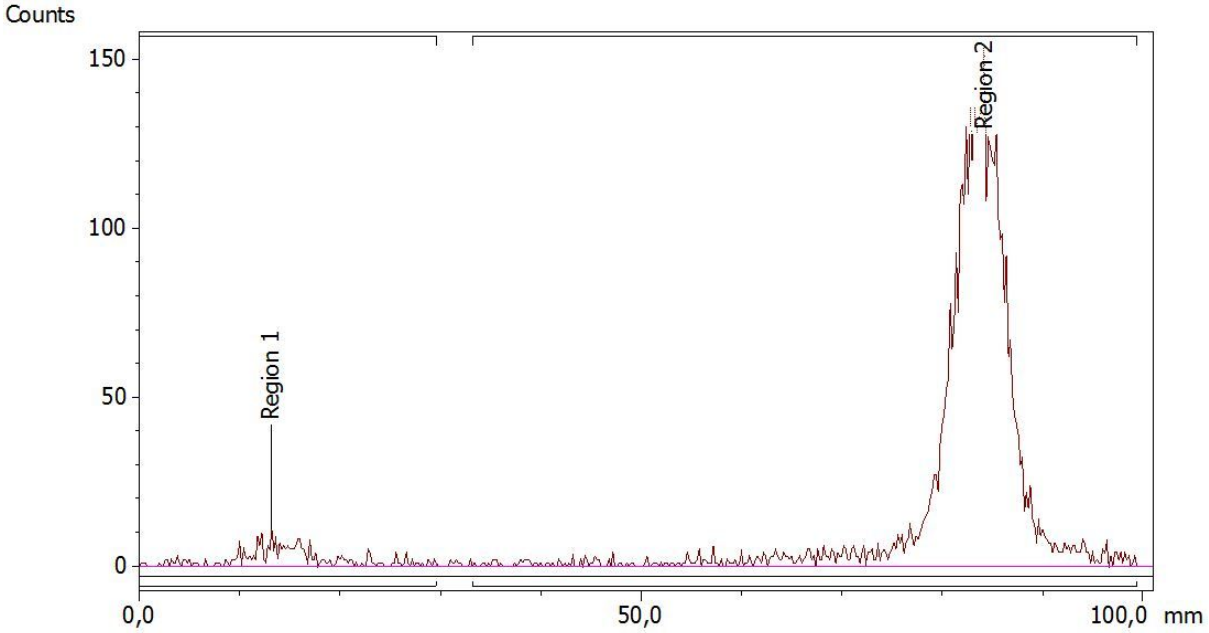

Figure S73: Radio-TLC chromatogram of [^{18}F]H_2N-(SiFA)SeFe-Gly-Asp-OH (1X_2) at pH 5.5 and 90°C, t = 120 min (flow agent: 60% MeCN/ 40% PBS (6/4 v/v) with 10% NaOAc in H_2O (2 M) and 1% TFA, stationary phase: TLC Silica gel 60 F254 from Merck Millipore).

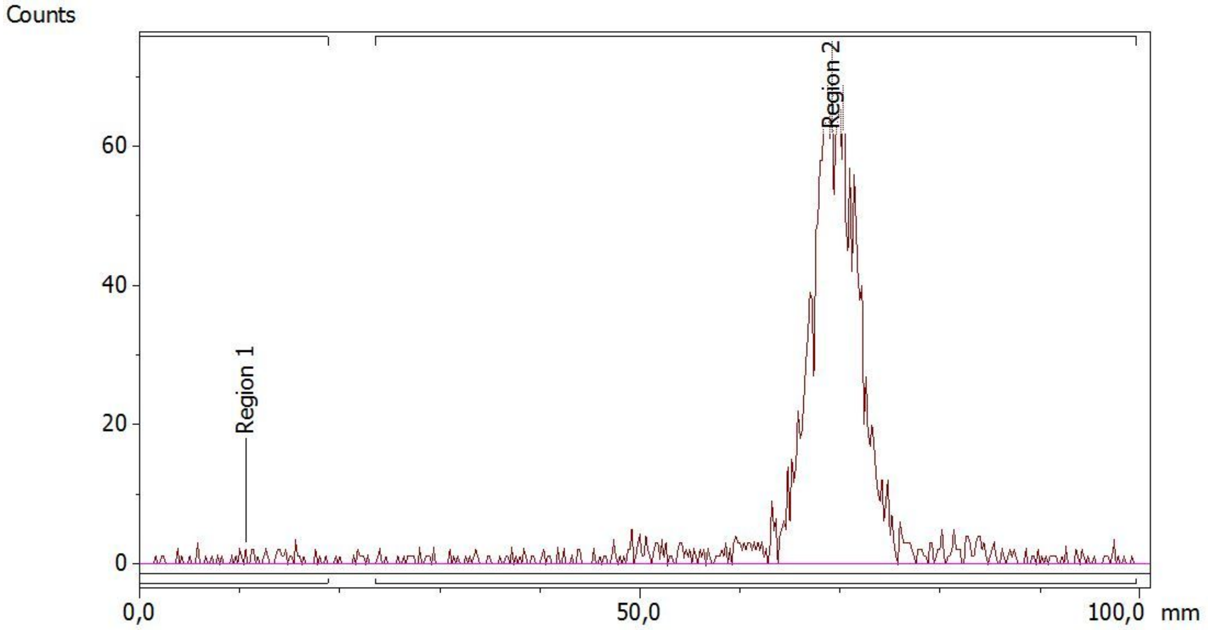

Figure S74: Radio-TLC chromatogram of [^{18}F]H_2N-Lys-(SiFA)SeFe-Gly-Asp-OH (2X_2) at pH 5.5 and 90°C, t = 0 min (flow agent: 60% MeCN/ 40% PBS (6/4 v/v) with 10% NaOAc in H_2O (2 M) and 1% TFA, stationary phase: TLC Silica gel 60 F254 from Merck Millipore).

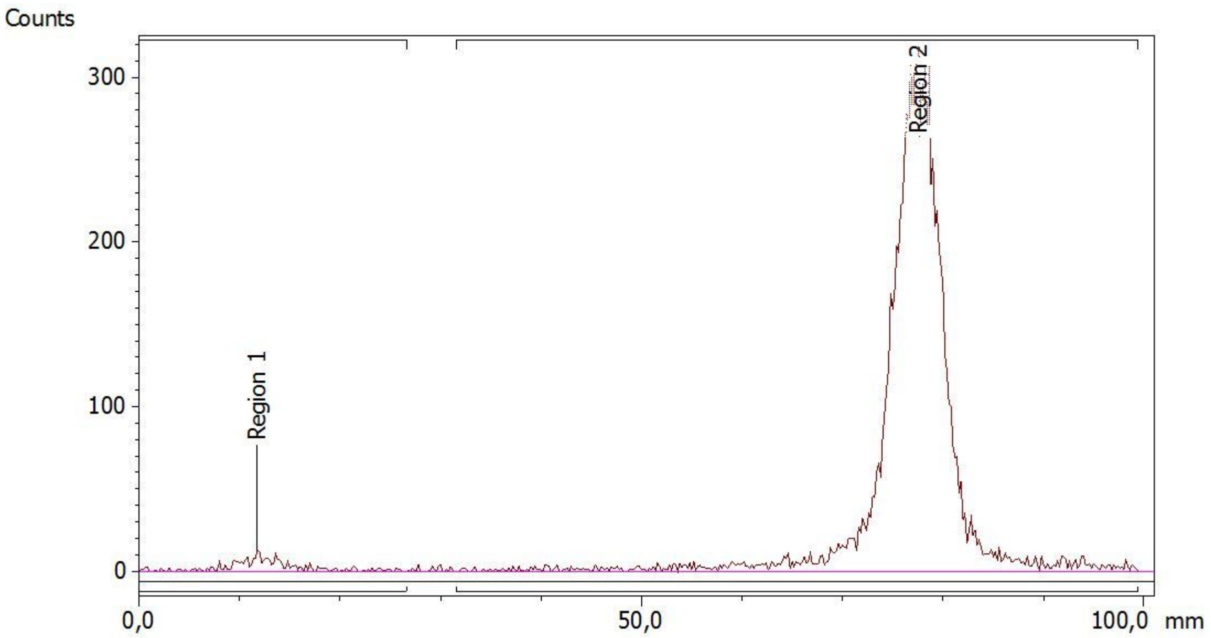

Figure S75: Radio-TLC chromatogram of [^{18}F]H_2N-Lys-(SiFA)SeFe-Gly-Asp-OH ($2X_2$) at pH 5.5 and 90 °C, t = 30 min (flow agent: 60% MeCN/ 40% PBS (6/4 v/v) with 10% NaOAc in H_2O (2 M) and 1% TFA, stationary phase: TLC Silica gel 60 F254 from Merck Millipore).

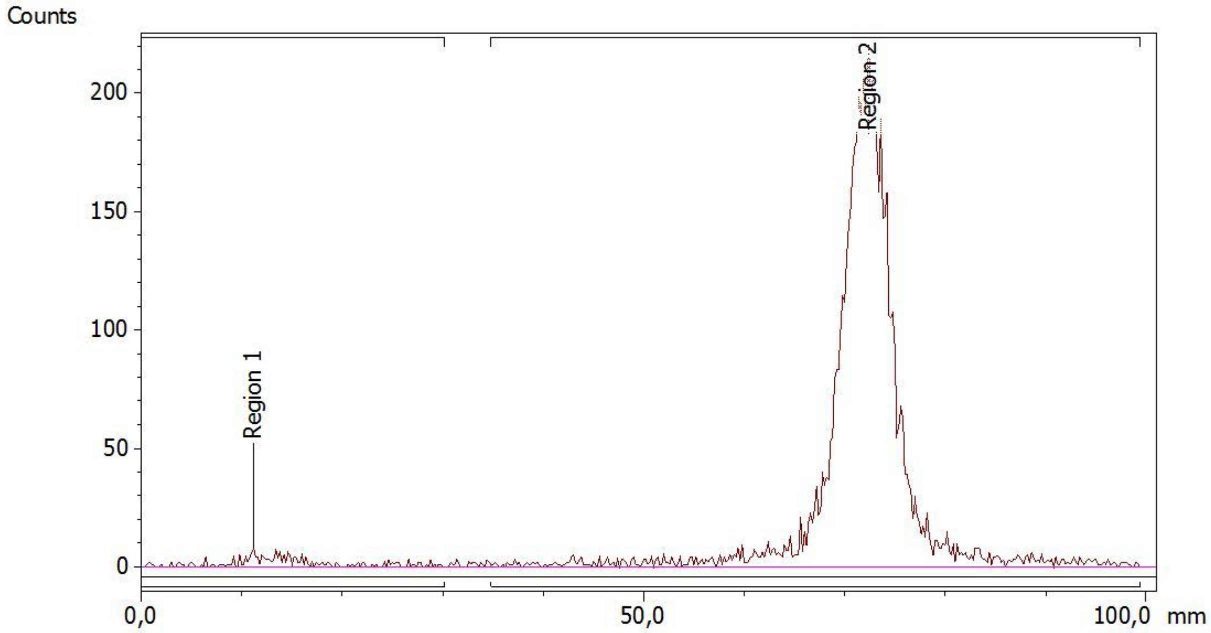

Figure S76: Radio-TLC chromatogram of [^{18}F]H_2N-Lys-(SiFA)SeFe-Gly-Asp-OH ($2X_2$) at pH 5.5 and 90 °C, t = 60 min (flow agent: 60% MeCN/ 40% PBS (6/4 v/v) with 10% NaOAc in H_2O (2 M) and 1% TFA, stationary phase: TLC Silica gel 60 F254 from Merck Millipore).

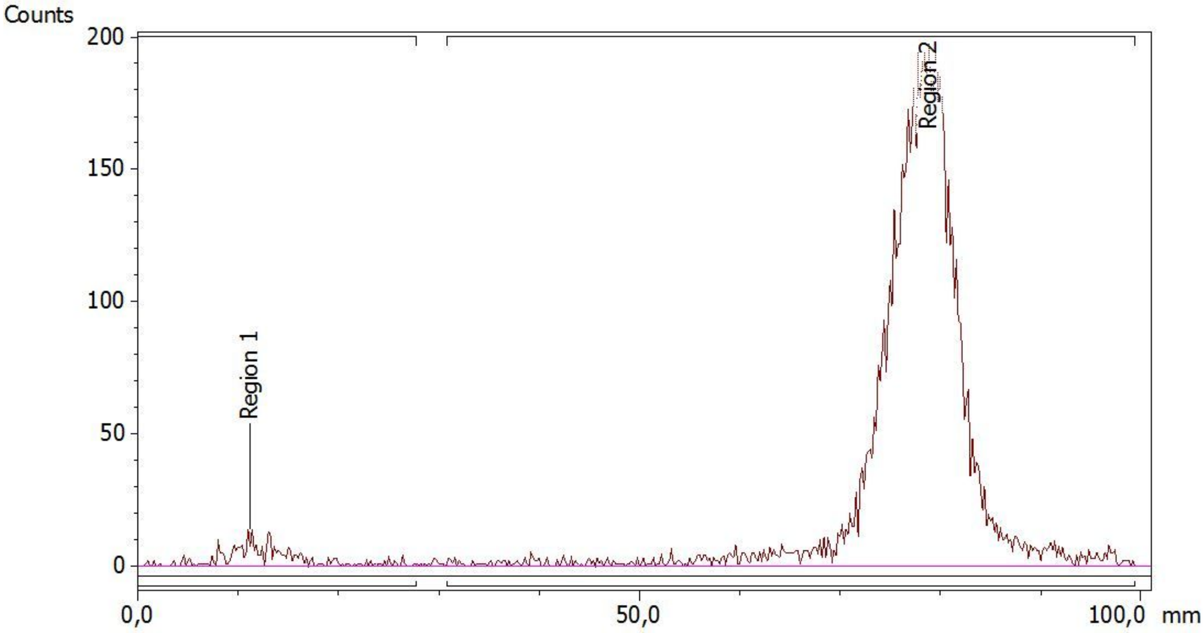

Figure S77: Radio-TLC chromatogram of [^{18}F]H$_2$N-Lys-(SiFA)SeFe-Gly-Asp-OH (2X$_2$) at pH 5.5 and 90 °C, t = 90 min (flow agent: 60% MeCN/ 40% PBS (6/4 v/v) with 10% NaOAc in H_2O (2 M) and 1% TFA, stationary phase: TLC Silica gel 60 F254 from Merck Millipore).

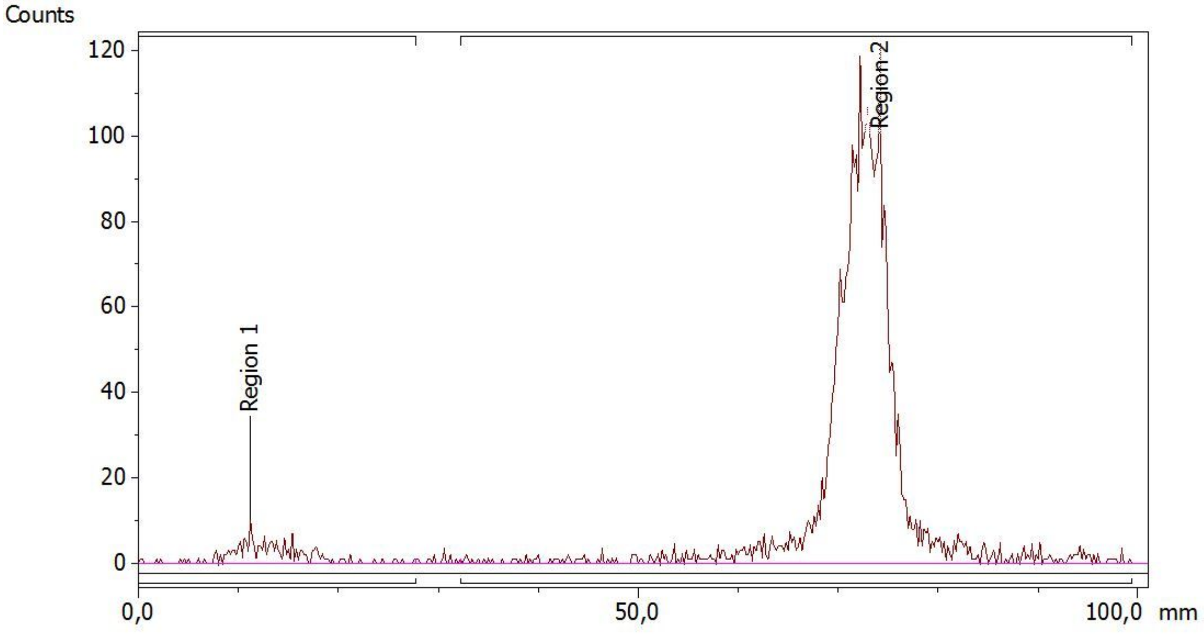

Figure S78: Radio-TLC chromatogram of [^{18}F]H$_2$N-Lys-(SiFA)SeFe-Gly-Asp-OH (2X$_2$) at pH 5.5 and 90 °C, t = 120 min (flow agent: 60% MeCN/ 40% PBS (6/4 v/v) with 10% NaOAc in H_2O (2 M) and 1% TFA, stationary phase: TLC Silica gel 60 F254 from Merck Millipore).

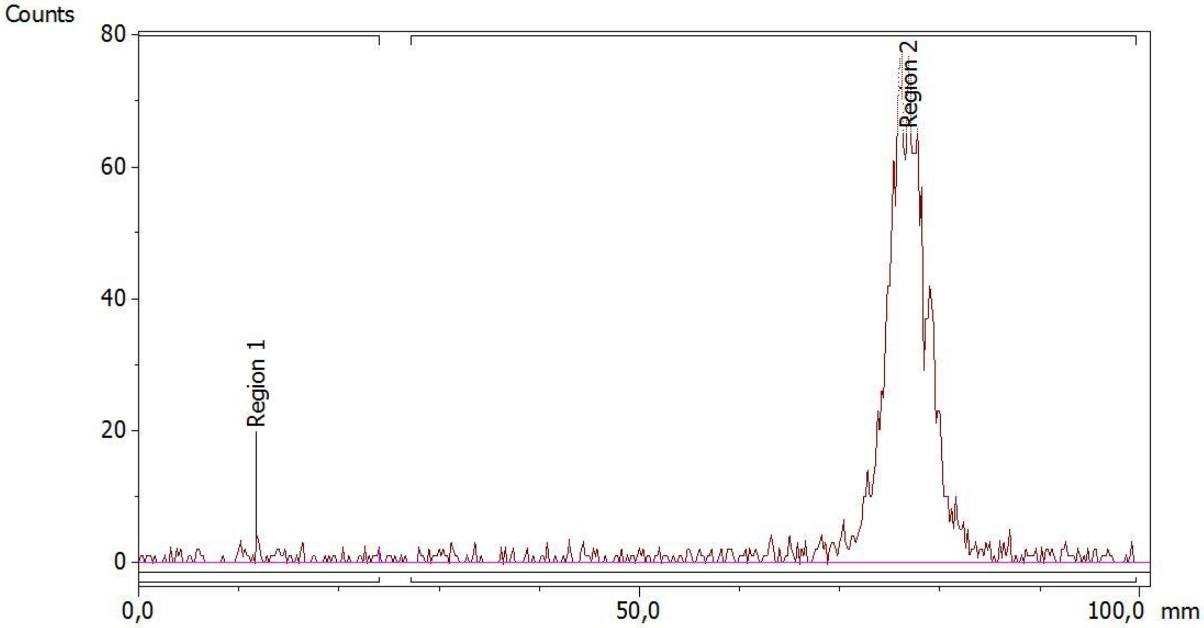

Figure S79: Radio-TLC chromatogram of [^{18}F]H_2N-Glu-(SiFA)SeFe-Gly-Asp-OH ($3X_2$) at pH 5.5 and 90 °C, t = 0 min (flow agent: 60% MeCN/ 40% PBS (6/4 v/v) with 10% NaOAc in H_2O (2 M) and 1% TFA, stationary phase: TLC Silica gel 60 F254 from Merck Millipore).

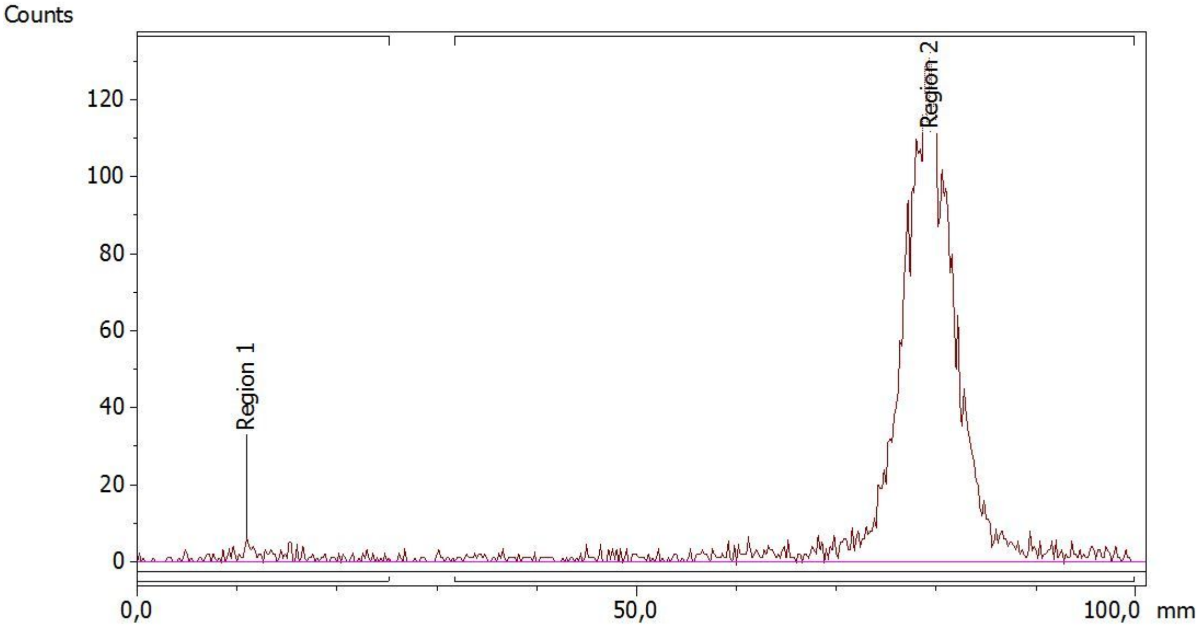

Figure S80: Radio-TLC chromatogram of [^{18}F]H_2N-Glu-(SiFA)SeFe-Gly-Asp-OH ($3X_2$) at pH 5.5 and 90 °C, t = 30 min (flow agent: 60% MeCN/ 40% PBS (6/4 v/v) with 10% NaOAc in H_2O (2 M) and 1% TFA, stationary phase: TLC Silica gel 60 F254 from Merck Millipore).

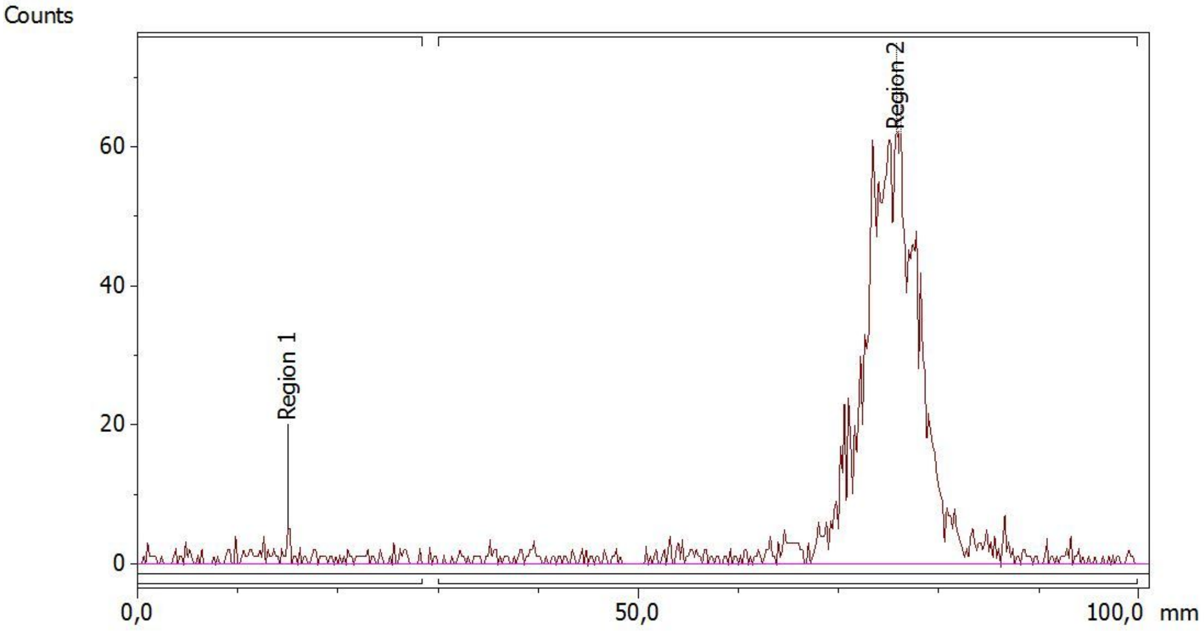

Figure S81: Radio-TLC chromatogram of [^{18}F]H_2N-Glu-(SiFA)SeFe-Gly-Asp-OH ($3X_2$) at pH 5.5 and 90 °C, t = 60 min (flow agent: 60% MeCN/ 40% PBS (6/4 v/v) with 10% NaOAc in H_2O (2 M) and 1% TFA, stationary phase: TLC Silica gel 60 F254 from Merck Millipore).

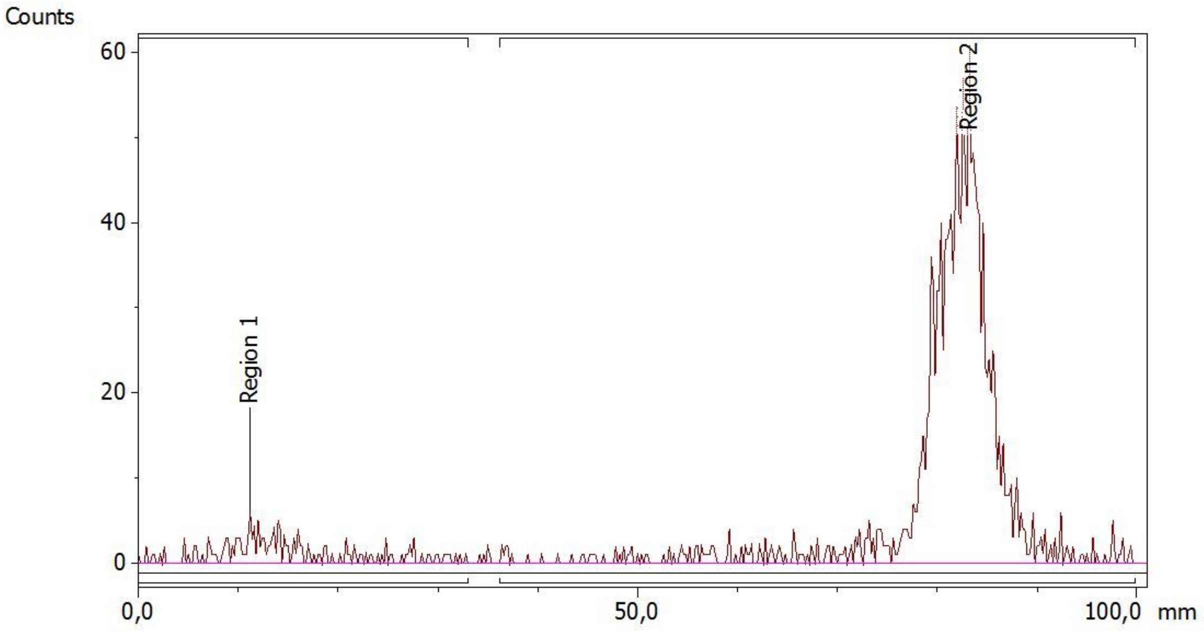

Figure S82: Radio-TLC chromatogram of [^{18}F]H_2N-Glu-(SiFA)SeFe-Gly-Asp-OH ($3X_2$) at pH 5.5 and 90 °C, t = 90 min (flow agent: 60% MeCN/ 40% PBS (6/4 v/v) with 10% NaOAc in H_2O (2 M) and 1% TFA, stationary phase: TLC Silica gel 60 F254 from Merck Millipore).

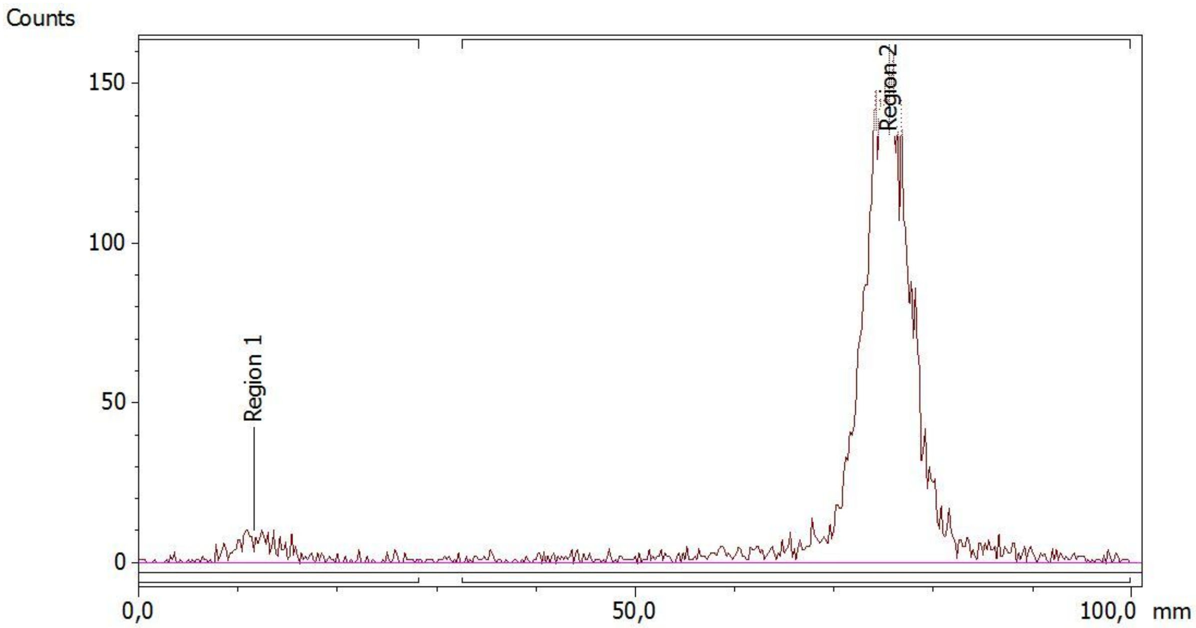

Figure S83: Radio-TLC chromatogram of [^{18}F]H$_2$N-Glu-(SiFA)SeFe-Gly-Asp-OH (3X$_2$) at pH 5.5 and 90 °C, t = 120 min (flow agent: 60% MeCN/ 40% PBS (6/4 v/v) with 10% NaOAc in H$_2$O (2 M) and 1% TFA, stationary phase: TLC Silica gel 60 F254 from Merck Millipore).

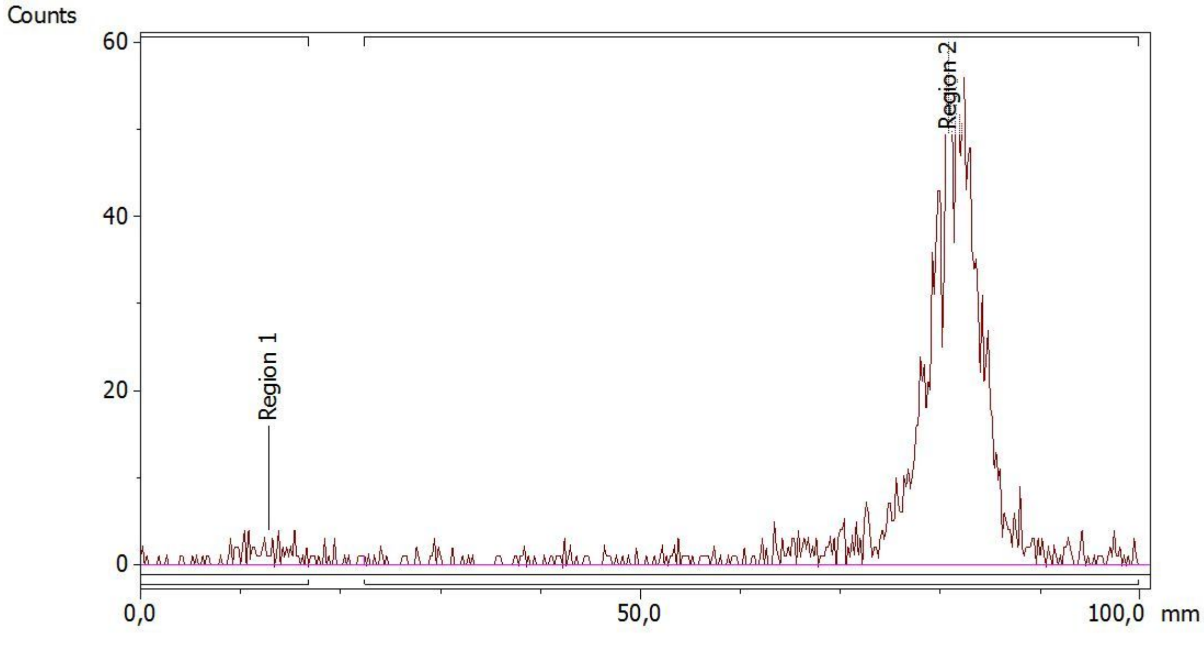

Figure S84: Radio-TLC chromatogram of [^{18}F]H$_2$N-(SiFA)SeFe-Gly-Tyr-OH (1X$_3$) at pH 5.5 and 90 °C, t = 0 min (flow agent: 60% MeCN/ 40% PBS (6/4 v/v) with 10% NaOAc in H$_2$O (2 M) and 1% TFA, stationary phase: TLC Silica gel 60 F254 from Merck Millipore).

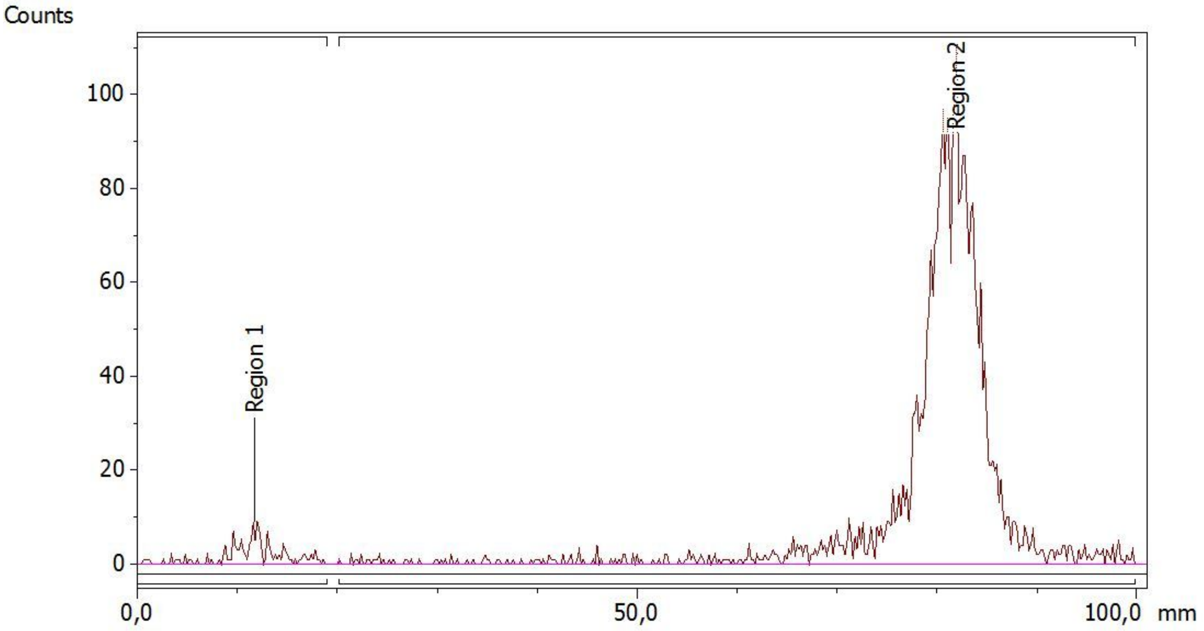

Figure S85: Radio-TLC chromatogram of [^{18}F]H_2N-(SiFA)SeFe-Gly-Tyr-OH (1X_3) at pH 5.5 and 90 °C, t = 30 min (flow agent: 60% MeCN/ 40% PBS (6/4 v/v) with 10% NaOAc in H_2O (2 M) and 1% TFA, stationary phase: TLC Silica gel 60 F254 from Merck Millipore).

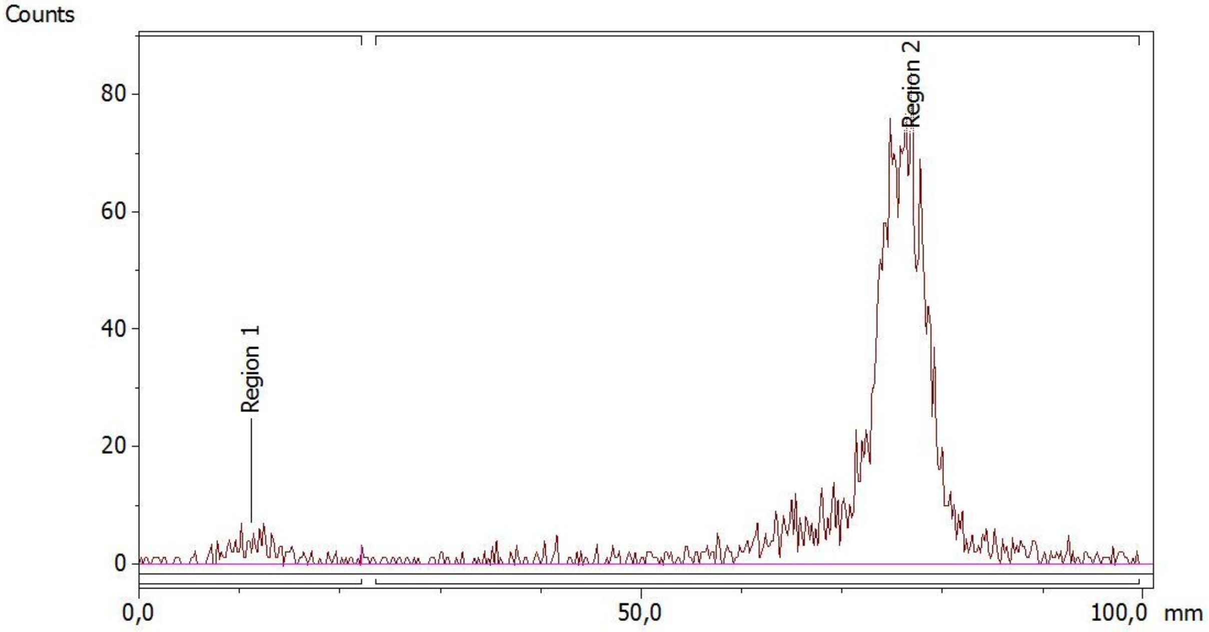

Figure S86: Radio-TLC chromatogram of [^{18}F]H_2N-(SiFA)SeFe-Gly-Tyr-OH (1X_3) at pH 5.5 and 90 °C, t = 60 min (flow agent: 60% MeCN/ 40% PBS (6/4 v/v) with 10% NaOAc in H_2O (2 M) and 1% TFA, stationary phase: TLC Silica gel 60 F254 from Merck Millipore).

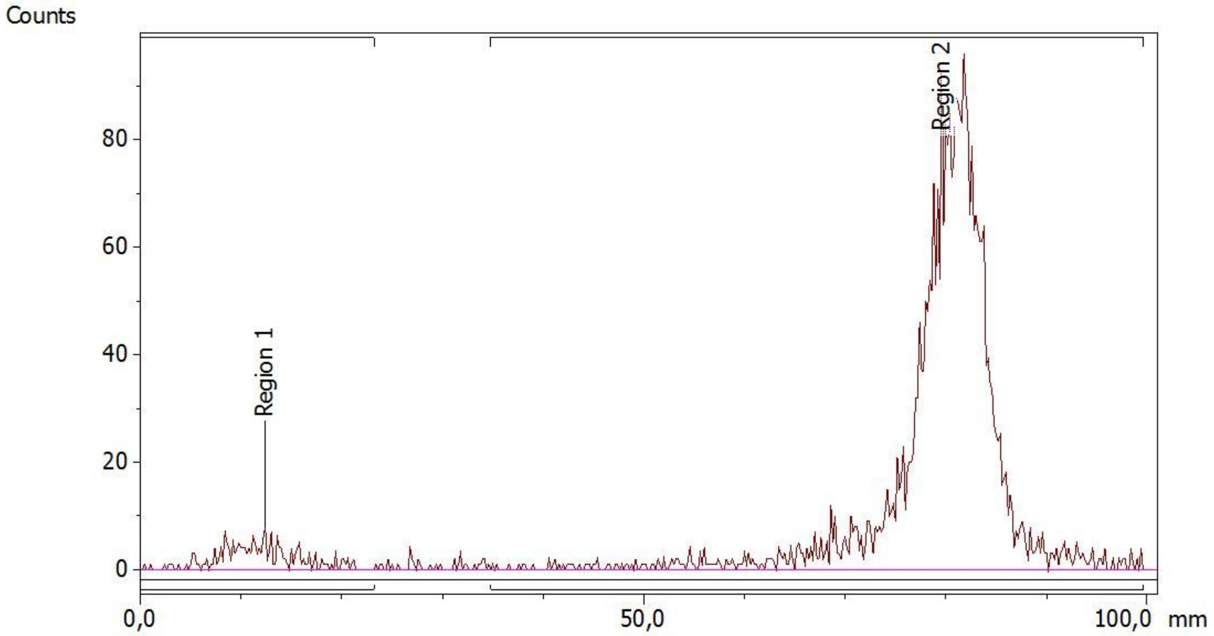

Figure S87: Radio-TLC chromatogram of [^{18}F]H_2N-(SiFA)SeFe-Gly-Tyr-OH (1X_3) at pH 5.5 and 90 °C, t = 90 min (flow agent: 60% MeCN/ 40% PBS (6/4 v/v) with 10% NaOAc in H_2O (2 M) and 1% TFA, stationary phase: TLC Silica gel 60 F254 from Merck Millipore).

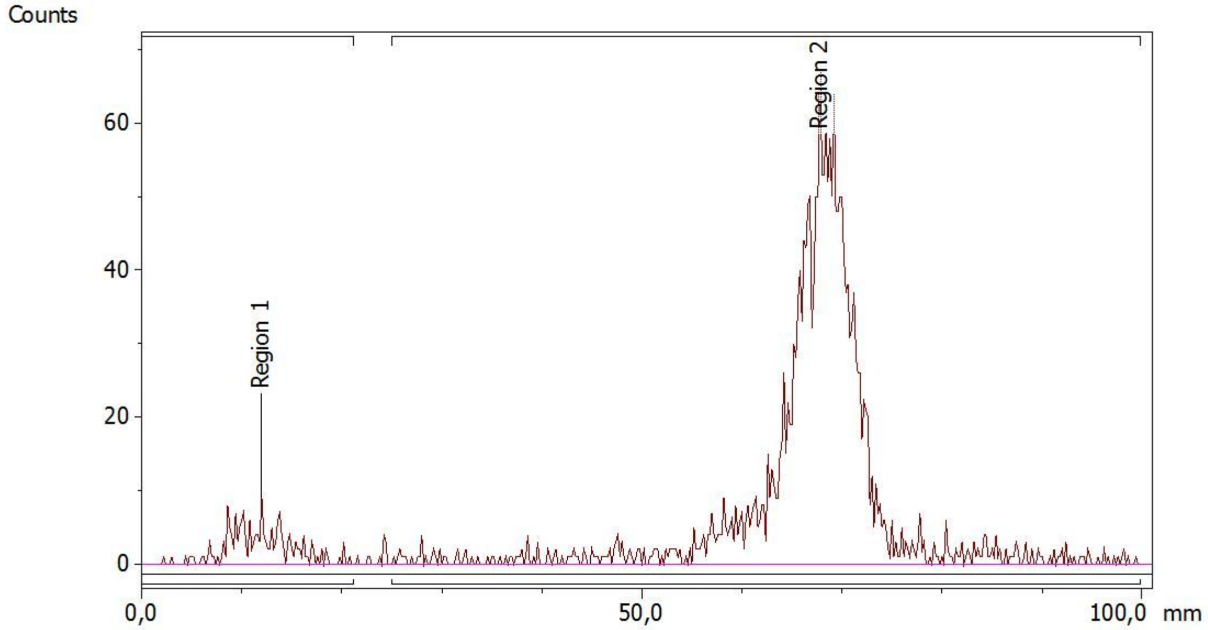

Figure S88: Radio-TLC chromatogram of [^{18}F]H_2N-(SiFA)SeFe-Gly-Tyr-OH (1X_3) at pH 5.5 and 90 °C, t = 120 min (flow agent: 60% MeCN/ 40% PBS (6/4 v/v) with 10% NaOAc in H_2O (2 M) and 1% TFA, stationary phase: TLC Silica gel 60 F254 from Merck Millipore).

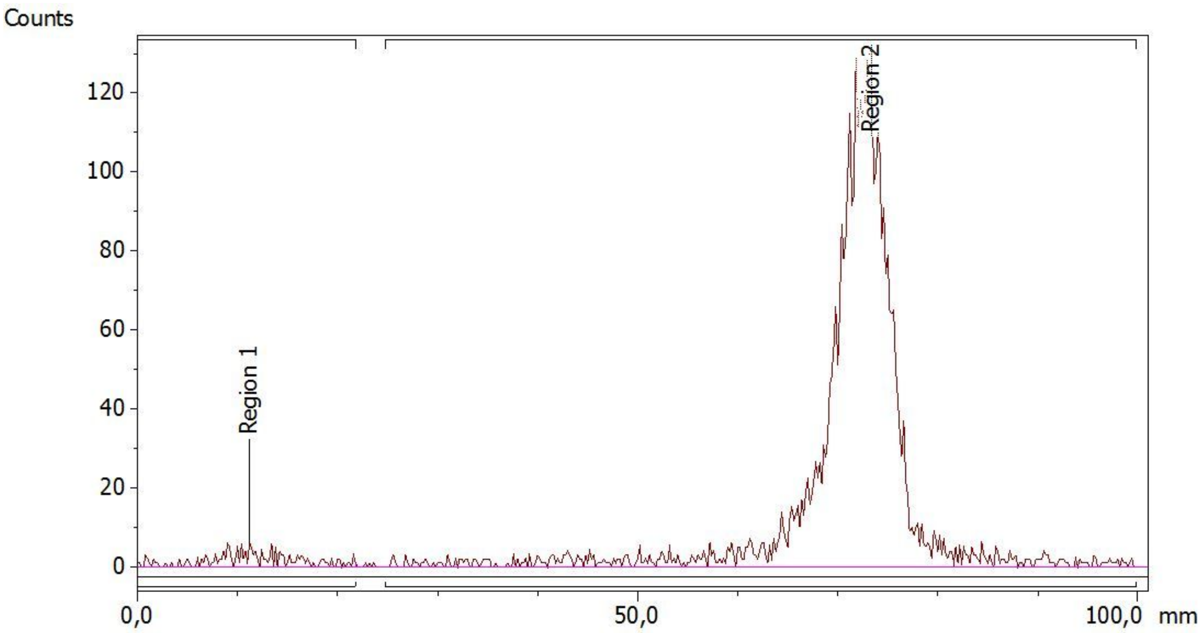

Figure S89: Radio-TLC chromatogram of [^{18}F]H_2N-Lys-(SiFA)SeFe-Gly-Tyr-OH ($2X_3$) at pH 5.5 and 90 °C, t = 0 min (flow agent: 60% MeCN/ 40% PBS (6/4 v/v) with 10% NaOAc in H_2O (2 M) and 1% TFA, stationary phase: TLC Silica gel 60 F254 from Merck Millipore).

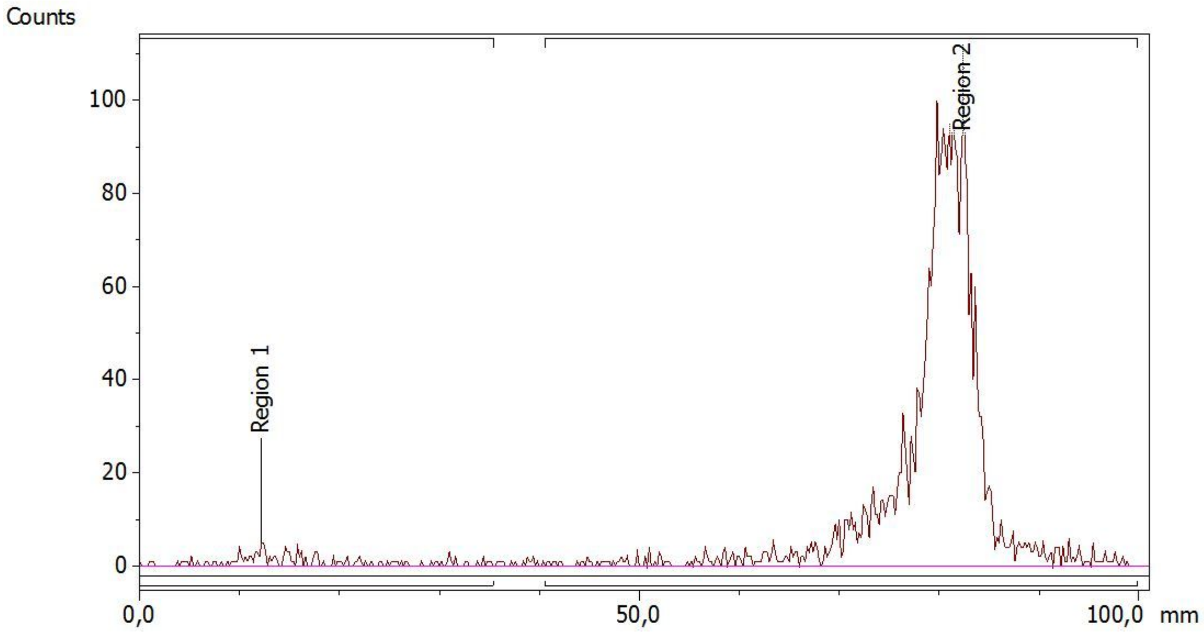

Figure S90: Radio-TLC chromatogram of [^{18}F]H_2N-Lys-(SiFA)SeFe-Gly-Tyr-OH ($2X_3$) at pH 5.5 and 90 °C, t = 30 min (flow agent: 60% MeCN/ 40% PBS (6/4 v/v) with 10% NaOAc in H_2O (2 M) and 1% TFA, stationary phase: TLC Silica gel 60 F254 from Merck Millipore).

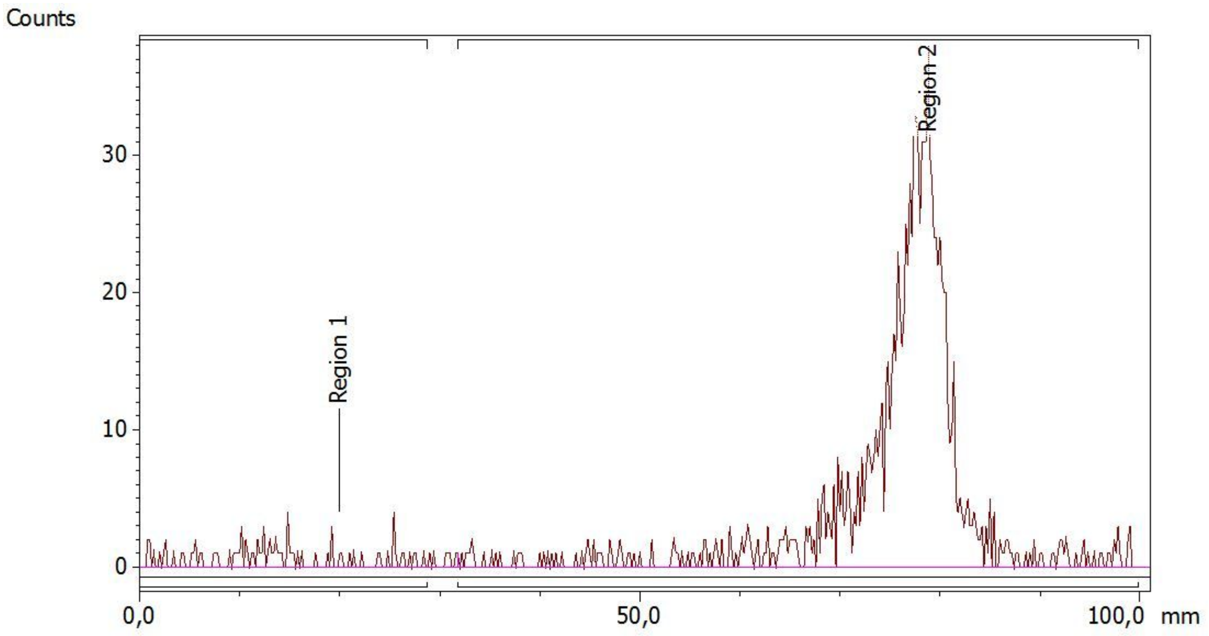

Figure S91: Radio-TLC chromatogram of [^{18}F]H_2N-Lys-(SiFA)SeFe-Gly-Tyr-OH ($2X_3$) at pH 5.5 and 90 °C, t = 60 min (flow agent: 60% MeCN/ 40% PBS (6/4 v/v) with 10% NaOAc in H_2O (2 M) and 1% TFA, stationary phase: TLC Silica gel 60 F254 from Merck Millipore).

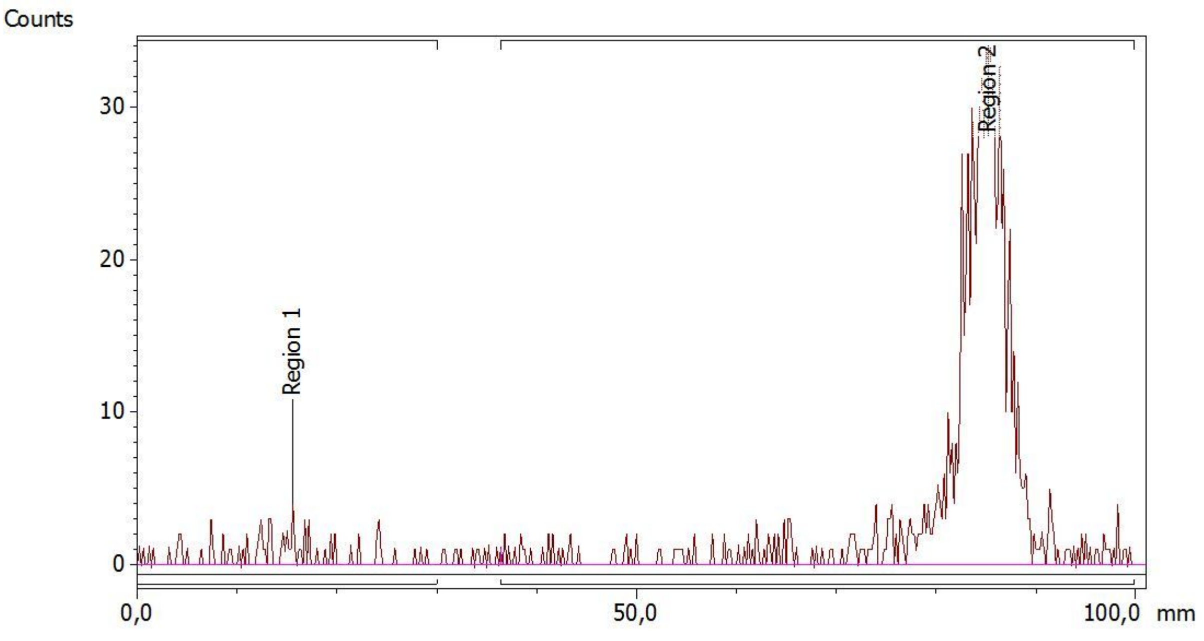

Figure S92: Radio-TLC chromatogram of [^{18}F]H_2N-Lys-(SiFA)SeFe-Gly-Tyr-OH ($2X_3$) at pH 5.5 and 90 °C, t = 90 min (flow agent: 60% MeCN/ 40% PBS (6/4 v/v) with 10% NaOAc in H_2O (2 M) and 1% TFA, stationary phase: TLC Silica gel 60 F254 from Merck Millipore).

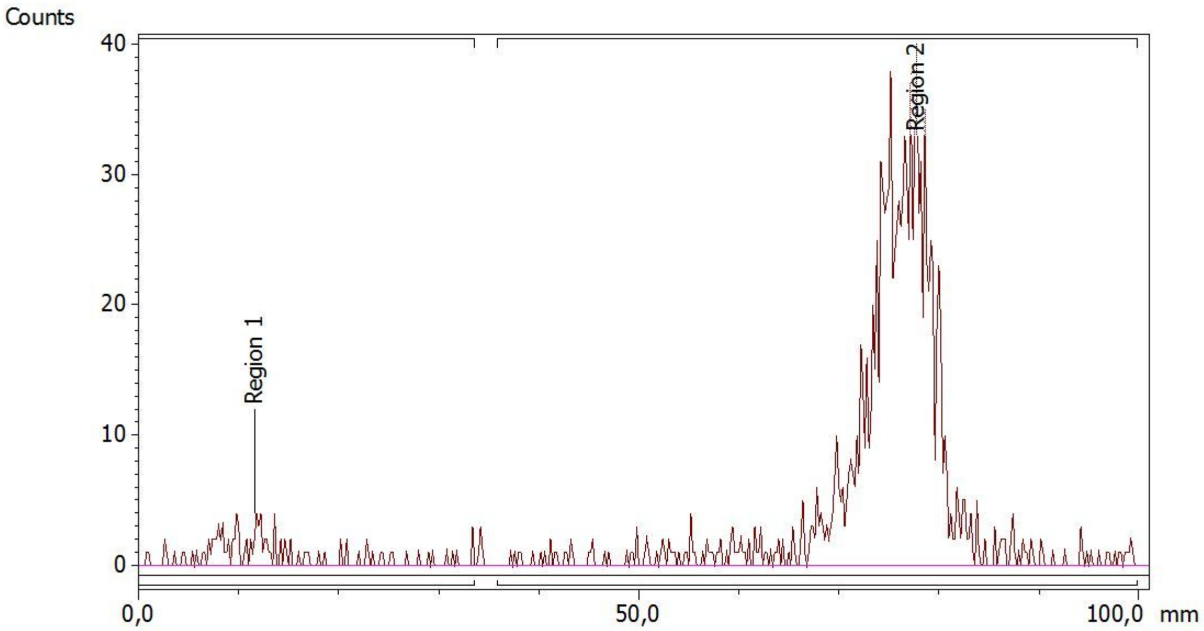

Figure S93: Radio-TLC chromatogram of [^{18}F]H_2N-Lys-(SiFA)SeFe-Gly-Tyr-OH ($2X_3$) at pH 5.5 and 90 °C, t = 120 min (flow agent: 60% MeCN/ 40% PBS (6/4 v/v) with 10% NaOAc in H_2O (2 M) and 1% TFA, stationary phase: TLC Silica gel 60 F254 from Merck Millipore).

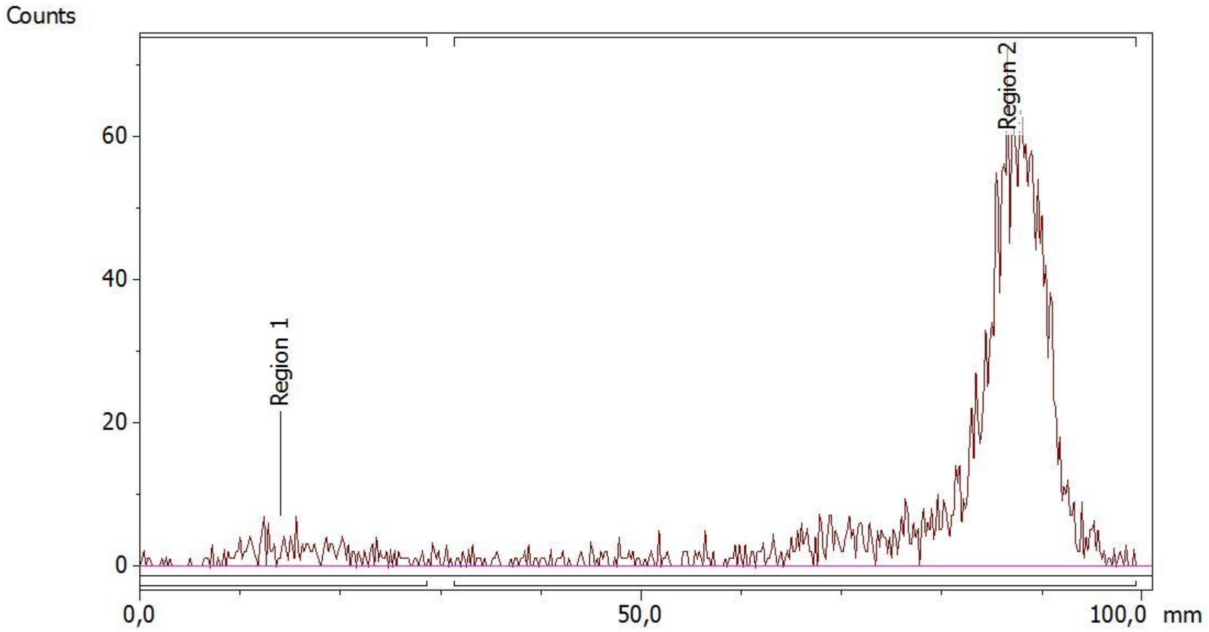

Figure S94*:* Radio-TLC chromatogram of [^{18}F]H_2N-Glu-(SiFA)SeFe-Gly-Tyr-OH ($3X_3$) at pH 5.5 and 90 °C, t = 0 min (flow agent: 60% MeCN/ 40% PBS (6/4 *v*/*v*) with 10% NaOAc in H_2O (2 M) and 1% TFA, stationary phase: TLC Silica gel 60 F254 from *Merck Millipore*).

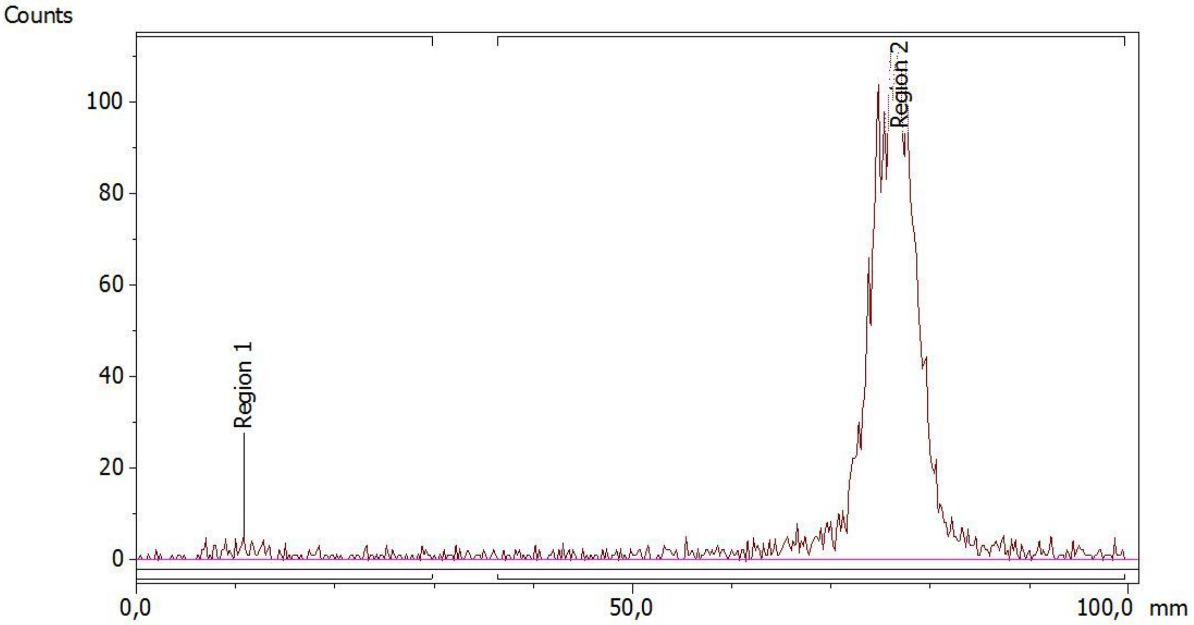

Figure S95*:* Radio-TLC chromatogram of [^{18}F]H_2N-Glu-(SiFA)SeFe-Gly-Tyr-OH ($3X_3$) at pH 5.5 and 90 °C, t = 30 min (flow agent: 60% MeCN/ 40% PBS (6/4 *v*/*v*) with 10% NaOAc in H_2O (2 M) and 1% TFA, stationary phase: TLC Silica gel 60 F254 from *Merck Millipore*).

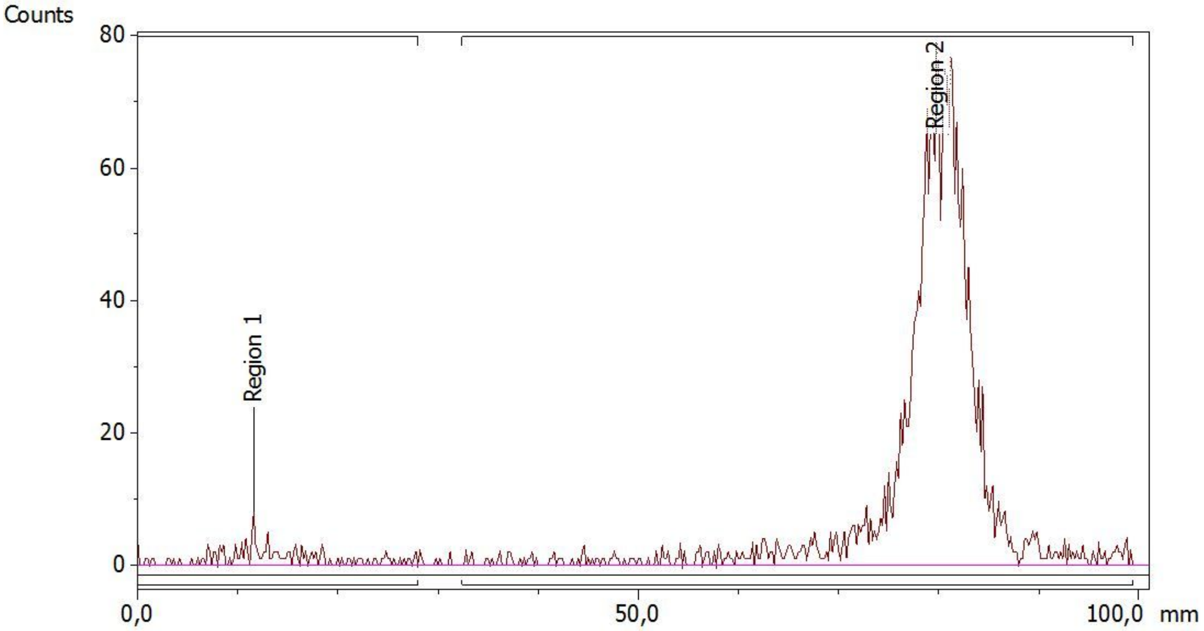

Figure S96*:* Radio-TLC chromatogram of [^{18}F]H_2N-Glu-(SiFA)SeFe-Gly-Tyr-OH ($3X_3$) at pH 5.5 and 90 °C, t = 60 min (flow agent: 60% MeCN/ 40% PBS (6/4 *v*/*v*) with 10% NaOAc in H_2O (2 M) and 1% TFA, stationary phase: TLC Silica gel 60 F254 from *Merck Millipore*).

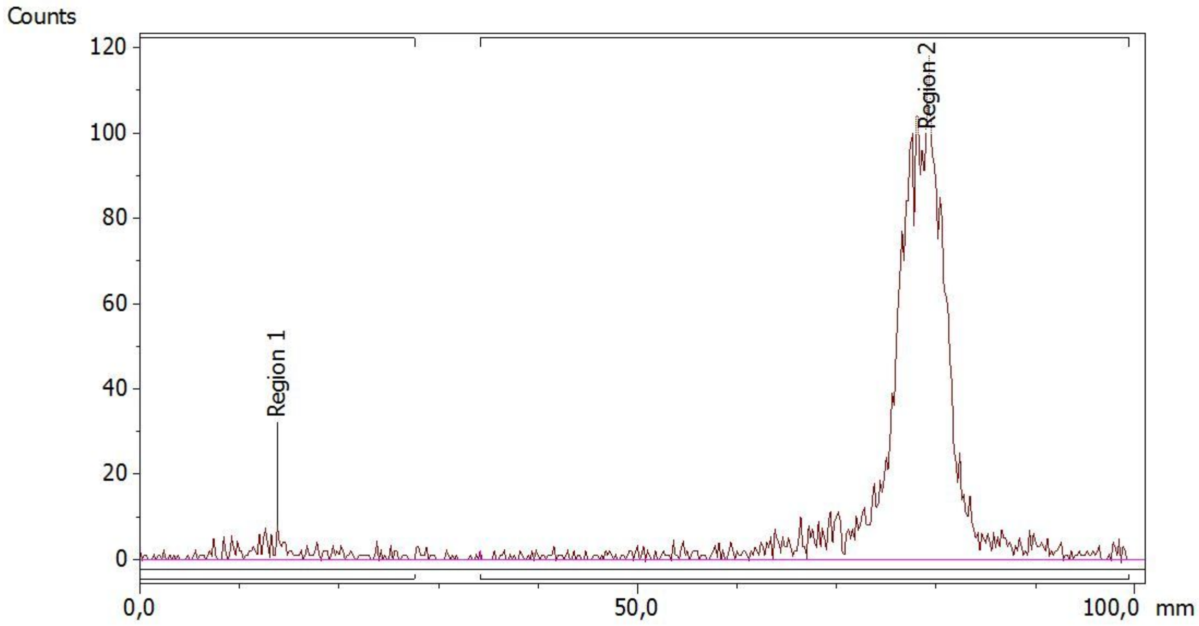

Figure S97: Radio-TLC chromatogram of [^{18}F]H$_2$N-Glu-(SiFA)SeFe-Gly-Tyr-OH (3X$_3$) at pH 5.5 and 90 °C, t = 90 min (flow agent: 60% MeCN/ 40% PBS (6/4 *v*/*v*) with 10% NaOAc in H_2O (2 M) and 1% TFA, stationary phase: TLC Silica gel 60 F254 from *Merck Millipore*).

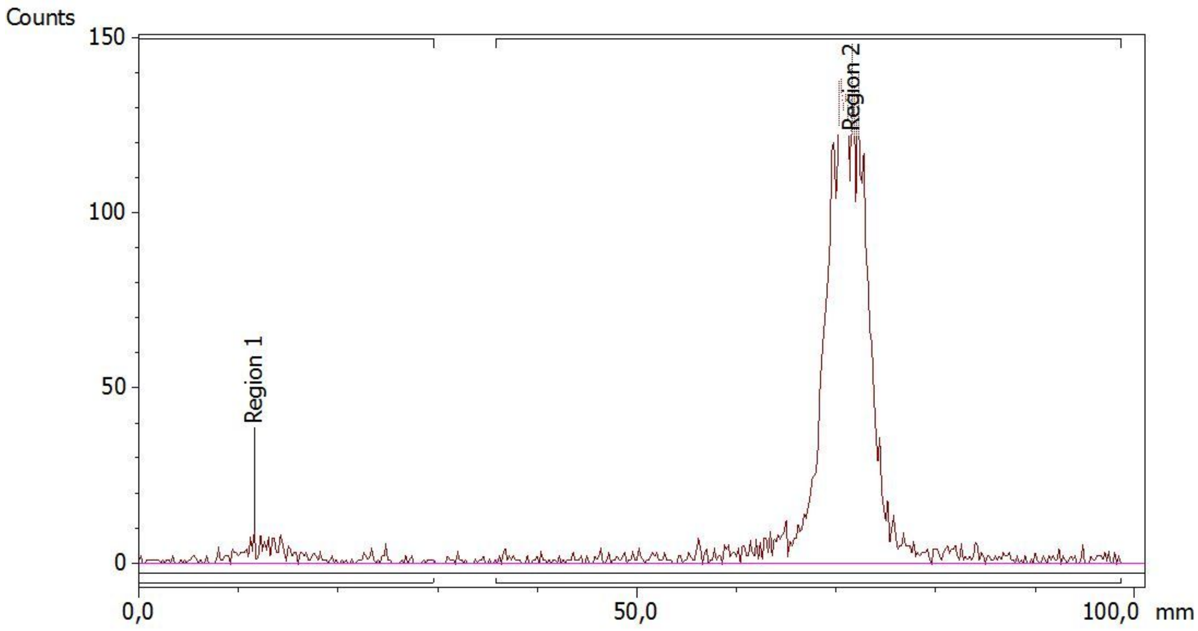

Figure S98: Radio-TLC chromatogram of [^{18}F]H$_2$N-Glu-(SiFA)SeFe-Gly-Tyr-OH (3X$_3$) at pH 5.5, 90 °C, t = 120 min (flow agent: 60% MeCN/ 40% PBS (6/4 *v*/*v*) with 10% NaOAc in H_2O (2 M) and 1% TFA, stationary phase: TLC Silica gel 60 F254 from *Merck Millipore*).

Stability towards reverse Isotopic exchange

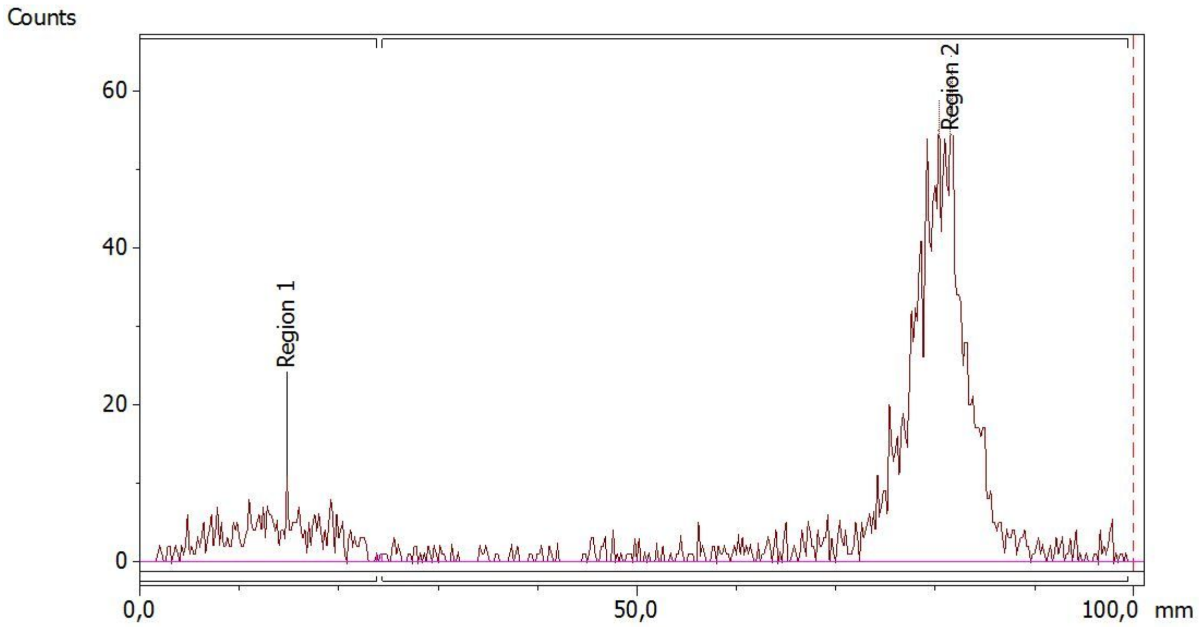

Figure S99: Radio-TLC chromatogram of [^{18}F]H_2N-(SiFA)SeFe-Gly-Lys-OH ($1X_1$) at pH 6.5, 1 mM aq. NaF, RT, t = 0 min (flow agent: 60% MeCN/ 40% PBS (6/4 v/v) with 10% NaOAc in H_2O (2 M) and 1% TFA, stationary phase: TLC Silica gel 60 F254 from Merck Millipore).

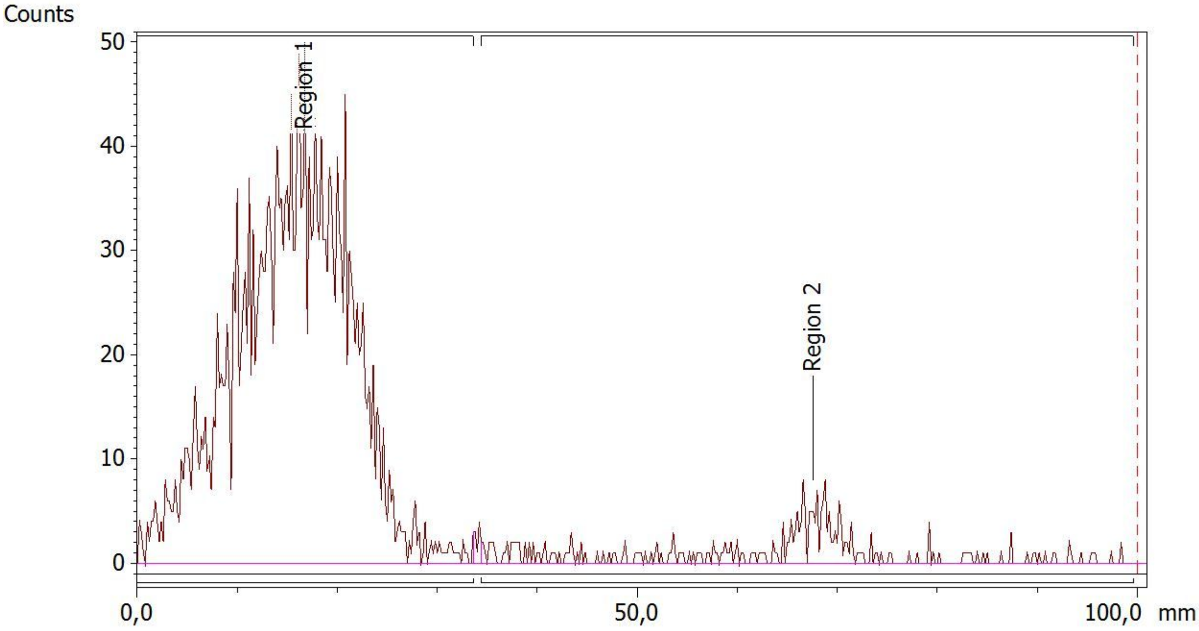

Figure S100: Radio-TLC chromatogram of [^{18}F]H_2N-(SiFA)SeFe-Gly-Lys-OH ($1X_1$) at pH 6.5, 1 mM aq. NaF, RT, t = 30 min (flow agent: 60% MeCN/ 40% PBS (6/4 v/v) with 10% NaOAc in H_2O (2 M) and 1% TFA, stationary phase: TLC Silica gel 60 F254 from Merck Millipore).

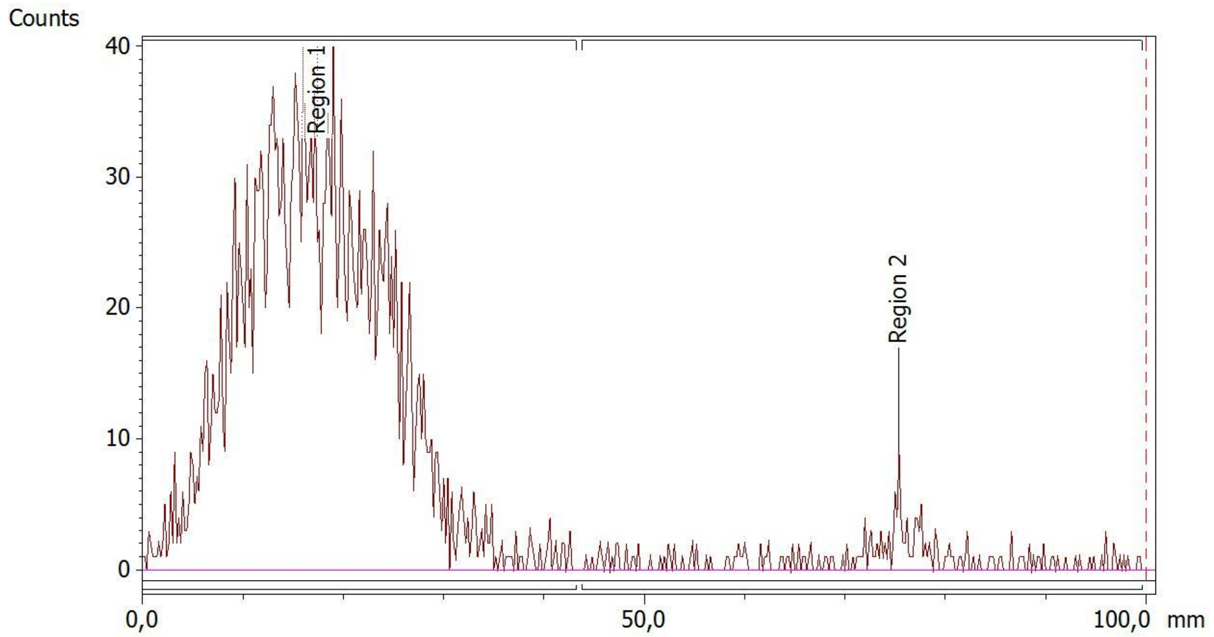

Figure S101: Radio-TLC chromatogram of [^{18}F]H_2N-(SiFA)SeFe-Gly-Lys-OH ($1X_1$) at pH 6.5, 1 mM aq. NaF, RT, t = 60 min (flow agent: 60% MeCN/ 40% PBS (6/4 v/v) with 10% NaOAc in H_2O (2 M) and 1% TFA, stationary phase: TLC Silica gel 60 F254 from Merck Millipore).

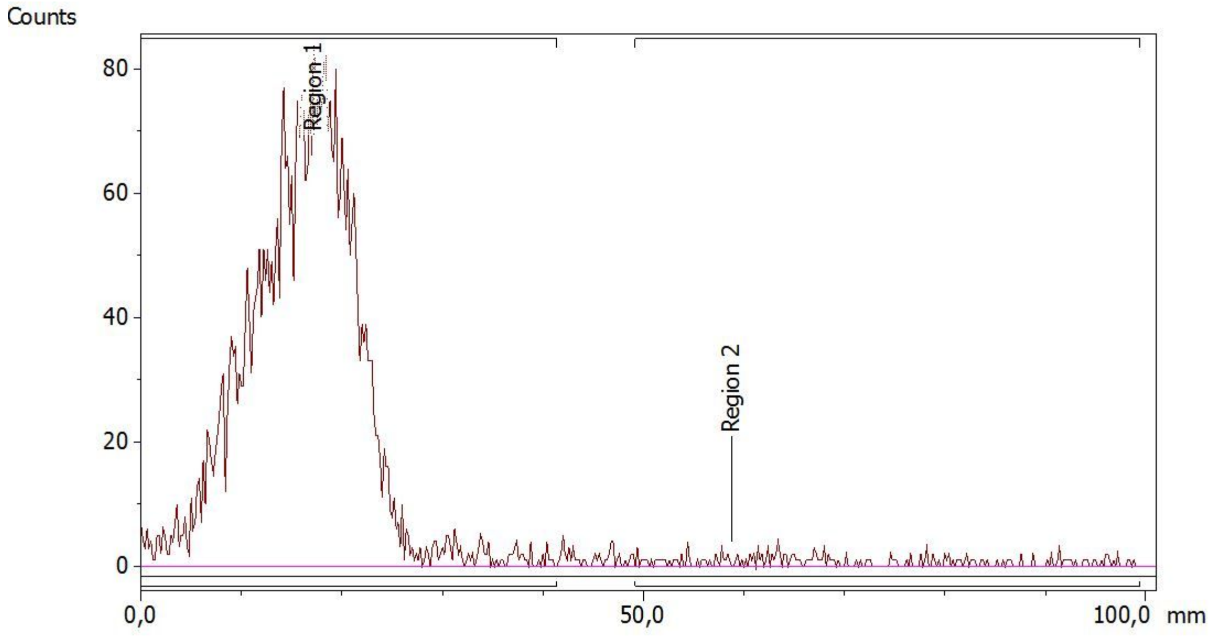

Figure S102: Radio-TLC chromatogram of [^{18}F]H_2N-(SiFA)SeFe-Gly-Lys-OH ($1X_1$) at pH 6.5, 1 mM aq. NaF, RT, t = 90 min (flow agent: 60% MeCN/ 40% PBS (6/4 v/v) with 10% NaOAc in H_2O (2 M) and 1% TFA, stationary phase: TLC Silica gel 60 F254 from Merck Millipore).

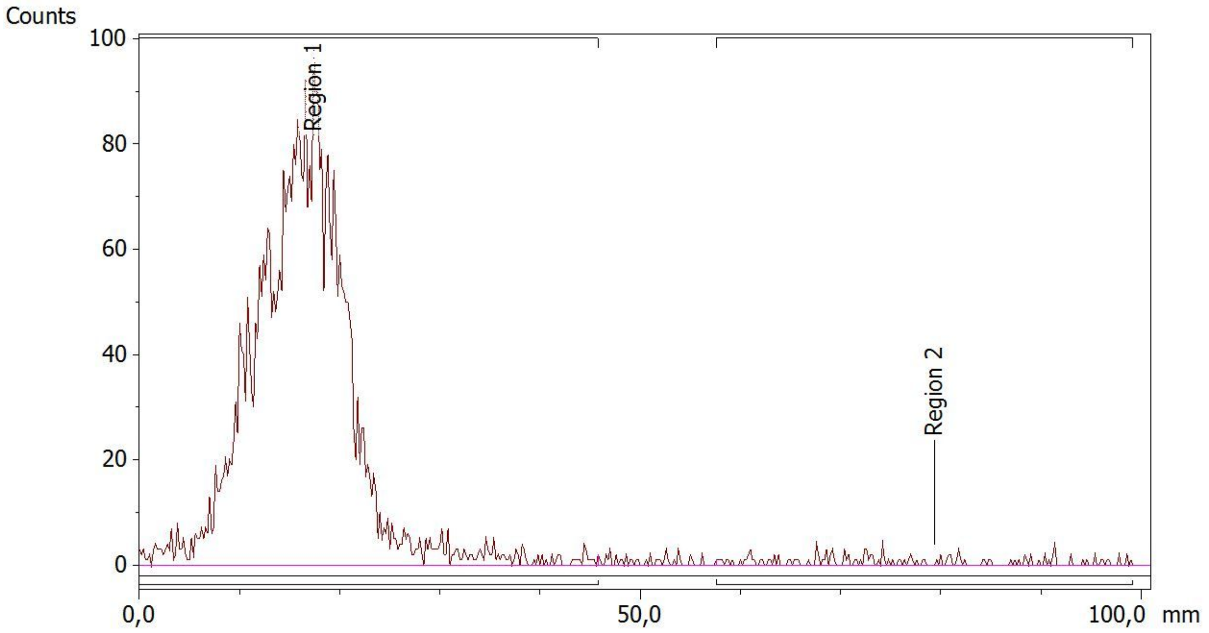

Figure S103: Radio-TLC chromatogram of [^{18}F]H_2N-(SiFA)SeFe-Gly-Lys-OH ($1X_1$) at pH 6.5, 1 mM aq. NaF, RT, t = 120 min (flow agent: 60% MeCN/ 40% PBS (6/4 v/v) with 10% NaOAc in H_2O (2 M) and 1% TFA, stationary phase: TLC Silica gel 60 F254 from Merck Millipore).

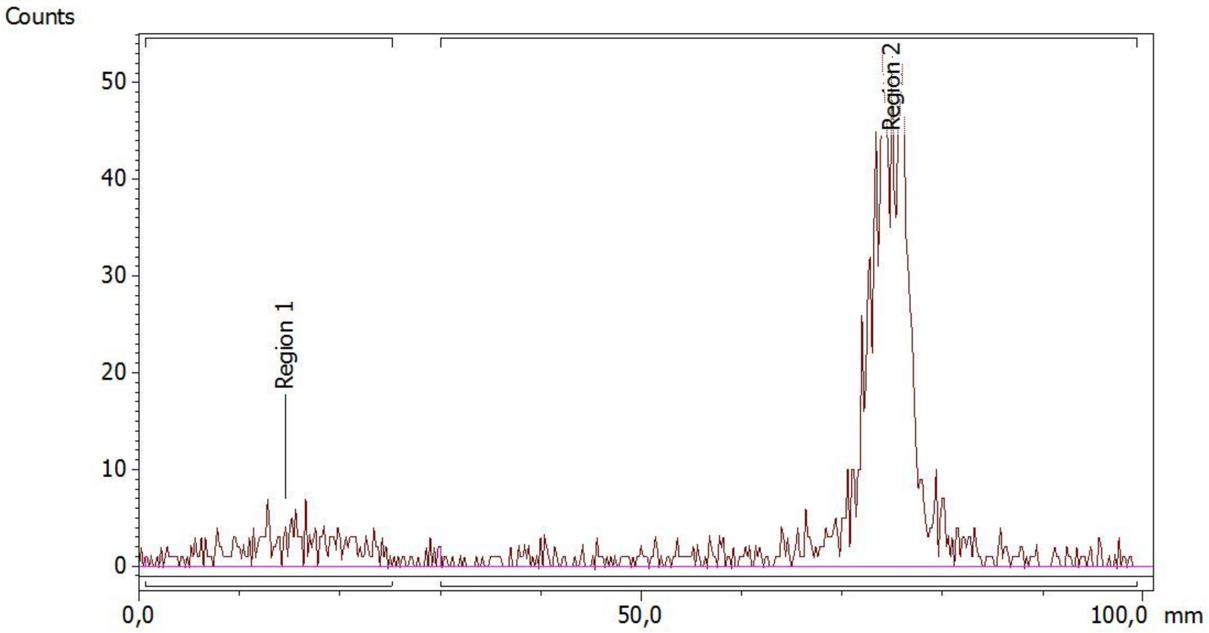

Figure S104: Radio-TLC chromatogram of [^{18}F]H_2N-Lys-(SiFA)SeFe-Gly-Lys-OH ($2X_1$) at pH 6.5, 1 mM aq. NaF, RT, t = 0 min (flow agent: 60% MeCN/ 40% PBS (6/4 v/v) with 10% NaOAc in H_2O (2 M) and 1% TFA, stationary phase: TLC Silica gel 60 F254 from Merck Millipore).

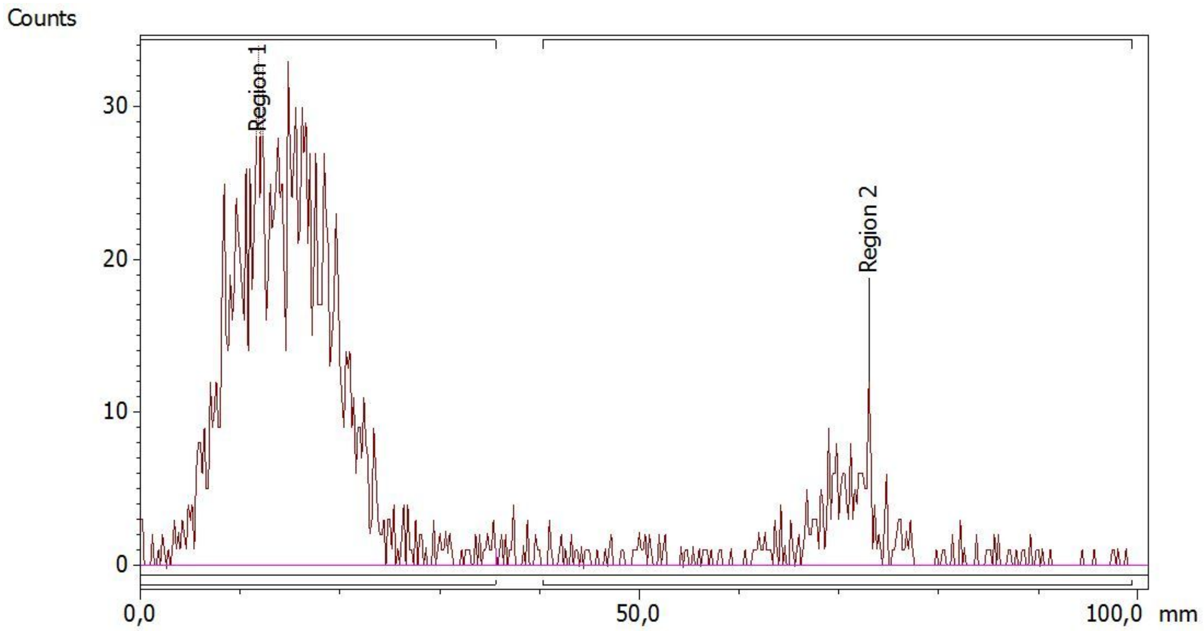

Figure S105: Radio-TLC chromatogram of [^{18}F]H$_2$N-Lys-(SiFA)SeFe-Gly-Lys-OH (2X$_1$) at pH 6.5, 1 mM aq. NaF, RT, t = 30 min (flow agent: 60% MeCN/ 40% PBS (6/4 v/v) with 10% NaOAc in H_2O (2 M) and 1% TFA, stationary phase: TLC Silica gel 60 F254 from Merck Millipore).

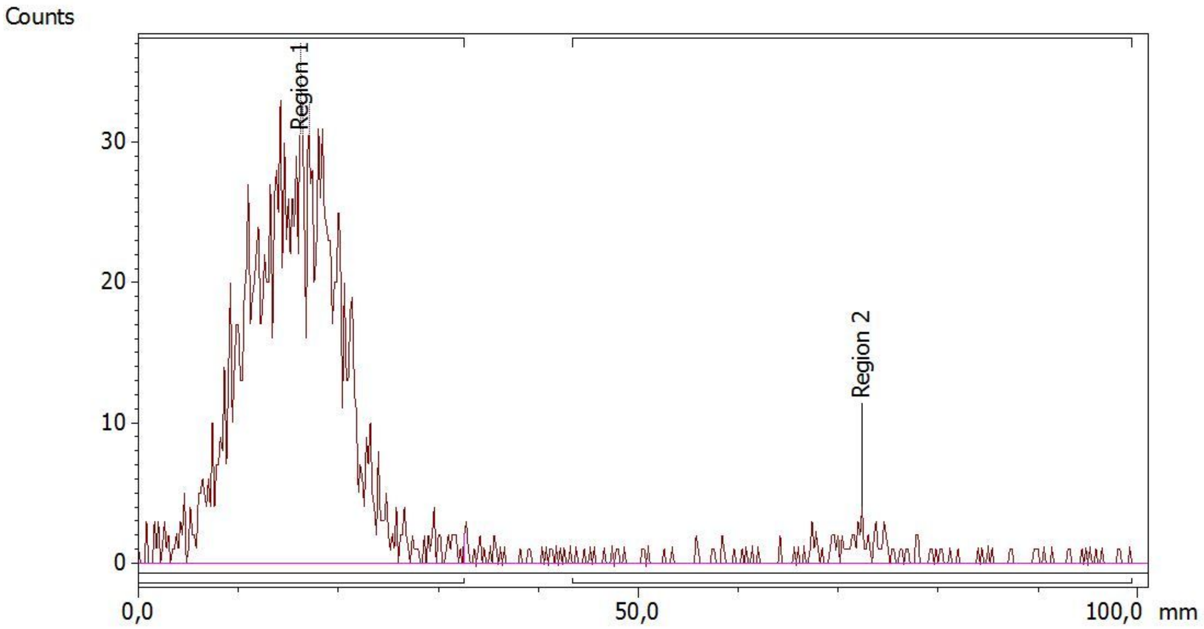

Figure S106: Radio-TLC chromatogram of [^{18}F]H$_2$N-Lys-(SiFA)SeFe-Gly-Lys-OH (2X$_1$) at pH 6.5, 1 mM aq. NaF, RT, t = 60 min (flow agent: 60% MeCN/ 40% PBS (6/4 v/v) with 10% NaOAc in H_2O (2 M) and 1% TFA, stationary phase: TLC Silica gel 60 F254 from Merck Millipore).

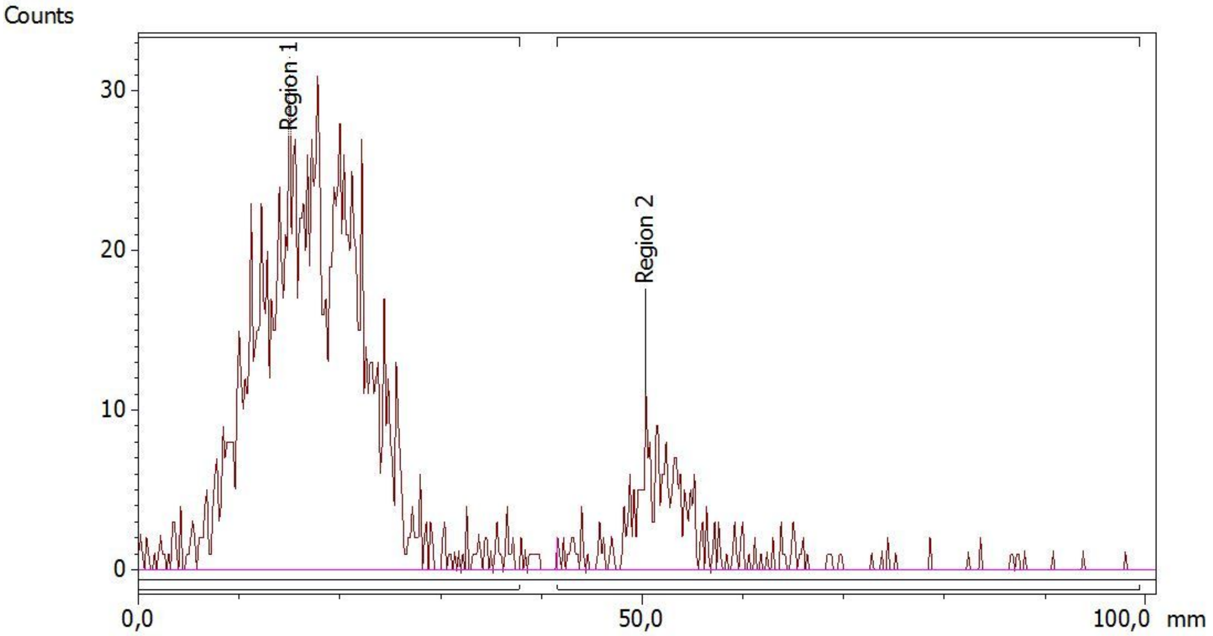

Figure S107: Radio-TLC chromatogram of [^{18}F]H_2N-Lys-(SiFA)SeFe-Gly-Lys-OH (2X_1) at pH 6.5, 1 mM aq. NaF, RT, t = 90 min (flow agent: 60% MeCN/ 40% PBS (6/4 v/v) with 10% NaOAc in H_2O (2 M) and 1% TFA, stationary phase: TLC Silica gel 60 F254 from Merck Millipore).

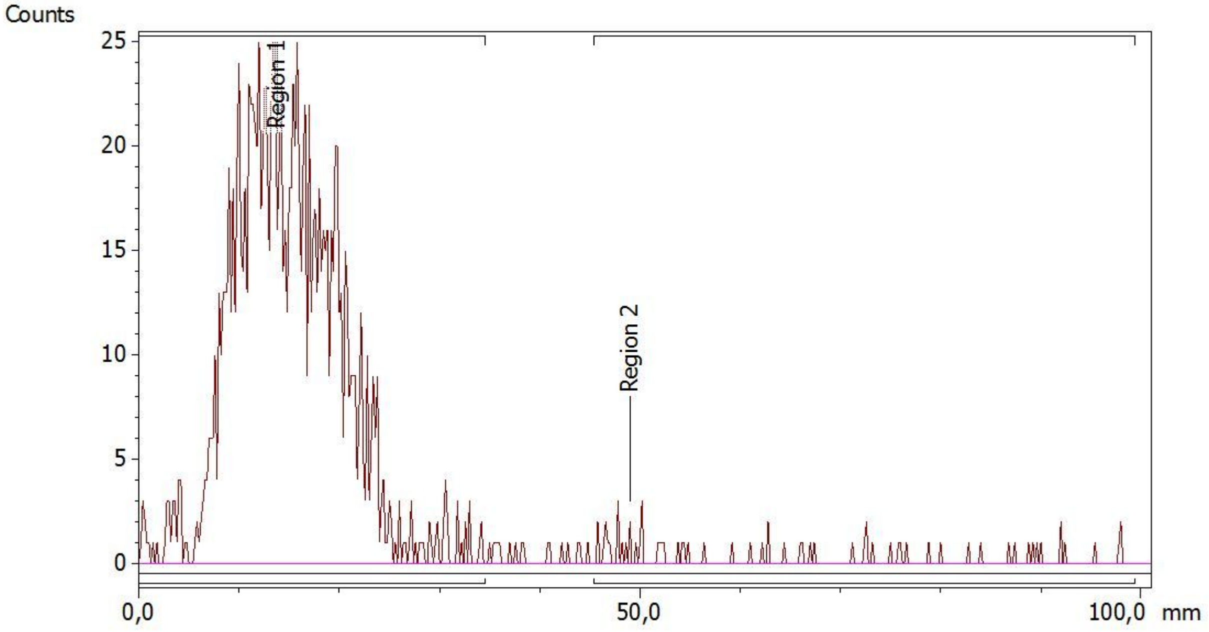

Figure S108: Radio-TLC chromatogram of [^{18}F]H_2N-Lys-(SiFA)SeFe-Gly-Lys-OH (2X_1) at pH 6.5, 1 mM aq. NaF, RT, t = 120 min (flow agent: 60% MeCN/ 40% PBS (6/4 v/v) with 10% NaOAc in H_2O (2 M) and 1% TFA, stationary phase: TLC Silica gel 60 F254 from Merck Millipore).

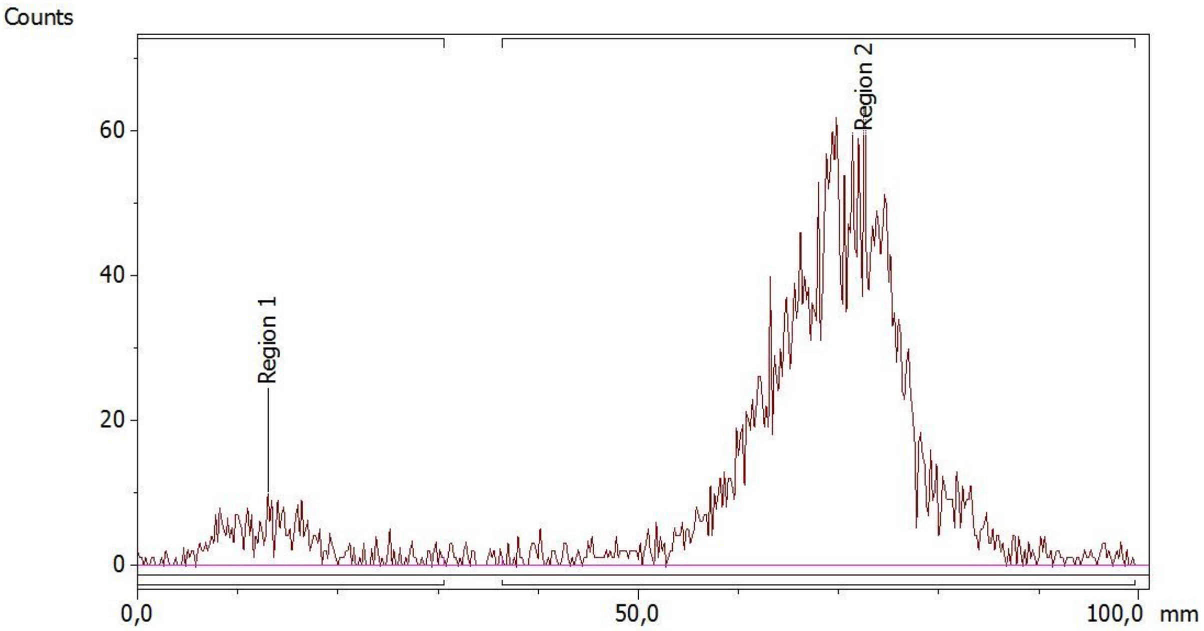

Figure S109: Radio-TLC chromatogram of [^{18}F]H_2N-Glu-(SiFA)SeFe-Gly-Lys-OH ($3X_1$) at pH 6.5, 1 mM aq. NaF, RT, t = 0 min (flow agent: 60% MeCN/ 40% PBS (6/4 v/v) with 10% NaOAc in H_2O (2 M) and 1% TFA, stationary phase: TLC Silica gel 60 F254 from Merck Millipore).

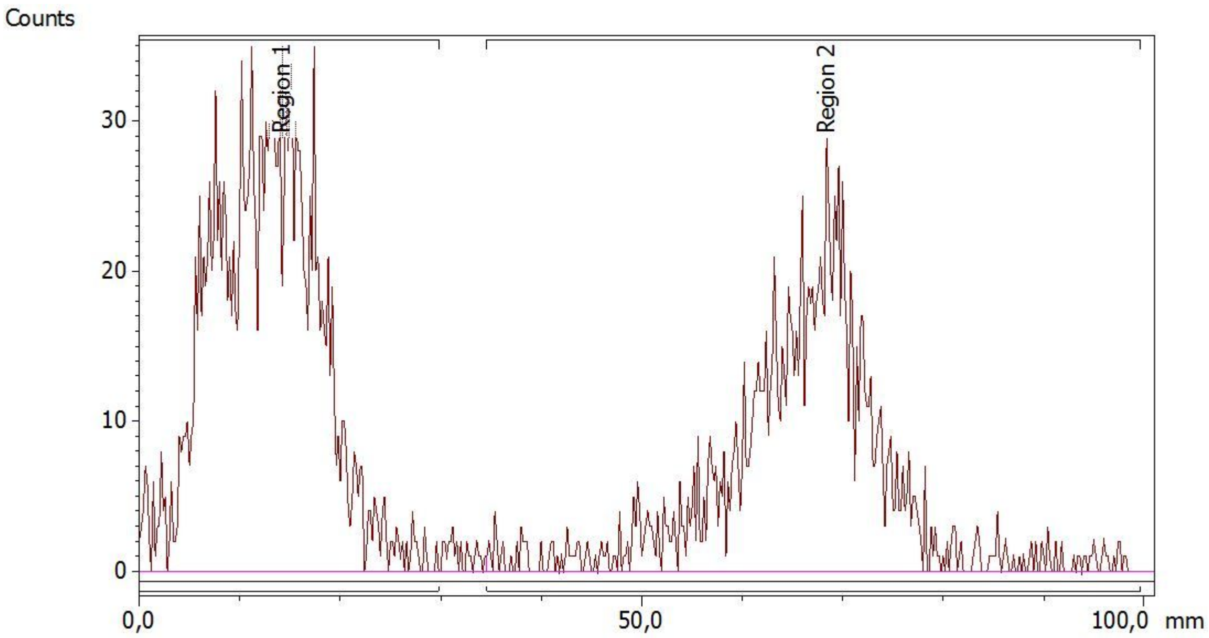

Figure S110: Radio-TLC chromatogram of [^{18}F]H_2N-Glu-(SiFA)SeFe-Gly-Lys-OH ($3X_1$) at pH 6.5, 1 mM aq. NaF, RT, t = 30 min (flow agent: 60% MeCN/ 40% PBS (6/4 v/v) with 10% NaOAc in H_2O (2 M) and 1% TFA, stationary phase: TLC Silica gel 60 F254 from Merck Millipore).

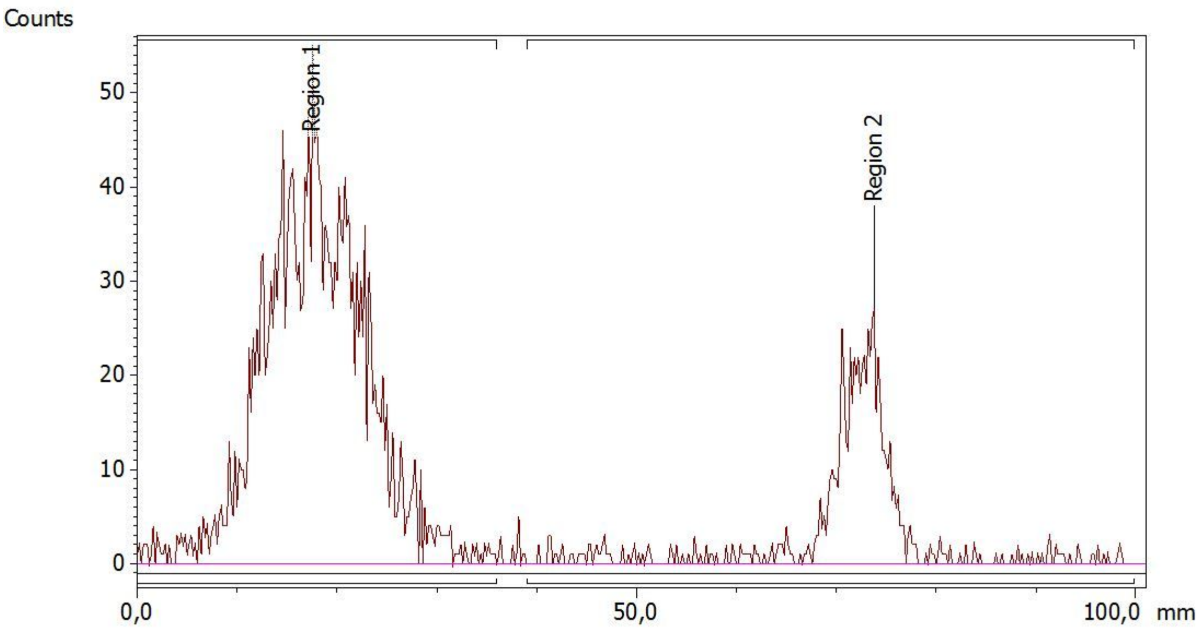

Figure S111: Radio-TLC chromatogram of [18F]H_2N-Glu-(SiFA)SeFe-Gly-Lys-OH ($3X_1$) at pH 6.5, 1 mM aq. NaF, RT, t = 60 min (flow agent: 60% MeCN/ 40% PBS (6/4 v/v) with 10% NaOAc in H_2O (2 M) and 1% TFA, stationary phase: TLC Silica gel 60 F254 from Merck Millipore).

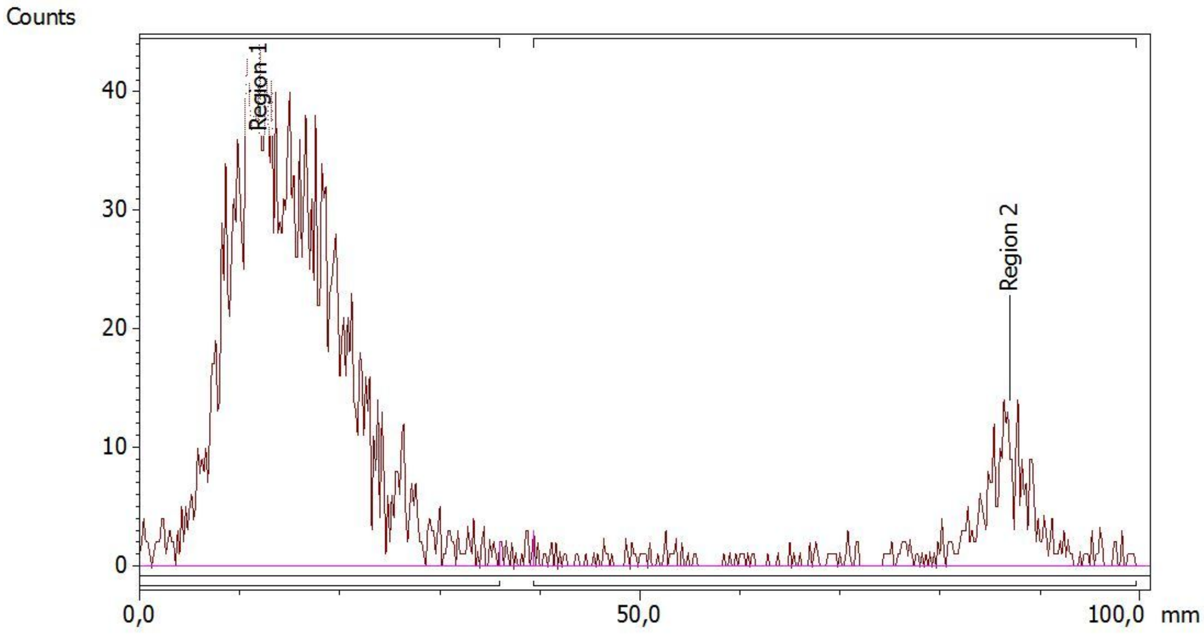

Figure S112: Radio-TLC chromatogram of [18F]H_2N-Glu-(SiFA)SeFe-Gly-Lys-OH ($3X_1$) at pH 6.5, 1 mM aq. NaF, RT, t = 90 min (flow agent: 60% MeCN/ 40% PBS (6/4 v/v) with 10% NaOAc in H_2O (2 M) and 1% TFA, stationary phase: TLC Silica gel 60 F254 from Merck Millipore).

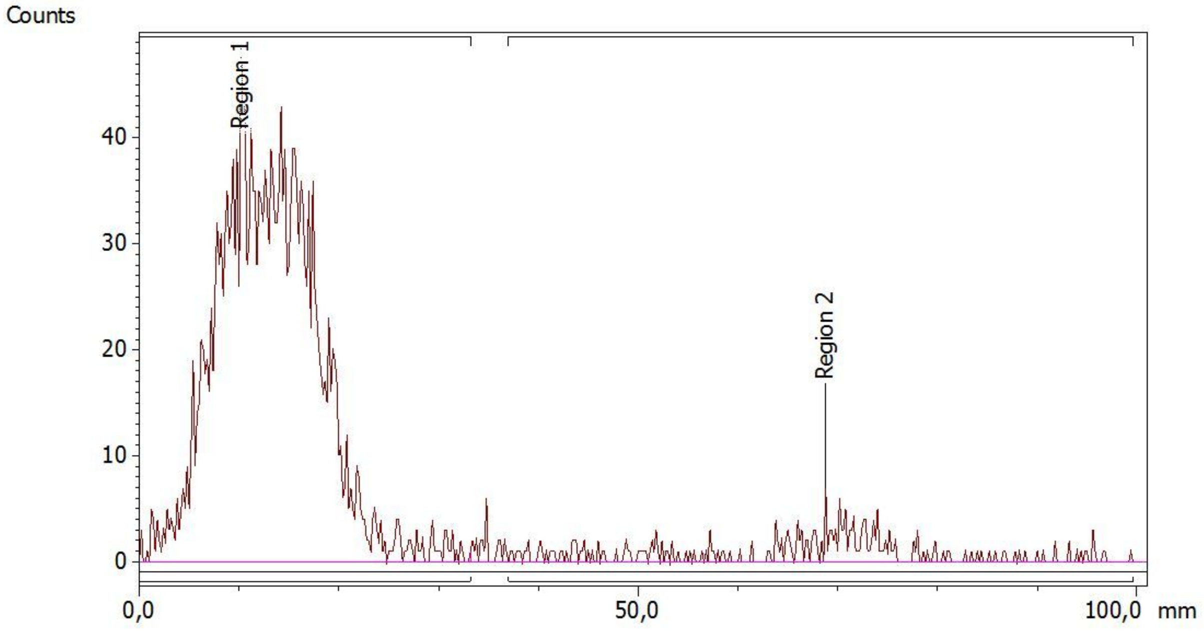

Figure S113: Radio-TLC chromatogram of [^{18}F]H_2N-Glu-(SiFA)SeFe-Gly-Lys-OH ($3X_1$) at pH 6.5, 1 mM aq. NaF, RT, t = 120 min (flow agent: 60% MeCN/ 40% PBS (6/4 v/v) with 10% NaOAc in H_2O (2 M) and 1% TFA, stationary phase: TLC Silica gel 60 F254 from Merck Millipore).

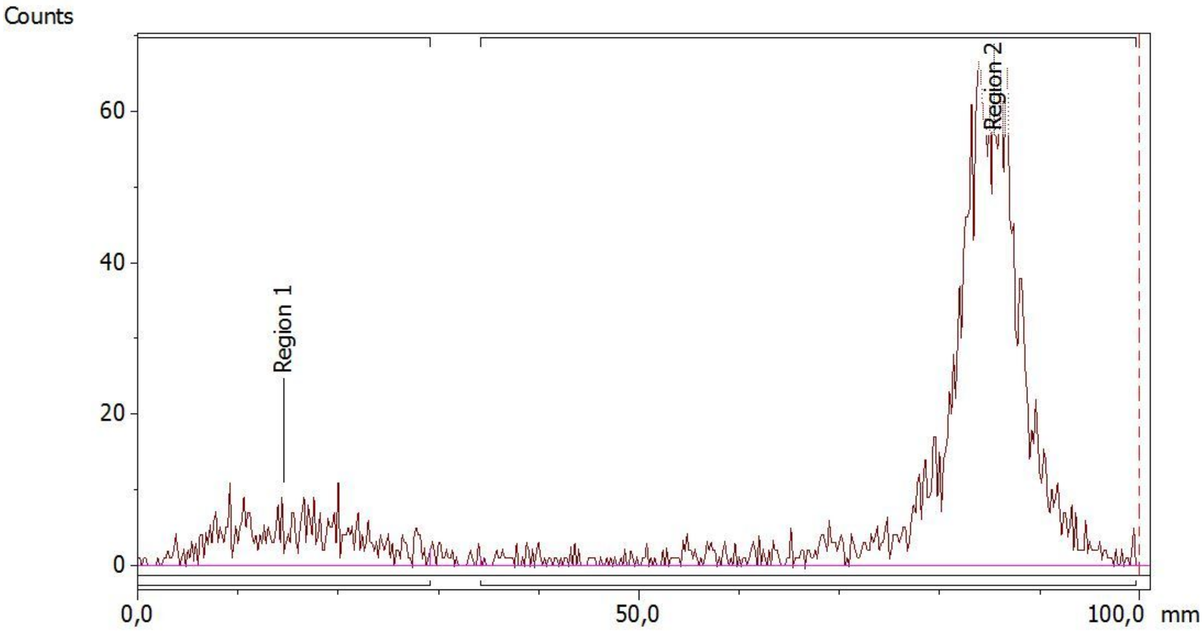

Figure S114: Radio-TLC chromatogram of [^{18}F]H_2N-(SiFA)SeFe-Gly-Asp-OH ($1X_2$) at pH 6.5, 1 mM aq. NaF, RT, t = 0 min (flow agent: 60% MeCN/ 40% PBS (6/4 v/v) with 10% NaOAc in H_2O (2 M) and 1% TFA, stationary phase: TLC Silica gel 60 F254 from Merck Millipore).

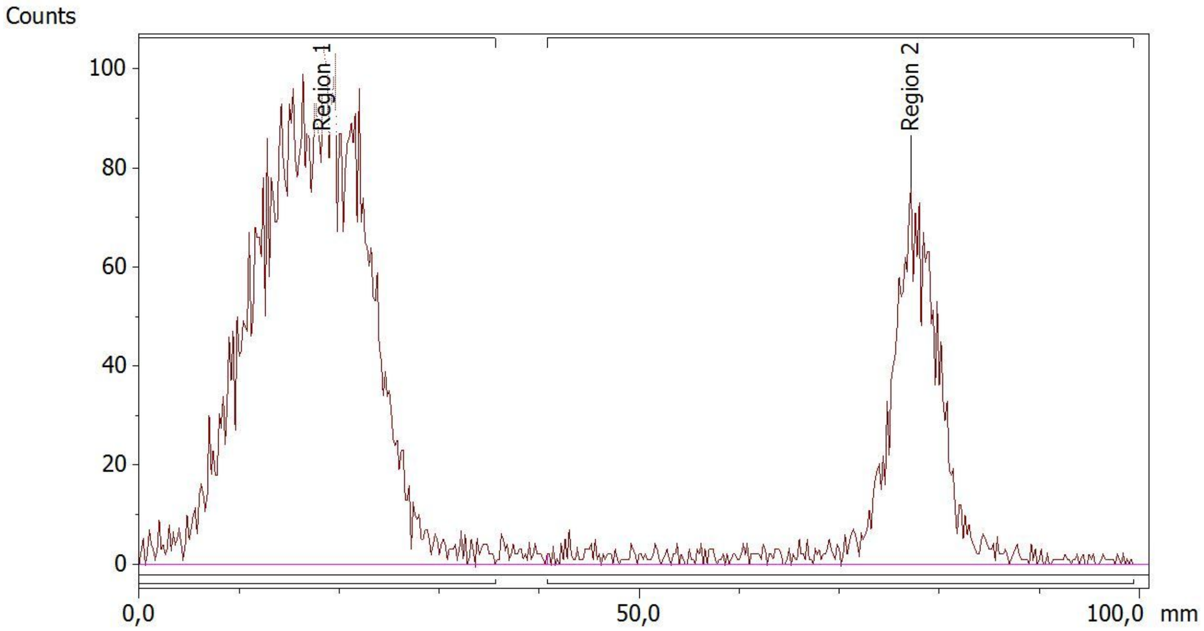

Figure S115: Radio-TLC chromatogram of [^{18}F]H_2N-(SiFA)SeFe-Gly-Asp-OH (1X_2) at pH 6.5, 1 mM aq. NaF, RT, t = 30 min (flow agent: 60% MeCN/ 40% PBS (6/4 v/v) with 10% NaOAc in H_2O (2 M) and 1% TFA, stationary phase: TLC Silica gel 60 F254 from Merck Millipore).

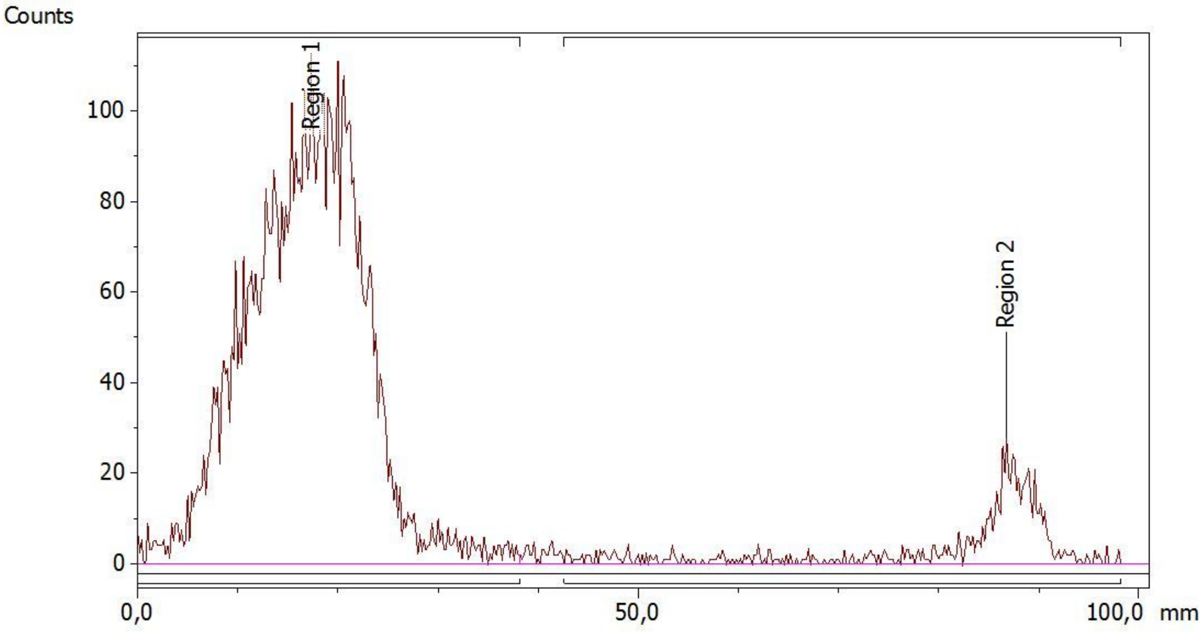

Figure S116: Radio-TLC chromatogram of [^{18}F]H_2N-(SiFA)SeFe-Gly-Asp-OH (1X_2) at pH 6.5, 1 mM aq. NaF, RT, t = 60 min (flow agent: 60% MeCN/ 40% PBS (6/4 v/v) with 10% NaOAc in H_2O (2 M) and 1% TFA, stationary phase: TLC Silica gel 60 F254 from Merck Millipore).

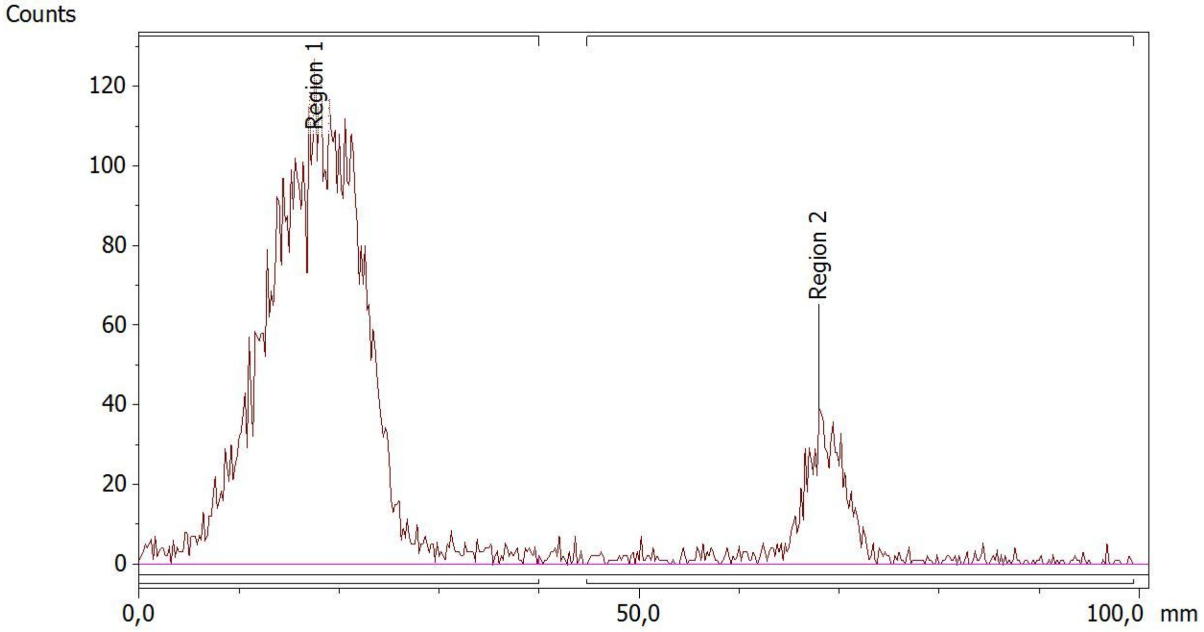

Figure S117: Radio-TLC chromatogram of [^{18}F]H_2N-(SiFA)SeFe-Gly-Asp-OH ($1X_2$) at pH 6.5, 1 mM aq. NaF, RT, t = 90 min (flow agent: 60% MeCN/ 40% PBS (6/4 v/v) with 10% NaOAc in H_2O (2 M) and 1% TFA, stationary phase: TLC Silica gel 60 F254 from Merck Millipore).

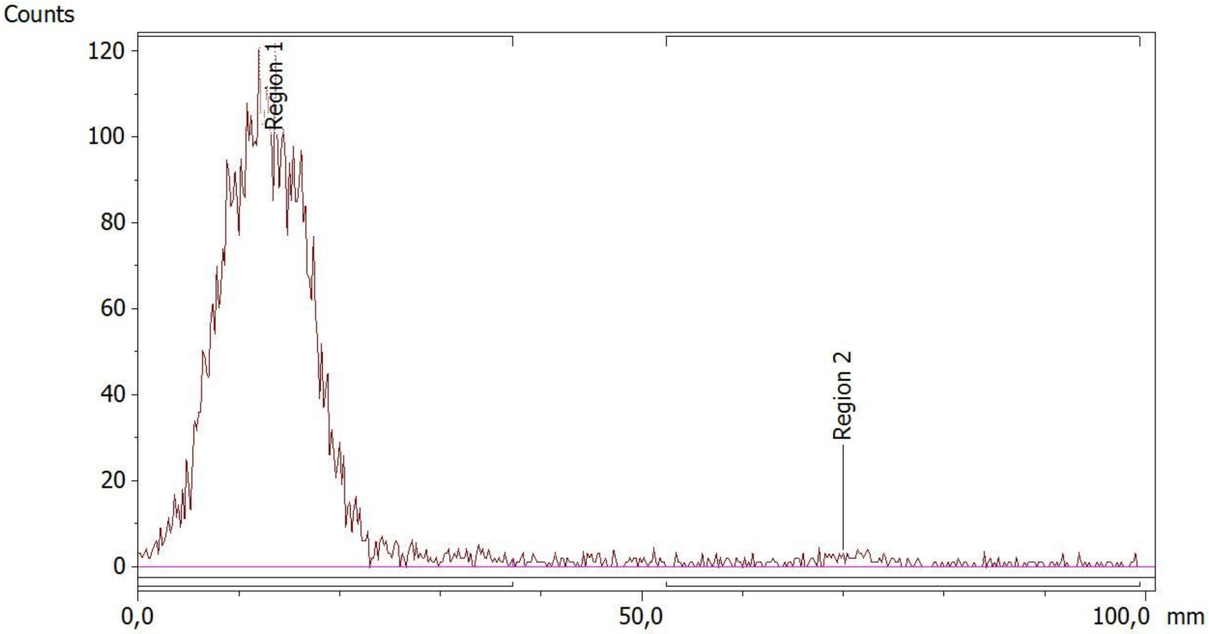

Figure S118: Radio-TLC chromatogram of [^{18}F]H_2N-(SiFA)SeFe-Gly-Asp-OH ($1X_2$) at pH 6.5, 1 mM aq. NaF, RT, t = 120 min (flow agent: 60% MeCN/ 40% PBS (6/4 v/v) with 10% NaOAc in H_2O (2 M) and 1% TFA, stationary phase: TLC Silica gel 60 F254 from Merck Millipore).

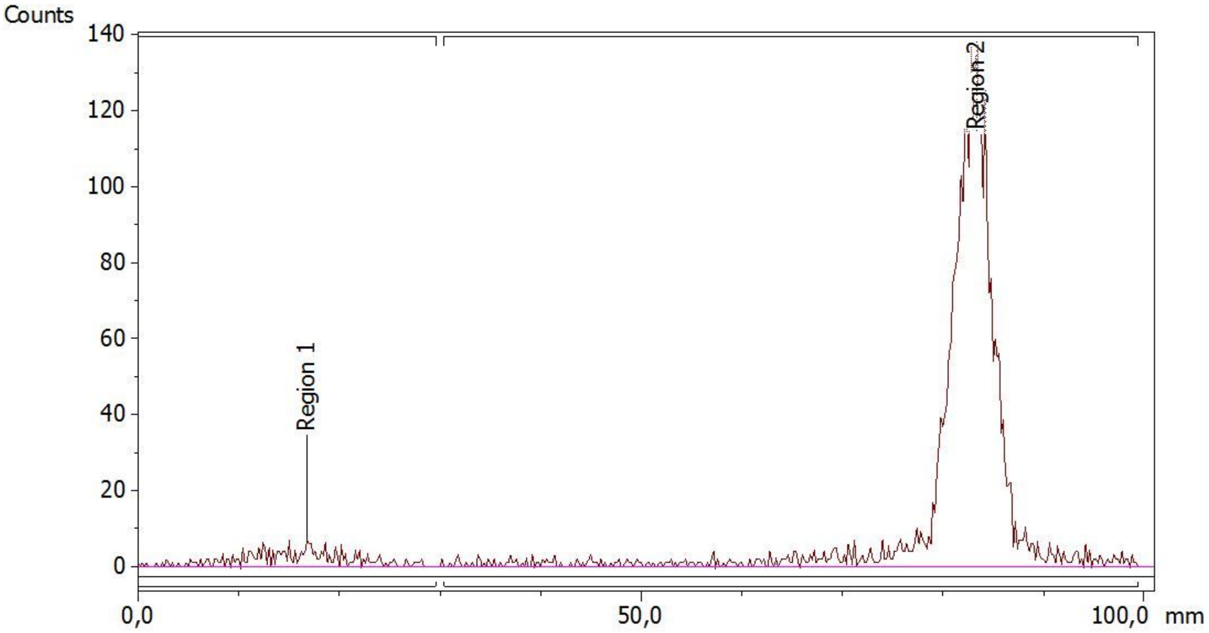

Figure S119: Radio-TLC chromatogram of [^{18}F]H_2N-Lys-(SiFA)SeFe-Gly-Asp-OH ($2X_2$) at pH 6.5, 1 mM aq. NaF, RT, t = 0 min (flow agent: 60% MeCN/ 40% PBS (6/4 v/v) with 10% NaOAc in H_2O (2 M) and 1% TFA, stationary phase: TLC Silica gel 60 F254 from Merck Millipore).

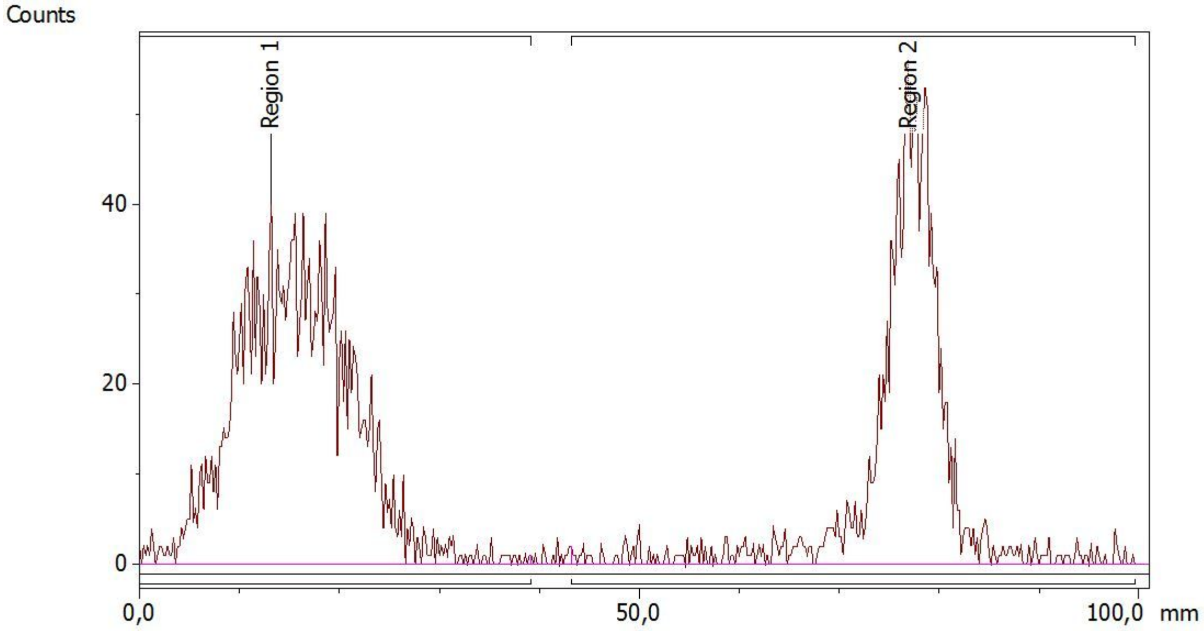

Figure S120: Radio-TLC chromatogram of [^{18}F]H_2N-Lys-(SiFA)SeFe-Gly-Asp-OH ($2X_2$) at pH 6.5, 1 mM aq. NaF, RT, t = 30 min (flow agent: 60% MeCN/ 40% PBS (6/4 v/v) with 10% NaOAc in H_2O (2 M) and 1% TFA, stationary phase: TLC Silica gel 60 F254 from Merck Millipore).

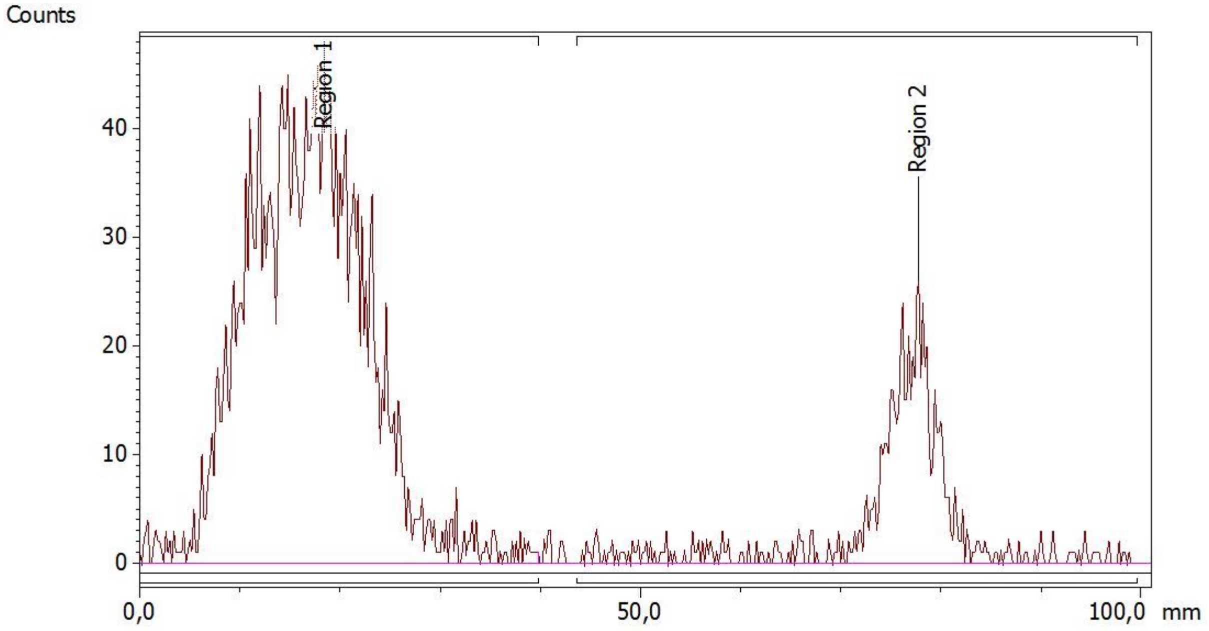

Figure S121: Radio-TLC chromatogram of [^{18}F]H$_2$N-Lys-(SiFA)SeFe-Gly-Asp-OH (2X$_2$) at pH 6.5, 1 mM aq. NaF, RT, t = 60 min (flow agent: 60% MeCN/ 40% PBS (6/4 v/v) with 10% NaOAc in H_2O (2 M) and 1% TFA, stationary phase: TLC Silica gel 60 F254 from Merck Millipore).

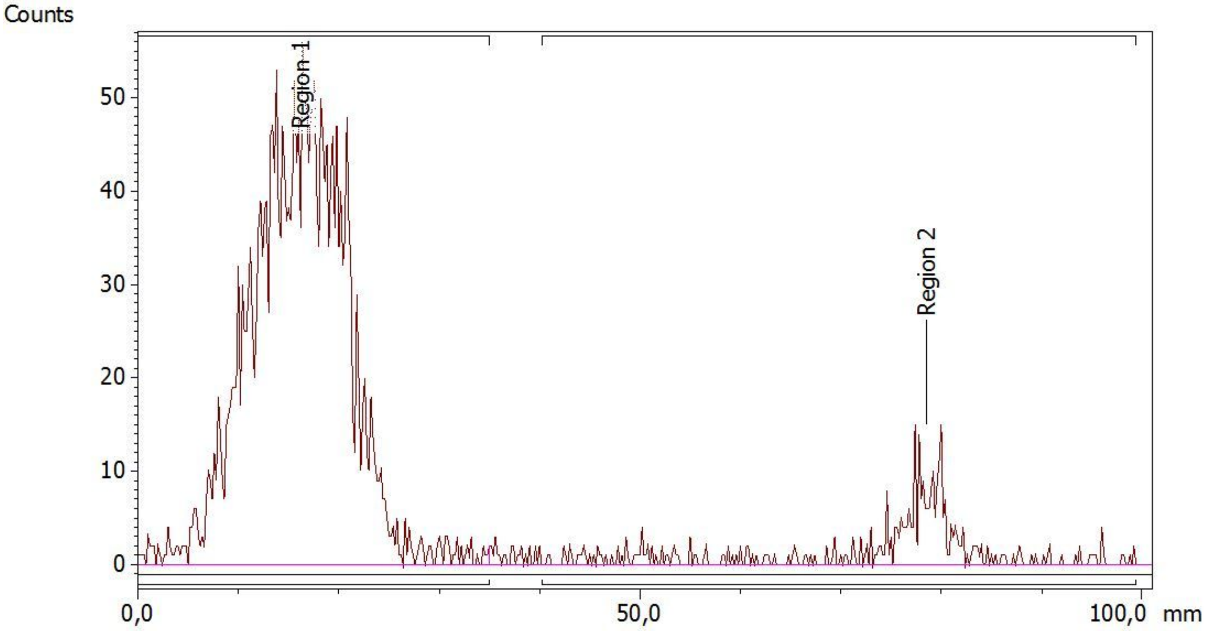

Figure S122: Radio-TLC chromatogram of [^{18}F]H$_2$N-Lys-(SiFA)SeFe-Gly-Asp-OH (2X$_2$) at pH 6.5, 1 mM aq. NaF, RT, t = 90 min (flow agent: 60% MeCN/ 40% PBS (6/4 v/v) with 10% NaOAc in H_2O (2 M) and 1% TFA, stationary phase: TLC Silica gel 60 F254 from Merck Millipore).

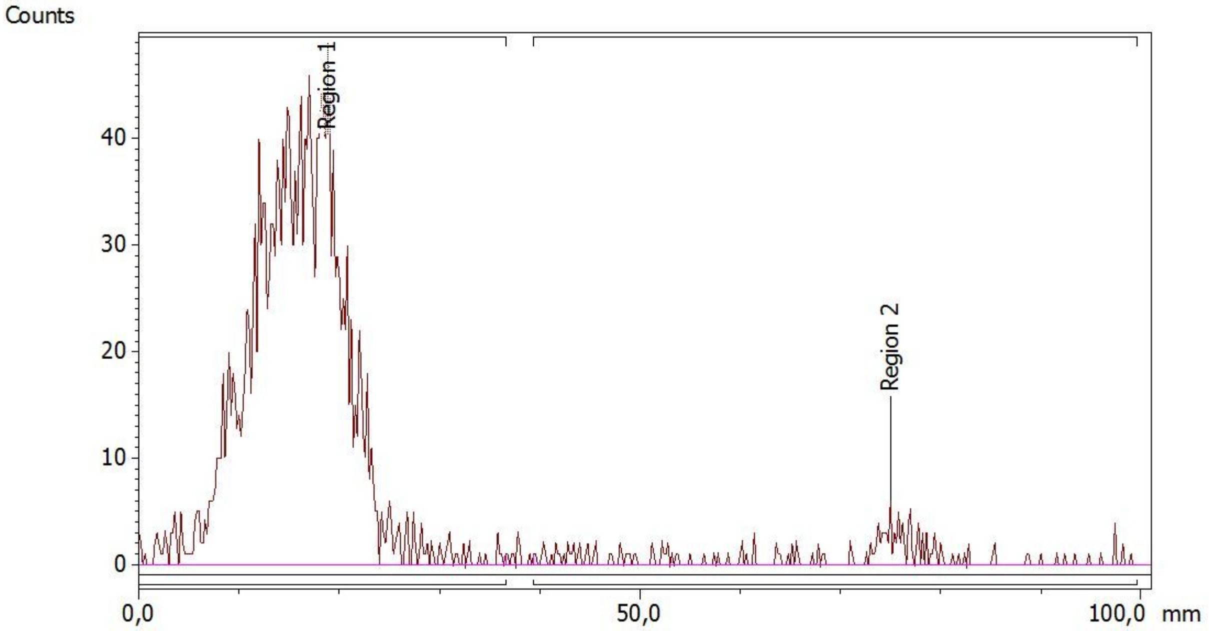

Figure S123: Radio-TLC chromatogram of [^{18}F]H_2N-Lys-(SiFA)SeFe-Gly-Asp-OH (2X_2) at pH 6.5, 1 mM aq. NaF, RT, t = 120 min (flow agent: 60% MeCN/ 40% PBS (6/4 v/v) with 10% NaOAc in H_2O (2 M) and 1% TFA, stationary phase: TLC Silica gel 60 F254 from Merck Millipore).

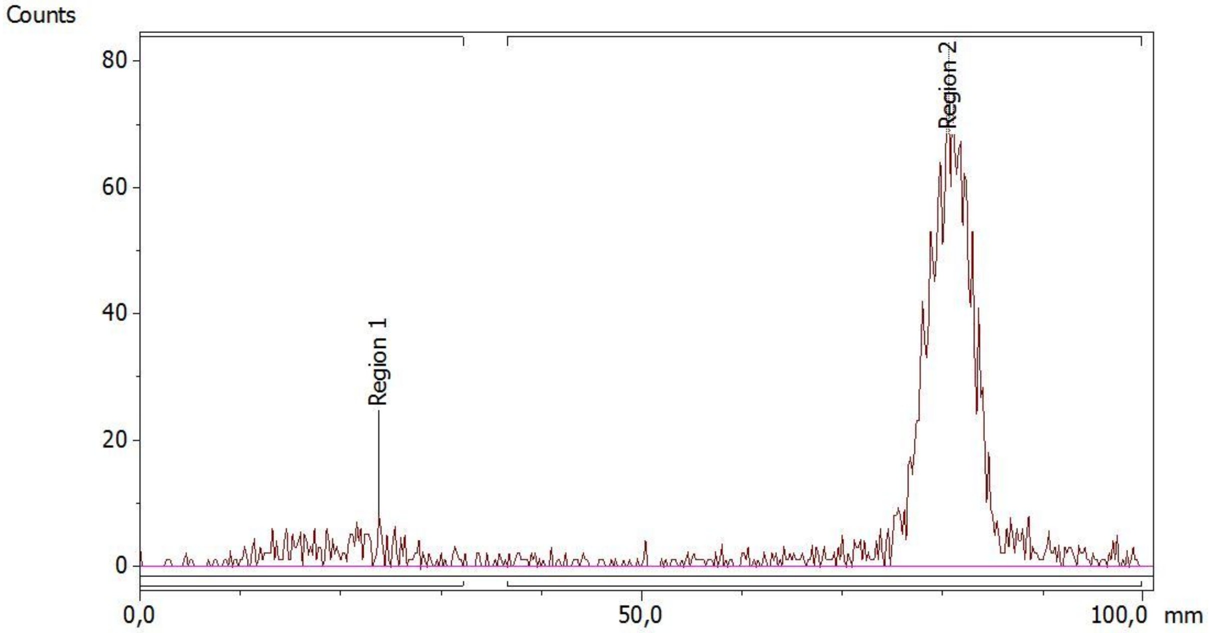

Figure S124: Radio-TLC chromatogram of [^{18}F]H_2N-Glu-(SiFA)SeFe-Gly-Asp-OH (3X_2) at pH 6.5, 1 mM aq. NaF, RT, t = 0 min (flow agent: 60% MeCN/ 40% PBS (6/4 v/v) with 10% NaOAc in H_2O (2 M) and 1% TFA, stationary phase: TLC Silica gel 60 F254 from Merck Millipore).

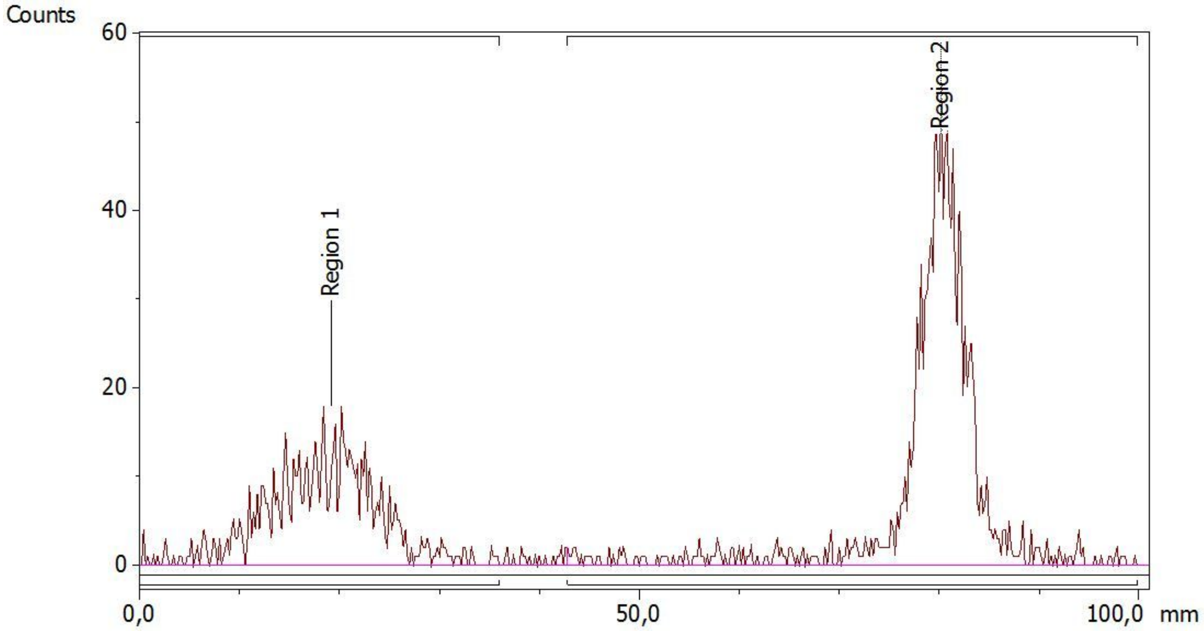

Figure S125: Radio-TLC chromatogram of [^{18}F]H_2N-Glu-(SiFA)SeFe-Gly-Asp-OH (3X_2) at pH 6.5, 1 mM aq. NaF, RT, t = 30 min (flow agent: 60% MeCN/ 40% PBS (6/4 v/v) with 10% NaOAc in H_2O (2 M) and 1% TFA, stationary phase: TLC Silica gel 60 F254 from Merck Millipore).

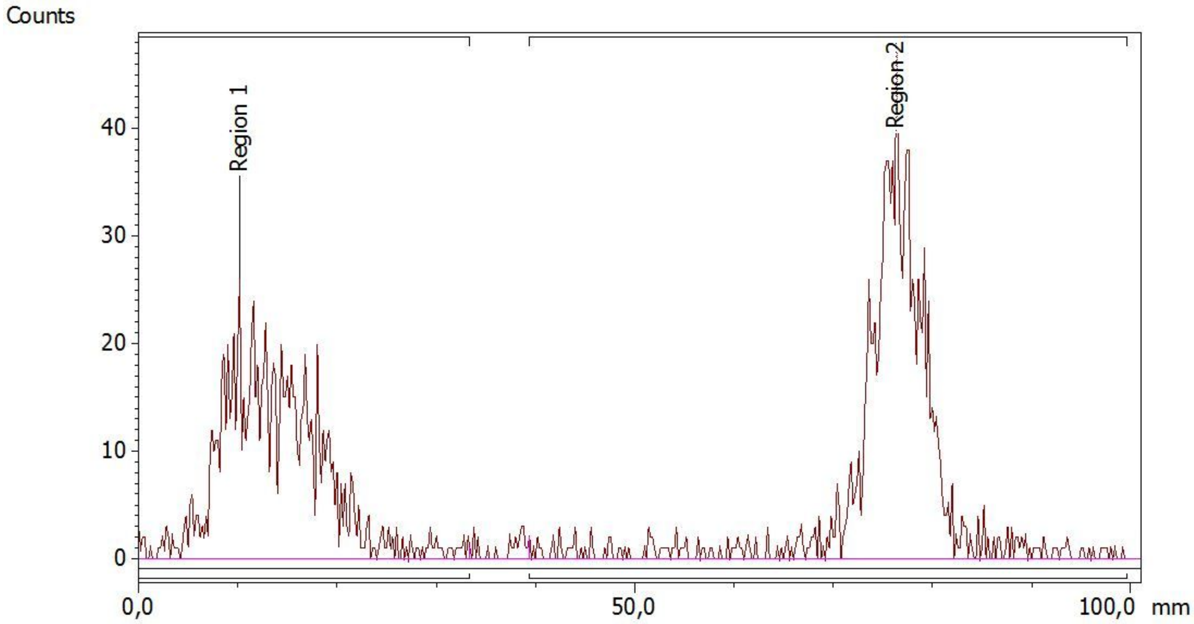

Figure S126: Radio-TLC chromatogram of [^{18}F]H_2N-Glu-(SiFA)SeFe-Gly-Asp-OH (3X_2) at pH 6.5, 1 mM aq. NaF, RT, t = 60 min (flow agent: 60% MeCN/ 40% PBS (6/4 v/v) with 10% NaOAc in H_2O (2 M) and 1% TFA, stationary phase: TLC Silica gel 60 F254 from Merck Millipore).

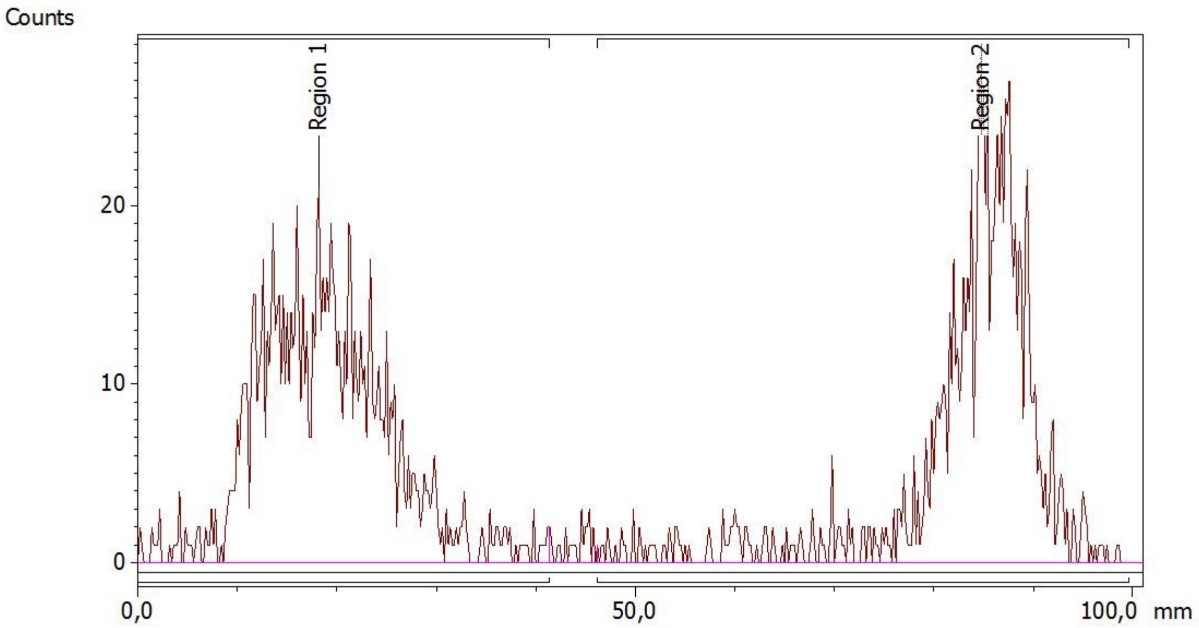

Figure S127: Radio-TLC chromatogram of [^{18}F]H_2N-Glu-(SiFA)SeFe-Gly-Asp-OH (3X_2) at pH 6.5, 1 mM aq. NaF, RT, t = 90 min (flow agent: 60% MeCN/ 40% PBS (6/4 v/v) with 10% NaOAc in H_2O (2 M) and 1% TFA, stationary phase: TLC Silica gel 60 F254 from Merck Millipore).

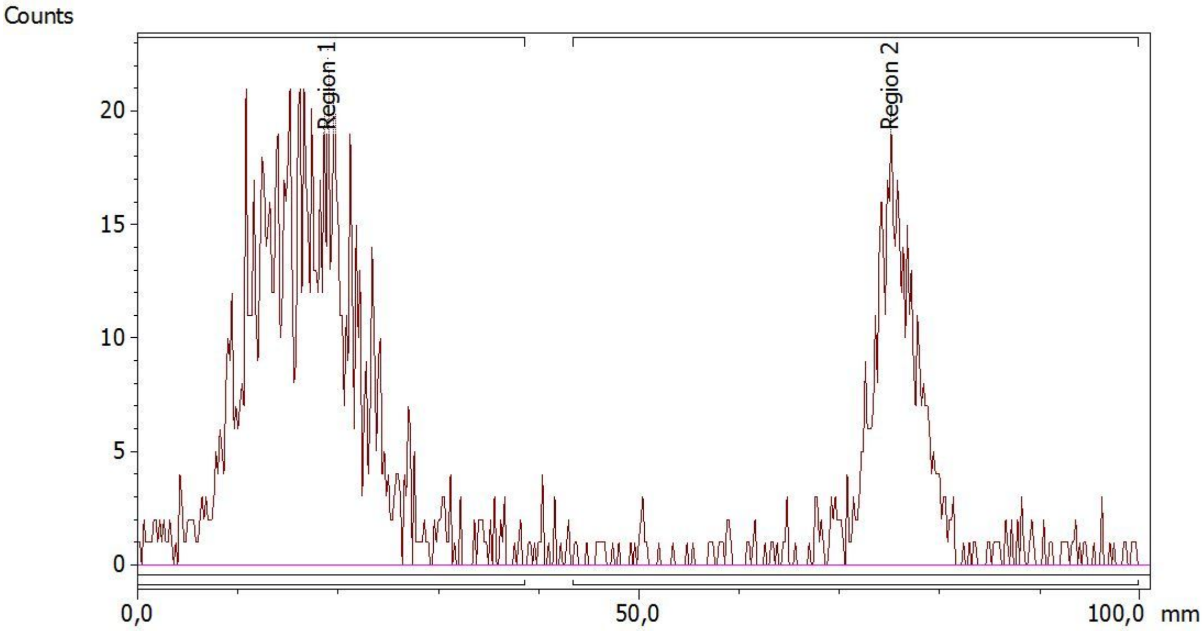

Figure S128: Radio-TLC chromatogram of [^{18}F]H_2N-Glu-(SiFA)SeFe-Gly-Asp-OH (3X_2) at pH 6.5, 1 mM aq. NaF, RT, t = 120 min (flow agent: 60% MeCN/ 40% PBS (6/4 v/v) with 10% NaOAc in H_2O (2 M) and 1% TFA, stationary phase: TLC Silica gel 60 F254 from Merck Millipore).

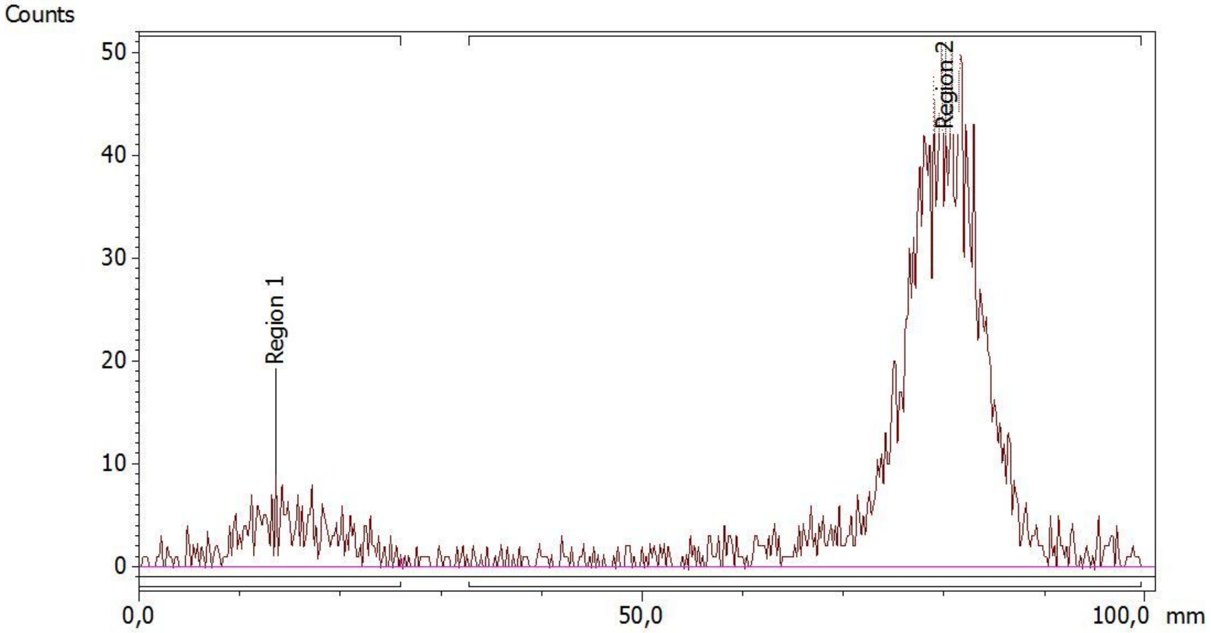

Figure S129: Radio-TLC chromatogram of [^{18}F]H_2N-(SiFA)SeFe-Gly-Tyr-OH (1X_3) at pH 6.5, 1 mM aq. NaF, RT, t = 0 min (flow agent: 60% MeCN/ 40% PBS (6/4 v/v) with 10% NaOAc in H_2O (2 M) and 1% TFA, stationary phase: TLC Silica gel 60 F254 from Merck Millipore).

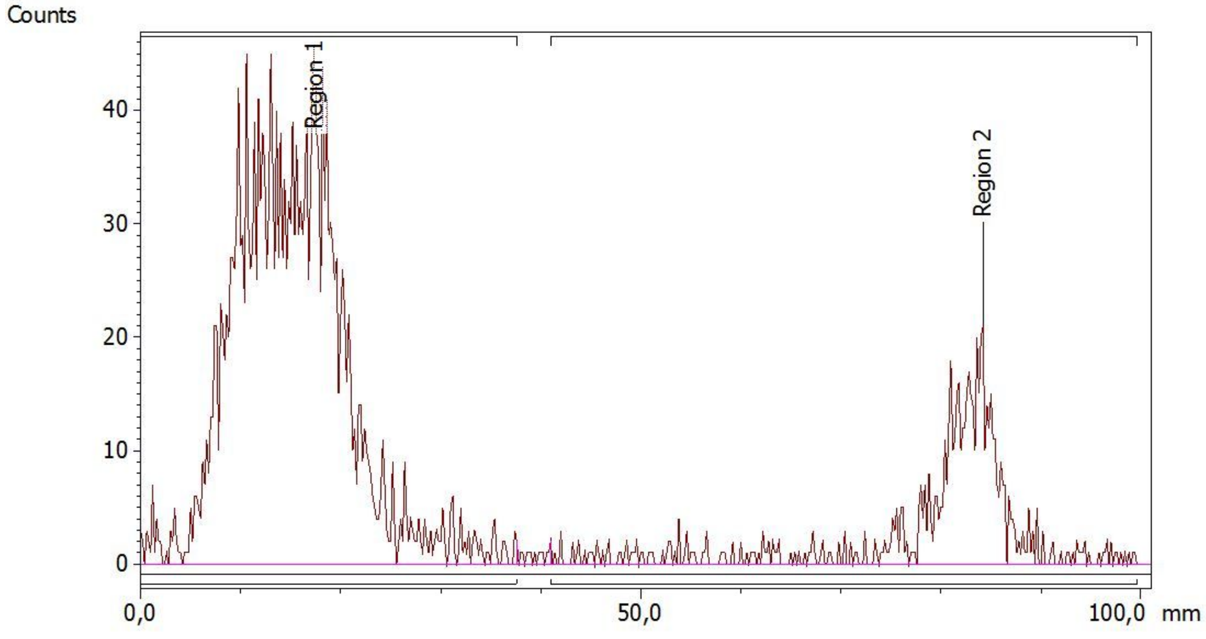

Figure S130: Radio-TLC chromatogram of [^{18}F]H_2N-(SiFA)SeFe-Gly-Tyr-OH (1X_3) at pH 6.5, 1 mM aq. NaF, RT, t = 30 min (flow agent: 60% MeCN/ 40% PBS (6/4 v/v) with 10% NaOAc in H_2O (2 M) and 1% TFA, stationary phase: TLC Silica gel 60 F254 from Merck Millipore).

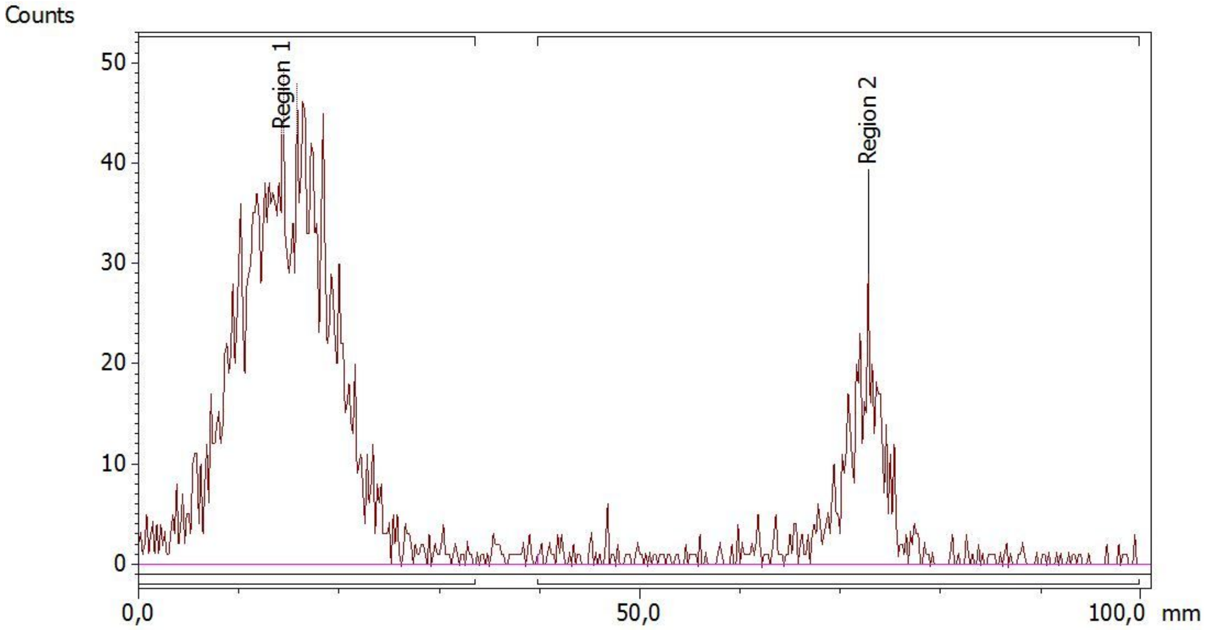

Figure S131: Radio-TLC chromatogram of [^{18}F]H_2N-(SiFA)SeFe-Gly-Tyr-OH (1X_3) at pH 6.5, 1 mM aq. NaF, RT, t = 60 min (flow agent: 60% MeCN/ 40% PBS (6/4 v/v) with 10% NaOAc in H_2O (2 M) and 1% TFA, stationary phase: TLC Silica gel 60 F254 from Merck Millipore).

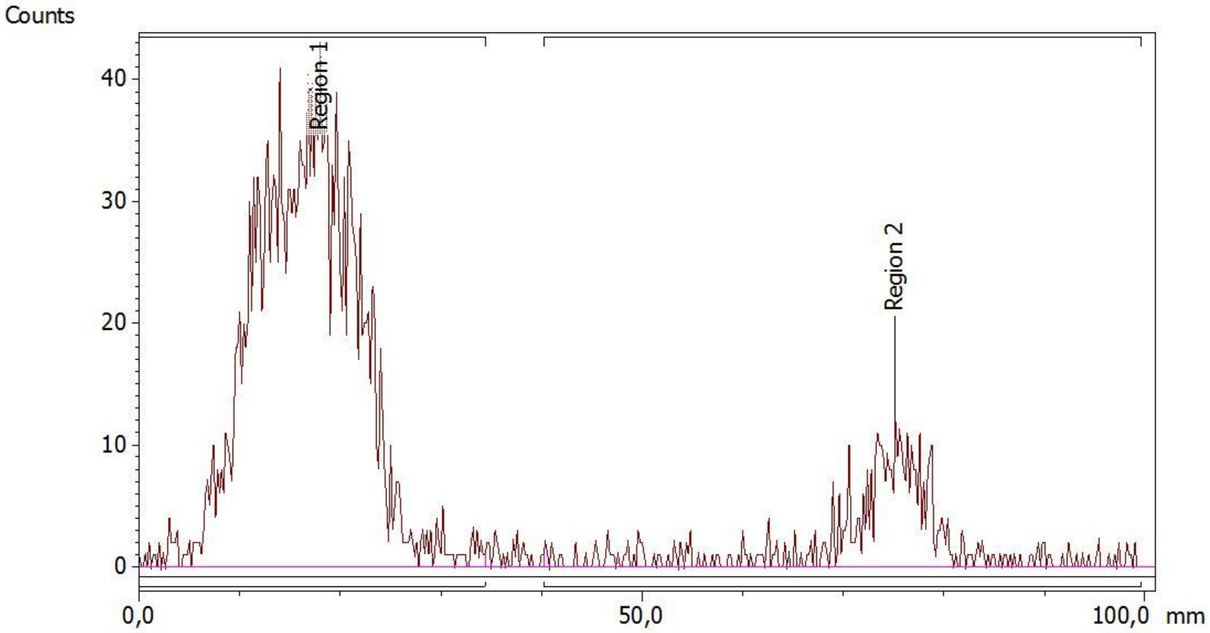

Figure S132: Radio-TLC chromatogram of [^{18}F]H_2N-(SiFA)SeFe-Gly-Tyr-OH (1X_3) at pH 6.5, 1 mM aq. NaF, RT, t = 90 min (flow agent: 60% MeCN/ 40% PBS (6/4 v/v) with 10% NaOAc in H_2O (2 M) and 1% TFA, stationary phase: TLC Silica gel 60 F254 from Merck Millipore).

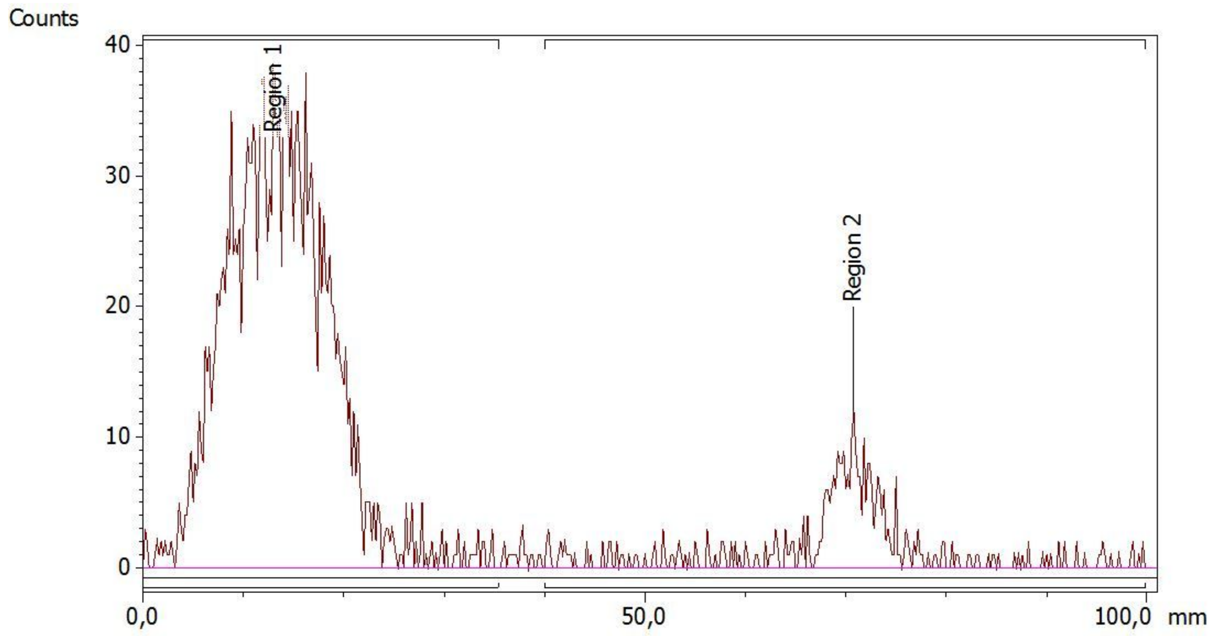

Figure S133: Radio-TLC chromatogram of [^{18}F]H_2N-(SiFA)SeFe-Gly-Tyr-OH ($1X_3$) at pH 6.5, 1 mM aq. NaF, RT, t = 120 min (flow agent: 60% MeCN/ 40% PBS (6/4 v/v) with 10% NaOAc in H_2O (2 M) and 1% TFA, stationary phase: TLC Silica gel 60 F254 from Merck Millipore).

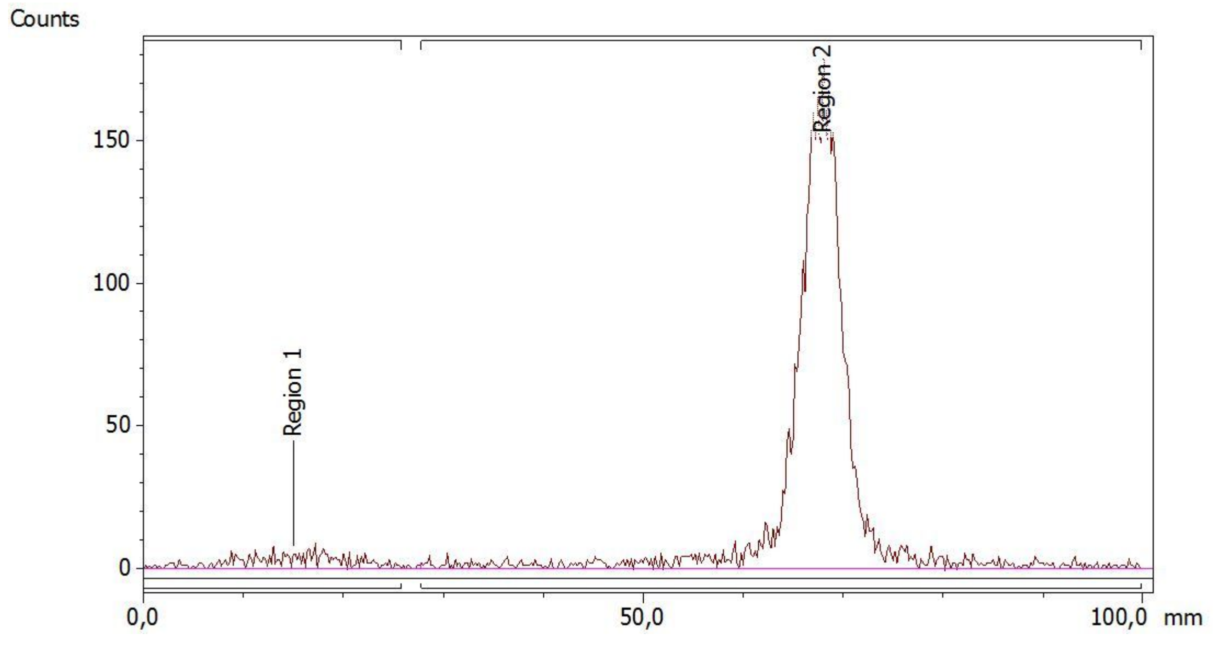

Figure S134: Radio-TLC chromatogram of [^{18}F]H_2N-Lys-(SiFA)SeFe-Gly-Tyr-OH ($2X_3$) at pH 6.5, 1 mM aq. NaF, RT, t = 0 min (flow agent: 60% MeCN/ 40% PBS (6/4 v/v) with 10% NaOAc in H_2O (2 M) and 1% TFA, stationary phase: TLC Silica gel 60 F254 from Merck Millipore).

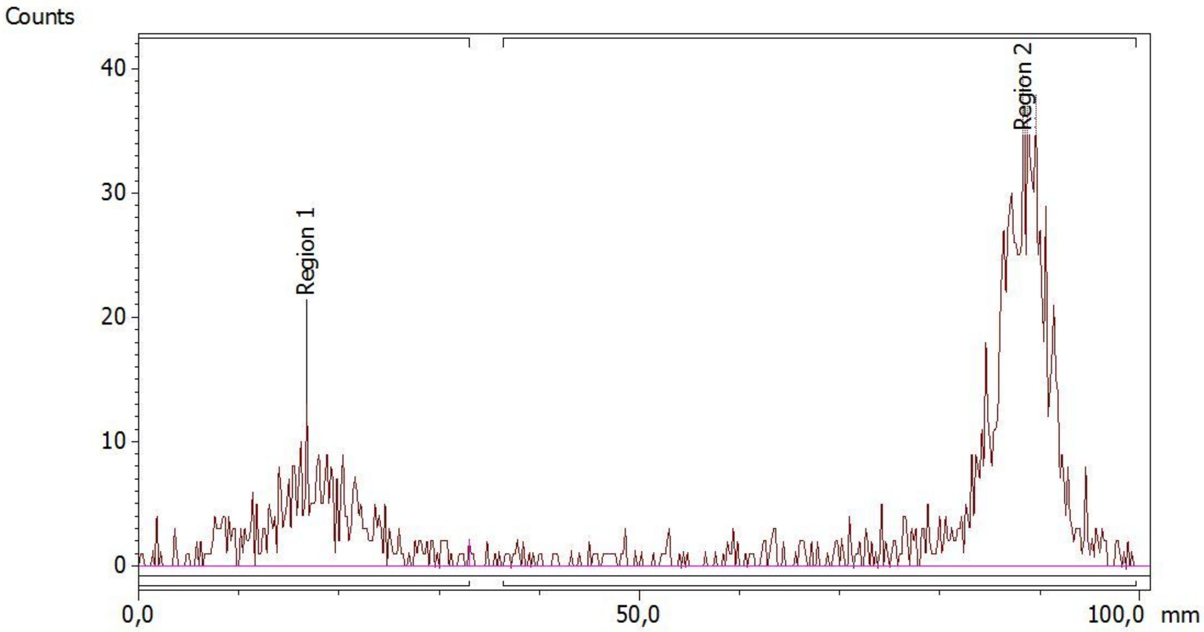

Figure S135: Radio-TLC chromatogram of [^{18}F]H_2N-Lys-(SiFA)SeFe-Gly-Tyr-OH (2X_3) at pH 6.5, 1 mM aq. NaF, RT, t = 30 min (flow agent: 60% MeCN/ 40% PBS (6/4 v/v) with 10% NaOAc in H_2O (2 M) and 1% TFA, stationary phase: TLC Silica gel 60 F254 from Merck Millipore).

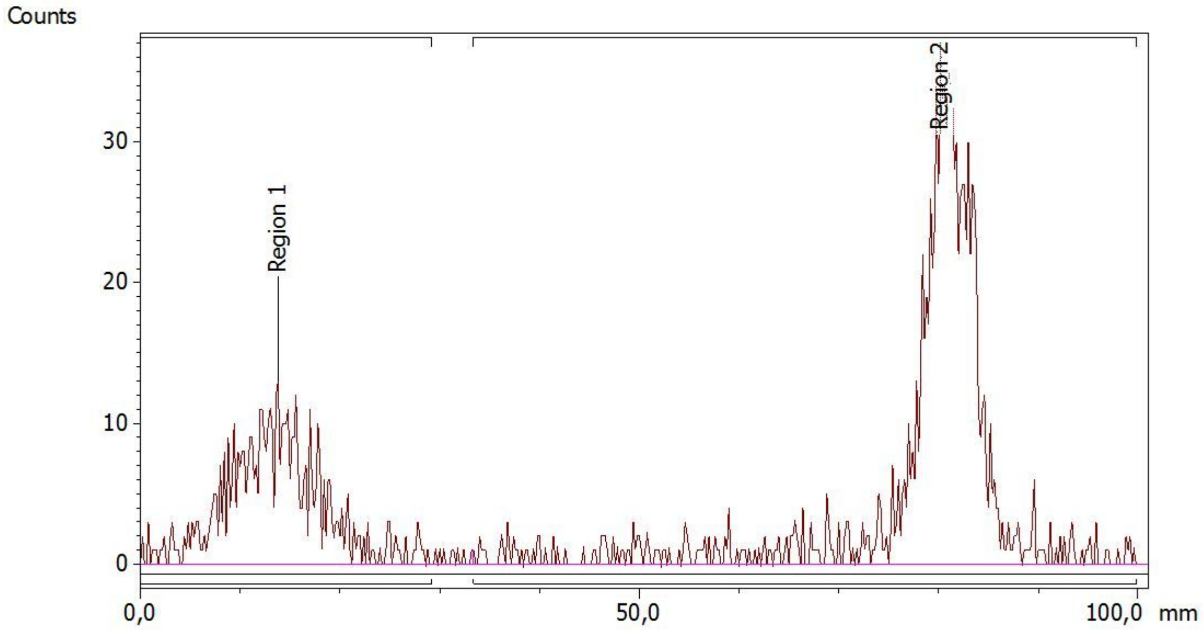

Figure S136: Radio-TLC chromatogram of [^{18}F]H_2N-Lys-(SiFA)SeFe-Gly-Tyr-OH (2X_3) at pH 6.5, 1 mM aq. NaF, RT, t = 60 min (flow agent: 60% MeCN/ 40% PBS (6/4 v/v) with 10% NaOAc in H_2O (2 M) and 1% TFA, stationary phase: TLC Silica gel 60 F254 from Merck Millipore).

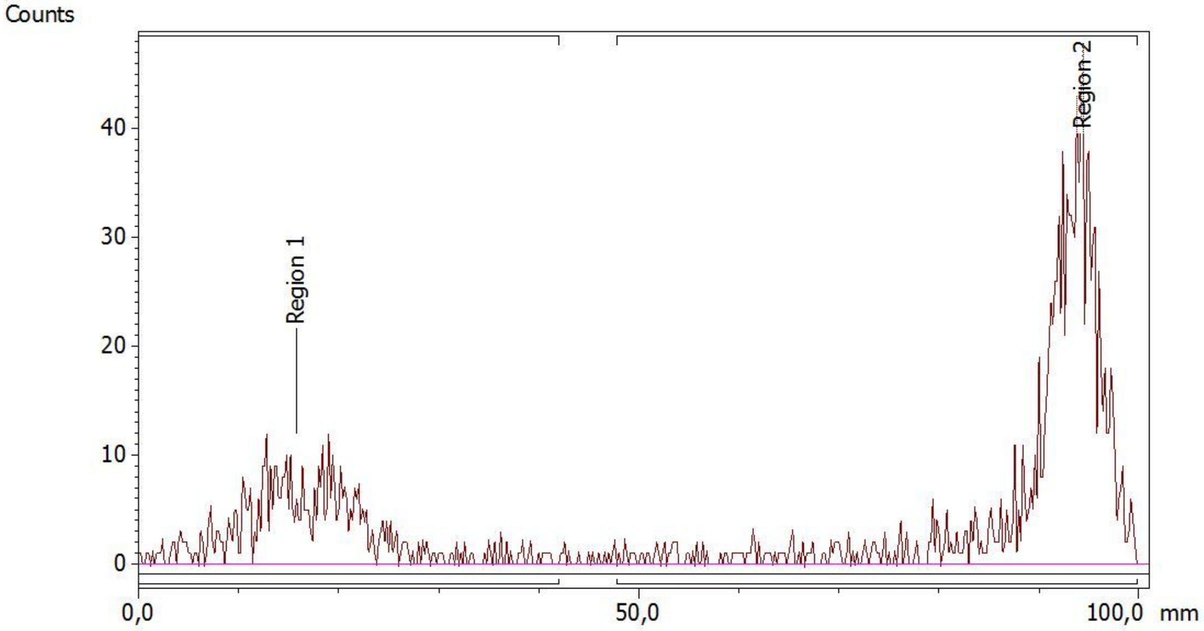

Figure S137: Radio-TLC chromatogram of [^{18}F]H_2N-Lys-(SiFA)SeFe-Gly-Tyr-OH (2X_3) at pH 6.5, 1 mM aq. NaF, RT, t = 90 min (flow agent: 60% MeCN/ 40% PBS (6/4 v/v) with 10% NaOAc in H_2O (2 M) and 1% TFA, stationary phase: TLC Silica gel 60 F254 from Merck Millipore).

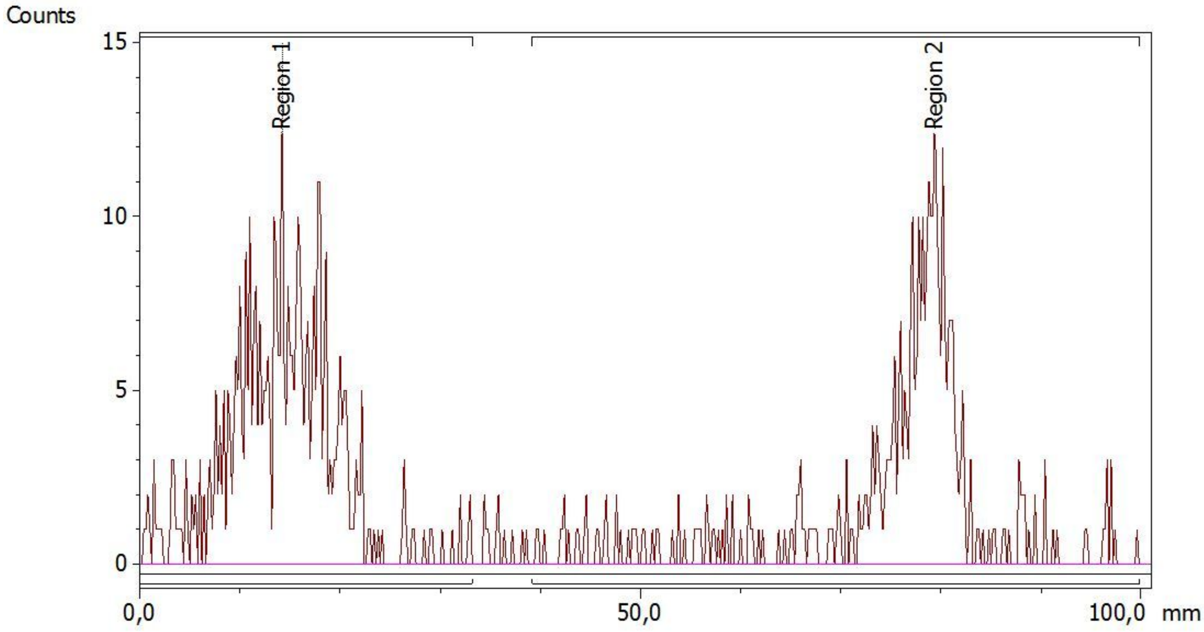

Figure S138: Radio-TLC chromatogram of [^{18}F]H_2N-Lys-(SiFA)SeFe-Gly-Tyr-OH (2X_3) at pH 6.5, 1 mM aq. NaF, RT, t = 120 min (flow agent: 60% MeCN/ 40% PBS (6/4 v/v) with 10% NaOAc in H_2O (2 M) and 1% TFA, stationary phase: TLC Silica gel 60 F254 from Merck Millipore).

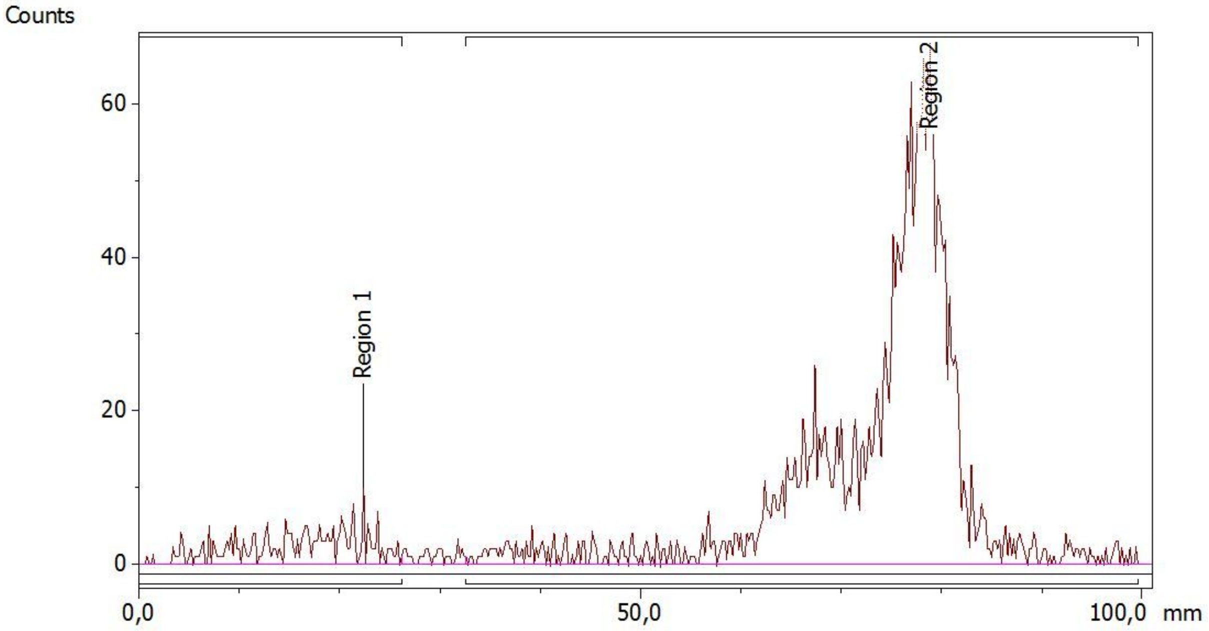

Figure S139: Radio-TLC chromatogram of [^{18}F]H_2N-Glu-(SiFA)SeFe-Gly-Tyr-OH ($3X_3$) at pH 6.5, 1 mM aq. NaF, RT, t = 0 min (flow agent: 60% MeCN/ 40% PBS (6/4 v/v) with 10% NaOAc in H_2O (2 M) and 1% TFA, stationary phase: TLC Silica gel 60 F254 from Merck Millipore).

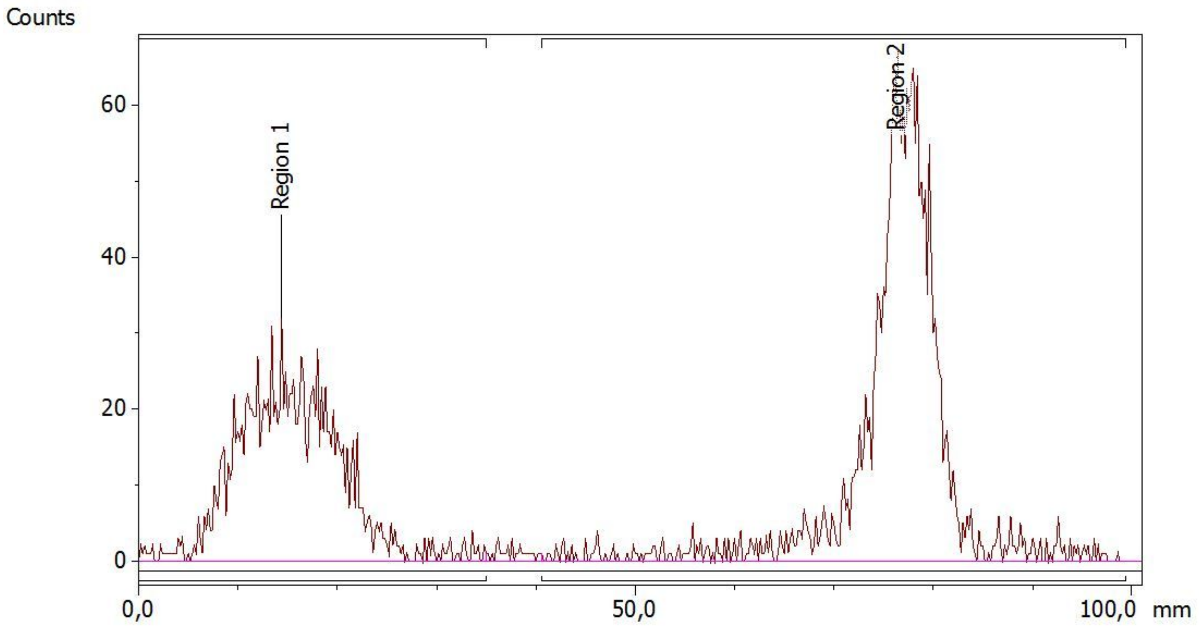

Figure S140: Radio-TLC chromatogram of [^{18}F]H_2N-Glu-(SiFA)SeFe-Gly-Tyr-OH ($3X_3$) at pH 6.5, 1 mM aq. NaF, RT, t = 30 min (flow agent: 60% MeCN/ 40% PBS (6/4 v/v) with 10% NaOAc in H_2O (2 M) and 1% TFA, stationary phase: TLC Silica gel 60 F254 from Merck Millipore).

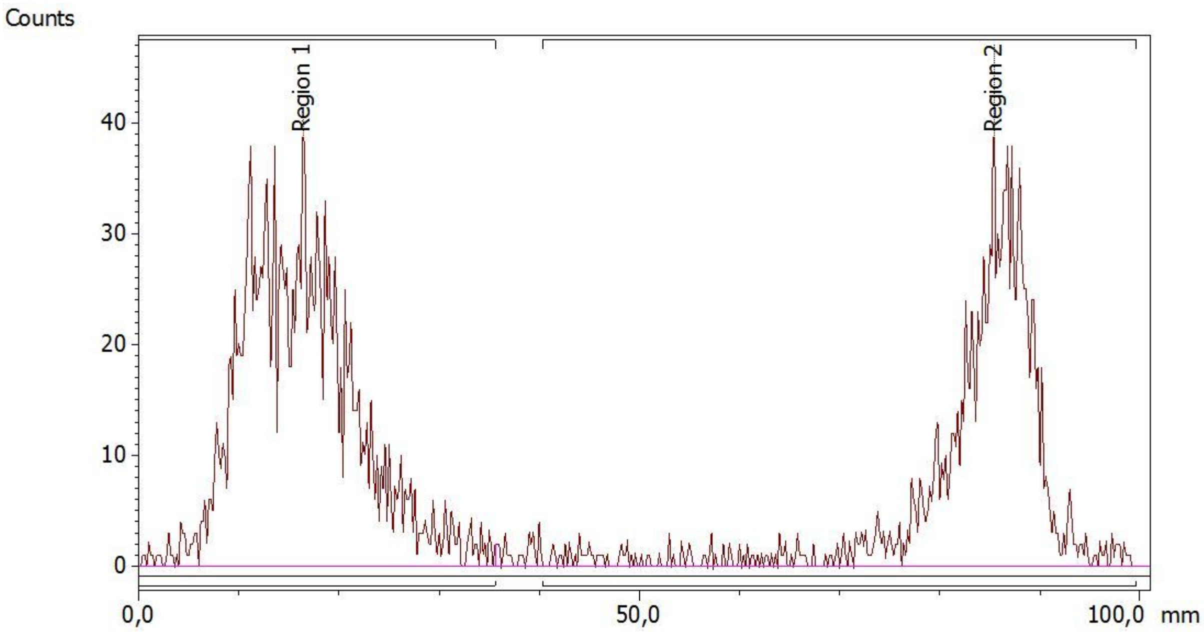

Figure S141: Radio-TLC chromatogram of [^{18}F]H_2N-Glu-(SiFA)SeFe-Gly-Tyr-OH ($3X_3$) at pH 6.5, 1 mM aq. NaF, RT, t = 60 min (flow agent: 60% MeCN/ 40% PBS (6/4 v/v) with 10% NaOAc in H_2O (2 M) and 1% TFA, stationary phase: TLC Silica gel 60 F254 from Merck Millipore).

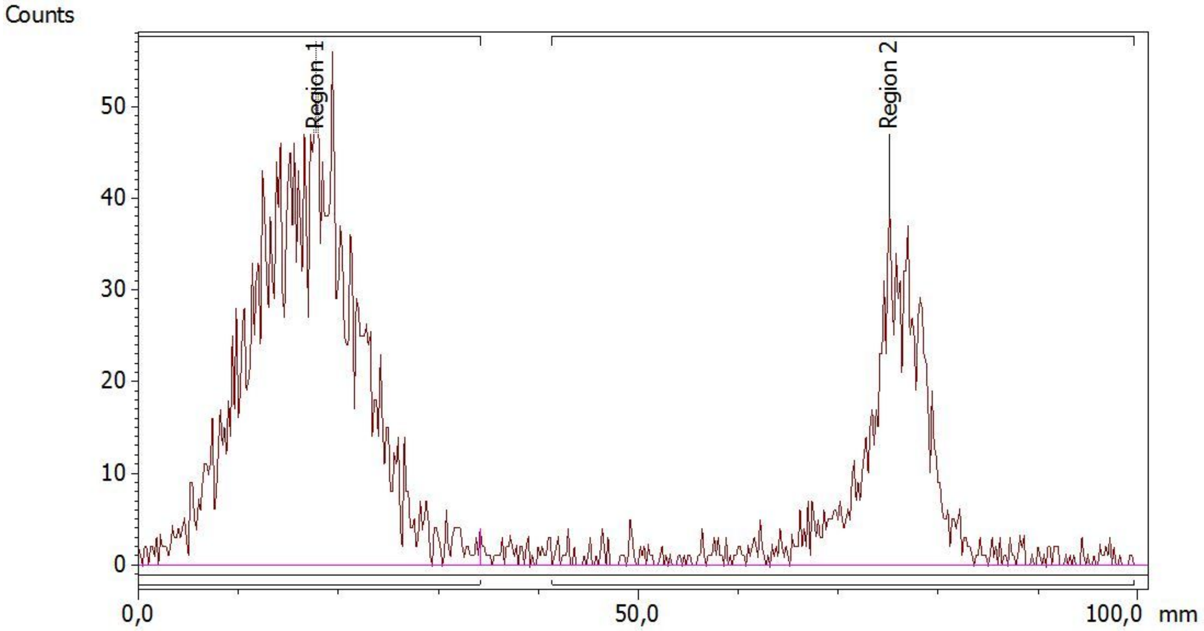

Figure S142: Radio-TLC chromatogram of [^{18}F]H_2N-Glu-(SiFA)SeFe-Gly-Tyr-OH ($3X_3$) at pH 6.5, 1 mM aq. NaF, RT, t = 90 min (flow agent: 60% MeCN/ 40% PBS (6/4 v/v) with 10% NaOAc in H_2O (2 M) and 1% TFA, stationary phase: TLC Silica gel 60 F254 from Merck Millipore).

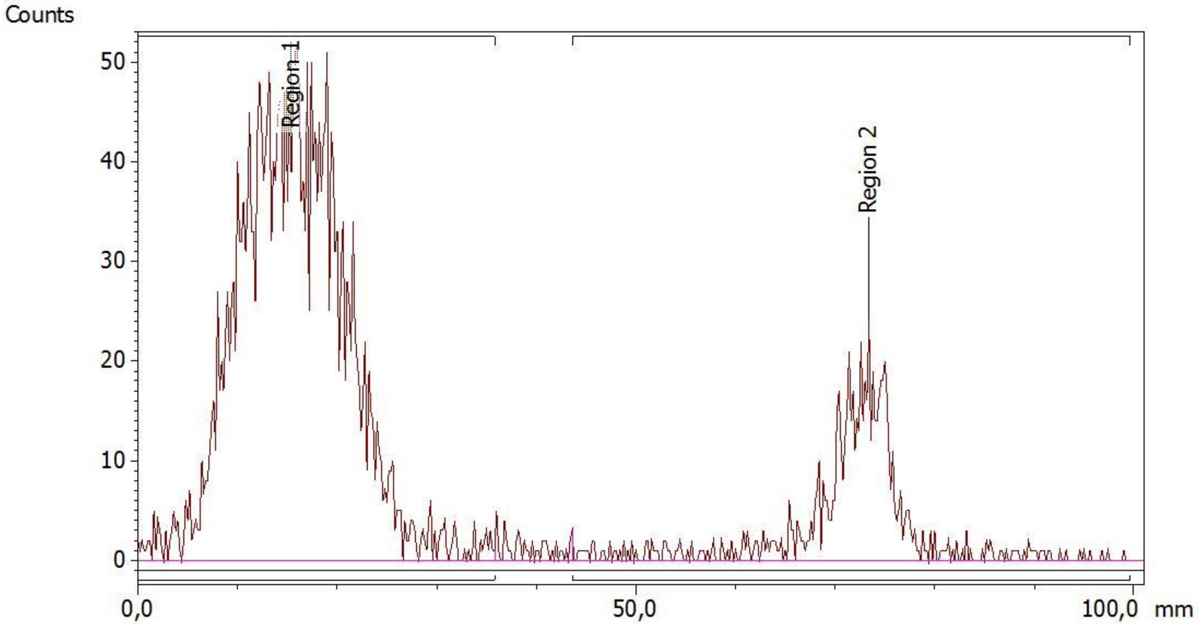

Figure S143: Radio-TLC chromatogram of [^{18}F]H_2N-Glu-(SiFA)SeFe-Gly-Tyr-OH ($3X_3$) at pH 6.5, 1 mM aq. NaF, RT, t = 120 min (flow agent: 60% MeCN/ 40% PBS (6/4 v/v) with 10% NaOAc in H_2O (2 M) and 1% TFA, stationary phase: TLC Silica gel 60 F254 from Merck Millipore).

2.6. Stability towards physiological conditions

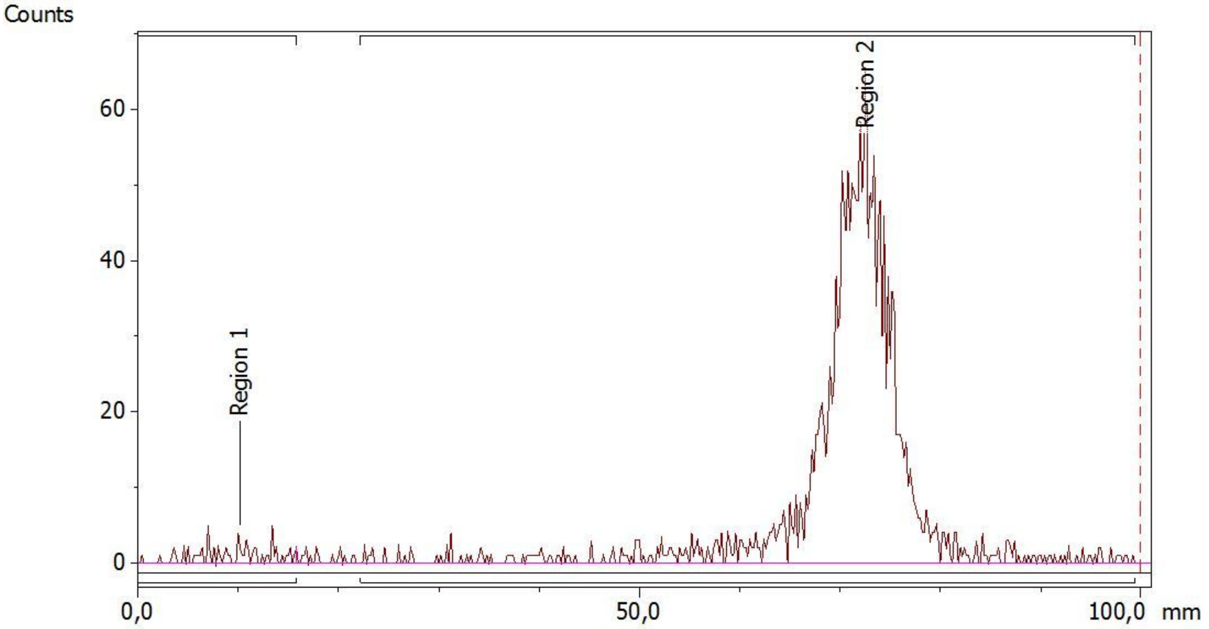

Figure S144: Radio-TLC chromatogram of [^{18}F]H_2N-(SiFA)SeFe-Gly-Lys-OH ($1X_1$) at pH 7.4, 37 °C, t = 0 min (flow agent: 60% MeCN/ 40% PBS (6/4 v/v) with 10% NaOAc in H_2O (2 M) and 1% TFA, stationary phase: TLC Silica gel 60 F254 from Merck Millipore).

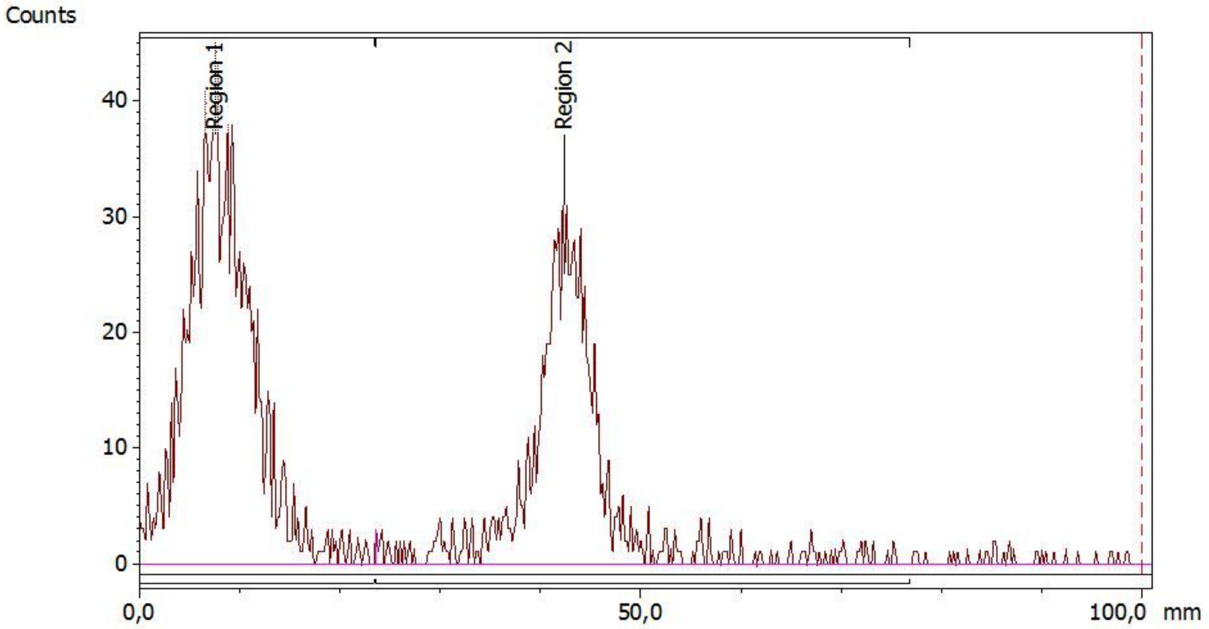

Figure S145: Radio-TLC chromatogram of [^{18}F]H_2N-(SiFA)SeFe-Gly-Lys-OH ($1X_1$) at pH 7.4, 37 °C, t = 30 min (flow agent: 60% MeCN/ 40% PBS (6/4 v/v) with 10% NaOAc in H_2O (2 M) and 1% TFA, stationary phase: TLC Silica gel 60 F254 from Merck Millipore).

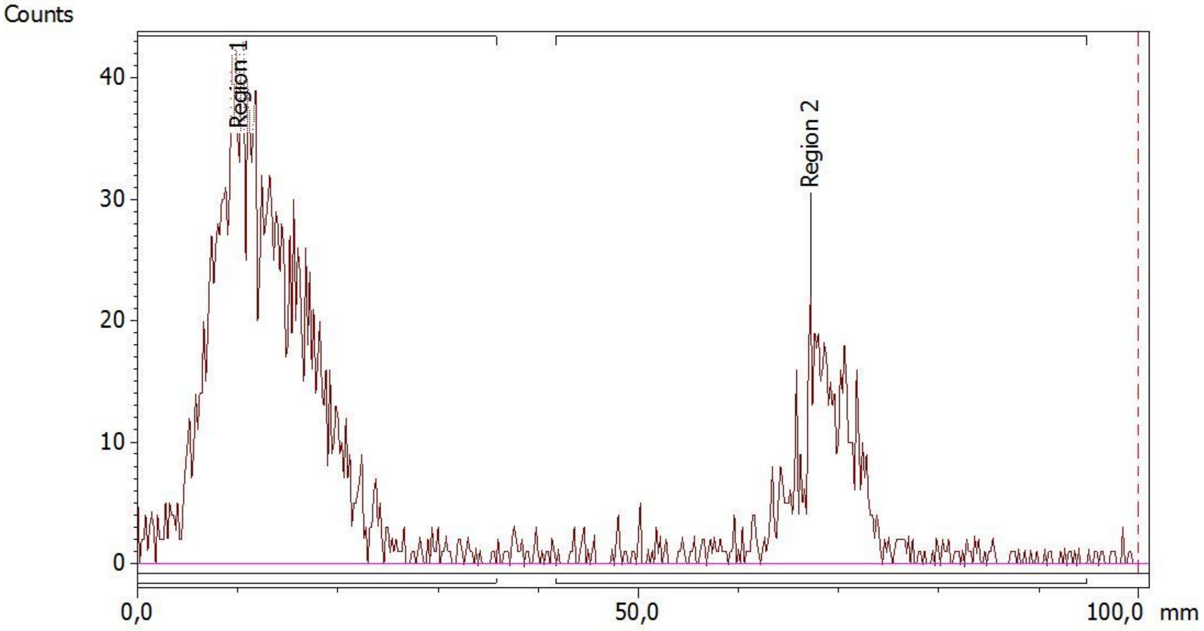

Figure S146: Radio-TLC chromatogram of [^{18}F]H_2N-(SiFA)SeFe-Gly-Lys-OH ($1X_1$) at pH 7.4, 37 °C, t = 60 min (flow agent: 60% MeCN/ 40% PBS (6/4 v/v) with 10% NaOAc in H_2O (2 M) and 1% TFA, stationary phase: TLC Silica gel 60 F254 from Merck Millipore).

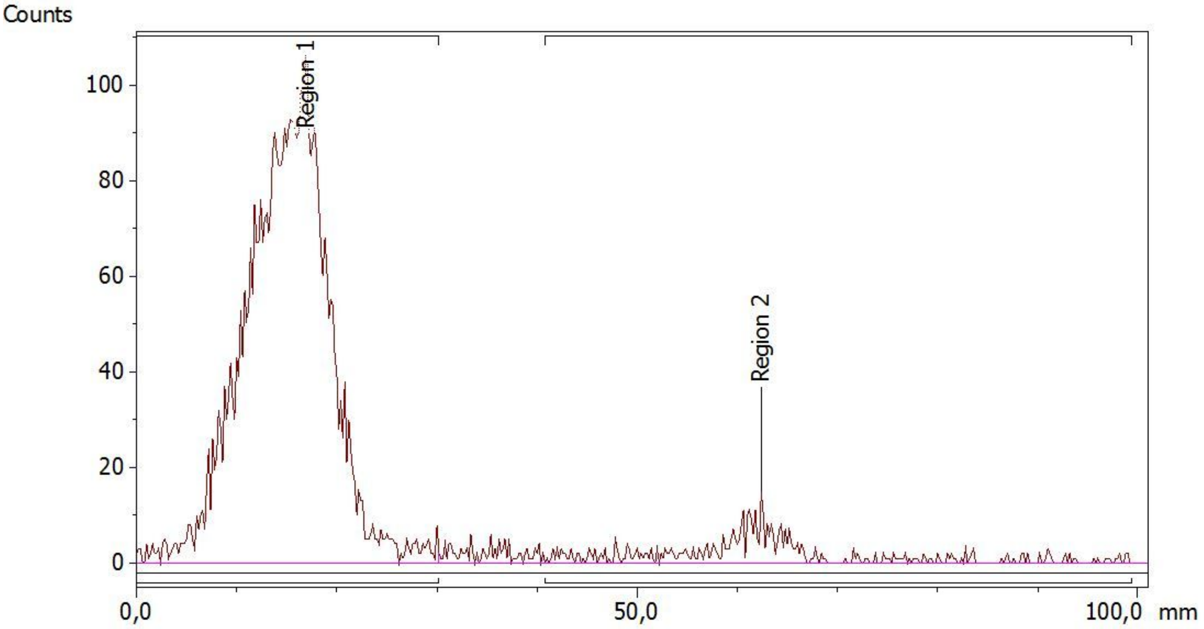

Figure S147: Radio-TLC chromatogram of [^{18}F]H_2N-(SiFA)SeFe-Gly-Lys-OH ($1X_1$) at pH 7.4, 37 °C, t = 90 min (flow agent: 60% MeCN/ 40% PBS (6/4 v/v) with 10% NaOAc in H_2O (2 M) and 1% TFA, stationary phase: TLC Silica gel 60 F254 from Merck Millipore).

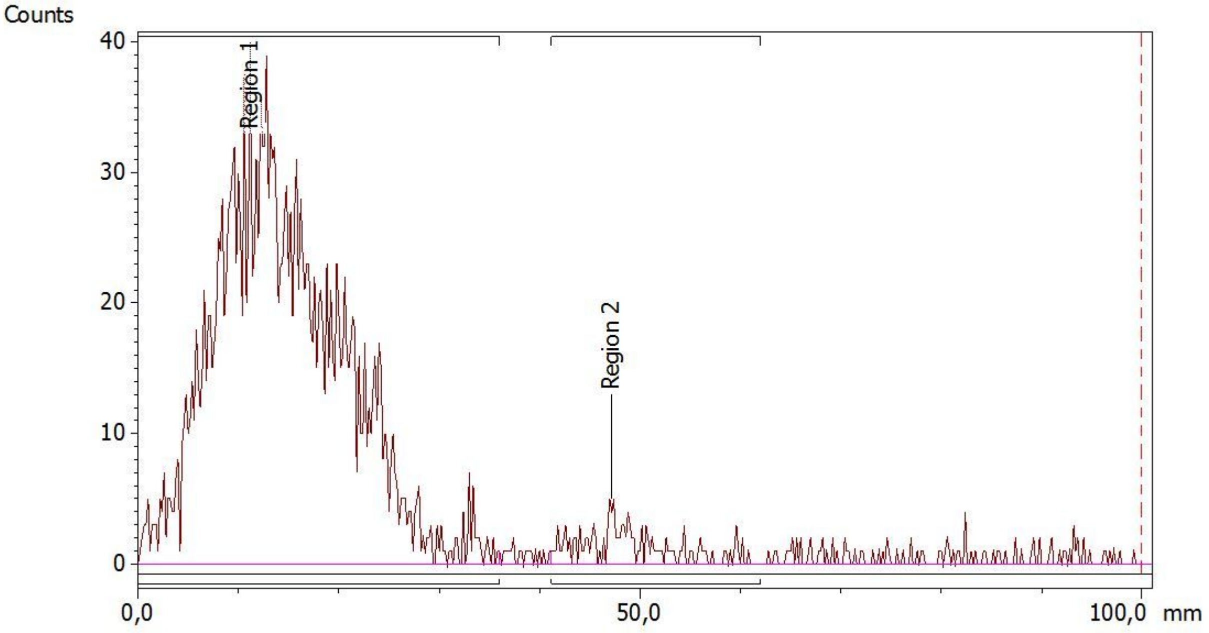

Figure S148: Radio-TLC chromatogram of [^{18}F]H_2N-(SiFA)SeFe-Gly-Lys-OH ($1X_1$) at pH 7.4, 37 °C, t = 120 min (flow agent: 60% MeCN/ 40% PBS (6/4 v/v) with 10% NaOAc in H_2O (2 M) and 1% TFA, stationary phase: TLC Silica gel 60 F254 from Merck Millipore).

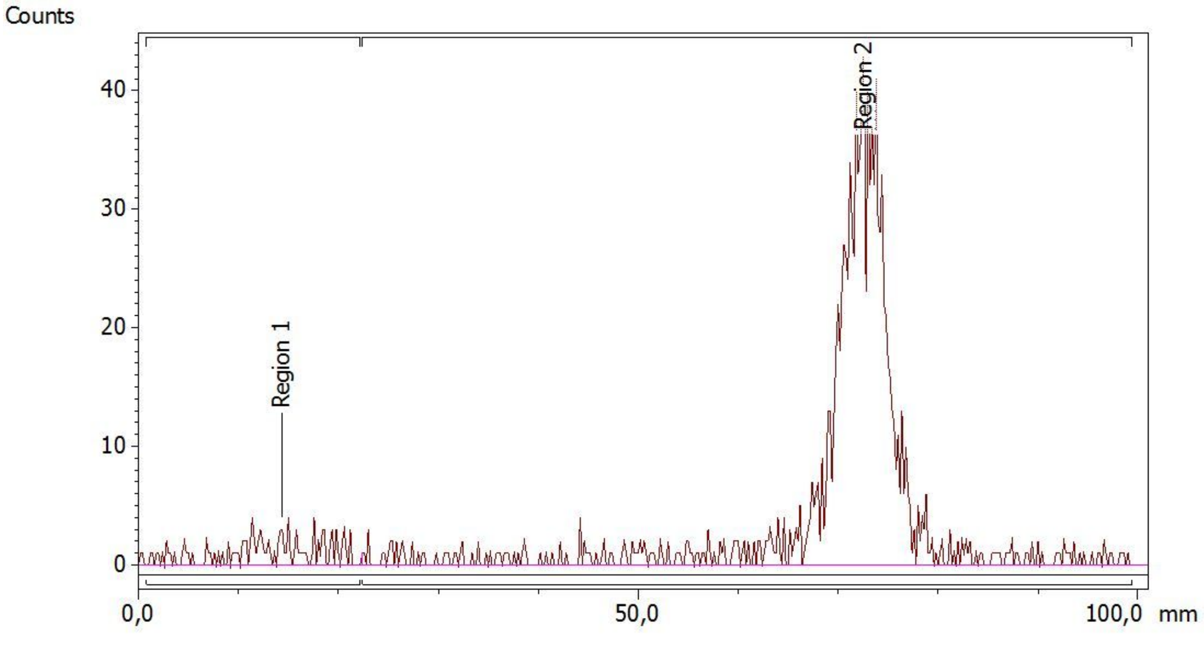

Figure S149: Radio-TLC chromatogram of [^{18}F]H_2N-Lys-(SiFA)SeFe-Gly-Lys-OH ($2X_1$) at pH 7.4, 37 °C, t = 0 min (flow agent: 60% MeCN/ 40% PBS (6/4 v/v) with 10% NaOAc in H_2O (2 M) and 1% TFA, stationary phase: TLC Silica gel 60 F254 from Merck Millipore).

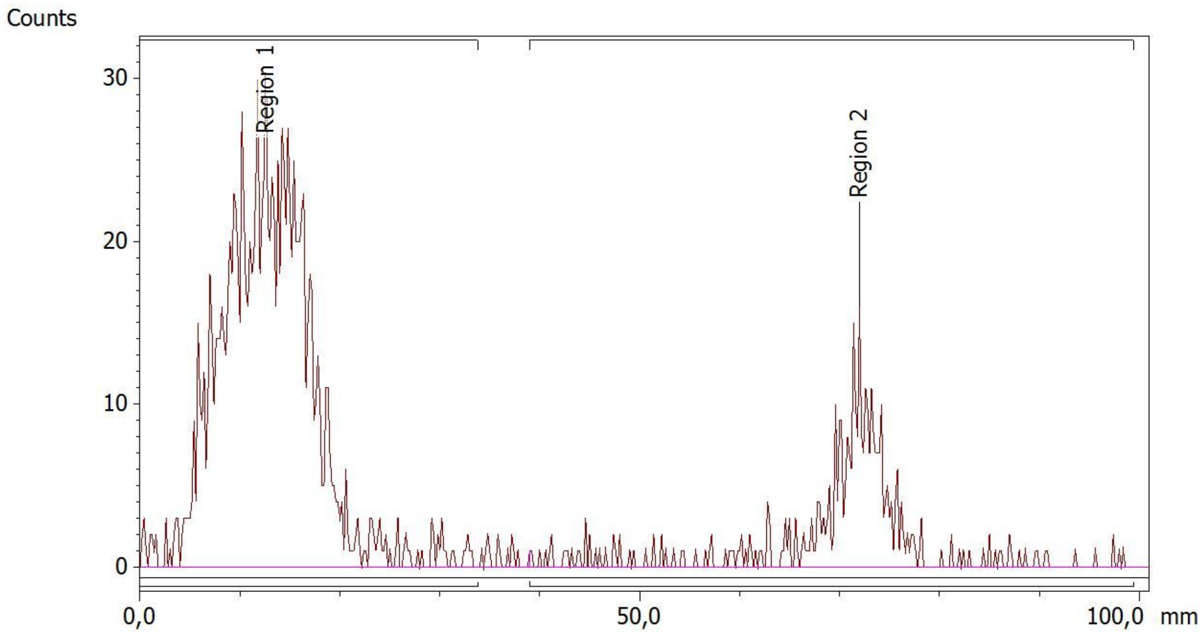

Figure S150: Radio-TLC chromatogram of [^{18}F]H$_2$N-Lys-(SiFA)SeFe-Gly-Lys-OH (2X$_1$) at pH 7.4, 37 °C, t = 30 min (flow agent: 60% MeCN/ 40% PBS (6/4 v/v) with 10% NaOAc in H$_2$O (2 M) and 1% TFA, stationary phase: TLC Silica gel 60 F254 from Merck Millipore).

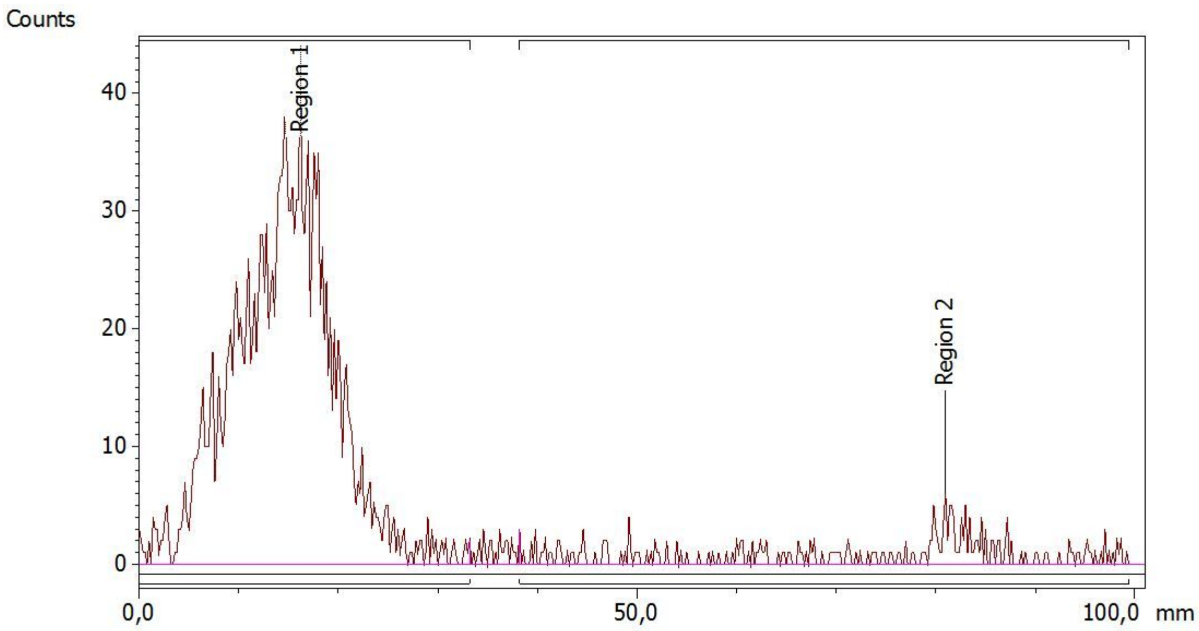

Figure S151: Radio-TLC chromatogram of [^{18}F]H$_2$N-Lys-(SiFA)SeFe-Gly-Lys-OH (2X$_1$) at pH 7.4, 37 °C, t = 60 min (flow agent: 60% MeCN/ 40% PBS (6/4 v/v) with 10% NaOAc in H$_2$O (2 M) and 1% TFA, stationary phase: TLC Silica gel 60 F254 from Merck Millipore).

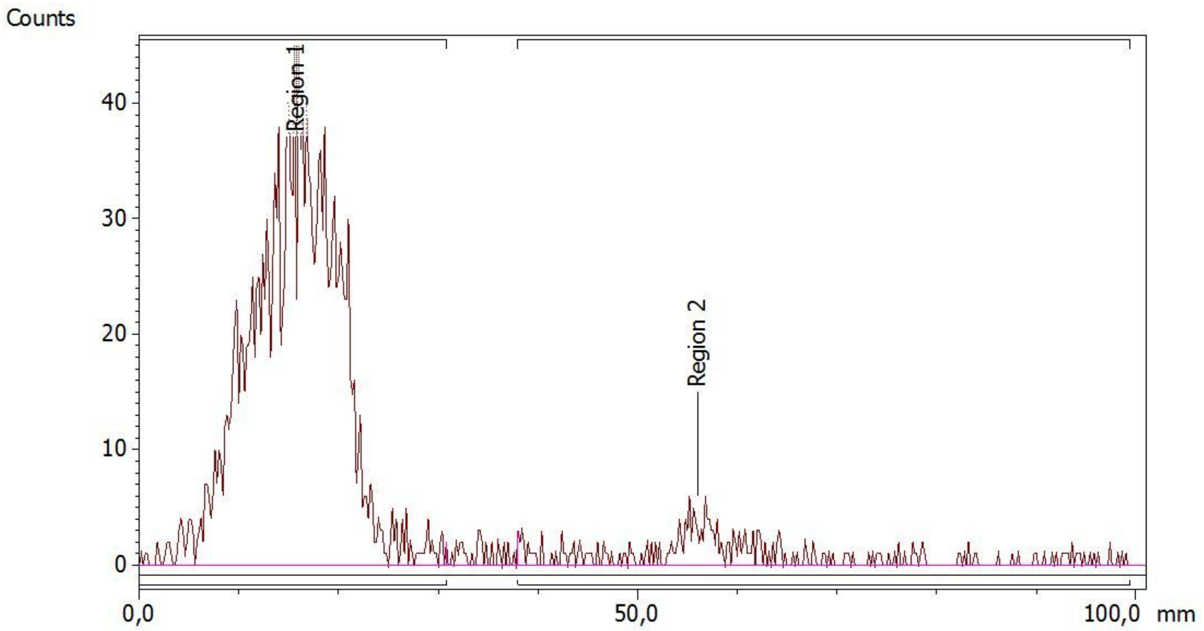

Figure S152: Radio-TLC chromatogram of [^{18}F]H_2N-Lys-(SiFA)SeFe-Gly-Lys-OH (2X_1) at pH 7.4, 37 °C, t = 90 min (flow agent: 60% MeCN/ 40% PBS (6/4 v/v) with 10% NaOAc in H_2O (2 M) and 1% TFA, stationary phase: TLC Silica gel 60 F254 from Merck Millipore).

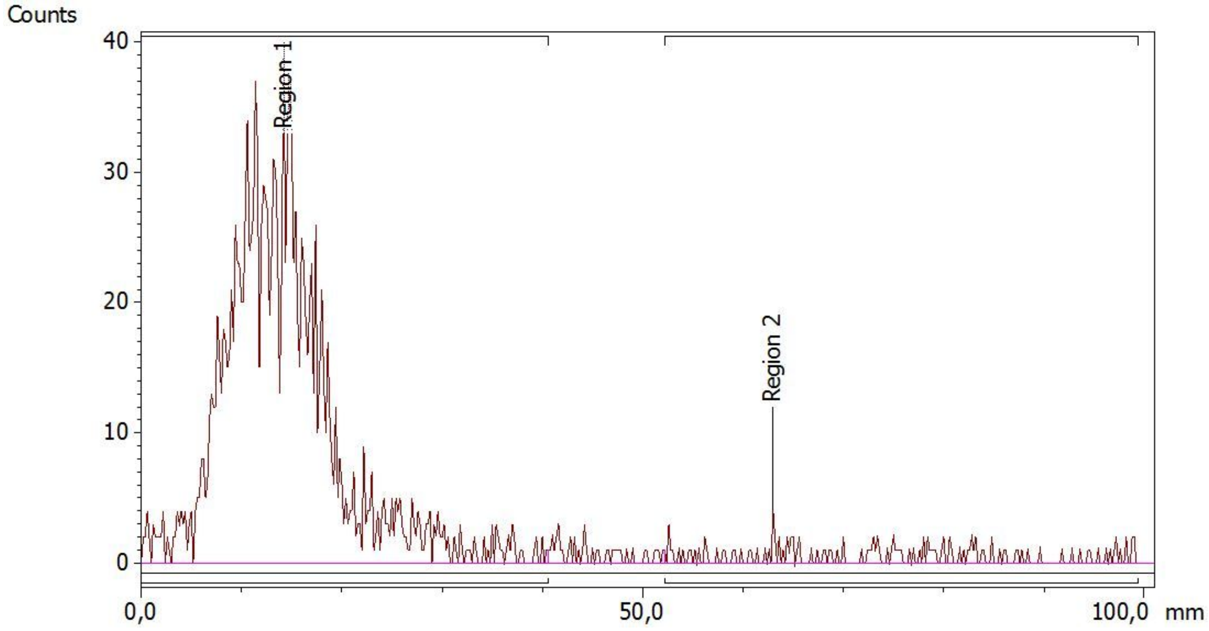

Figure S153: Radio-TLC chromatogram of [^{18}F]H_2N-Lys-(SiFA)SeFe-Gly-Lys-OH (2X_1) at pH 7.4, 37 °C, t = 120 min (flow agent: 60% MeCN/ 40% PBS (6/4 v/v) with 10% NaOAc in H_2O (2 M) and 1% TFA, stationary phase: TLC Silica gel 60 F254 from Merck Millipore).

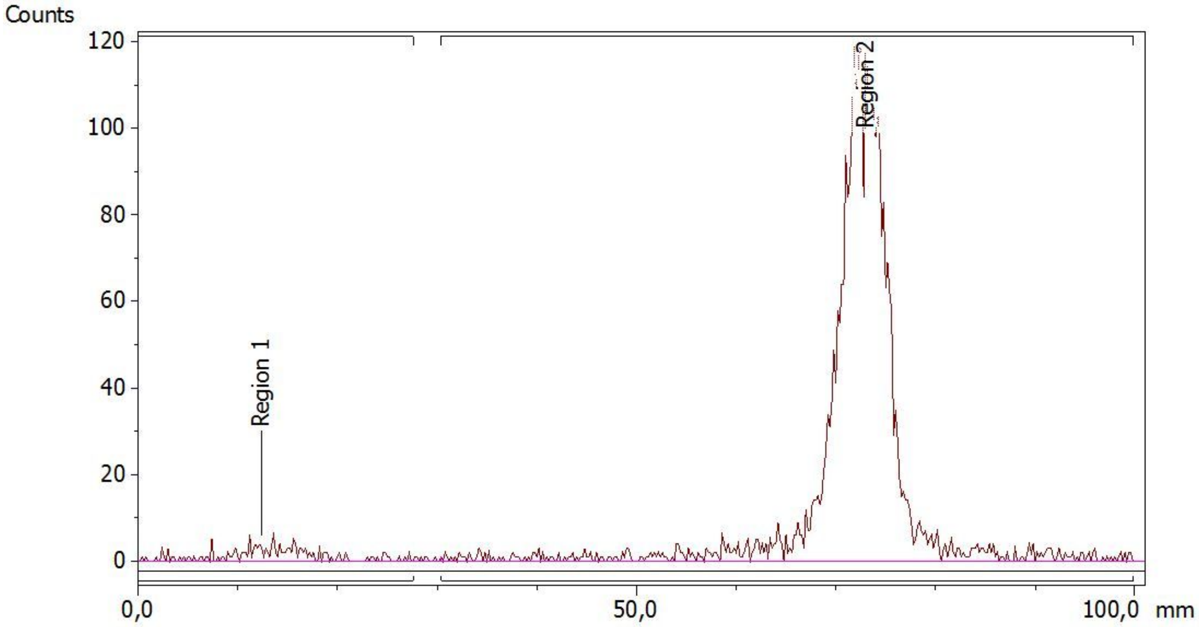

Figure S154: Radio-TLC chromatogram of [^{18}F]H_2N-Glu-(SiFA)SeFe-Gly-Lys-OH ($3X_1$) at pH 7.4, 37 °C, t = 0 min (flow agent: 60% MeCN/ 40% PBS (6/4 v/v) with 10% NaOAc in H_2O (2 M) and 1% TFA, stationary phase: TLC Silica gel 60 F254 from Merck Millipore).

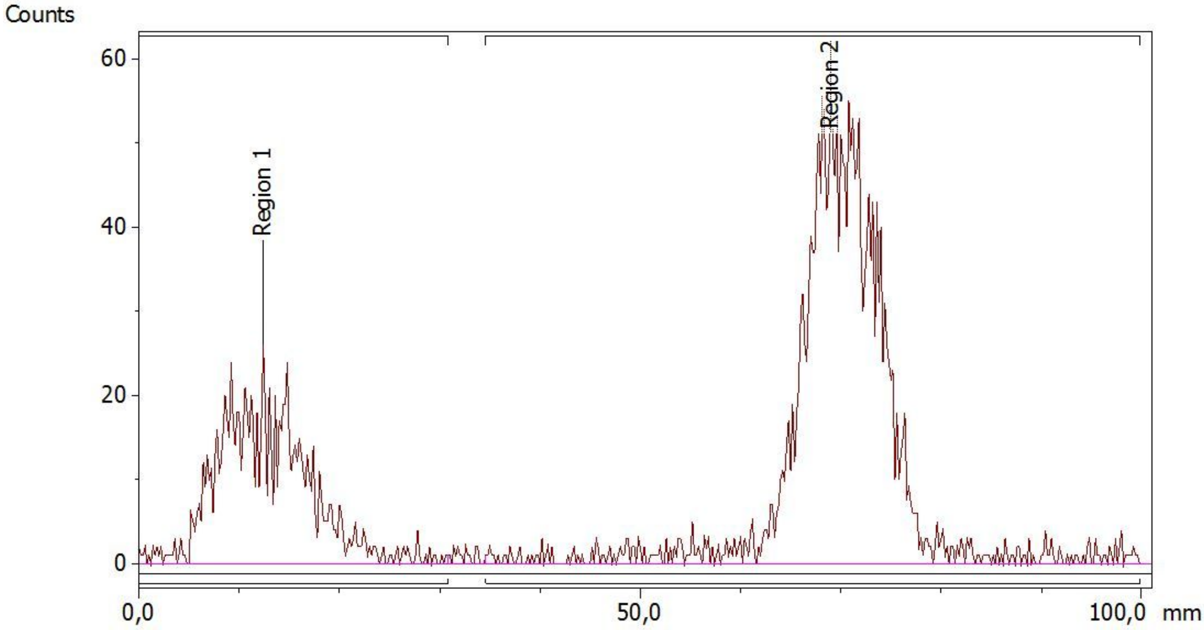

Figure S155: Radio-TLC chromatogram of [^{18}F]H_2N-Glu-(SiFA)SeFe-Gly-Lys-OH ($3X_1$) at pH 7.4, 37 °C, t = 30 min (flow agent: 60% MeCN/ 40% PBS (6/4 v/v) with 10% NaOAc in H_2O (2 M) and 1% TFA, stationary phase: TLC Silica gel 60 F254 from Merck Millipore).

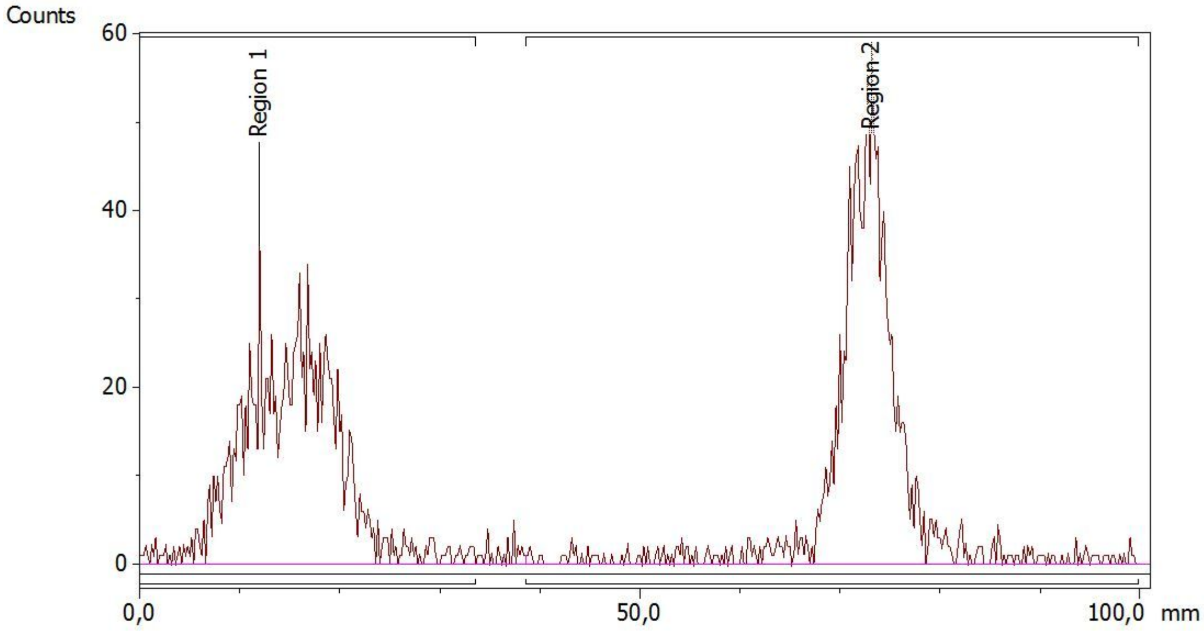

Figure S156: Radio-TLC chromatogram of [^{18}F]H_2N-Glu-(SiFA)SeFe-Gly-Lys-OH (3X_1) at pH 7.4, 37 °C, t = 60 min (flow agent: 60% MeCN/ 40% PBS (6/4 v/v) with 10% NaOAc in H_2O (2 M) and 1% TFA, stationary phase: TLC Silica gel 60 F254 from Merck Millipore).

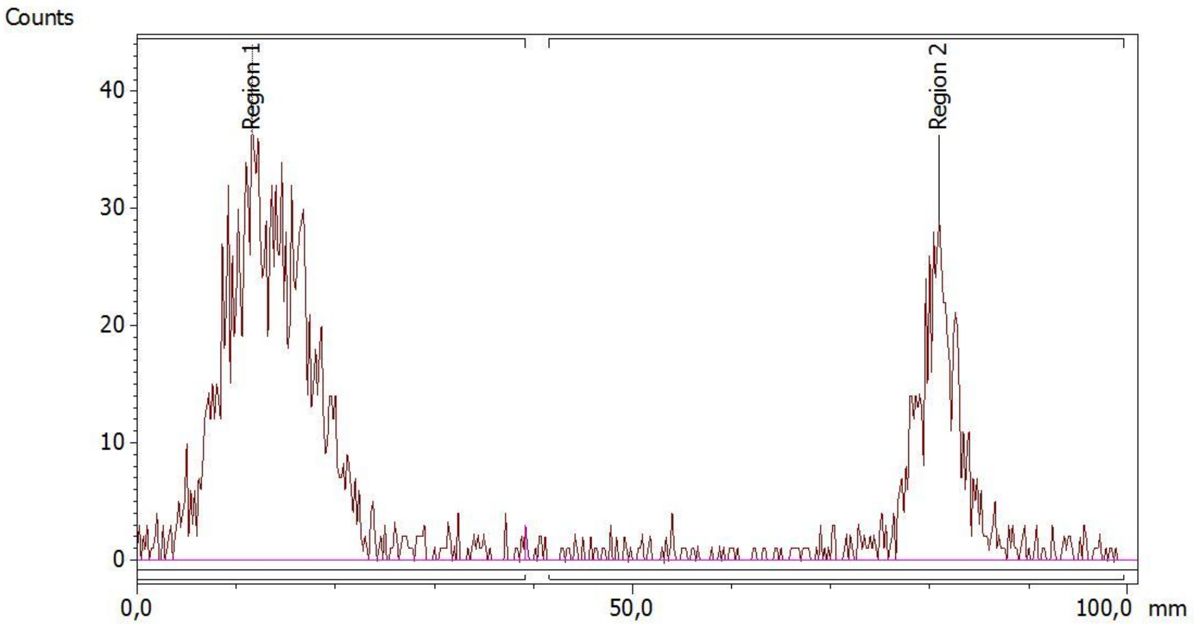

Figure S157: Radio-TLC chromatogram of [^{18}F]H_2N-Glu-(SiFA)SeFe-Gly-Lys-OH (3X_1) at pH 7.4, 37 °C, t = 90 min (flow agent: 60% MeCN/ 40% PBS (6/4 v/v) with 10% NaOAc in H_2O (2 M) and 1% TFA, stationary phase: TLC Silica gel 60 F254 from Merck Millipore).

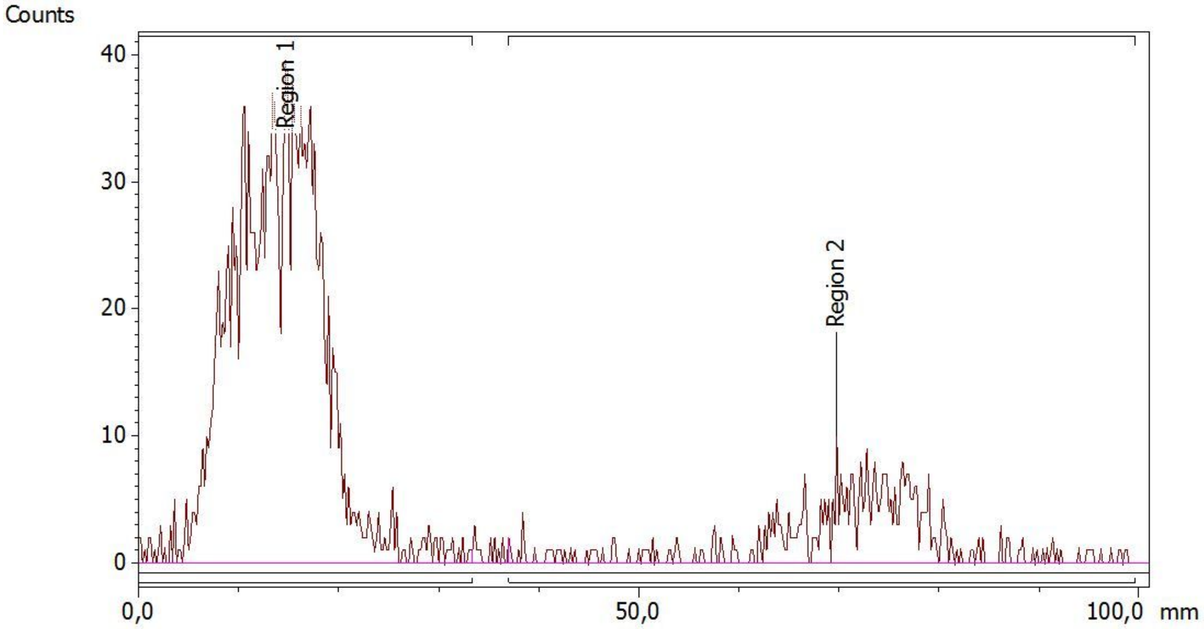

Figure S158: Radio-TLC chromatogram of [^{18}F]H_2N-Glu-(SiFA)SeFe-Gly-Lys-OH ($3X_1$) at pH 7.4, 37 °C, t = 120 min (flow agent: 60% MeCN/ 40% PBS (6/4 v/v) with 10% NaOAc in H_2O (2 M) and 1% TFA, stationary phase: TLC Silica gel 60 F254 from Merck Millipore).

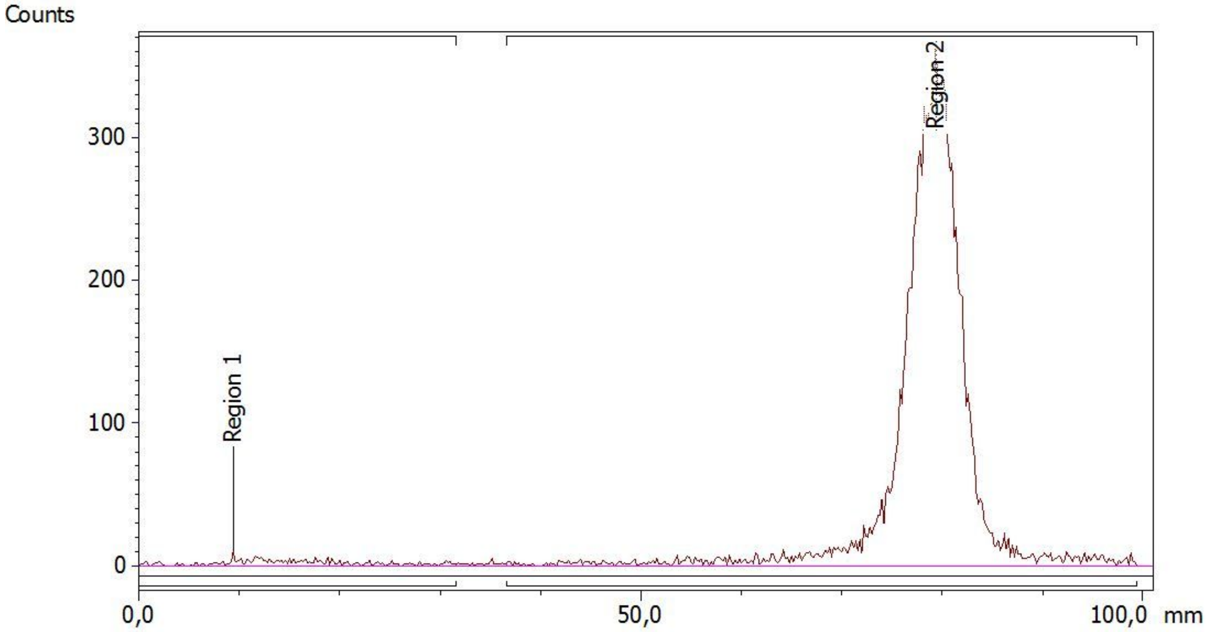

Figure S159: Radio-TLC chromatogram of [^{18}F]H_2N-(SiFA)SeFe-Gly-Asp-OH ($1X_2$) at pH 7.4, 37 °C, t = 0 min (flow agent: 60% MeCN/ 40% PBS (6/4 v/v) with 10% NaOAc in H_2O (2 M) and 1% TFA, stationary phase: TLC Silica gel 60 F254 from Merck Millipore).

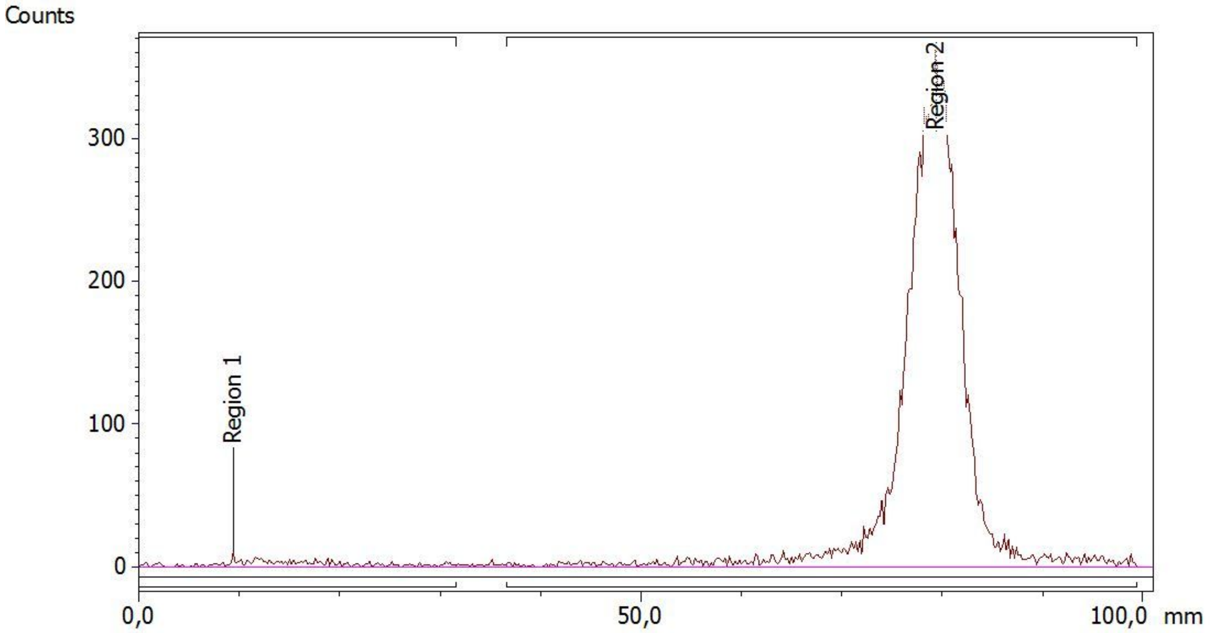

Figure S160: Radio-TLC chromatogram of [^{18}F]H_2N-(SiFA)SeFe-Gly-Asp-OH ($1X_2$) at pH 7.4, 37 °C, t = 30 min (flow agent: 60% MeCN/ 40% PBS (6/4 v/v) with 10% NaOAc in H_2O (2 M) and 1% TFA, stationary phase: TLC Silica gel 60 F254 from Merck Millipore).

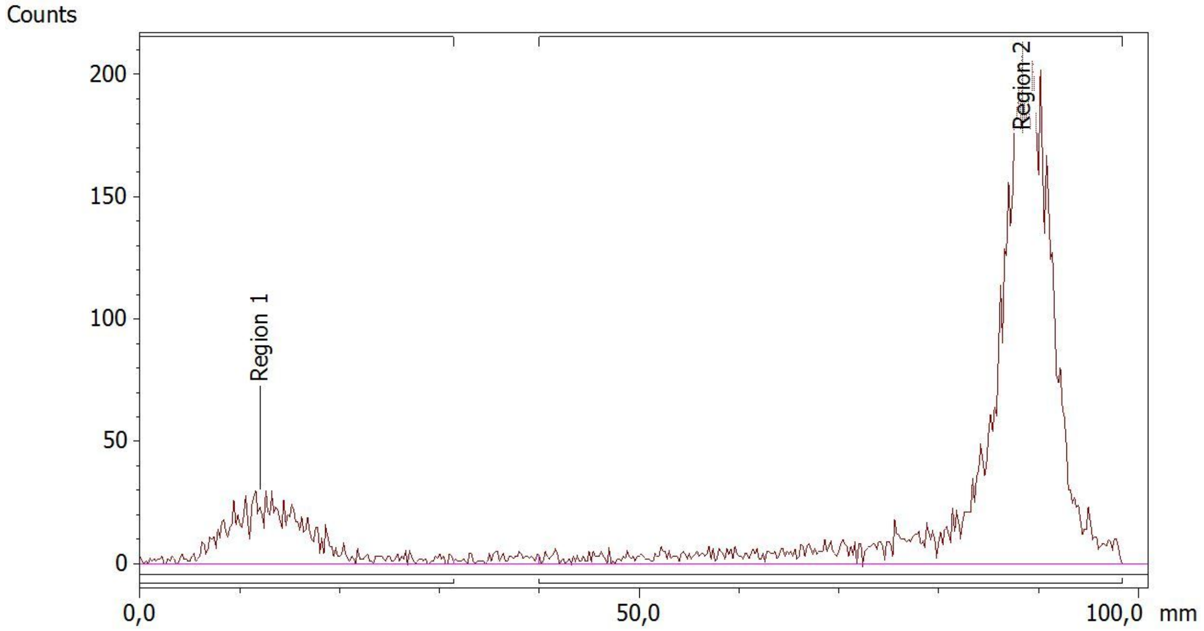

Figure S161: Radio-TLC chromatogram of [^{18}F]H_2N-(SiFA)SeFe-Gly-Asp-OH ($1X_2$) at pH 7.4, 37 °C, t = 60 min (flow agent: 60% MeCN/ 40% PBS (6/4 v/v) with 10% NaOAc in H_2O (2 M) and 1% TFA, stationary phase: TLC Silica gel 60 F254 from Merck Millipore).

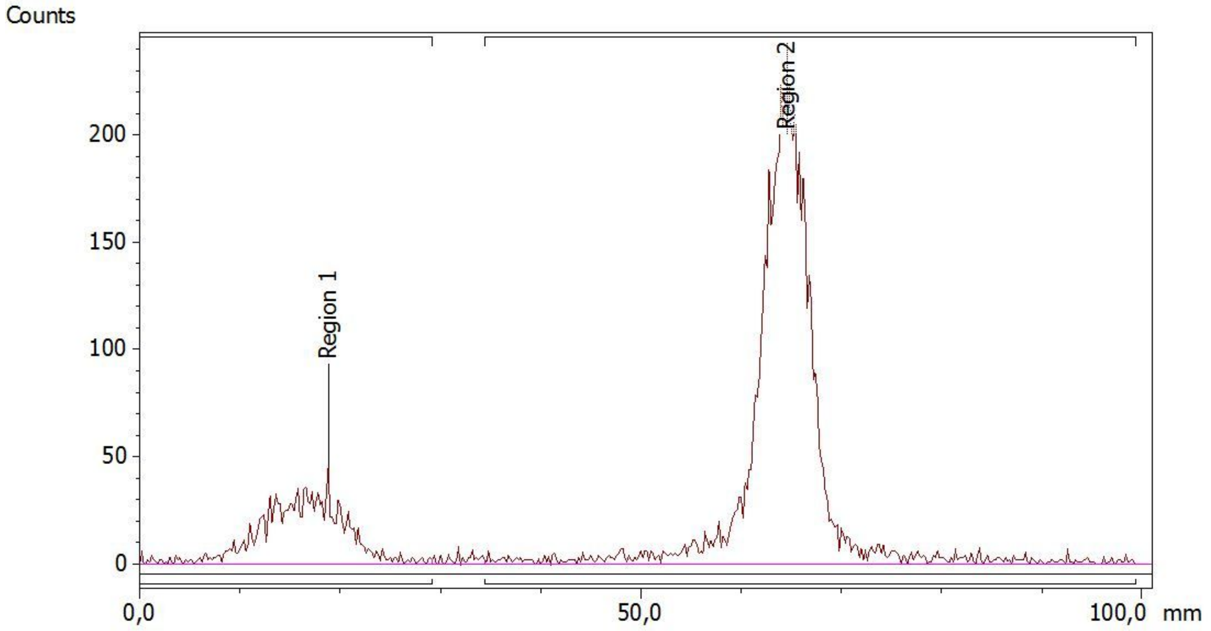

Figure S162: Radio-TLC chromatogram of [^{18}F]H_2N-(SiFA)SeFe-Gly-Asp-OH (1X_2) at pH 7.4, 37 °C, t = 90 min (flow agent: 60% MeCN/ 40% PBS (6/4 v/v) with 10% NaOAc in H_2O (2 M) and 1% TFA, stationary phase: TLC Silica gel 60 F254 from Merck Millipore).

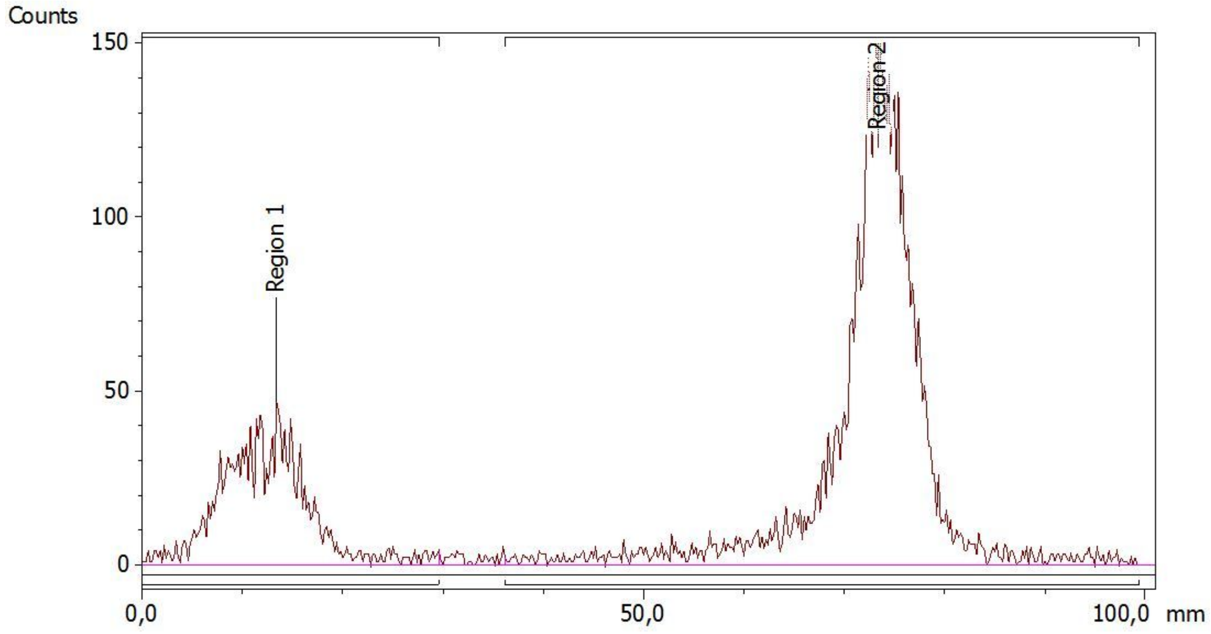

Figure S163: Radio-TLC chromatogram of [^{18}F]H_2N-(SiFA)SeFe-Gly-Asp-OH (1X_2) at pH 7.4, 37 °C, t = 120 min (flow agent: 60% MeCN/ 40% PBS (6/4 v/v) with 10% NaOAc in H_2O (2 M) and 1% TFA, stationary phase: TLC Silica gel 60 F254 from Merck Millipore).

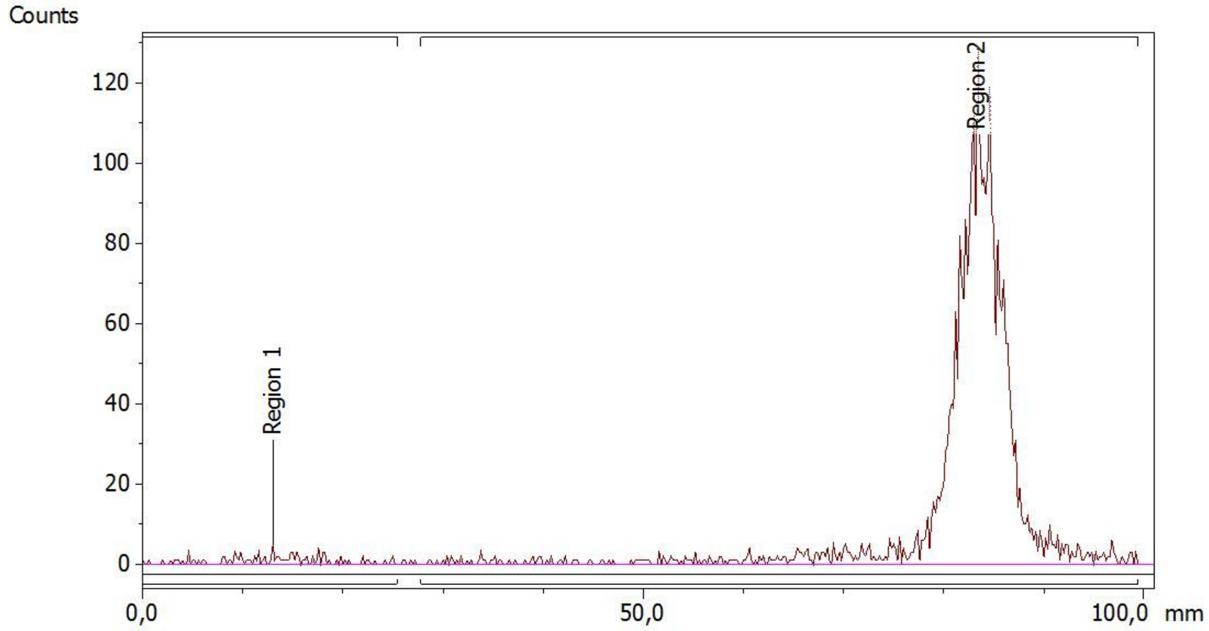

Figure S164: Radio-TLC chromatogram of [^{18}F]H$_2$N-Lys-(SiFA)SeFe-Gly-Asp-OH (2X$_2$) at pH 7.4, 37 °C, t = 0 min (flow agent: 60% MeCN/ 40% PBS (6/4 v/v) with 10% NaOAc in H_2O (2 M) and 1% TFA, stationary phase: TLC Silica gel 60 F254 from Merck Millipore).

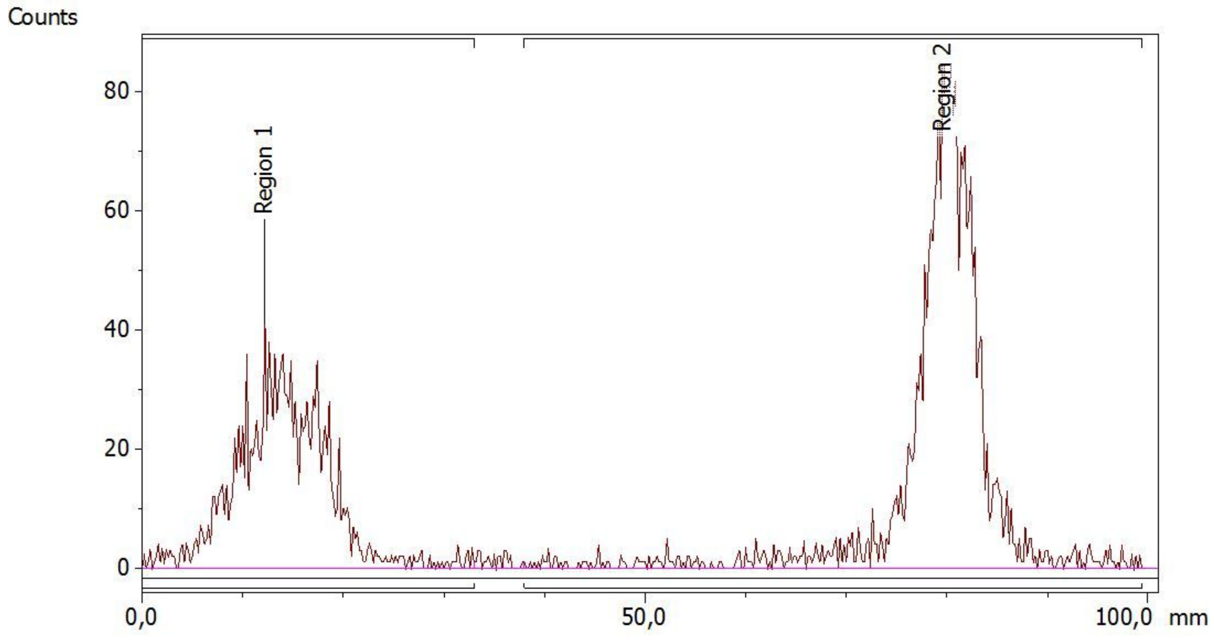

Figure S165: Radio-TLC chromatogram of [^{18}F]H$_2$N-Lys-(SiFA)SeFe-Gly-Asp-OH (2X$_2$) at pH 7.4, 37 °C, t = 30 min (flow agent: 60% MeCN/ 40% PBS (6/4 v/v) with 10% NaOAc in H_2O (2 M) and 1% TFA, stationary phase: TLC Silica gel 60 F254 from Merck Millipore).

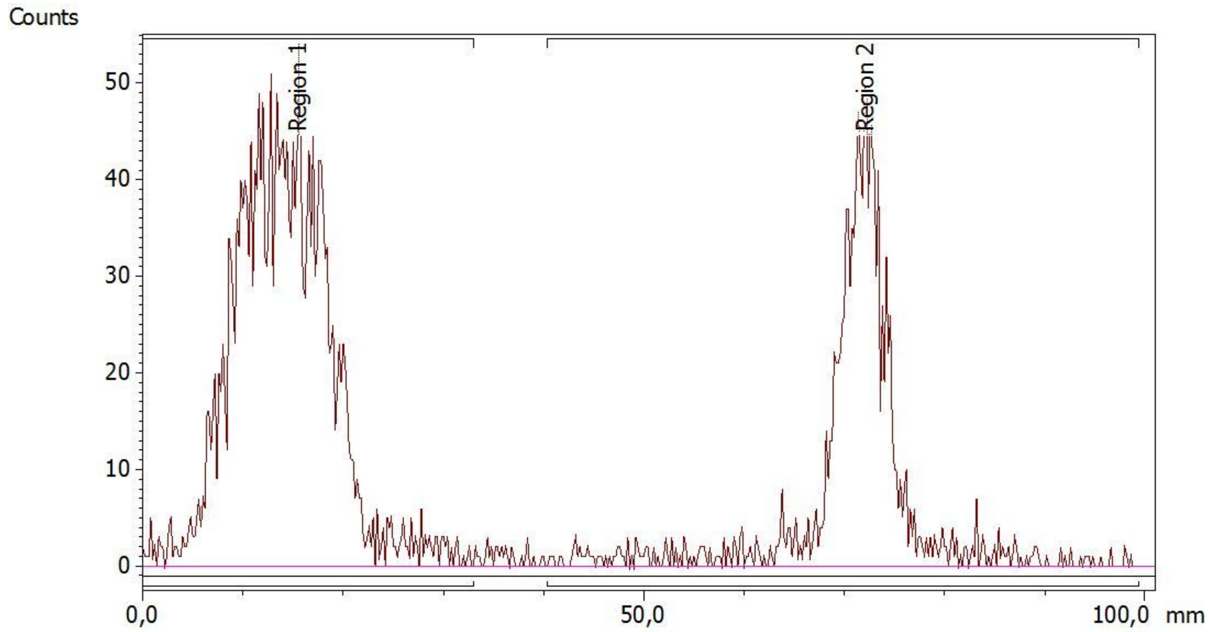

Figure S166: Radio-TLC chromatogram of [^{18}F]H_2N-Lys-(SiFA)SeFe-Gly-Asp-OH ($2X_2$) at pH 7.4, 37 °C, t= 60 min (flow agent: 60% MeCN/ 40% PBS (6/4 v/v) with 10% NaOAc in H_2O (2 M) and 1% TFA, stationary phase: TLC Silica gel 60 F254 from Merck Millipore).

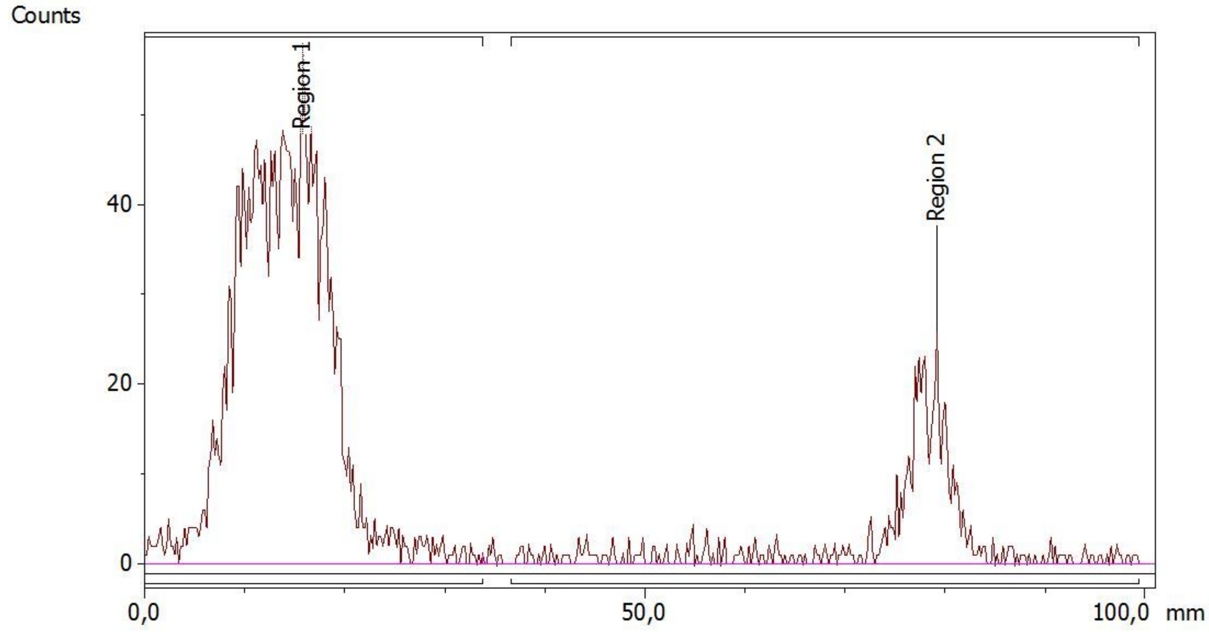

Figure S167: Radio-TLC chromatogram of [^{18}F]H_2N-Lys-(SiFA)SeFe-Gly-Asp-OH ($2X_2$) at pH 7.4, 37 °C, t = 90 min (flow agent: 60% MeCN/ 40% PBS (6/4 v/v) with 10% NaOAc in H_2O (2 M) and 1% TFA, stationary phase: TLC Silica gel 60 F254 from Merck Millipore).

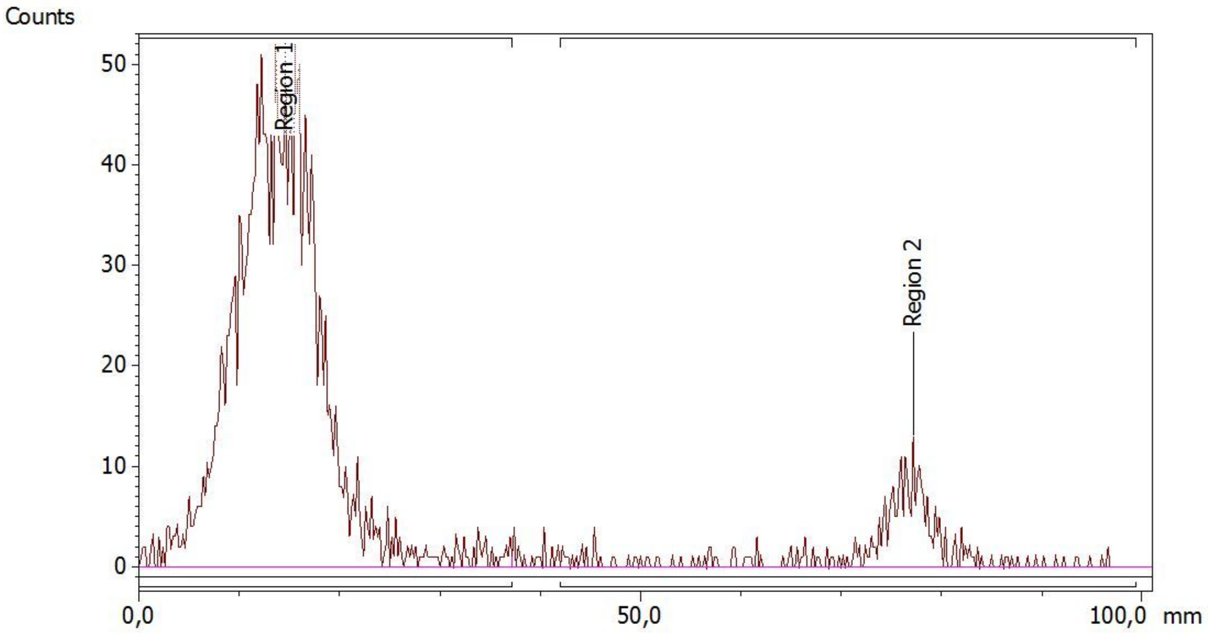

Figure S168: Radio-TLC chromatogram of [^{18}F]H$_2$N-Lys-(SiFA)SeFe-Gly-Asp-OH (2X$_2$) at pH 7.4, 37 °C, t = 120 min (flow agent: 60% MeCN/ 40% PBS (6/4 v/v) with 10% NaOAc in H_2O (2 M) and 1% TFA, stationary phase: TLC Silica gel 60 F254 from Merck Millipore).

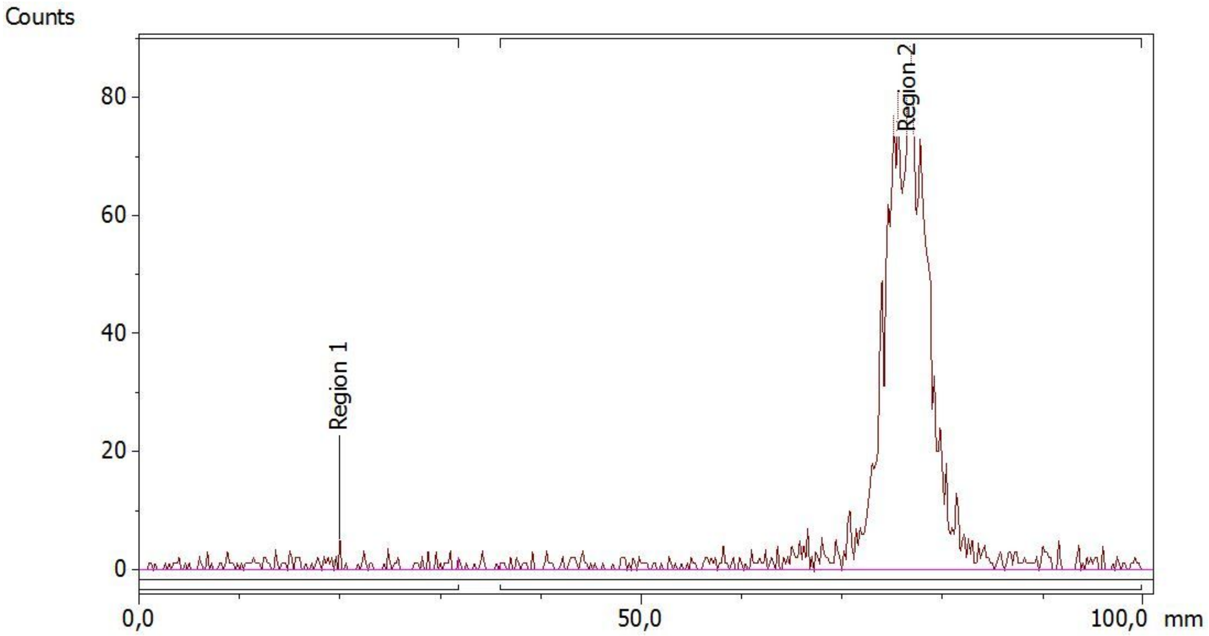

Figure S169: Radio-TLC chromatogram of [^{18}F]H$_2$N-Glu-(SiFA)SeFe-Gly-Asp-OH (3X$_2$) at pH 7.4, 37 °C, t = 0 min (flow agent: 60% MeCN/ 40% PBS (6/4 v/v) with 10% NaOAc in H_2O (2 M) and 1% TFA, stationary phase: TLC Silica gel 60 F254 from Merck Millipore).

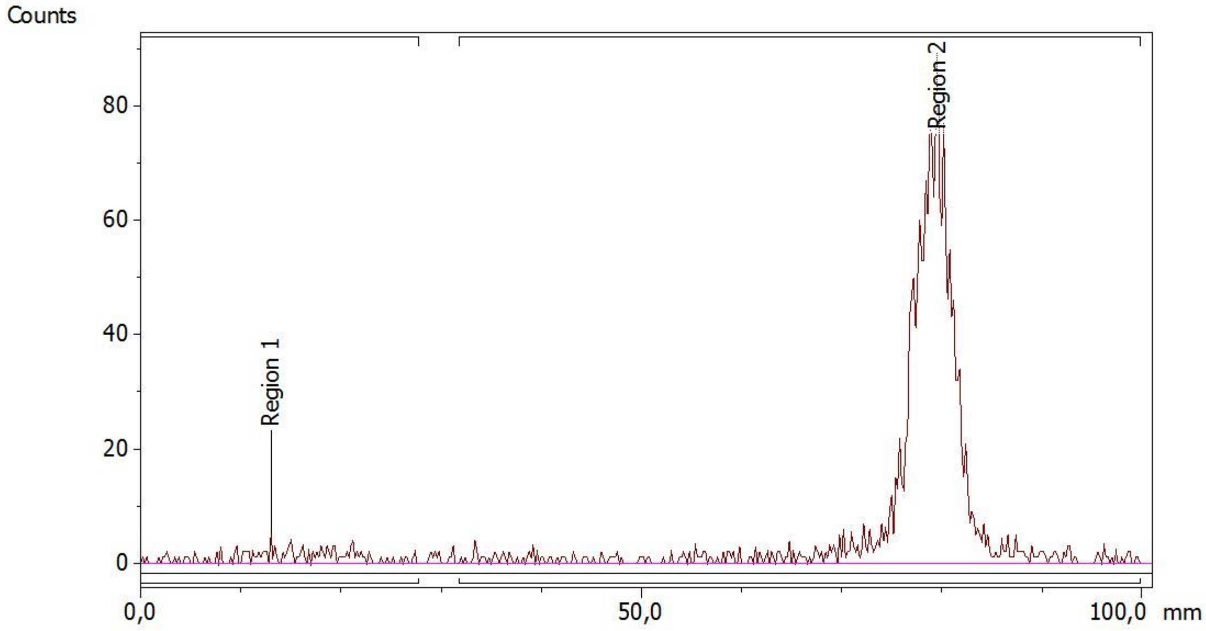

Figure S170: Radio-TLC chromatogram of [^{18}F]H$_2$N-Glu-(SiFA)SeFe-Gly-Asp-OH (3X$_2$) at pH 7.4, 37 °C, t = 30 min (flow agent: 60% MeCN/ 40% PBS (6/4 v/v) with 10% NaOAc in H_2O (2 M) and 1% TFA, stationary phase: TLC Silica gel 60 F254 from Merck Millipore).

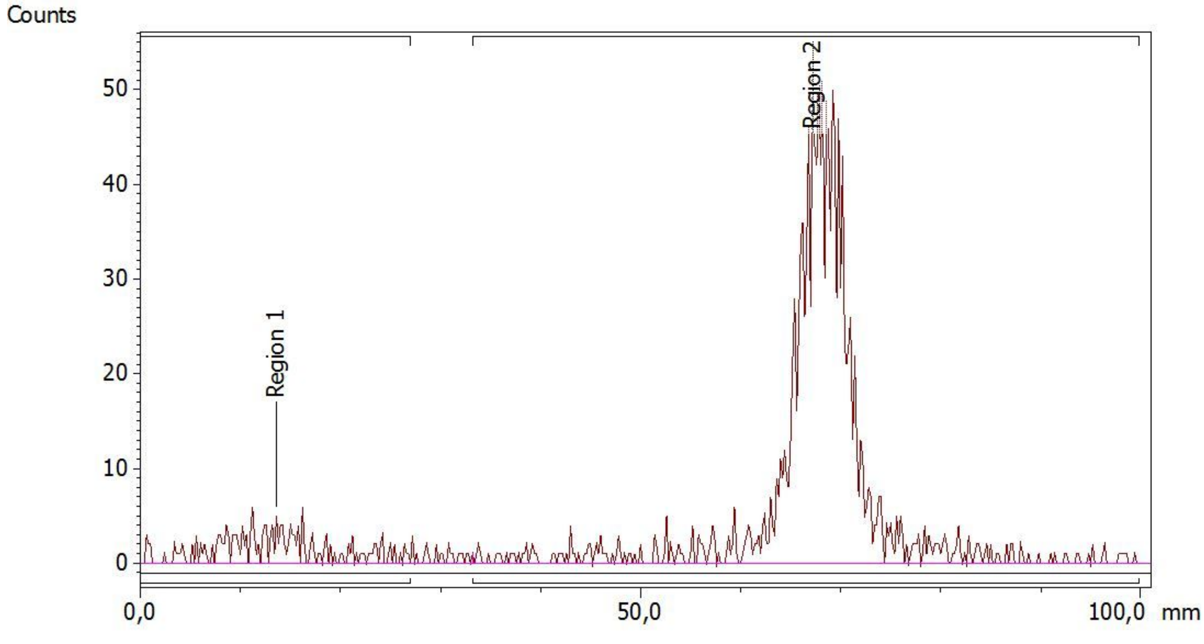

Figure S171: Radio-TLC chromatogram of [^{18}F]H$_2$N-Glu-(SiFA)SeFe-Gly-Asp-OH (3X$_2$) at pH 7.4, 37 °C, t = 60 min (flow agent: 60% MeCN/ 40% PBS (6/4 v/v) with 10% NaOAc in H_2O (2 M) and 1% TFA, stationary phase: TLC Silica gel 60 F254 from Merck Millipore).

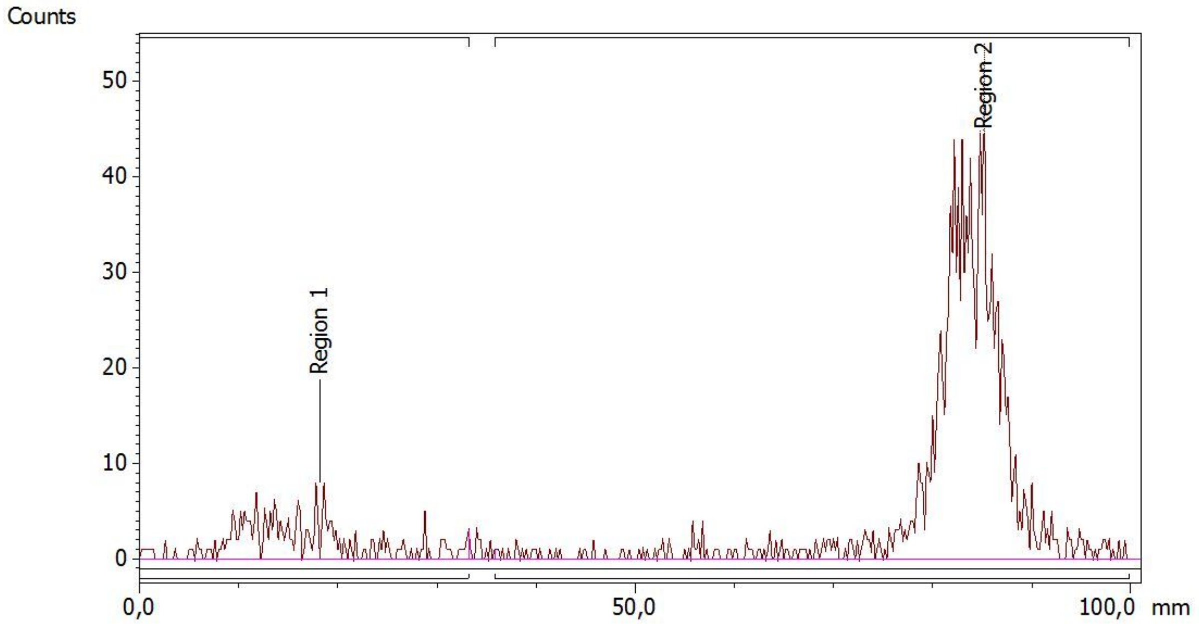

Figure S172: Radio-TLC chromatogram of [^{18}F]H$_2$N-Glu-(SiFA)SeFe-Gly-Asp-OH (3X$_2$) at pH 7.4, 37 °C, t = 90 min (flow agent: 60% MeCN/ 40% PBS (6/4 v/v) with 10% NaOAc in H$_2$O (2 M) and 1% TFA, stationary phase: TLC Silica gel 60 F254 from Merck Millipore).

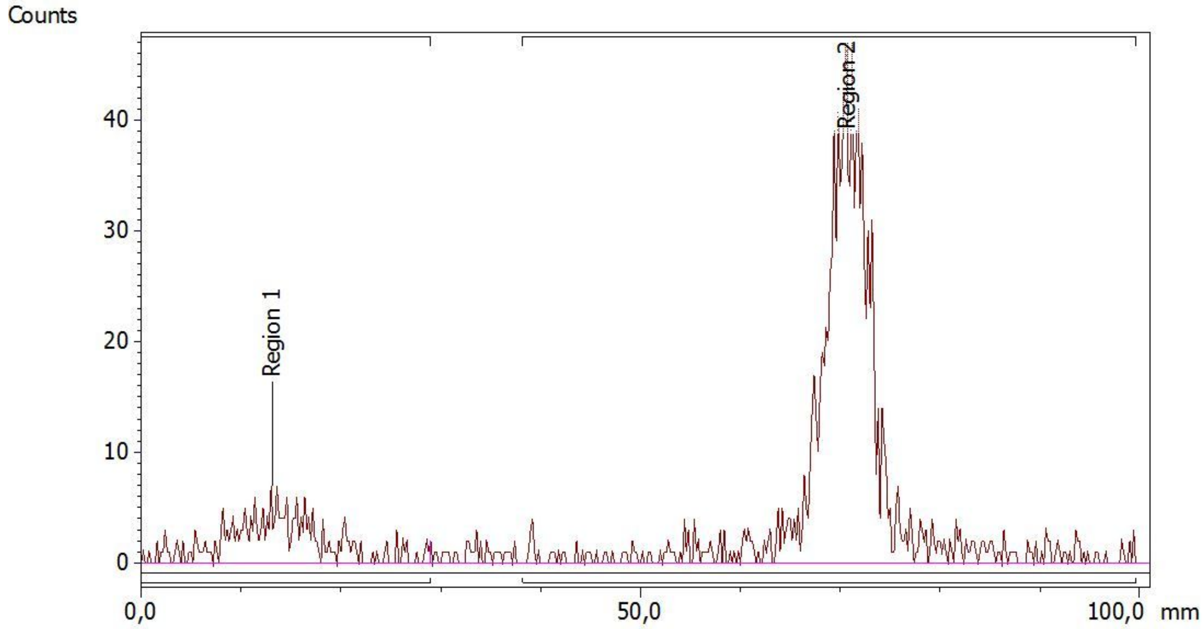

Figure S173: Radio-TLC chromatogram of [^{18}F]H$_2$N-Glu-(SiFA)SeFe-Gly-Asp-OH (3X$_2$) at pH 7.4, 37 °C, t = 120 min (flow agent: 60% MeCN/ 40% PBS (6/4 v/v) with 10% NaOAc in H$_2$O (2 M) and 1% TFA, stationary phase: TLC Silica gel 60 F254 from Merck Millipore).

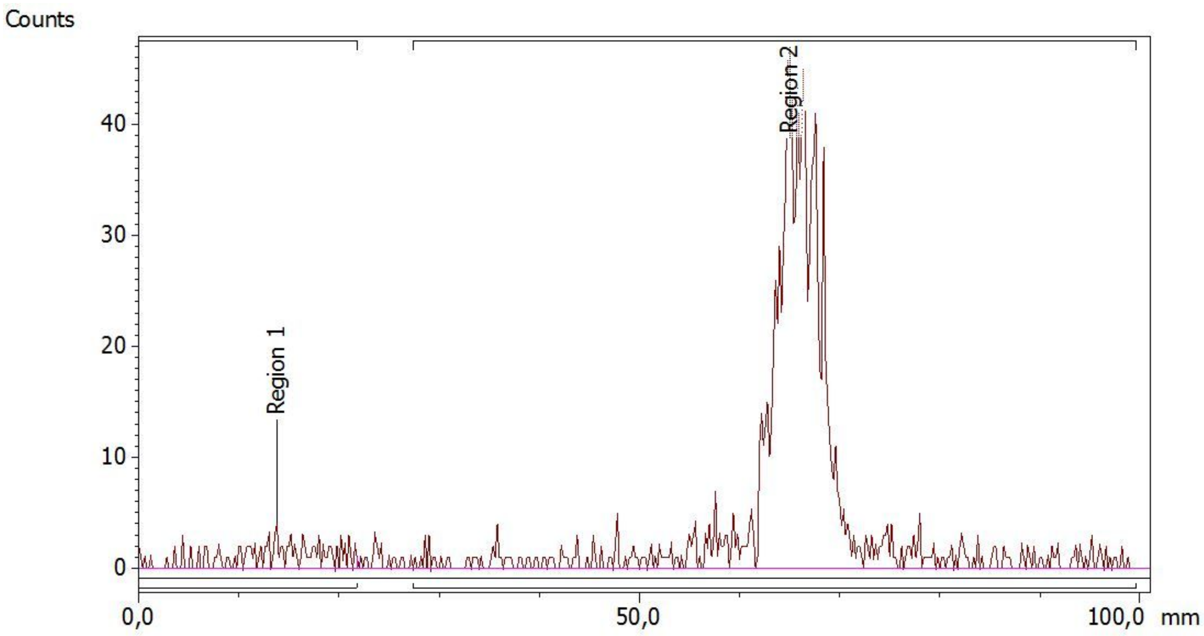

Figure S174: Radio-TLC chromatogram of [^{18}F]H_2N-(SiFA)SeFe-Gly-Tyr-OH ($1X_3$) at pH 7.4, 37 °C, t = 0 min (flow agent: 60% MeCN/ 40% PBS (6/4 v/v) with 10% NaOAc in H_2O (2 M) and 1% TFA, stationary phase: TLC Silica gel 60 F254 from Merck Millipore).

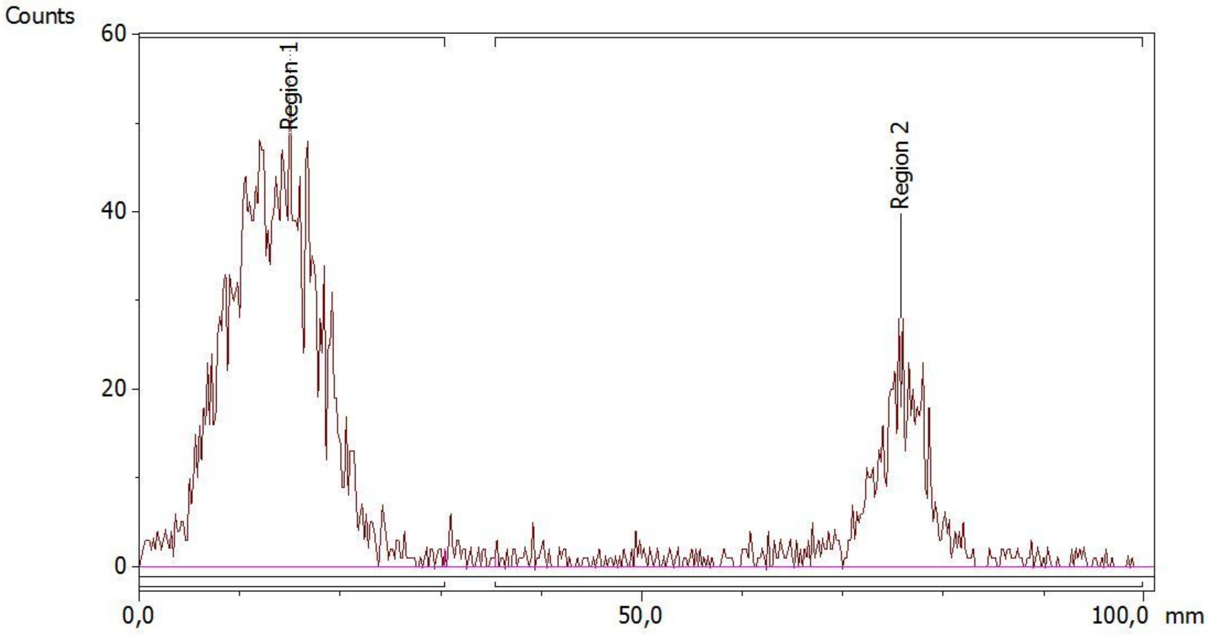

Figure S175: Radio-TLC chromatogram of [^{18}F]H_2N-(SiFA)SeFe-Gly-Tyr-OH ($1X_3$) at pH 7.4, 37 °C, t = 30 min (flow agent: 60% MeCN/ 40% PBS (6/4 v/v) with 10% NaOAc in H_2O (2 M) and 1% TFA, stationary phase: TLC Silica gel 60 F254 from Merck Millipore).

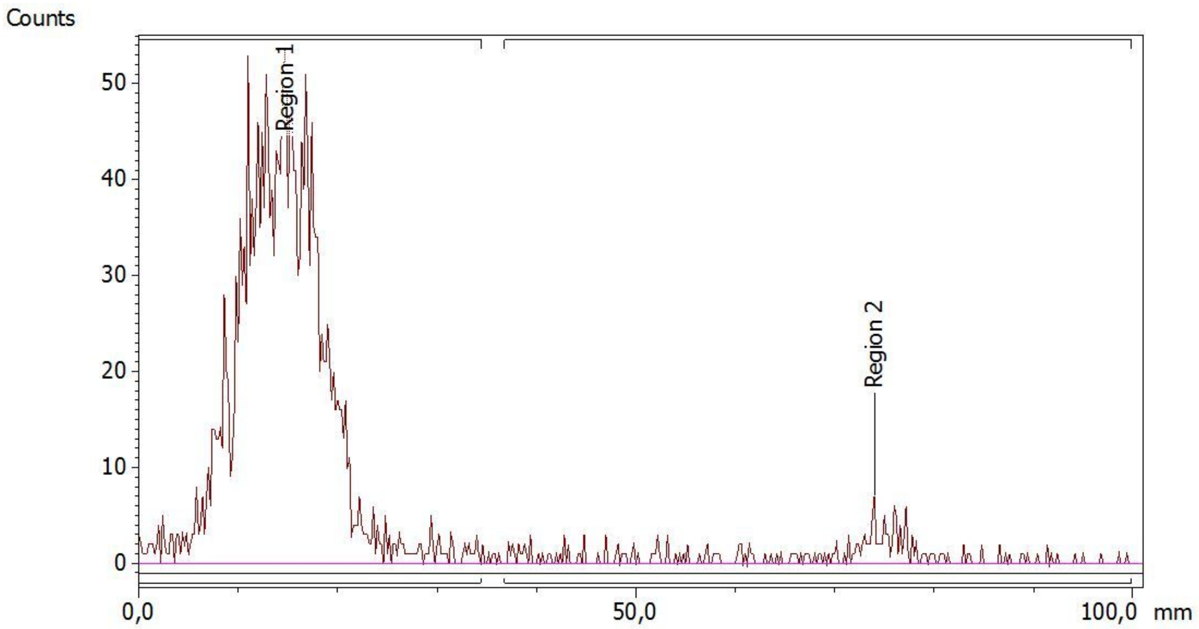

Figure S176: Radio-TLC chromatogram of [^{18}F]H_2N-(SiFA)SeFe-Gly-Tyr-OH ($1X_3$) at pH 7.4, 37 °C, t = 60 min (flow agent: 60% MeCN/ 40% PBS (6/4 v/v) with 10% NaOAc in H_2O (2 M) and 1% TFA, stationary phase: TLC Silica gel 60 F254 from Merck Millipore).

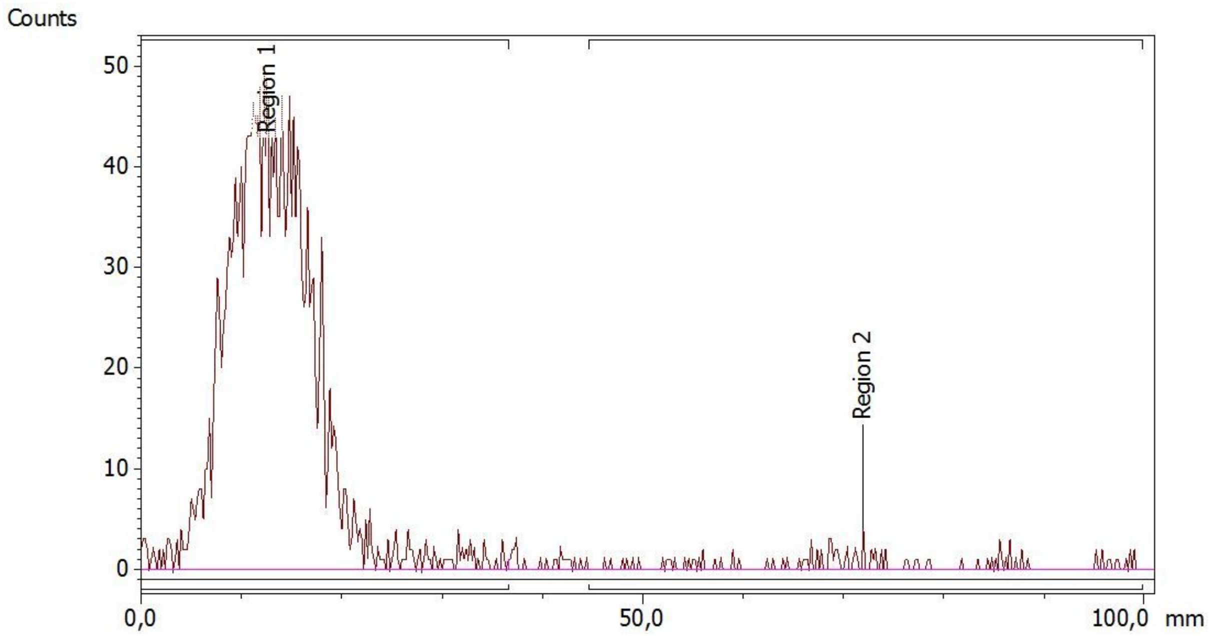

Figure S177: Radio-TLC chromatogram of [^{18}F]H_2N-(SiFA)SeFe-Gly-Tyr-OH ($1X_3$) at pH 7.4, 37 °C, t = 90 min (flow agent: 60% MeCN/ 40% PBS (6/4 v/v) with 10% NaOAc in H_2O (2 M) and 1% TFA, stationary phase: TLC Silica gel 60 F254 from Merck Millipore).

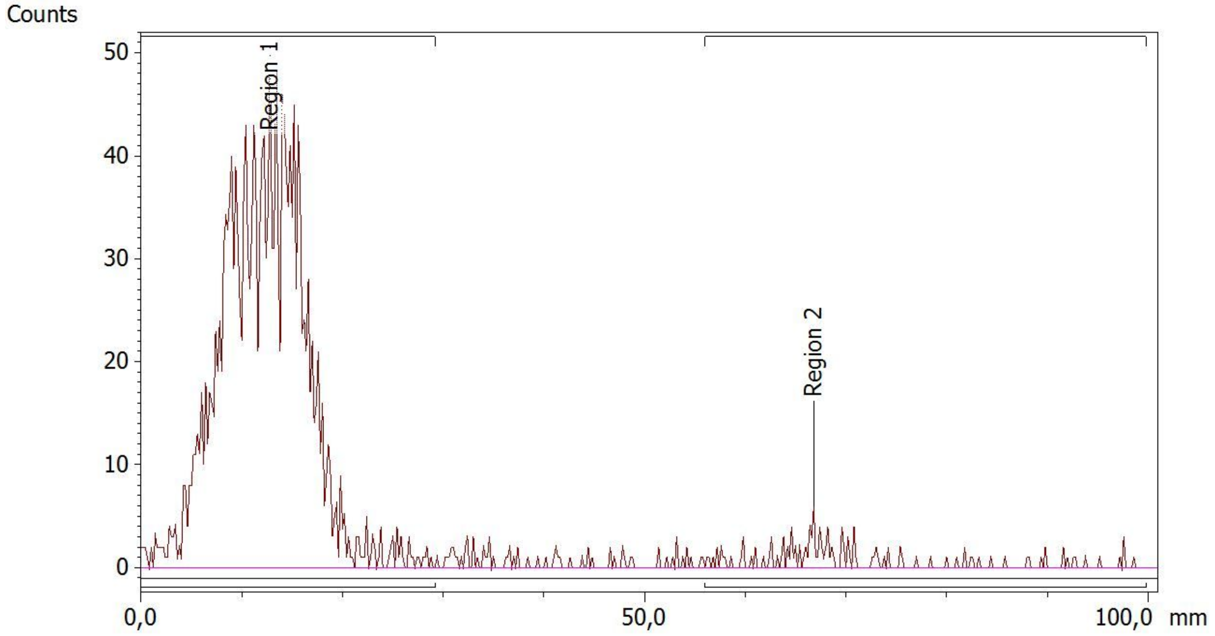

Figure S178: Radio-TLC chromatogram of [^{18}F]H_2N-(SiFA)SeFe-Gly-Tyr-OH ($1X_3$) at pH 7.4, 37 °C, t = 120 min (flow agent: 60% MeCN/ 40% PBS (6/4 v/v) with 10% NaOAc in H_2O (2 M) and 1% TFA, stationary phase: TLC Silica gel 60 F254 from Merck Millipore).

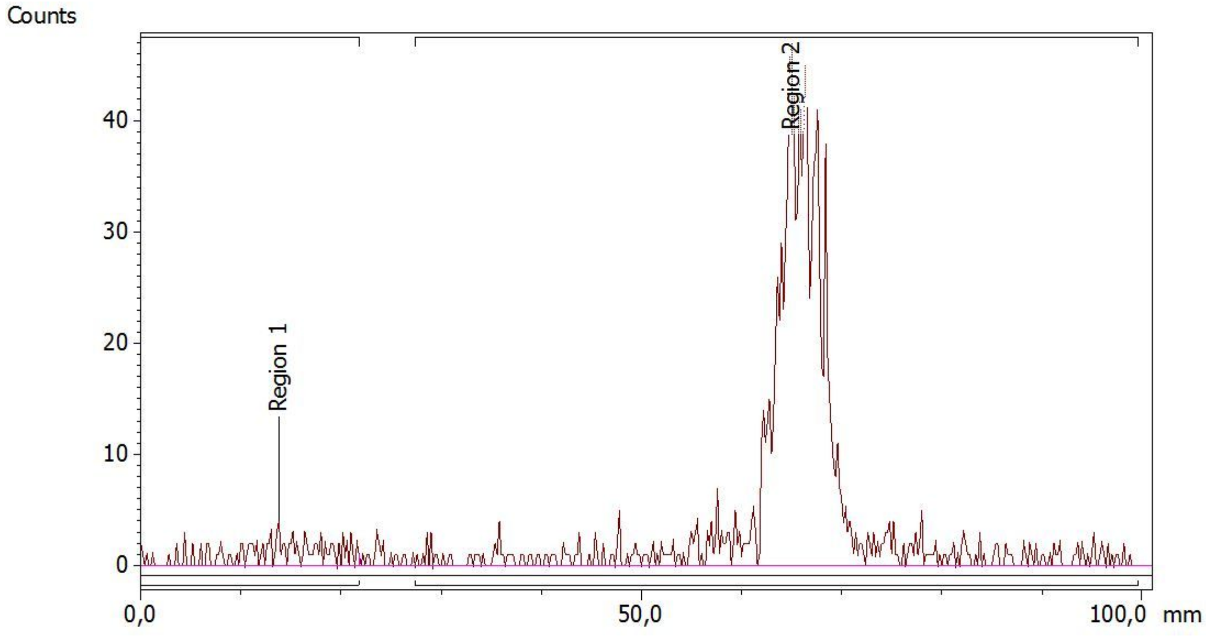

Figure S179: Radio-TLC chromatogram of [^{18}F]H_2N-Lys-(SiFA)SeFe-Gly-Tyr-OH ($2X_3$) at pH 7.4, 37 °C, t = 0 min (flow agent: 60% MeCN/ 40% PBS (6/4 v/v) with 10% NaOAc in H_2O (2 M) and 1% TFA, stationary phase: TLC Silica gel 60 F254 from Merck Millipore).

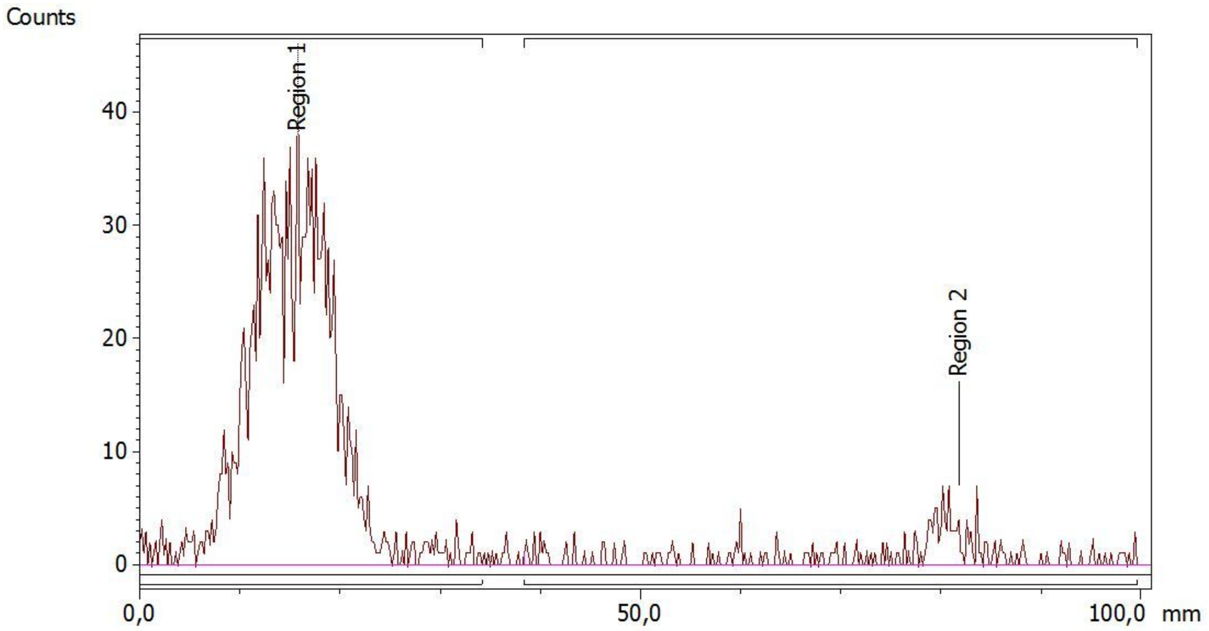

Figure S180: Radio-TLC chromatogram of [^{18}F]H_2N-Lys-(SiFA)SeFe-Gly-Tyr-OH ($2X_3$) at pH 7.4, 37 °C, t = 30 min (flow agent: 60% MeCN/ 40% PBS (6/4 v/v) with 10% NaOAc in H_2O (2 M) and 1% TFA, stationary phase: TLC Silica gel 60 F254 from Merck Millipore).

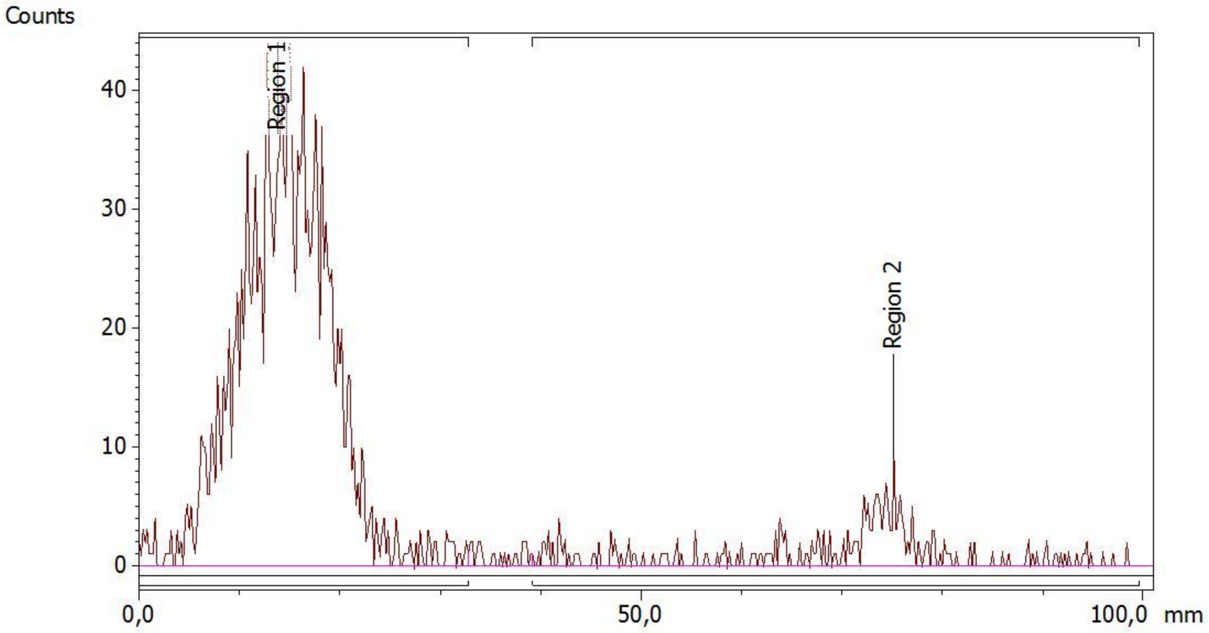

Figure S181: Radio-TLC chromatogram of [^{18}F]H_2N-Lys-(SiFA)SeFe-Gly-Tyr-OH ($2X_3$) at pH 7.4, 37 °C, t = 60 min (flow agent: 60% MeCN/ 40% PBS (6/4 v/v) with 10% NaOAc in H_2O (2 M) and 1% TFA, stationary phase: TLC Silica gel 60 F254 from Merck Millipore).

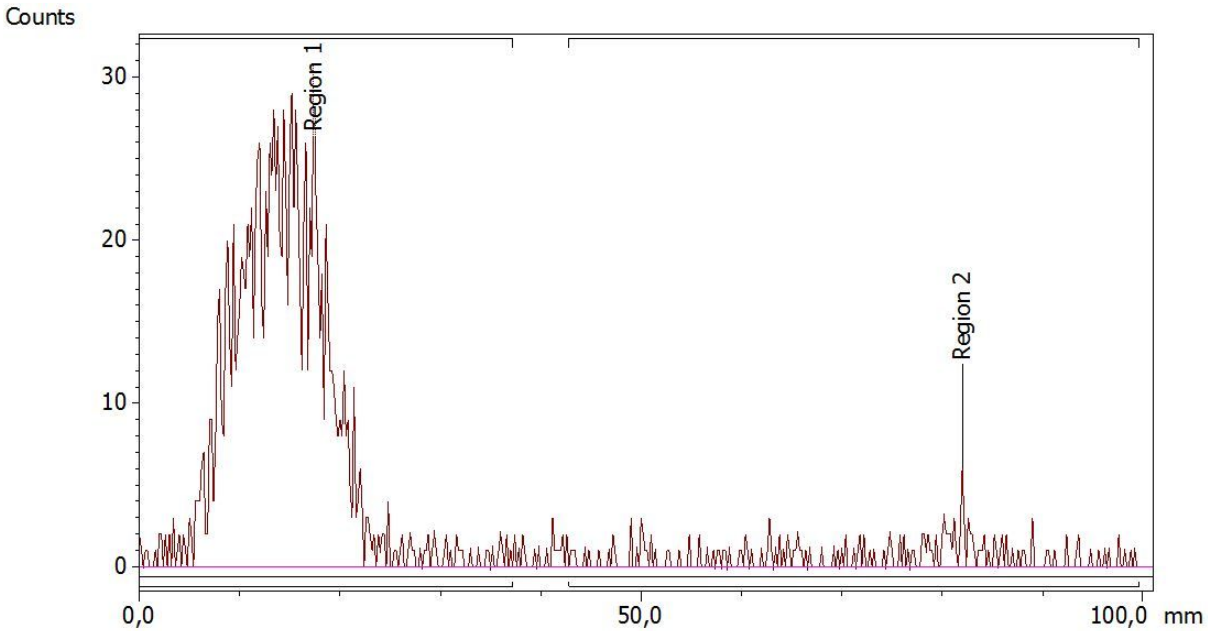

Figure S182: Radio-TLC chromatogram of [^{18}F]H$_2$N-Lys-(SiFA)SeFe-Gly-Tyr-OH (2X$_3$) at pH 7.4, 37 °C, t = 90 min (flow agent: 60% MeCN/ 40% PBS (6/4 v/v) with 10% NaOAc in H_2O (2 M) and 1% TFA, stationary phase: TLC Silica gel 60 F254 from Merck Millipore).

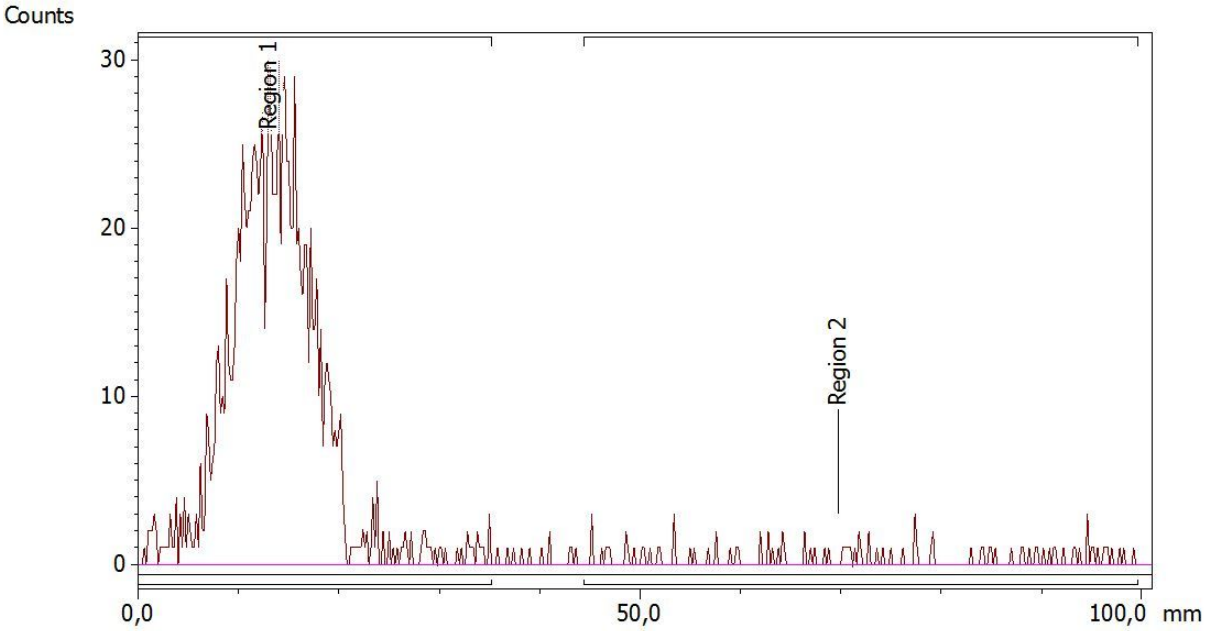

Figure S183: Radio-TLC chromatogram of [^{18}F]H$_2$N-Lys-(SiFA)SeFe-Gly-Tyr-OH (2X$_3$) at pH 7.4, 37 °C, t = 120 min (flow agent: 60% MeCN/ 40% PBS (6/4 v/v) with 10% NaOAc in H_2O (2 M) and 1% TFA, stationary phase: TLC Silica gel 60 F254 from Merck Millipore).

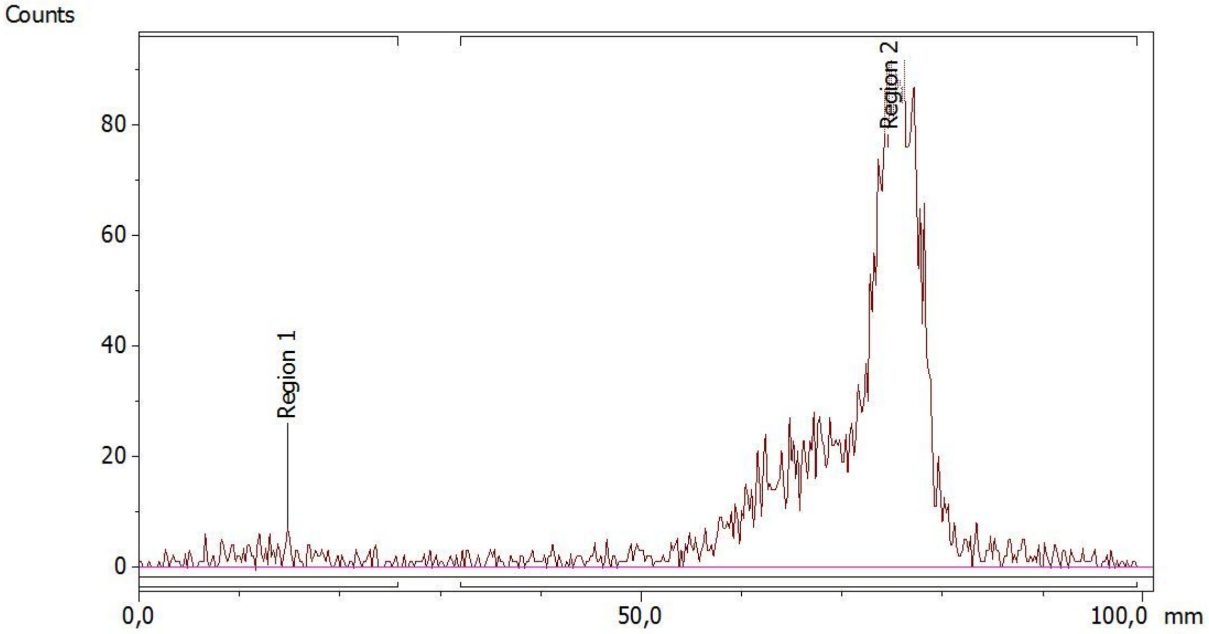

Figure S184: Radio-TLC chromatogram of [^{18}F]H_2N-Glu-(SiFA)SeFe-Gly-Tyr-OH ($3X_3$) at pH 7.4, 37 °C, t = 0 min (flow agent: 60% MeCN/ 40% PBS (6/4 v/v) with 10% NaOAc in H_2O (2 M) and 1% TFA, stationary phase: TLC Silica gel 60 F254 from Merck Millipore).

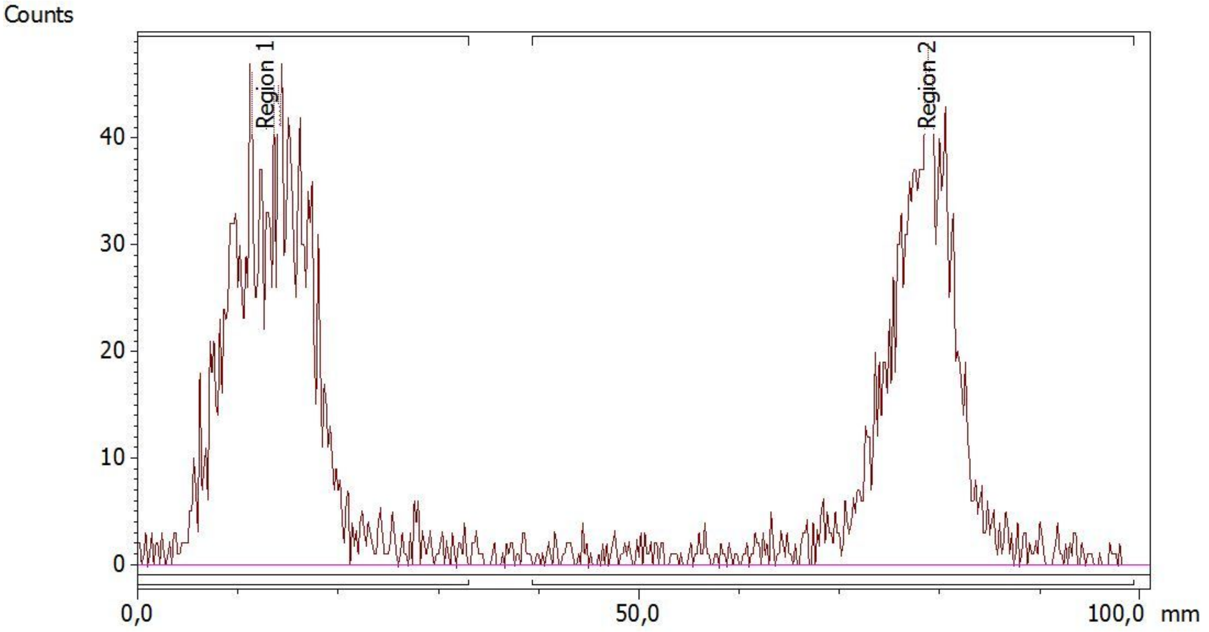

Figure S185: Radio-TLC chromatogram of [^{18}F]H_2N-Glu-(SiFA)SeFe-Gly-Tyr-OH ($3X_3$) at pH 7.4, 37 °C, t = 30 min (flow agent: 60% MeCN/ 40% PBS (6/4 v/v) with 10% NaOAc in H_2O (2 M) and 1% TFA, stationary phase: TLC Silica gel 60 F254 from Merck Millipore).

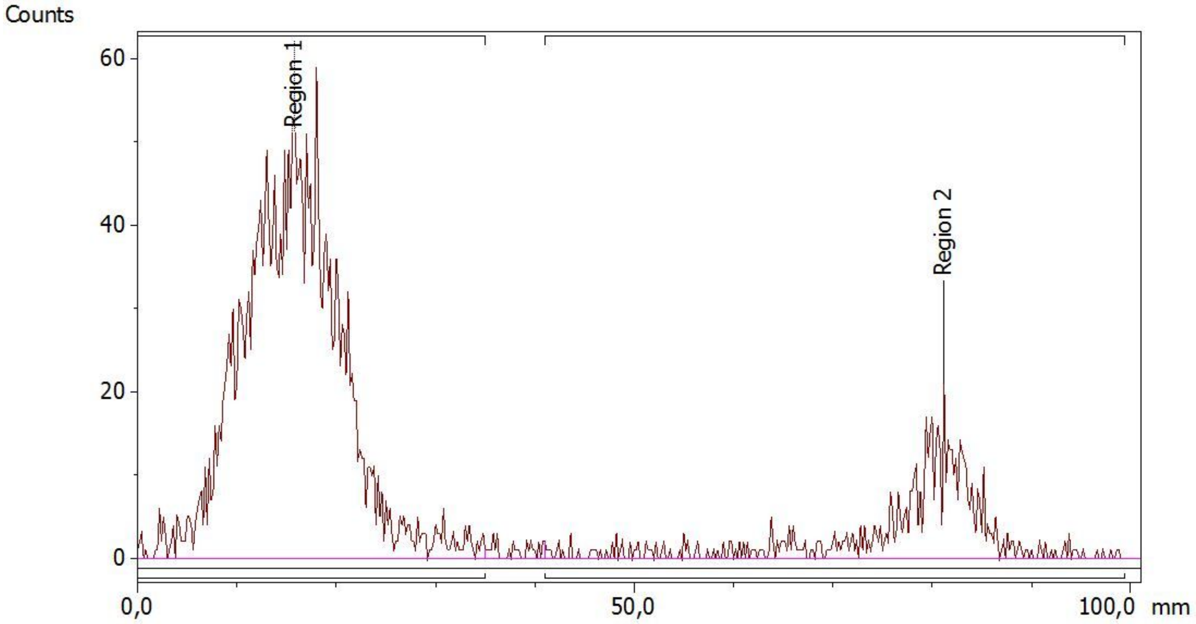

Figure S186: Radio-TLC chromatogram of [^{18}F]H_2N-Glu-(SiFA)SeFe-Gly-Tyr-OH ($3X_3$) at pH 7.4, 37 °C, t = 60 min (flow agent: 60% MeCN/ 40% PBS (6/4 v/v) with 10% NaOAc in H_2O (2 M) and 1% TFA, stationary phase: TLC Silica gel 60 F254 from Merck Millipore).

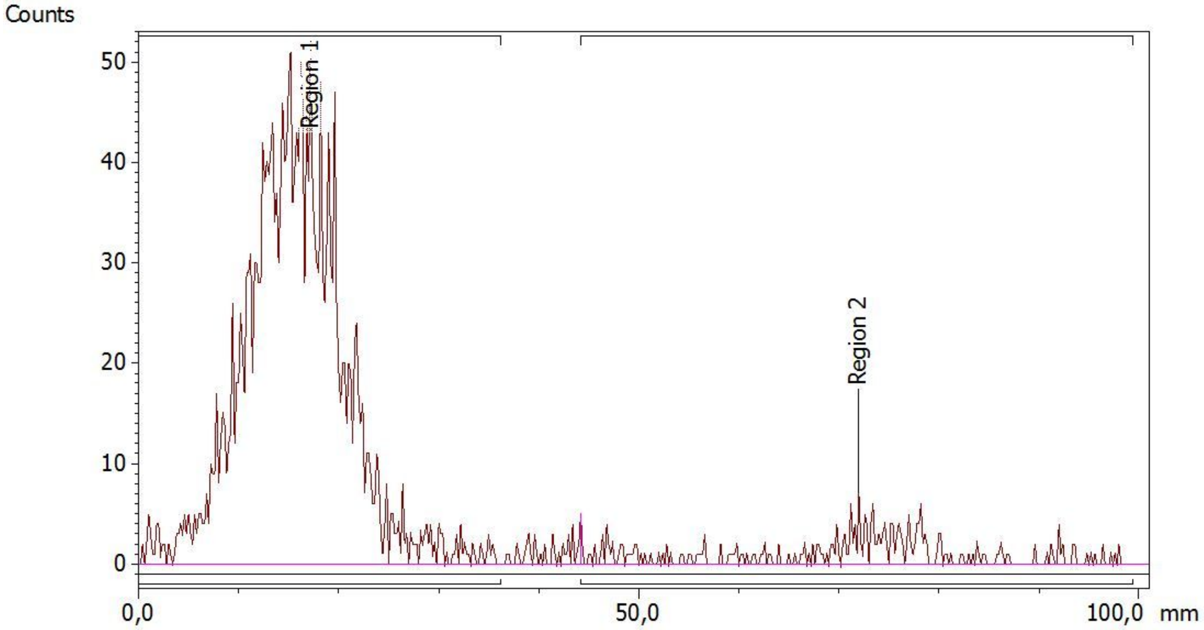

Figure S187: Radio-TLC chromatogram of [^{18}F]H_2N-Glu-(SiFA)SeFe-Gly-Tyr-OH ($3X_3$) at pH 7.4, 37 °C, t = 90 min (flow agent: 60% MeCN/ 40% PBS (6/4 v/v) with 10% NaOAc in H_2O (2 M) and 1% TFA, stationary phase: TLC Silica gel 60 F254 from Merck Millipore).

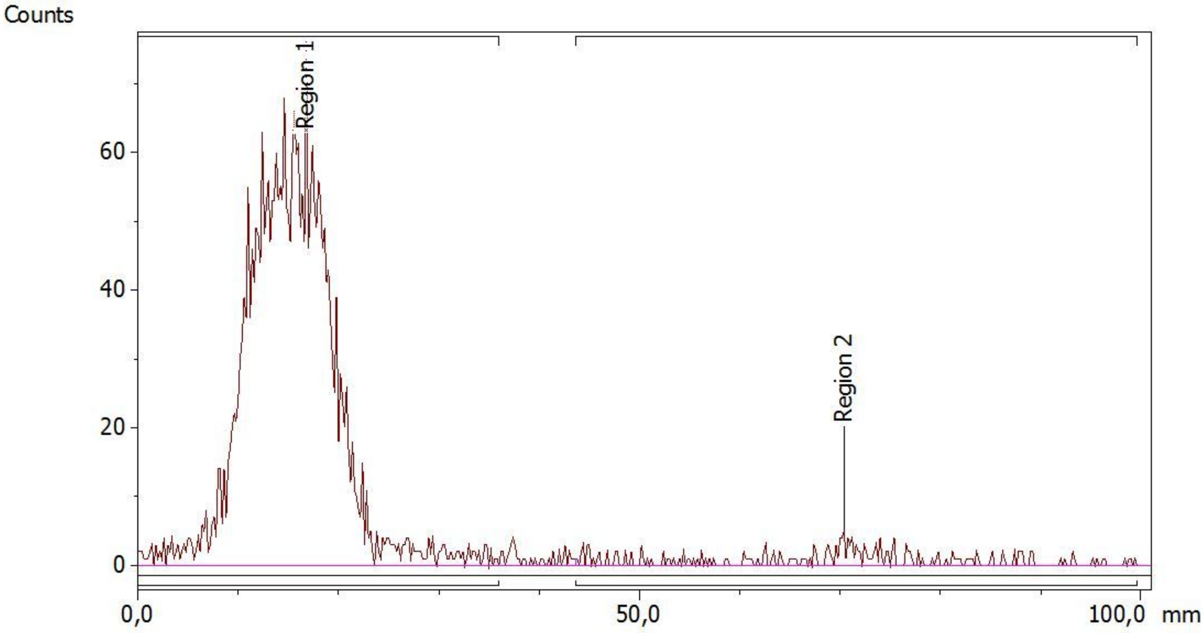

Figure S188: Radio-TLC chromatogram of [^{18}F]H_2N-Glu-(SiFA)SeFe-Gly-Tyr-OH ($3X_3$) at pH 7.4, 37 °C, t = 120 min (flow agent: 60% MeCN/ 40% PBS (6/4 v/v) with 10% NaOAc in H_2O (2 M) and 1% TFA, stationary phase: TLC Silica gel 60 F254 from Merck Millipore).

3. Characterization of the radiohybrid tracers and their benchmark compounds.

3.1. Synthesis of SST binding motif and SST-ligands

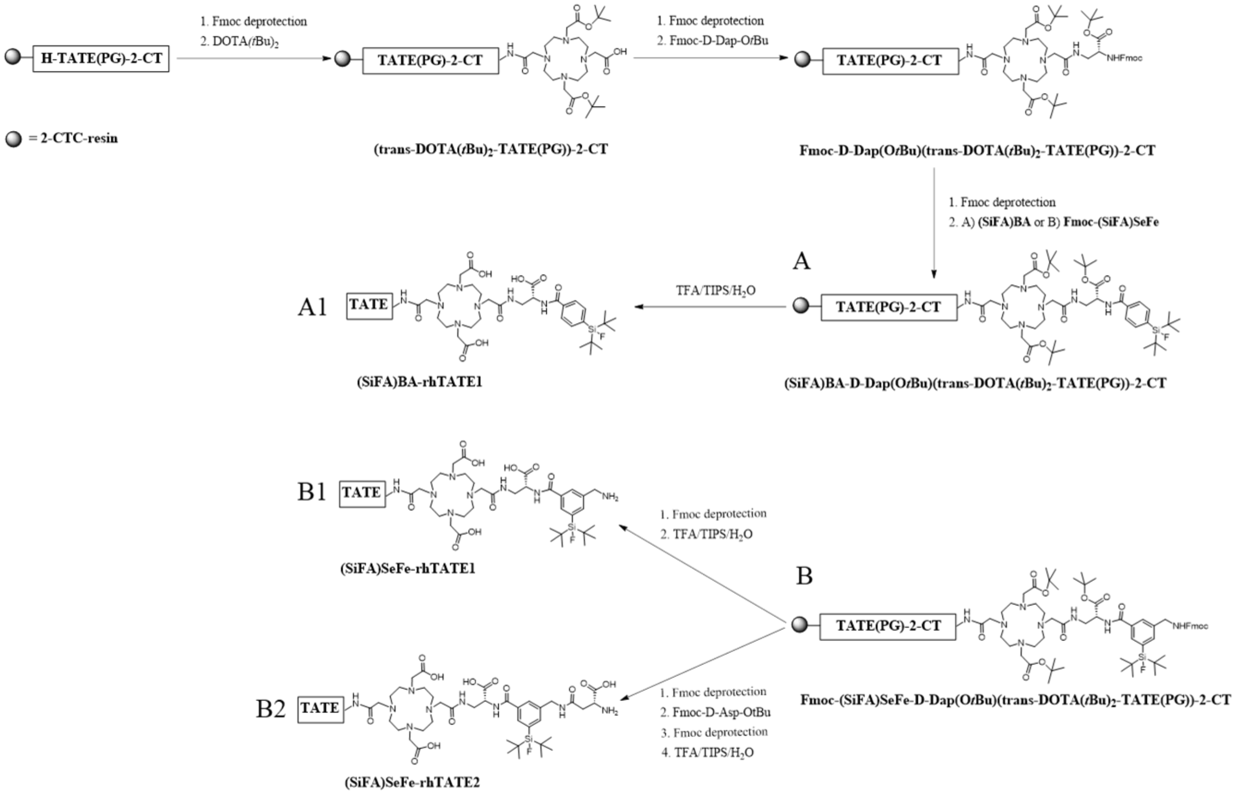

Scheme S1: General synthesis of diagnostic rh-compounds starting from the resin-bound binding motif H-TATE(PG)-2CT. The coupling of A) (SiFA)BA results in product A1: (SiFA)BA-rhTATE1. The coupling of B) Fmoc-(SiFA)SeFe results in the products B1: (SiFA)SeFe-rhTATE1 and B2: (SiFA)SeFe-rhTATE2.

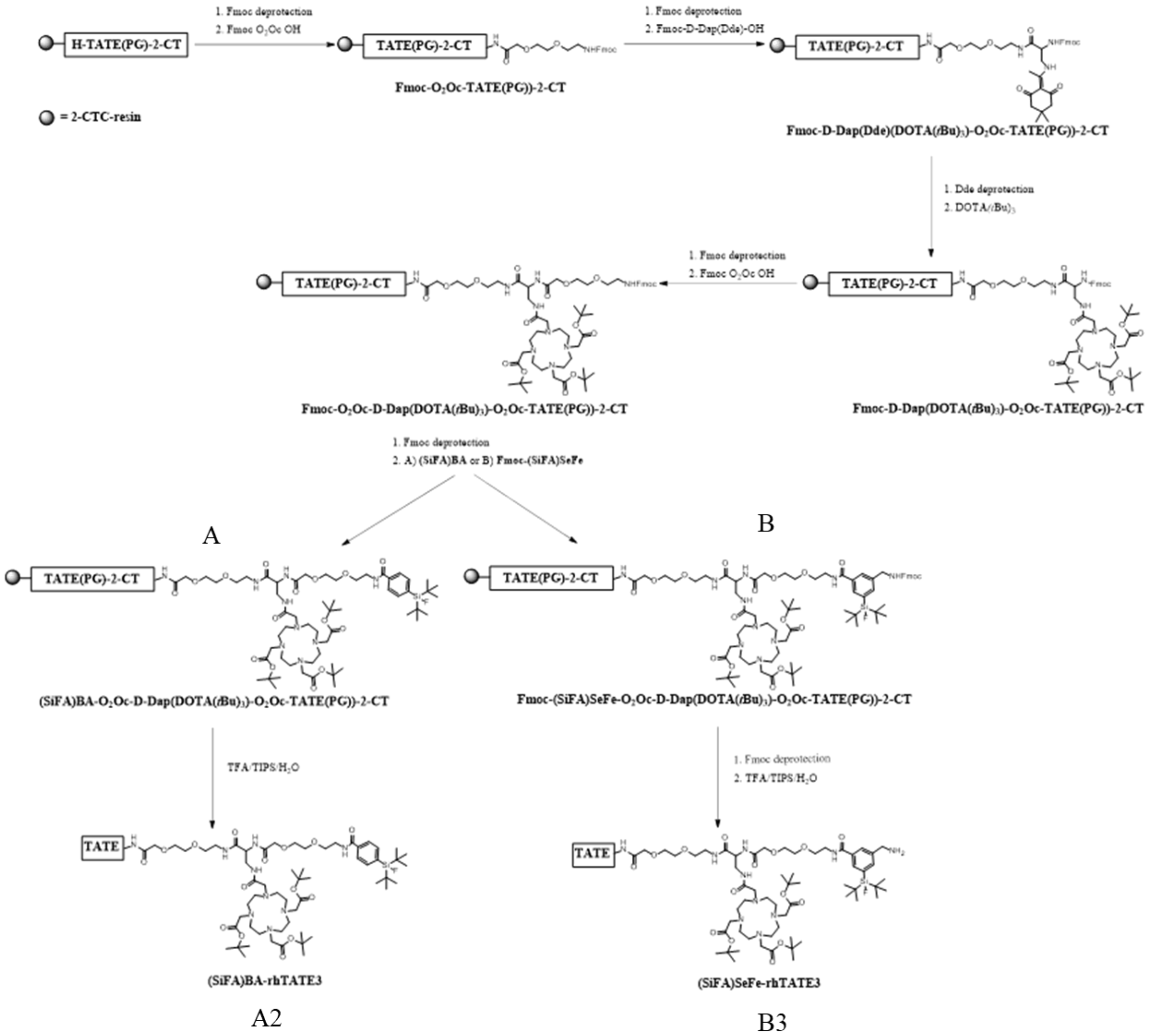

Scheme S2: General synthesis of theranostic rh-compounds starting from the resin-bound binding motif H-TATE(PG)-2CT. Bottom: The coupling of A (SiFA)BA results in product A2: (SiFA)BA-rhTATE3. The coupling of B Fmoc-(SiFA)SeFe results in the product B3: (SiFA)SeFe-rhTATE3.

3.2. RP-HPLC analysis

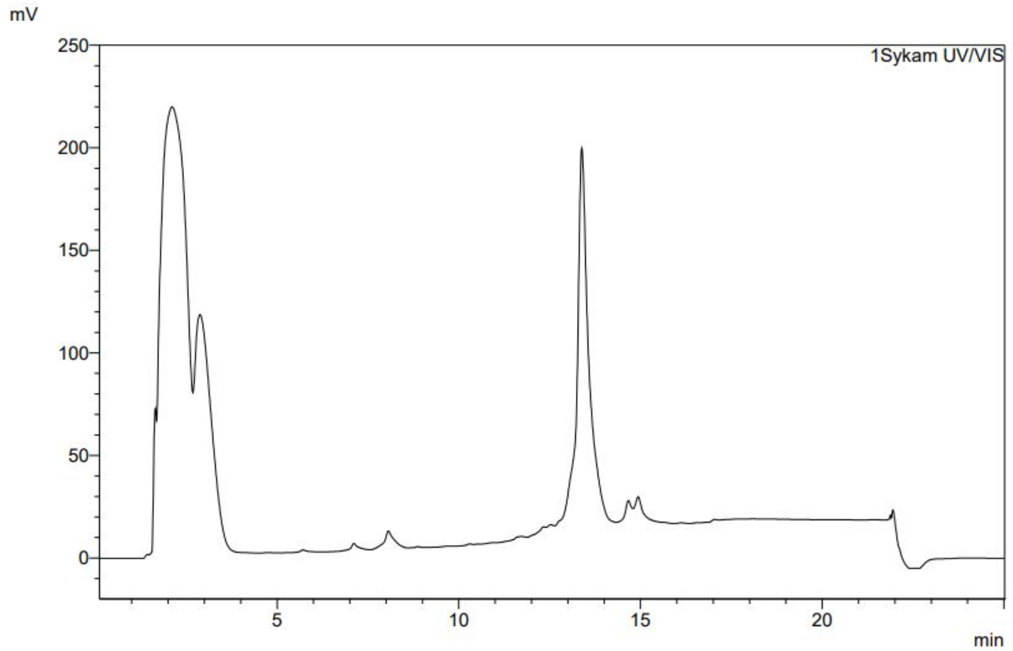

Figure S1891: RP-HPLC chromatogram of the reaction control of H-TATE(PG)-OH using the analytical control method 10-90% B (15 min, $\lambda = 220$ nm, MultoKrom® 100-5 C18-column (125 × 4.6 mm, 5 µm particle size, *CS Chromatographie GmbH*, $t_R = 13.4$ min).

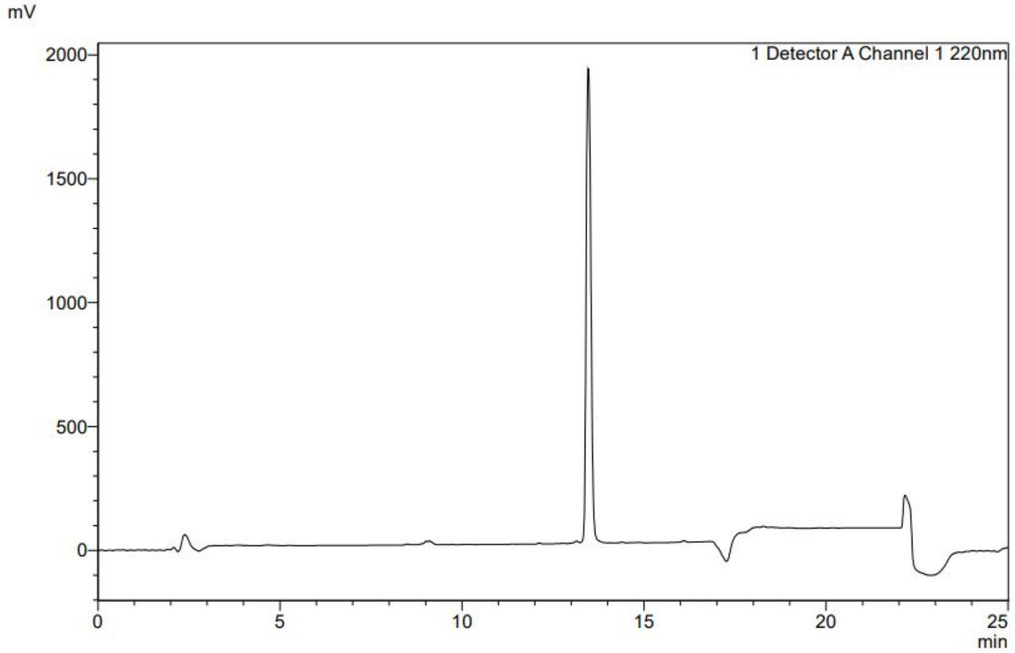

Figure S190: RP-HPLC chromatogram of SiFA*lin*-TATE using the analytical control method 10-60% B (15 min, $\lambda = 220$ nm, MultoKrom® 100-5 C18-column (125 × 4.6 mm, 5 µm particle size, CS Chromatographie GmbH), $t_R = 13.5$ min).

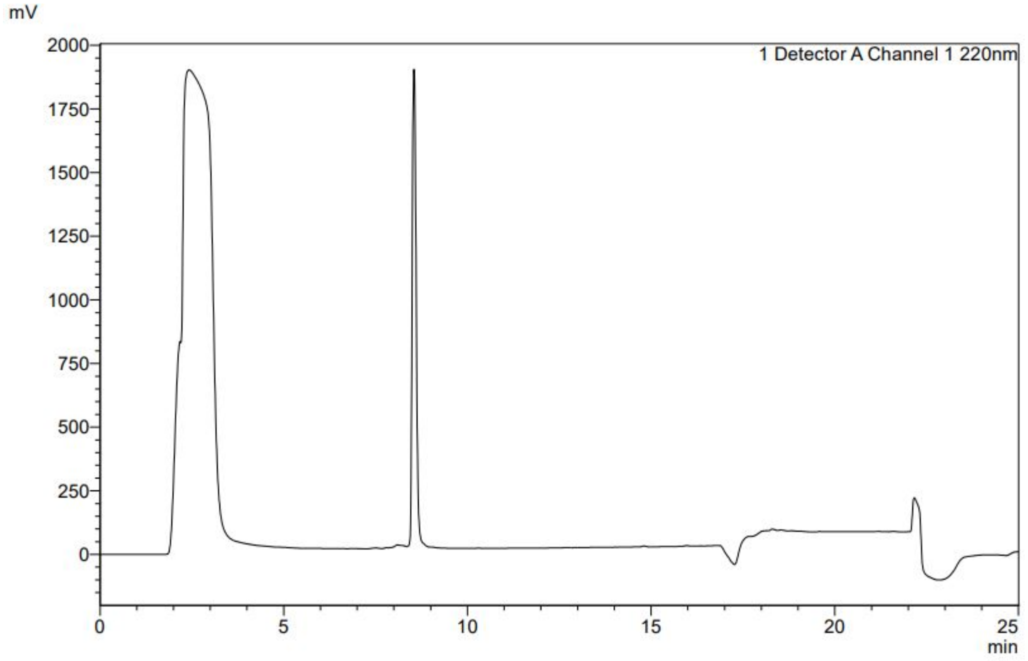

Figure S191: RP-HPLC chromatogram of [natGa]Ga-DOTA-TATE using the analytical control method 10-60% B (15 min, λ = 220 nm, MultoKrom® 100-5 C18-column (125 × 4.6 mm, 5 µm particle size, *CS Chromatographie GmbH*), t_R = 8.3 min).

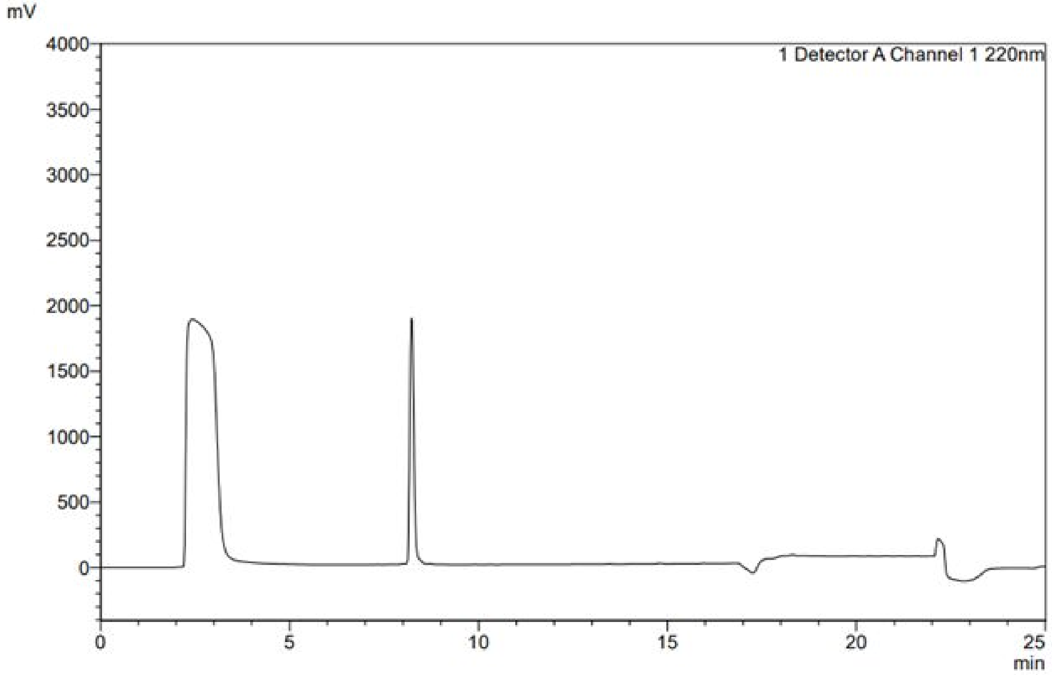

Figure S192: RP-HPLC chromatogram of [natLu]Lu-DOTA-TATE using the analytical control method 10-60% B (15 min, λ = 220 nm, MultoKrom® 100-5 C18-column (125 × 4.6 mm, 5 µm particle size, CS Chromatographie GmbH), t_R = 8.5 min).

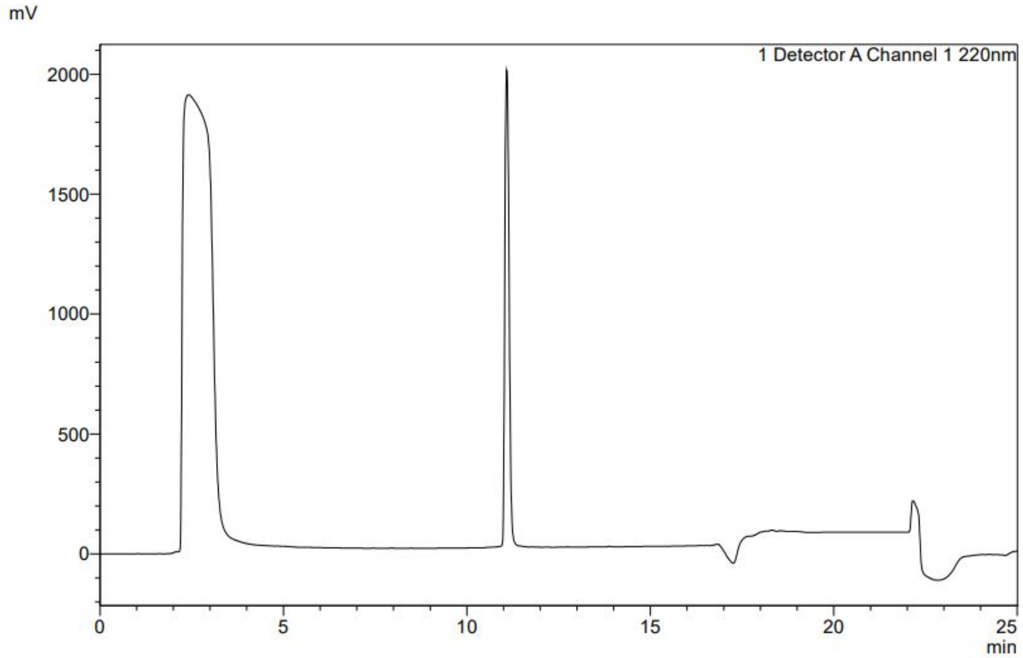

Figure S193: RP-HPLC chromatogram of [^{nat}Ga]Ga-(SiFA)SeFe-rhTATE1 using the analytical control method 10-60% B (15 min, λ = 220 nm, MultoKrom® 100-5 C18-column (125 × 4.6 mm, 5 µm particle size, *CS Chromatographie GmbH*), t_R = 11.1 min).

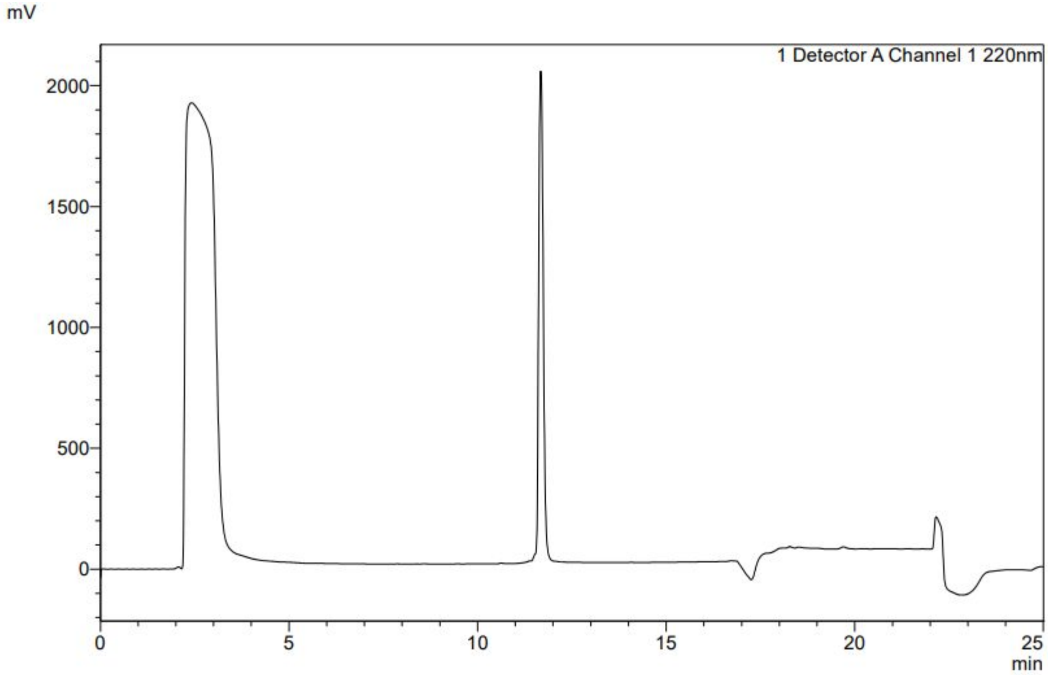

Figure S194: RP-HPLC chromatogram of [^{nat}Ga]Ga-(SiFA)SeFe-rhTATE2 using the analytical control method 10-90% B (15 min, λ = 220 nm, MultoKrom® 100-5 C18-column (125 × 4.6 mm, 5 µm particle size, *CS Chromatographie GmbH*), t_R = 11.7 min).

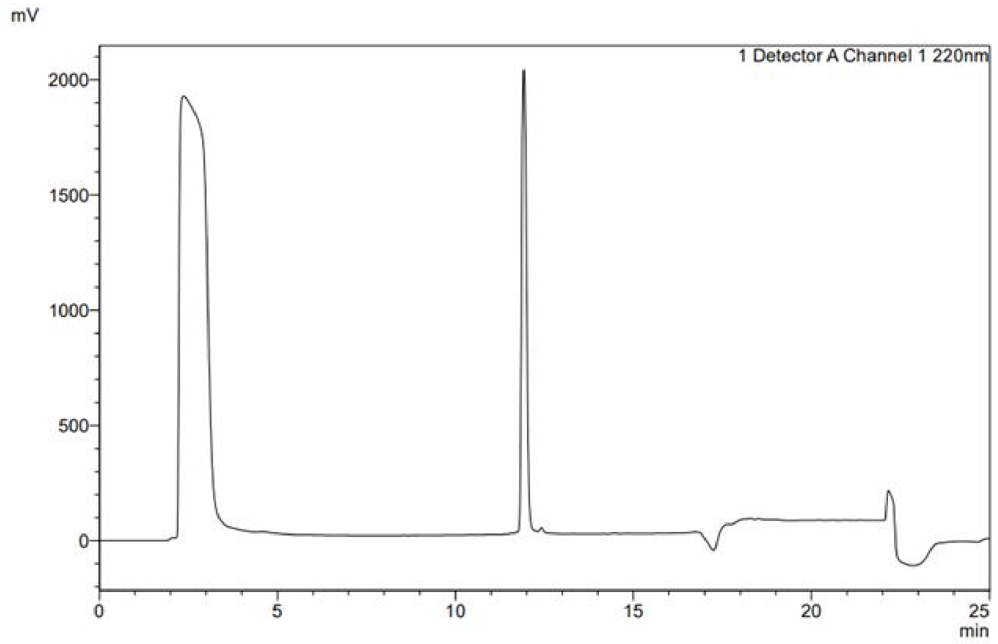

Figure S195: RP-HPLC chromatogram of [^{nat}Lu]Lu-(SiFA)SeFe-rhTATE3 using the analytical control method 10-90% B (15 min, λ = 220 nm, MultoKrom® 100-5 C18-column (125 × 4.6 mm, 5 µm particle size, CS Chromatographie GmbH), t_R = 11.9 min).

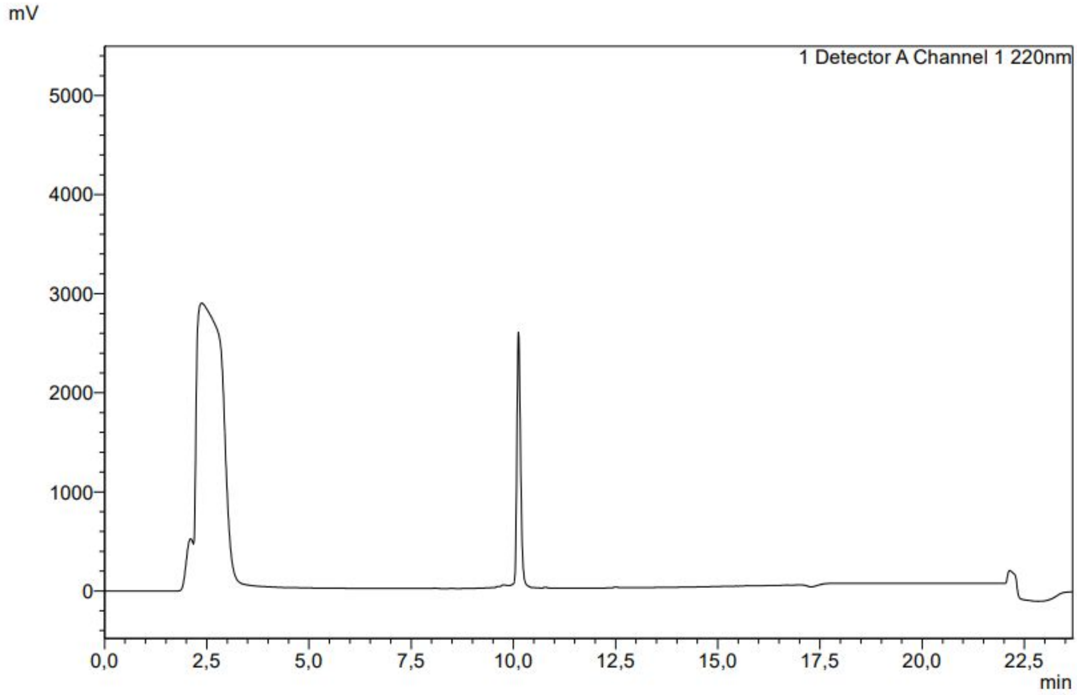

Figure S196: RP-HPLC chromatogram of [^{nat}Ga]Ga-(SiFA)BA-rhTATE1 using the analytical control method 10-90% B (15 min, λ = 220 nm, MultoKrom® 100-5 C18-column (125 × 4.6 mm, 5 µm particle size, *CS Chromatographie GmbH*), t_R = 10.1 min).

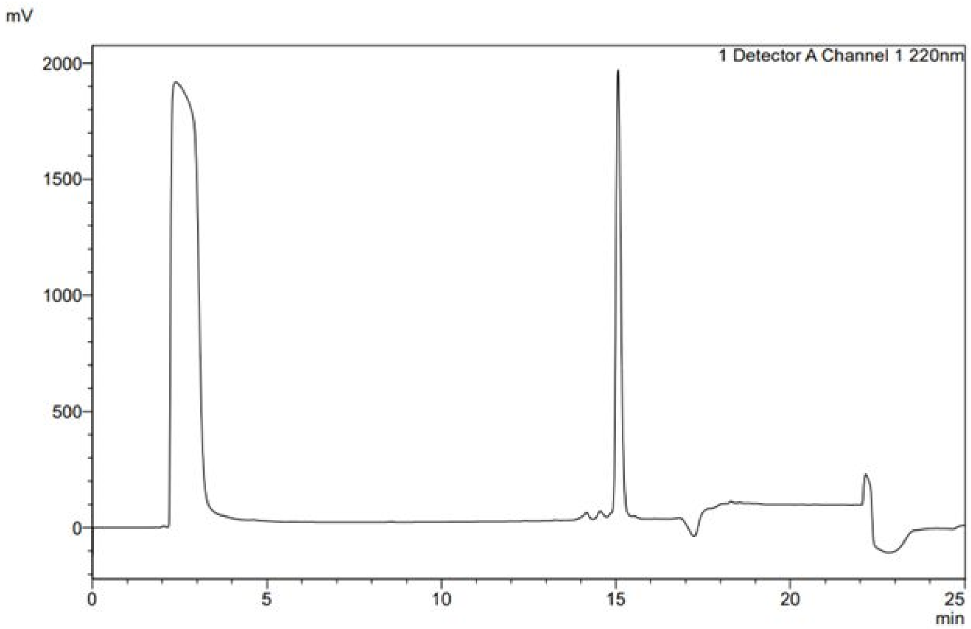

Figure S197: RP-HPLC chromatogram of [^{nat}Lu]Lu-(SiFA)BA-rhTATE3 using the analytical control method 10-90% B (15 min, λ = 220 nm, MultoKrom® 100-5 C18-column (125 × 4.6 mm, 5 µm particle size, CS Chromatographie GmbH), t_R = 15.1 min).

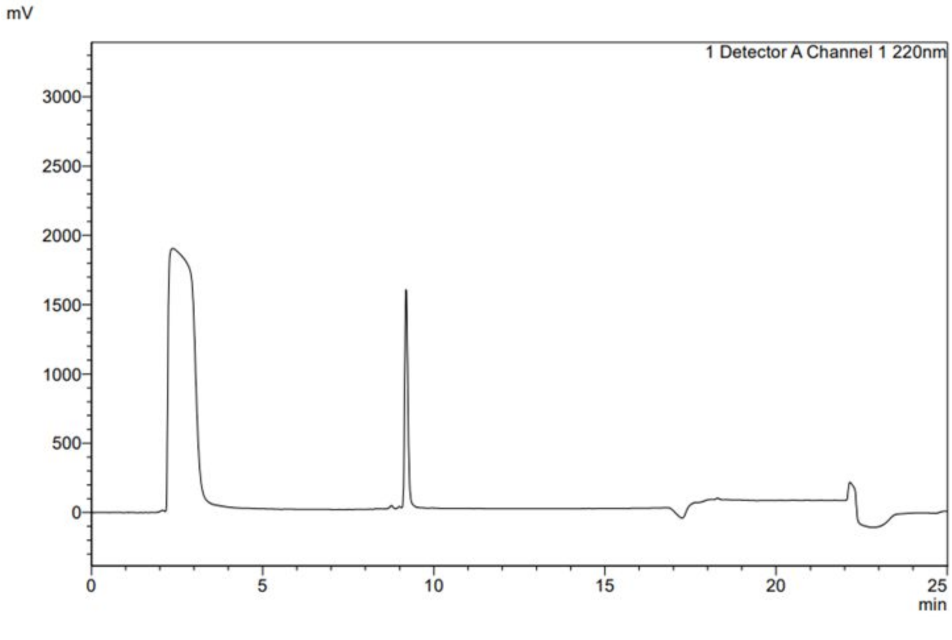

Figure S198: RP-HPLC chromatogram of [^{nat}I]TOC using the analytical control method 10-60% B (15 min, λ = 220 nm, MultoKrom® 100-5 C18-column (125 × 4.6 mm, 5 µm particle size, *CS Chromatographie GmbH*), t_R = 9.2 min).

3.3. Mass Spectrometry

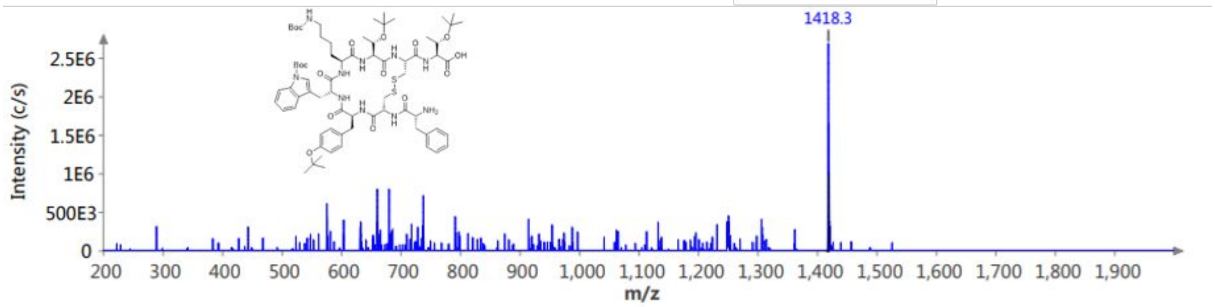

Figure S199: ESI^{+}-mass spectrum of H-TATE(PG)-OH.

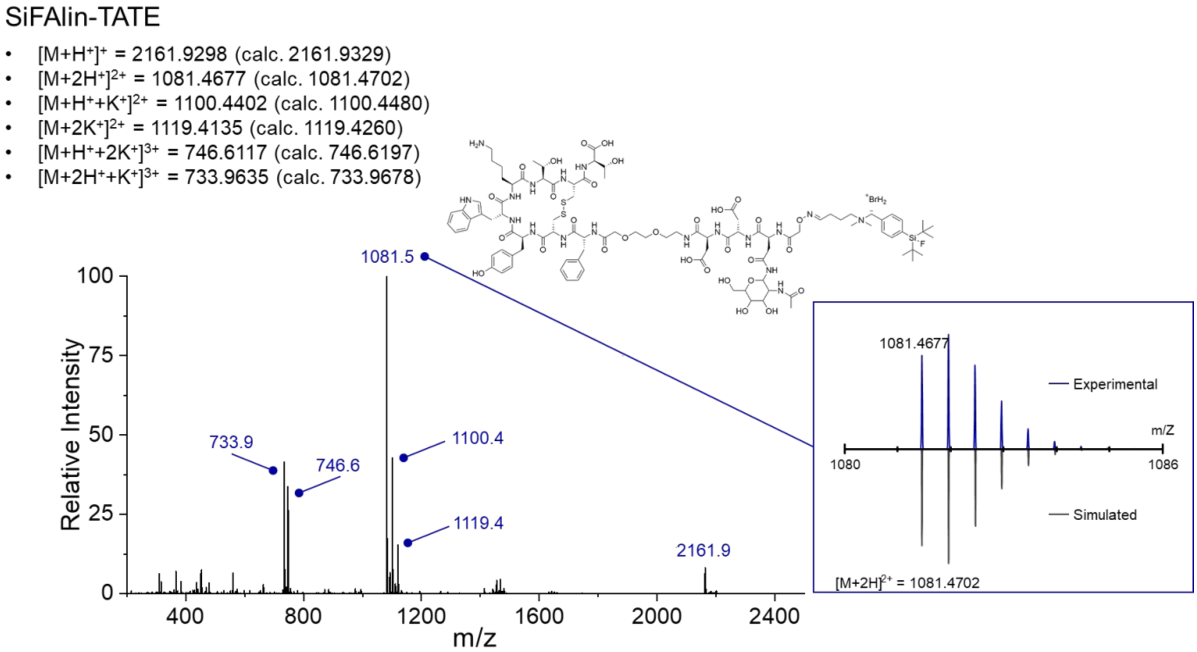

Figure S200: HR-ESI^{+}-mass spectrum of SiFA*lin*-TATE.

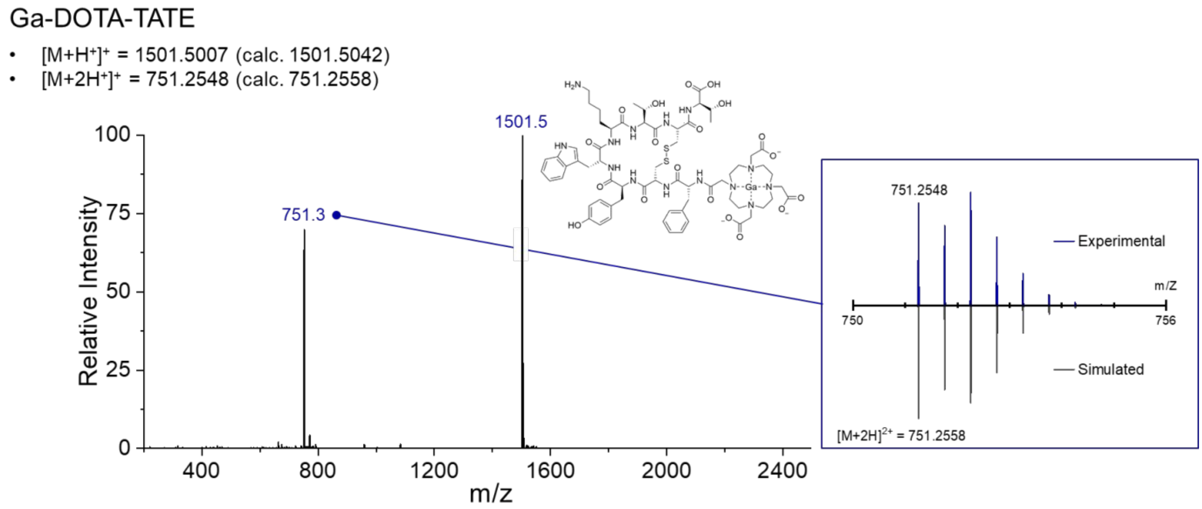

Figure S201: HR-ESI^{+}-mass spectrum of [^{nat}Ga]Ga-DOTA-TATE.

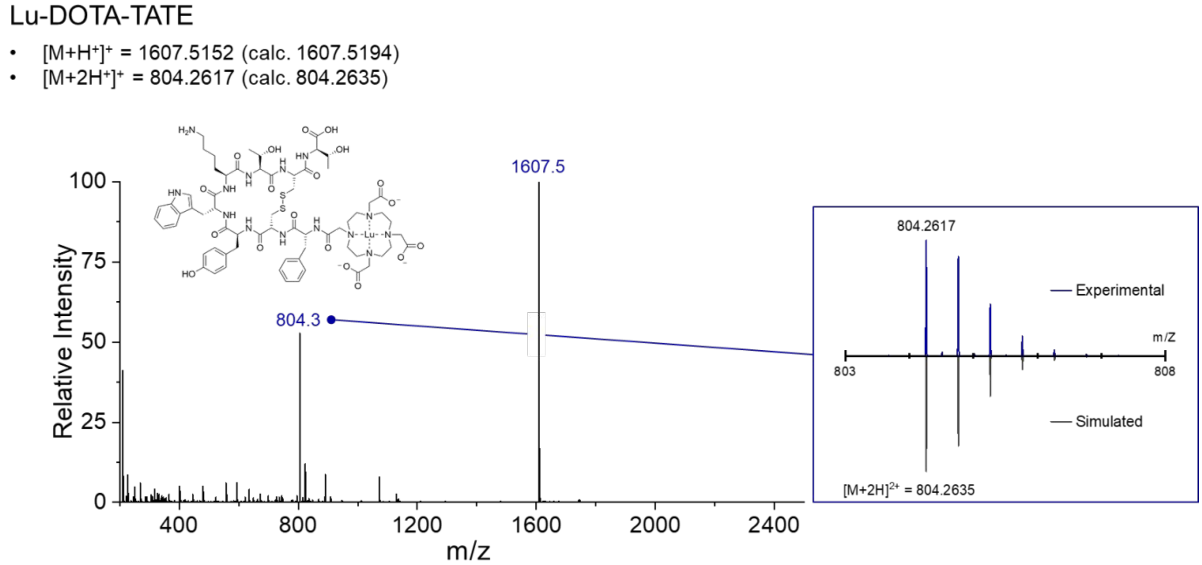

Figure S202: HR-ESI^{+}-mass spectrum of [^{nat}Lu]Lu-DOTA-TATE.

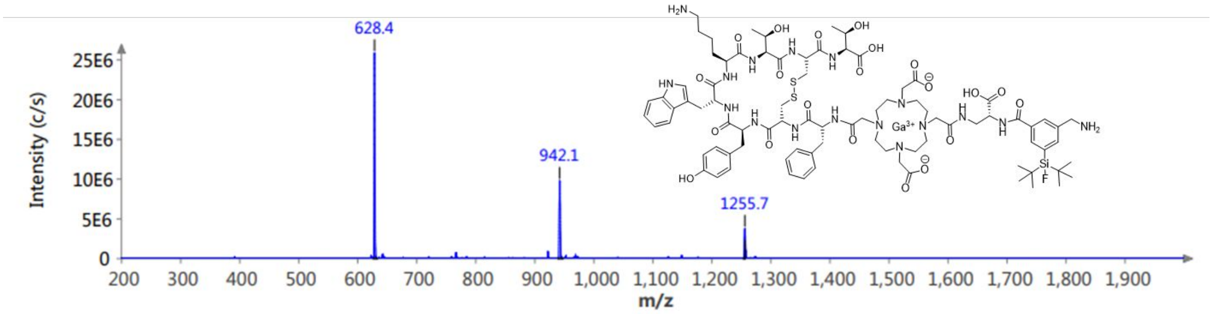

Figure S203: ESI^{+}-mass spectrum of [^{nat}Ga]Ga-(SiFA)SeFe-rhTATE1.

Ga-(SiFA)SeFe-rhTATE2

- $[M+H^+]^+$ = 1995.7416 (calc. 1995.7564)
- $[M+2H^+]^{2+}$ = 998.8754 (calc. 998.8782)
- $[2M+3H^+]^{3+}$ = 1331.4937 (calc. 1331.5121)

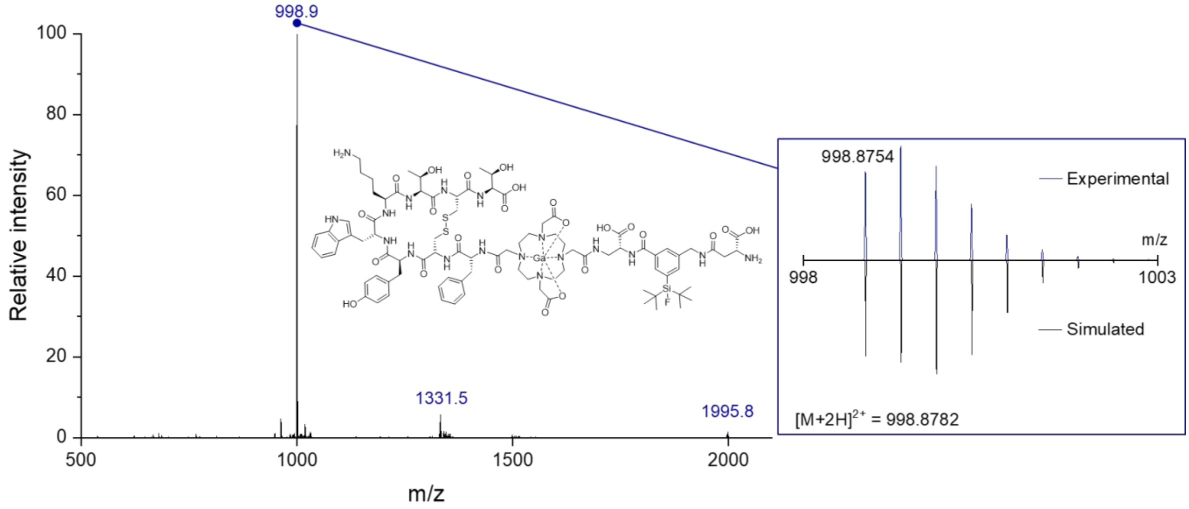

Figure S204:HR- ESI$^+$-mass spectrum of [^{nat}Ga]Ga-(SiFA)SeFe-rhTATE2.

(SiFA)SeFe-rhTATE3

- $[M+H^+]^+$ = 2104.9545 (calc. 2104.9595)
- $[M+2H^+]^{2+}$ = 1052.9805 (calc. 1052.9833)
- $[M+H^++K^+]^{2+}$ = 1071.9531 (calc. 1071.9611)
- $[M+3H^+]^{3+}$ = 702.3235 (calc. 702.3245)
- $[M+2H^++K^+]^{3+}$ = 714.9707 (calc. 714.9765)

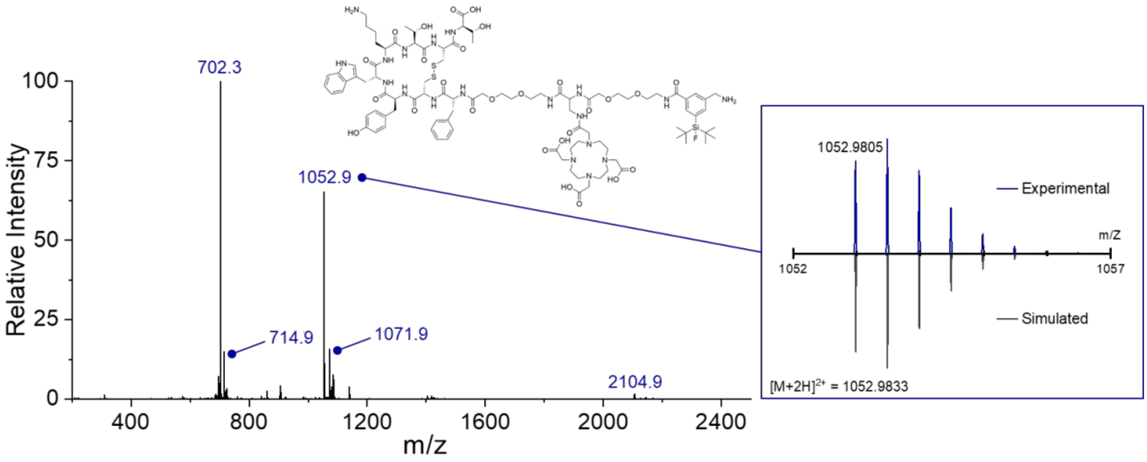

Figure S205: HR-ESI$^+$-mass spectrum of (SiFA)SeFe-rhTATE3.

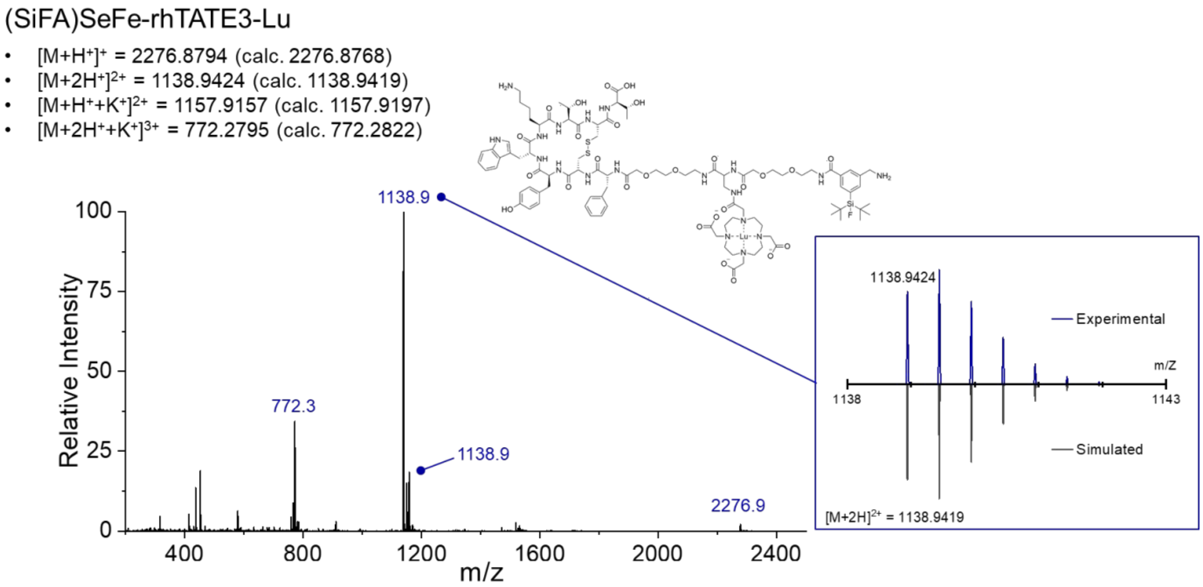

Figure S206: HR-ESI$^+$-mass spectrum of [^{nat}Lu]Lu-(SiFA)SeFe-rhTATE3.

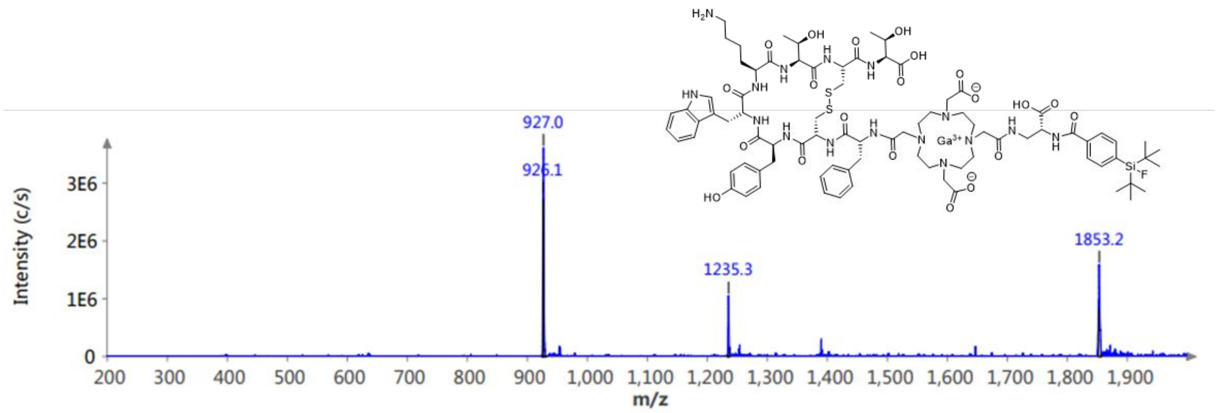

Figure S207: ESI$^+$-mass spectrum of [^{nat}Ga]Ga-(SiFA)BA-rhTATE1.

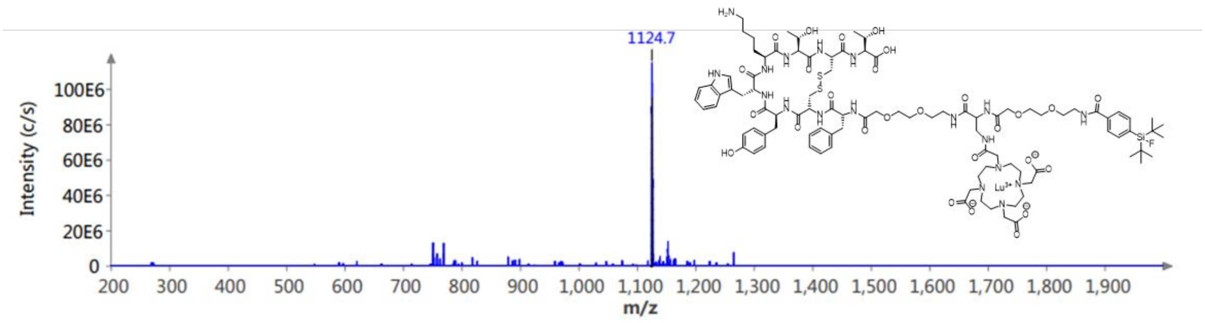

Figure S208: ESI$^+$-mass spectrum of [^{nat}Lu]Lu-(SiFA)BA-rhTATE3.

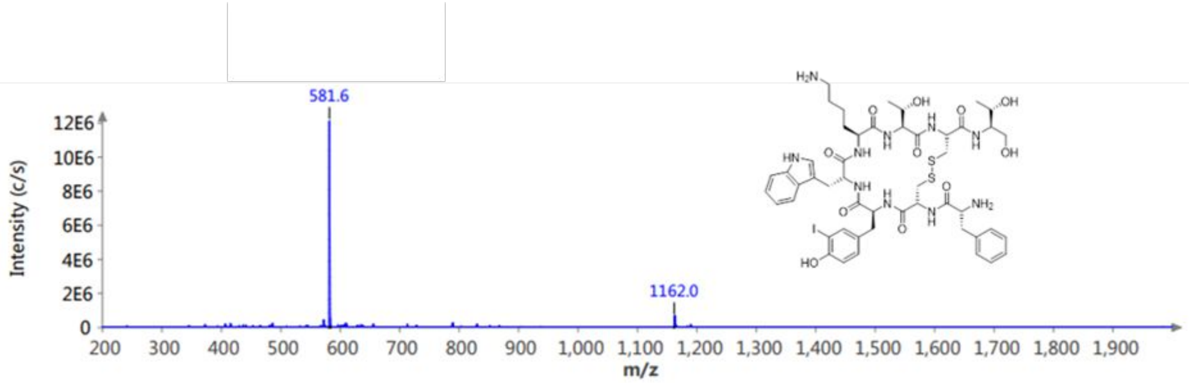

Figure S2092: ESI$^+$-mass spectrum of [^{nat}I]I-TOC.

3.4. Radiochemical characterization

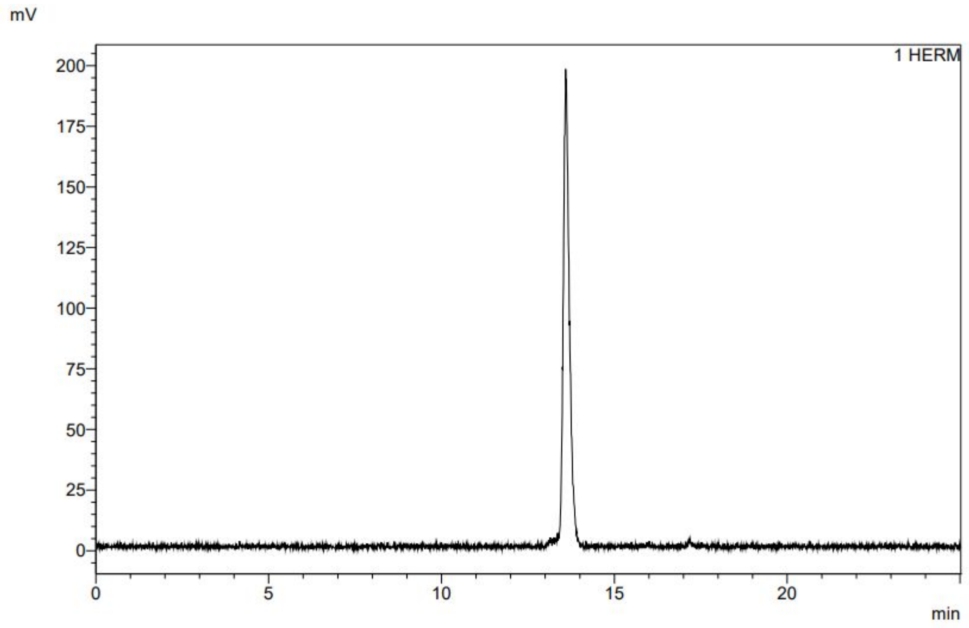

Figure S210: Radio-RP-HPLC chromatogram of [^{18}F]SiFA*lin*-TATE using the analytical control method 10-60% B (15 min, MultoKrom® 100-5 C18-column (125 × 4.6 mm, 5 µm particle size, CS Chromatographie GmbH), t_R = 13.6 min).

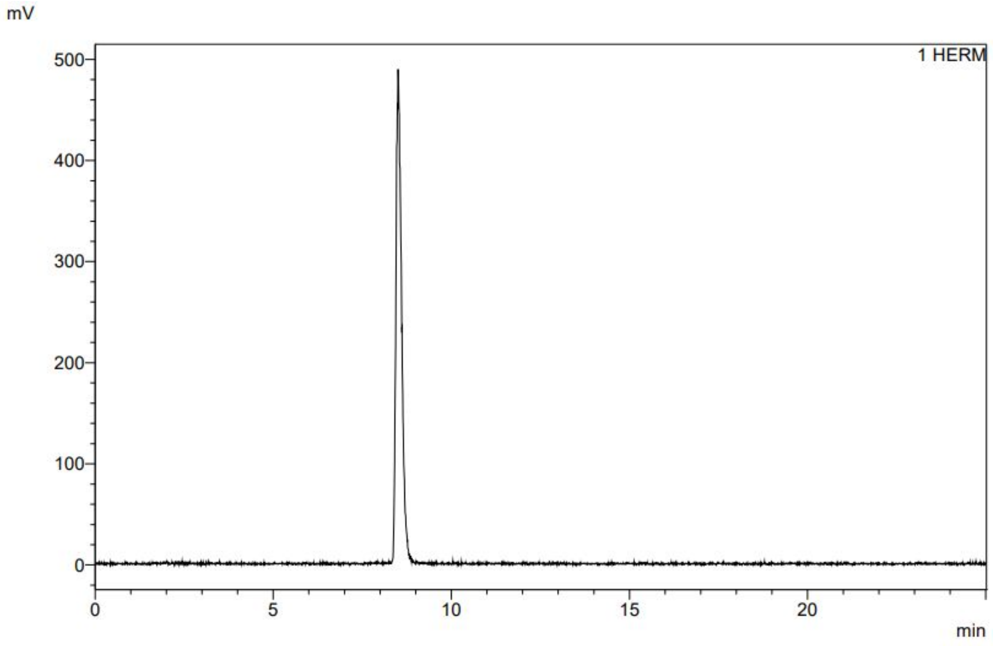

Figure S211: Radio-RP-HPLC chromatogram of [^{177}Lu]Lu-DOTA-TATE using the analytical control method 10-60% B (15 min, MultoKrom® 100-5 C18-column (125 × 4.6 mm, 5 µm particle size, CS Chromatographie GmbH), t_R = 8.5 min).

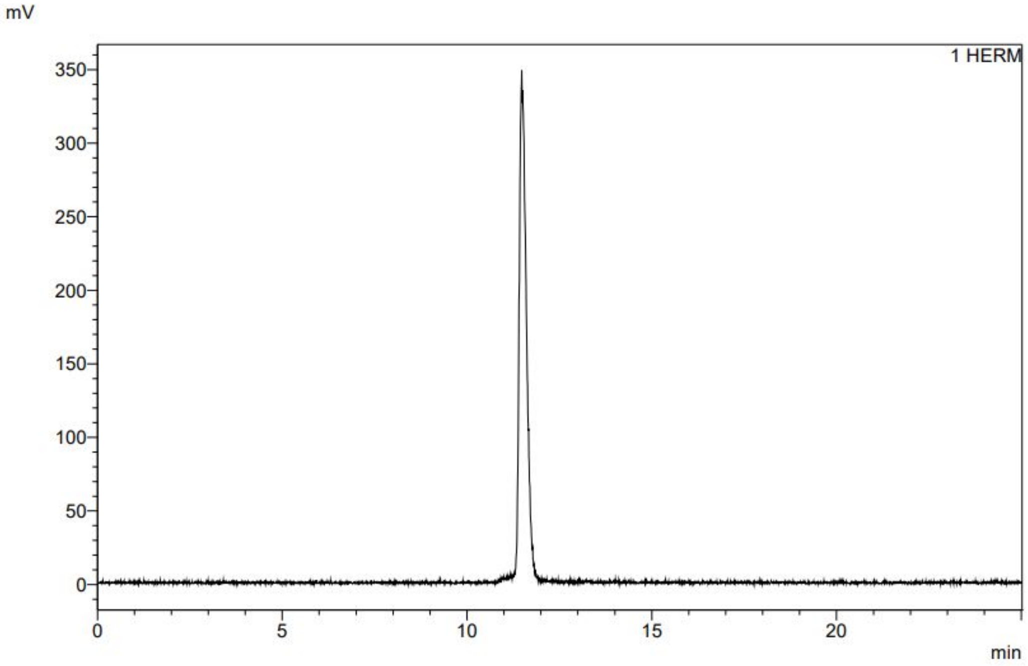

Figure S212: Radio-RP-HPLC chromatogram of [^{18}F](SiFA)SeFe-rhTATE1 using the analytical control method 10-60% B (15 min, MultoKrom® 100-5 C18-column (125 × 4.6 mm, 5 µm particle size, CS Chromatographie GmbH), t_R = 11.5 min).

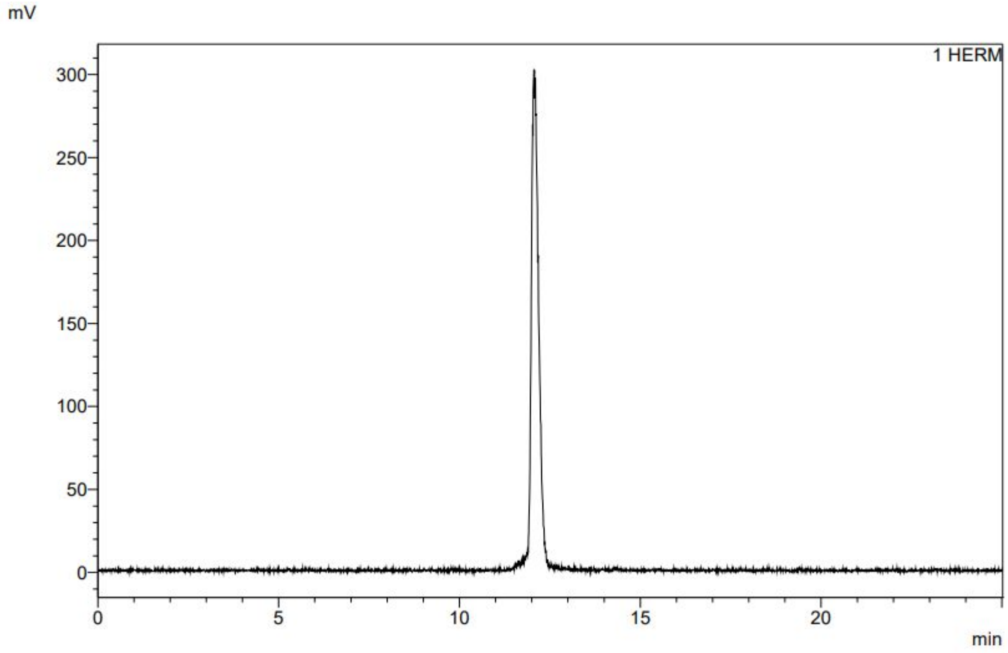

Figure S213: Radio-RP-HPLC chromatogram of [^{18}F](SiFA)SeFe-rhTATE2 using the analytical control method 10-60% B (15 min, MultoKrom® 100-5 C18-column (125 × 4.6 mm, 5 µm particle size, CS Chromatographie GmbH), t_R = 12.1 min).

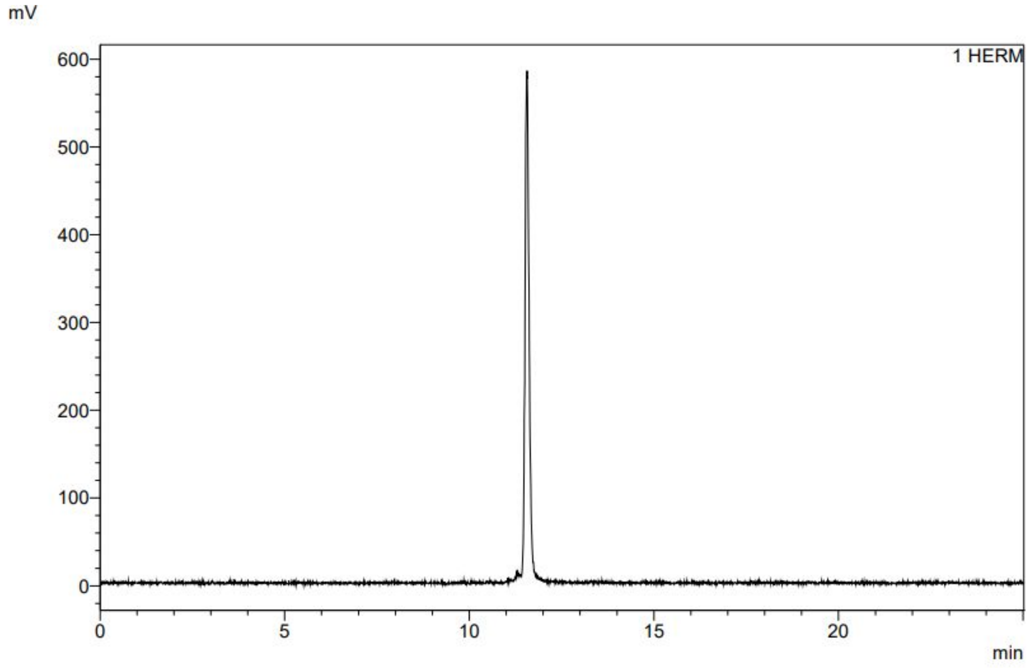

Figure S214: Radio-RP-HPLC chromatogram of [^{18}F](SiFA)SeFe-rhTATE3 using the analytical control method 10-60% B (15 min, MultoKrom® 100-5 C18-column (125 × 4.6 mm, 5 µm particle size, CS Chromatographie GmbH), t_R = 11.4 min).

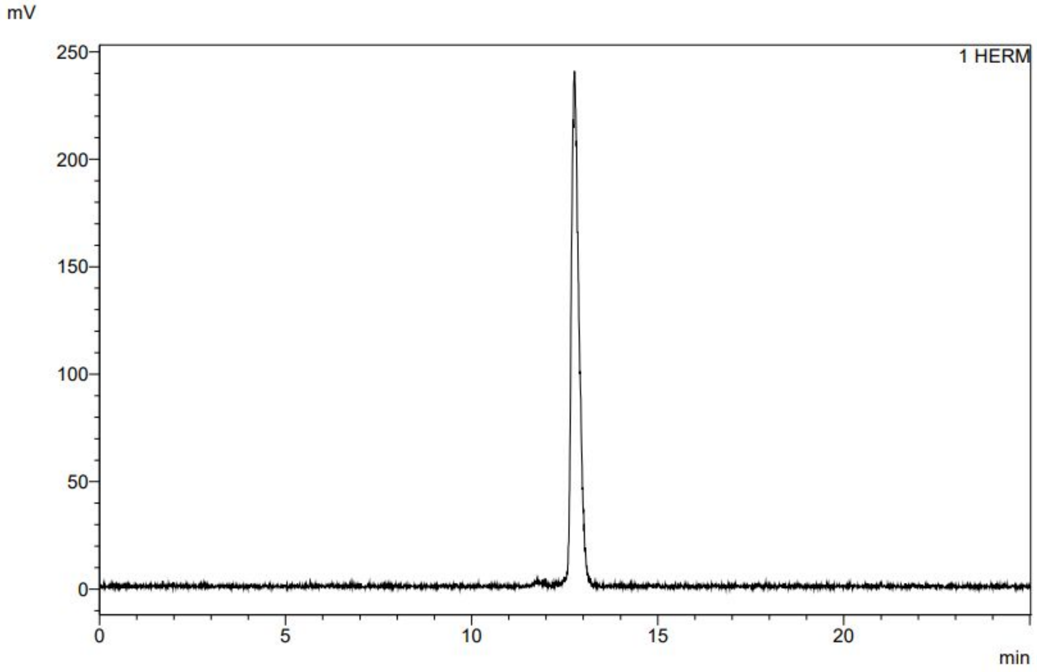

Figure S215: Radio-RP-HPLC chromatogram of [^{177}Lu]Lu-(SiFA)SeFe-rhTATE3 using the analytical control method 10-60% B (15 min, MultoKrom® 100-5 C18-column (125 × 4.6 mm, 5 µm particle size, CS Chromatographie GmbH), t_R = 11.9 min).

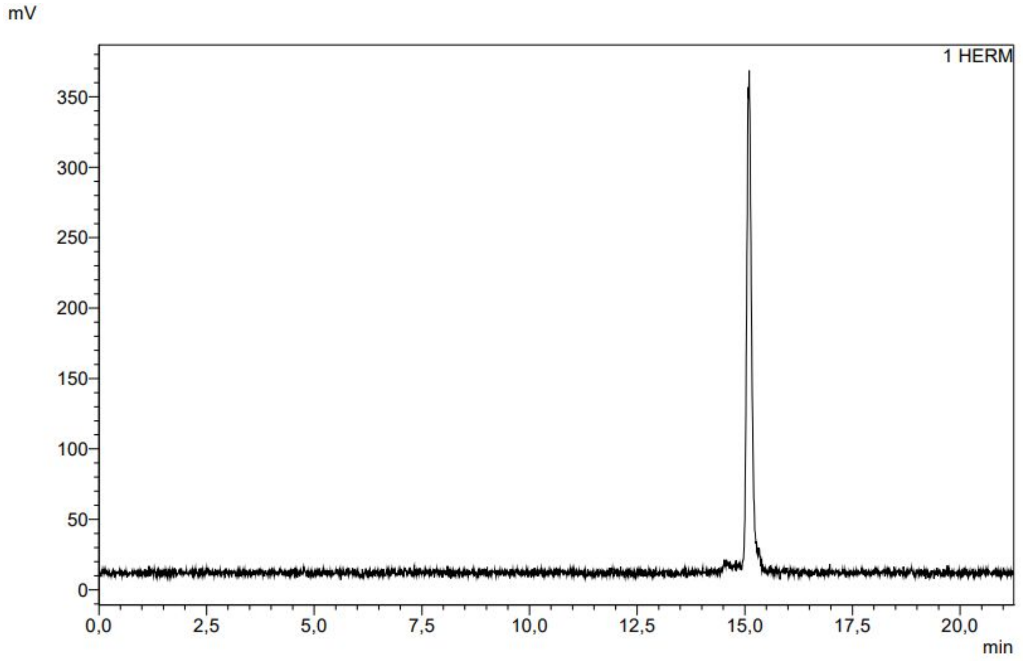

Figure S216: Radio-RP-HPLC chromatogram of [^{18}F](SiFA)BA-rhTATE1 using the analytical control method 10-60% B (15 min, MultoKrom® 100-5 C18-column (125 × 4.6 mm, 5 µm particle size, CS Chromatographie GmbH), t_R = 15.1 min)**.**

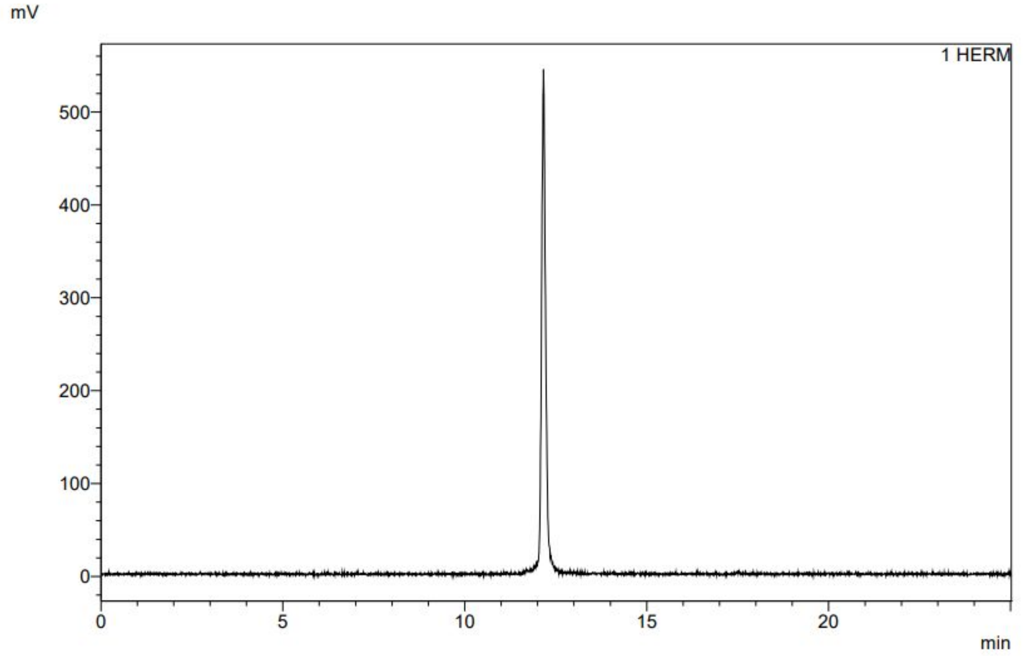

Figure S217: Radio-RP-HPLC chromatogram of [^{18}F](SiFA)BA-rhTATE3 using the analytical control method 10-60% B (15 min, MultoKrom® 100-5 C18-column (125 × 4.6 mm, 5 µm particle size, CS Chromatographie GmbH), t_R = 14.2 min).

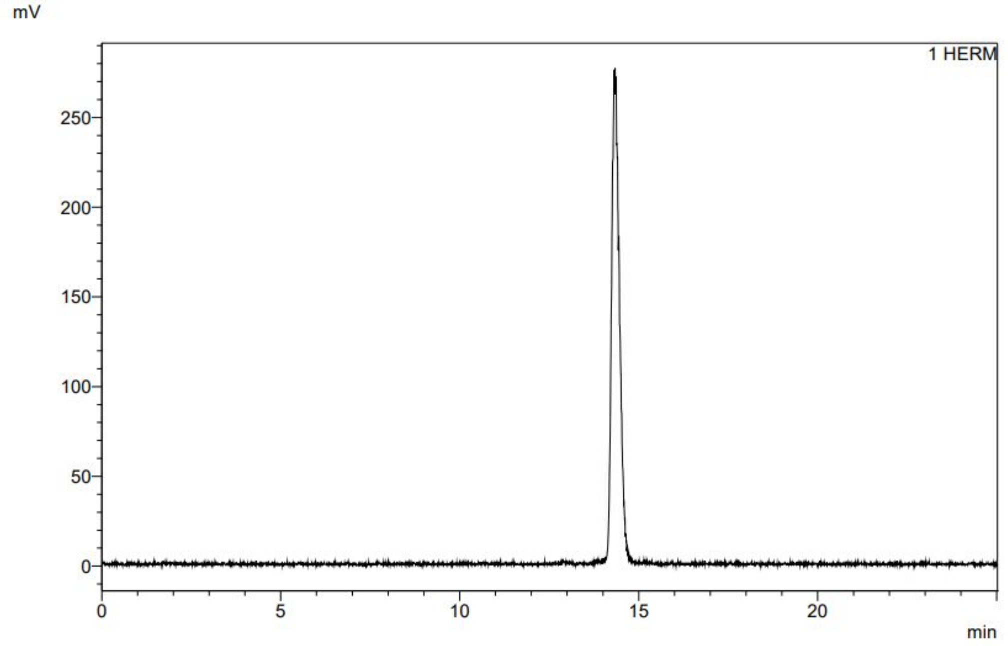

Figure S218: Radio-RP-HPLC chromatogram of [^{177}Lu]Lu-(SiFA)BA-rhTATE3 using the analytical control method 10-60% B (15 min, MultoKrom® 100-5 C18-column (125 × 4.6 mm, 5 µm particle size, CS Chromatographie GmbH), t_R = 14.3 min)**.**

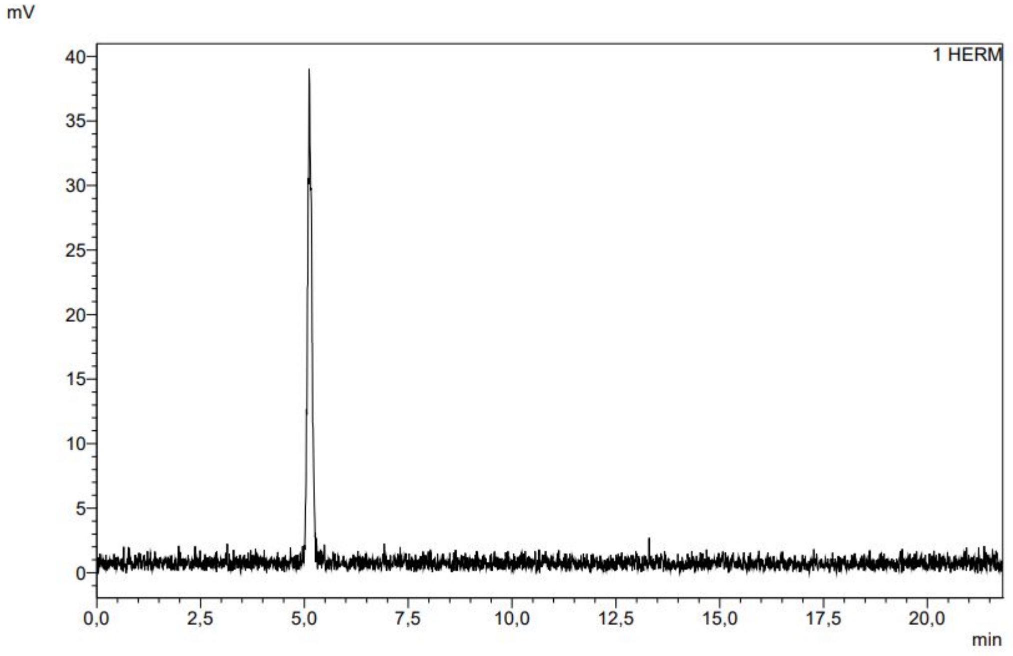

Figure S219: Radio-RP-HPLC chromatogram of [^{125}I]TOC using the analytical control method 20-50% B (15 min, MultoKrom® 100-5 C18-column (125 × 4.6 mm, 5 µm particle size, CS Chromatographie GmbH), t_R = 5.1 min).

3.5. Radio-TLC sstR2 peptides

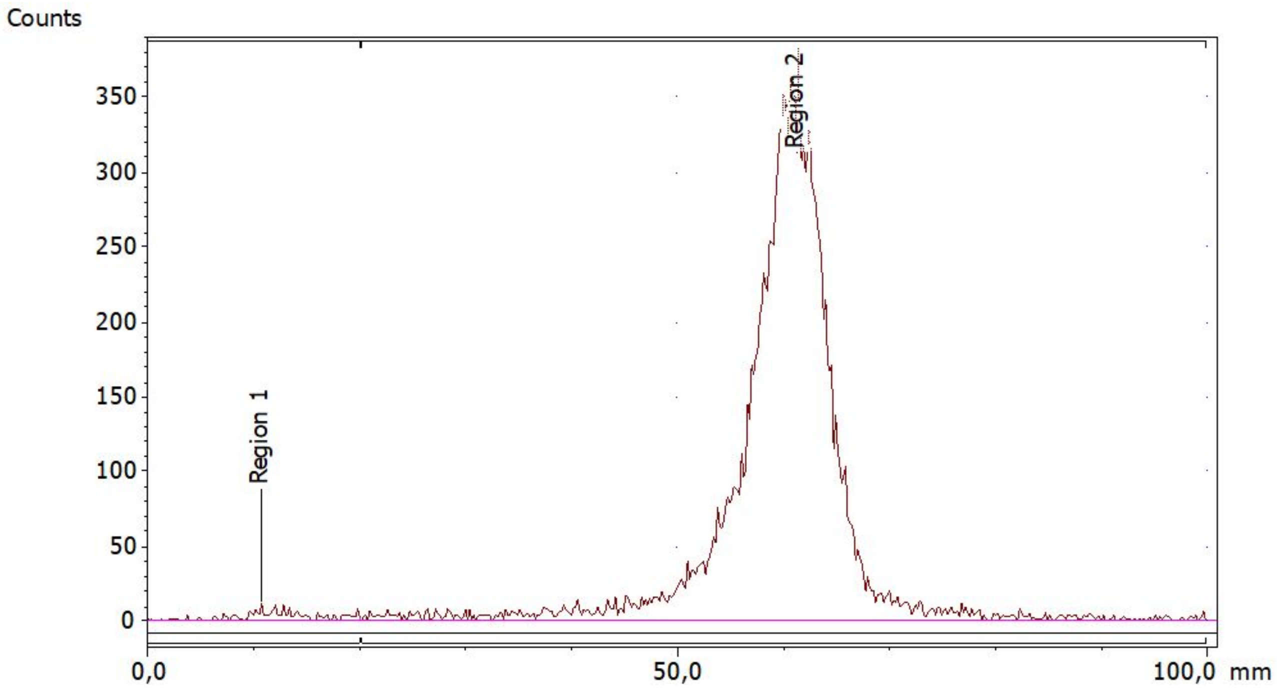

Figure S220: Radio-TLC chromatogram of [^{18}F]SiFA*lin*-TATE (flow agent: 60% MeCN/ 40% PBS (6/4 *v*/*v*) with 10% NaOAc in H_2O (2 M) and 1% TFA, stationary phase: TLC Silica gel 60 F254 from Merck Millipore).

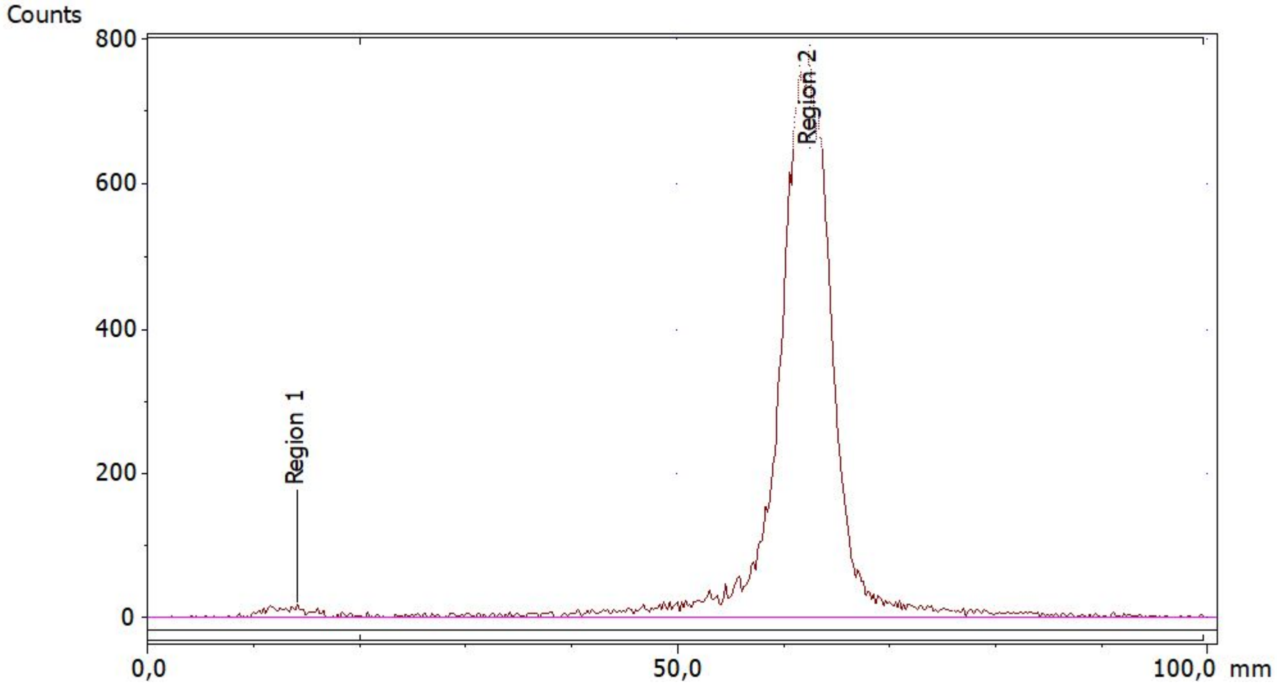

Figure S221*:* Radio-TLC chromatogram of [^{18}F](SiFA)SeFe-rhTATE1 (flow agent: 60% MeCN/ 40% PBS (6/4 *v*/*v*) with 10% NaOAc in H_2O (2 M) and 1% TFA, stationary phase: TLC Silica gel 60 F254 from *Merck Millipore*).

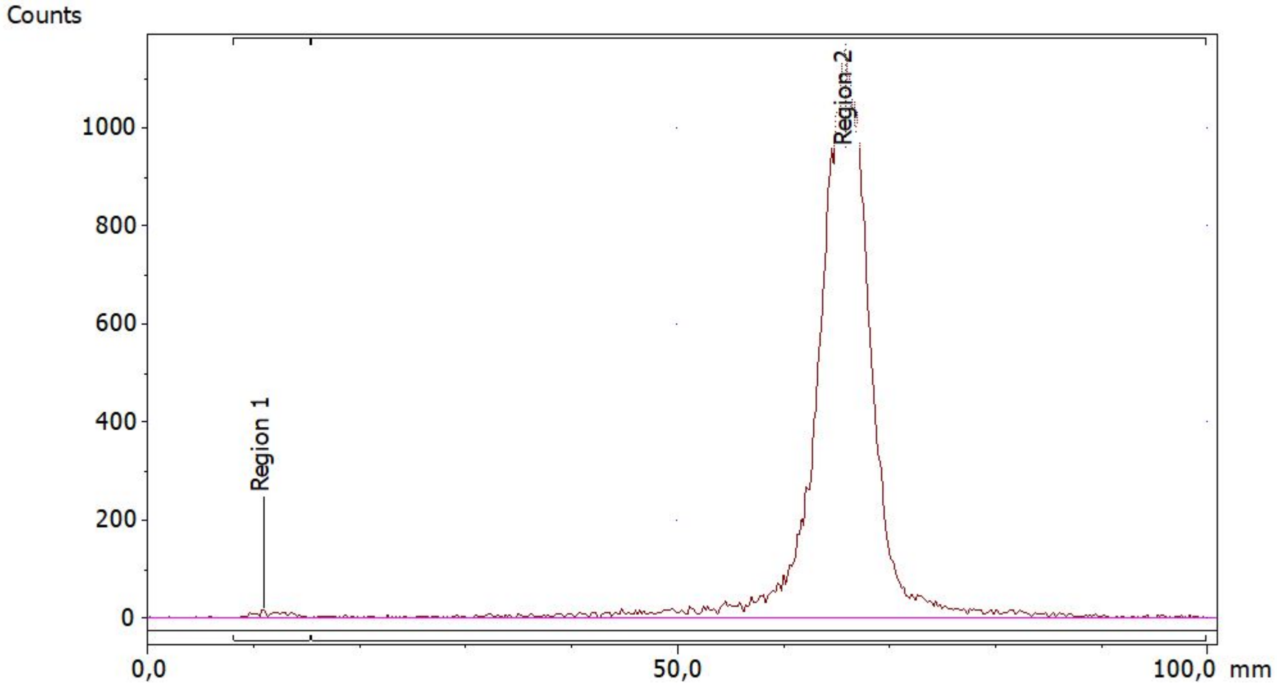

Figure S222*:* Radio-TLC chromatogram of [^{18}F](SiFA)SeFe-rhTATE2 (flow agent: 60% MeCN/ 40% PBS (6/4 *v*/*v*) with 10% NaOAc in H_2O (2 M) and 1% TFA, stationary phase: TLC Silica gel 60 F254 from *Merck Millipore*).

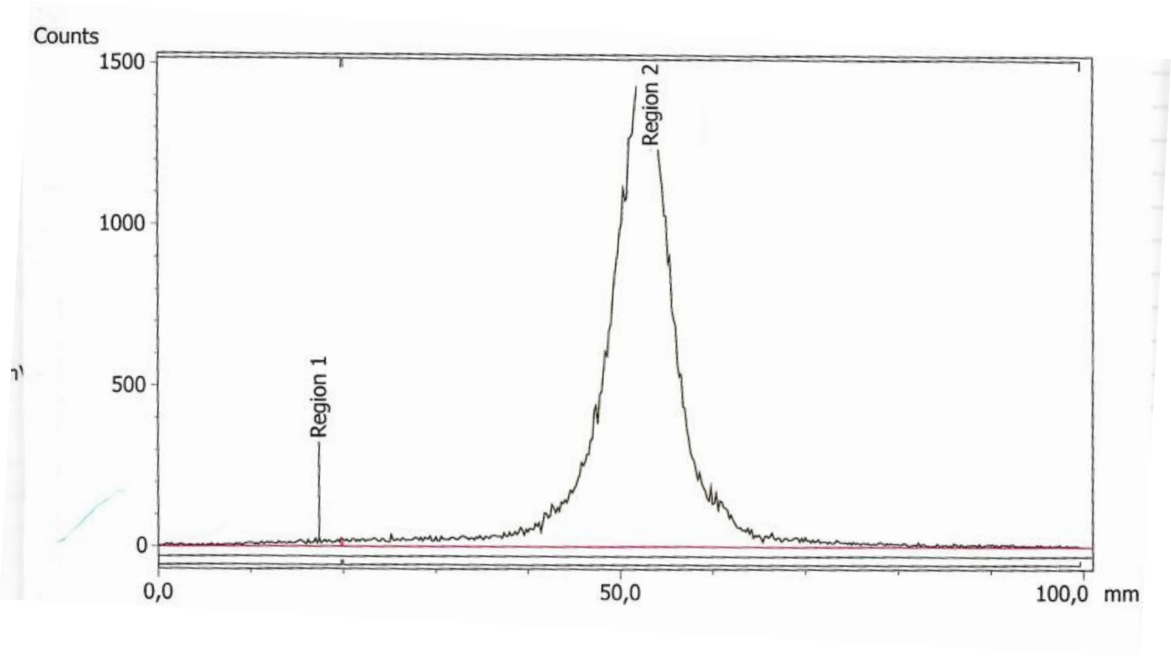

Figure S223: Radio-TLC chromatogram of [^{18}F](SiFA)SeFe-rhTATE3 (flow agent: 60% MeCN/ 40% PBS (6/4 *v*/*v*) with 10% NaOAc in H_2O (2 M) and 1% TFA, stationary phase: TLC Silica gel 60 F254 from *Merck Millipore*).

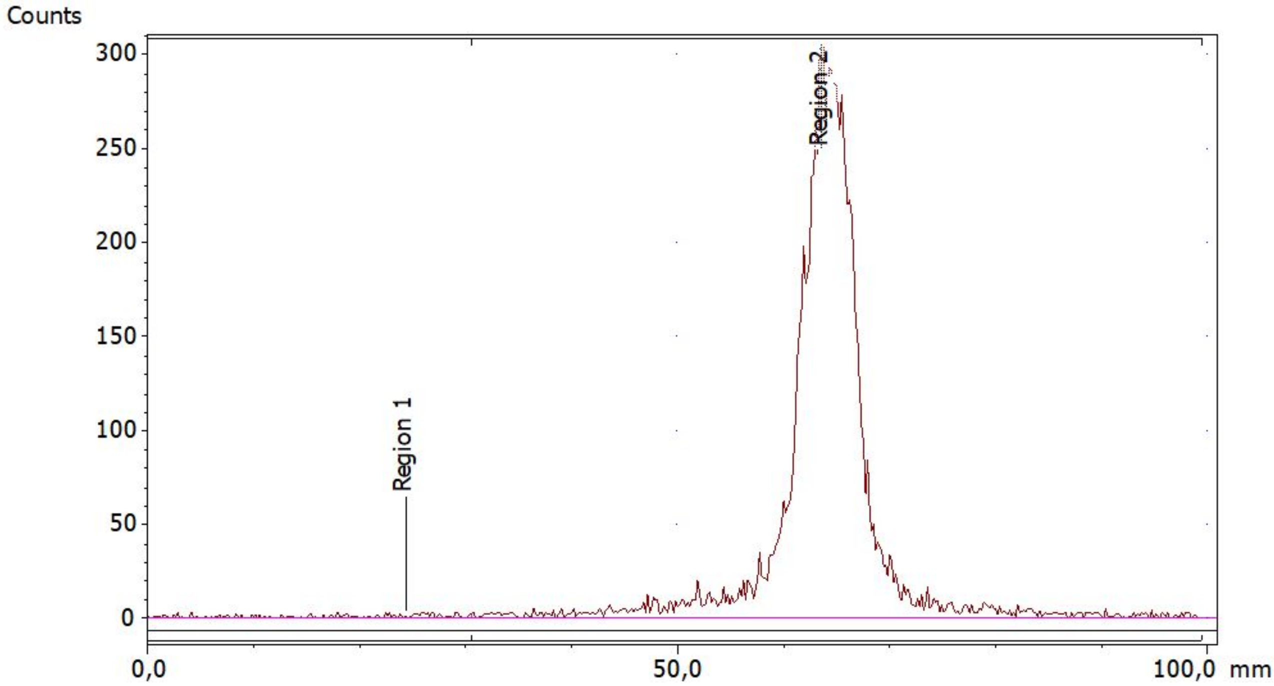

Figure S224*:* Radio-TLC chromatogram of [^{18}F](SiFA)BA-rhTATE1 (flow agent: 60% MeCN/ 40% PBS (6/4 *v*/*v*) with 10% NaOAc in H_2O (2 M) and 1% TFA, stationary phase: TLC Silica gel 60 F254 from *Merck Millipore*).

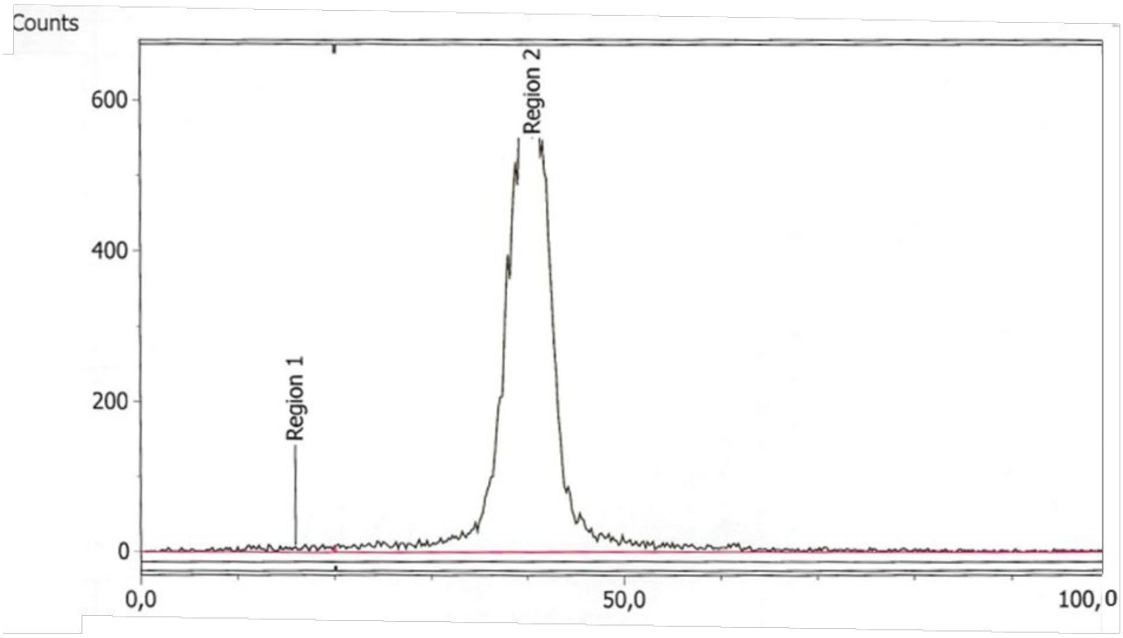

Figure S225: Radio-TLC chromatogram of **[^{18}F](SiFA)BA-rhTATE3** (flow agent: 60% MeCN/ 40% PBS (6/4 *v*/*v*) with 10% NaOAc in H_2O (2 M) and 1% TFA, stationary phase: TLC Silica gel 60 F254 from *Merck Millipore*)**.**

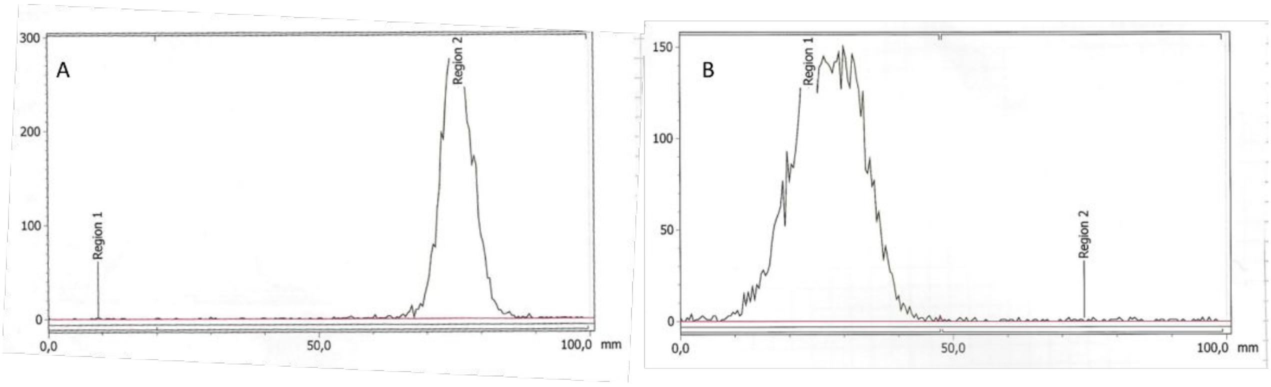

Figure S226: Radio-TLC chromatogram of [^{177}Lu]Lu-DOTA-TATE using A: 1 M NH_4OAc/DMF (1/1, *v*/*v*), stationary phase: TLC Silica gel 60 F254 and B: 0.1 M sodium citrate × 1.5 H_2O, stationary phase: iTLC-SC.**.**

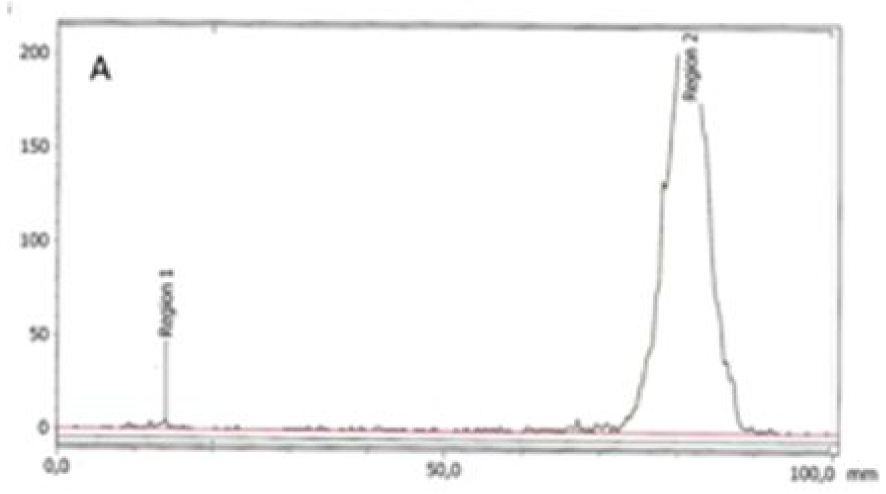

Figure S227: Radio-TLC chromatogram of [^{177}Lu]Lu-(SiFA)SeFe-rhTATE3 (flow agent: 60% MeCN/ 40% PBS (6/4 *v*/*v*) with 10% NaOAc in H_2O (2 M) and 1% TFA, stationary phase: TLC Silica gel 60 F254 from *Merck Millipore*)**.**

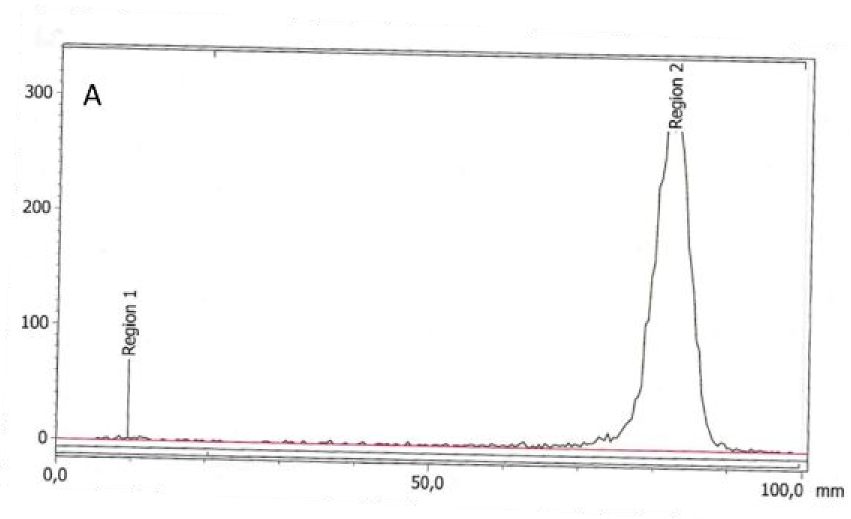

Figure S228: Radio-TLC chromatogram of [^{177}Lu]Lu-(SiFA)BA-rhTATE3 (flow agent: 60% MeCN/ 40% PBS (6/4 *v*/*v*) with 10% NaOAc in H_2O (2 M) and 1% TFA, stationary phase: TLC Silica gel 60 F254 from *Merck Millipore*)**.**

3.6. Optimization of the ^{177}Lu-labeling

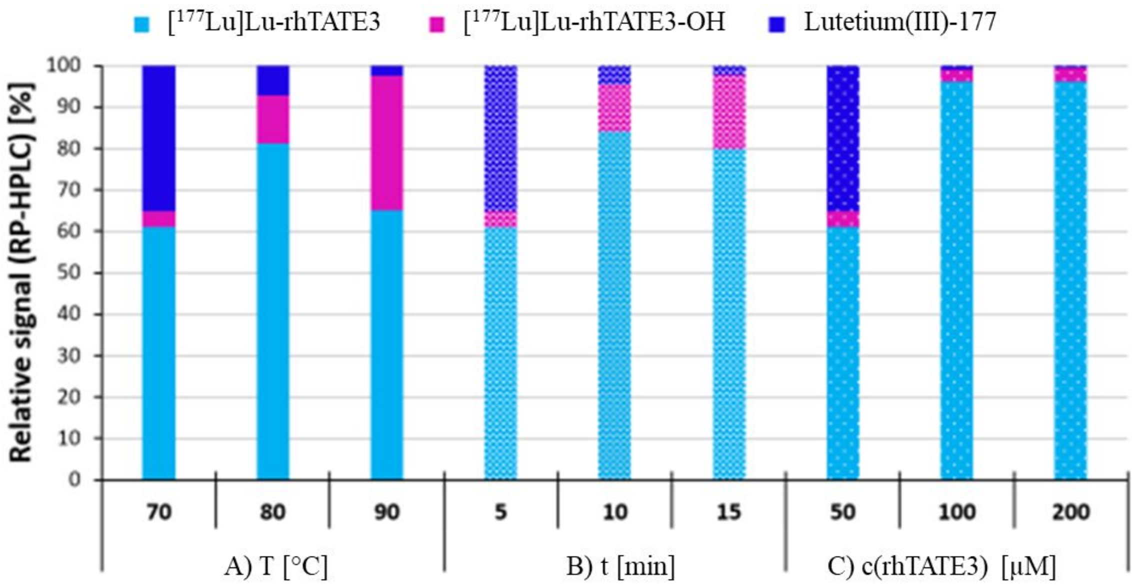

Figure S229: Relative signals [%] of the radiolabeled product [^{177}Lu]Lu-(SiFA)SeFe-rhTATE3 (light blue), the hydrolyzed byproduct [^{177}Lu]Lu-(SiFA)SeFe-rhTATE3-OH (pink), and uncomplexed Lutetium(III)-177 (blue), observed in the radio-RP-HPLC chromatograms after ^{177}Lu-labeling of rhTATE3. Labeling was carried out in NaOAc buffered hydrochloric acid (pH = 5.5) at different temperatures (A; Ligand = 50 µmol, t = 5 min), reaction times (B; Ligand = 50 µmol, T = 70°C), and ligand concentrations (C; t = 5 min, T = 70°C).

4. Western Blots for sstR2 expression

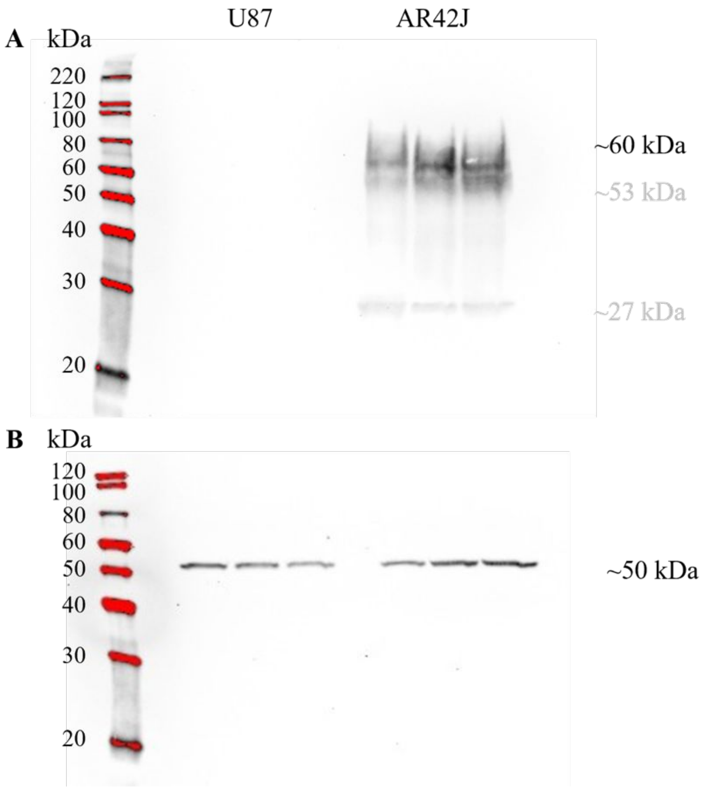

Figure S230: Full-size Western blots for Figure 5. A) Determination of the sst2R expression in U87 cell lysates (left) and AR42J lysates (right). B) alpha-tubulin control for U87 lysates (left) and AR42J lysates (right). Main bands are marked in black. Magic MarkTM XP Western Protein Standard was used as the ladder.

5. Supplementary Tables

Table S1: Results of ^{18}F-labeling of model bioconjugate peptides reporting radiochemical conversation (RCC) (n = 3), radiochemical yield decay corrected ($RCY_{d.c.}$) (n = 3), radiochemical purity via radio-RP-HPLC (RCP_{HPLC}).

Compound	RCC [%]	$RCY_{d.c.}$ [%]	RCP_{HPLC} [%]
[^{18}F]H_2N-(SiFA)SeFe-Gly-Lys-OH ($1X_1$)	52 ± 8.5	27 ± 2.4	100
[^{18}F]H_2N-Lys-(SiFA)SeFe-Gly-Lys-OH ($2X_1$)	37 ± 3.1	19 ± 2.2	100
[^{18}F]H_2N-Glu-(SiFA)SeFe-Gly-Lys-OH ($3X_1$)	53 ± 0.3	35 ± 2.2	99
[^{18}F]H_2N-(SiFA)SeFe-Gly-Asp-OH ($1X_2$)	90 ± 1.4	61 ± 5.3	99
[^{18}F]H_2N-Lys-(SiFA)SeFe-Gly-Asp-OH ($2X_2$)	40 ± 5.1	27 ± 6.1	100
[^{18}F]H_2N-Glu-(SiFA)SeFe-Gly-Asp-OH ($3X_2$)	45 ± 0.6	31 ± 5.0	100
[^{18}F]H_2N-(SiFA)SeFe-Gly-Tyr-OH ($1X_3$)	67 ± 4.0	47 ± 8.6	100
[^{18}F]H_2N-Lys-(SiFA)SeFe-Gly-Tyr-OH ($2X_3$)	52 ±1.9	22 ± 2.2	100
[^{18}F]H_2N-Glu-(SiFA)SeFe-Gly-Tyr-OH ($3X_3$)	49 ± 1.9	34 ± 3.3	100

Table S2: Half-life of (SiFA)SeFe model bioconjugate peptides towards pH 5.5, 90 °C (Lutetium labeling conditions); pH 6.5, 1mM aq. NaF, RT (reverse isotopic exchange) and pH 7.4, 37 °C (physiological conditions).

Compound	pH 5.5, 90 °C [h]	pH 6.5, 1 mm aq. NaF, RT [min]	pH 7.4, 37 °C [min]
[^{18}F]H_2N-(SiFA)SeFe-Gly-Lys-OH ($1X_1$)	38 ± 0.9	10 ± 1.1	27 ± 2.1
[^{18}F]H_2N-Lys-(SiFA)SeFe-Gly-Lys-OH ($2X_1$)	25 ± 5.5	14 ± 0.8	18 ± 2.1
[^{18}F]H_2N-Glu-(SiFA)SeFe-Gly-Lys-OH ($3X_1$)	40 ± 0.6	34 ± 2.1	57 ± 1.3
[^{18}F]H_2N-(SiFA)SeFe-Gly-Asp-OH ($1X_2$)	37 ± 11	25 ± 6.1	299 ± 12
[^{18}F]H_2N-Lys-(SiFA)SeFe-Gly-Asp-OH ($2X_2$)	49 ± 7.1	29 ± 0.8	43 ± 1.3
[^{18}F]H_2N-Glu-(SiFA)SeFe-Gly-Asp-OH ($3X_2$)	59 ± 5.2	90 ± 3.6	597 ± 43
[^{18}F]H_2N-(SiFA)SeFe-Gly-Tyr-OH ($1X_3$)	43 ± 2.8	27 ± 2.2	14 ± 3.0
[^{18}F]H_2N-Lys-(SiFA)SeFe-Gly-Tyr-OH ($2X_3$)	48 ± 13	20 ± 2.9	21 ± 6.0
[^{18}F]H_2N-Glu-(SiFA)SeFe-Gly-Tyr-OH ($3X_3$)	42 ± 7.8	57 ± 1.6	29 ± 0.8

Table S3: Results of ^{18}F-/^{177}Lu-labeling of sstR2-rh-compounds reporting radiochemical conversation (RCC), radiochemical yield decay corrected ($RCY_{d.c.}$), radiochemical purity *via* radio-RP-HPLC (RCP_{HPLC}) and radiochemical purity *via* radio-TLC (RCP_{TLC}).

Compound	RCC [%]	$RCY_{d.c.}$ [%]	RCP_{HPLC} [%]	RCP_{TLC} [%]
[^{18}F]SiFA*lin*-TATE	59	59	98	98
[^{18}F][^{nat}Ga]Ga-(SiFA)BA-rhTATE1	39	47	94	99
[^{18}F][^{nat}Lu]Lu-(SiFA)BA-rhTATE3	54	36	≥99	98
[^{18}F][^{nat}Ga]Ga-(SiFA)SeFe-rhTATE1	54	47	98	98
[^{18}F][^{nat}Ga]Ga-(SiFA)SeFe-rhTATE2	60	39	97	99
[^{18}F][^{nat}Lu]Lu-(SiFA)SeFe-rhTATE3	63	47	98	99
[^{177}Lu]Lu-DOTA-TATE	-	≥99	≥99	≥99
[^{177}Lu]Lu-(SiFA)BA-rhTATE3	-	99	99	99
[^{177}Lu]Lu-(SiFA)SeFe-rhTATE3	-	97	97	99

Table S4: Summary of *in vitro* evaluations collected for sstR2-rh-compounds, including binding affinity (IC_{50}, n=3), lipophilicity ($\log D_{pH=7.4}$, n=6), human serum albumin binding (HSA, n=1) and stability studies in human serum (% intact tracer, n=3) after 1 h (^{18}F-labeled, excepted of [^{177}Lu]Lu-DOTA-TATE) and 24 h (^{177}Lu-labeled) incubation at 37°C. (*Data taken from the literature,[1] #Data taken from the literature.[2])

Compound	IC_{50} [nM]	$\log D_{pH=7.4}$	HSA [%]	Intact tracer [%] 1 h	Intact tracer [%] 24 h
[^{nat}Ga]Ga-DOTA-TATE	2.07 ± 0.24	-	23	-	-
[^{68}Ga]Ga-DOTA-TATE	-	− 3.69*	-	-	-
[^{nat}Lu]Lu-DOTA-TATE	7.24 ± 0.91	-	51	-	-
[^{177}Lu]Lu-DOTA-TATE	-	− 3.70 ± 0.05	-	≥99 ± 0.0	98 ± 3.0
SiFA*lin*-TATE	7.46 ± 1.40	-	92	-	-
[^{18}F]SiFA*lin*-TATE	-	− 1.41 ± 0.07	-	98 ± 0.0#	-
[^{nat}Ga]Ga-(SiFA)BA-rhTATE1	4.60 ± 0.20	-	99	-	-
[^{18}F][^{nat}Ga]Ga-(SiFA)BA-rhTATE1	-	− 0.14 ± 0.05	-	-	-
[^{nat}Lu]Lu-(SiFA)BA-rhTATE3	5.19 ± 0.85	-	98	-	-
[^{18}F][^{nat}Lu]Lu-(SiFA)BA-rhTATE3	-	− 0.18 ± 0.05	-	≥99 ± 0.0	-
[^{177}Lu]Lu-(SiFA)BA-rhTATE3	-	− 0.39 ± 0.08	-	-	60 ± 1.0
[^{nat}Ga]Ga-(SiFA)SeFe-rhTATE1	3.10 ± 0.20	-	99	-	-
[^{18}F][^{nat}Ga]Ga-(SiFA)SeFe-rhTATE1	-	− 1.55 ± 0.08	-	≥99 ± 0.0	-
[^{nat}Ga]Ga-(SiFA)SeFe-rhTATE2	4.69 ± 0.83	-	99	-	-
[^{18}F][^{nat}Ga]Ga-(SiFA)SeFe-rhTATE2	-	− 1.58 ± 0.09	-	97 ± 1.3	-
[^{nat}Lu]Lu-(SiFA)SeFe-rhTATE3	5.46 ± 1.20	-	98	-	-
[^{18}F][^{nat}Lu]Lu-(SiFA)SeFe-rhTATE3	-	− 1.68 ± 0.08	-	99 ± 0.0	-

[177]Lu]Lu-(SiFA)SeFe-rhTATE3	-	–<br>1.71 ± 0.08	-	-	94 ± 3.0

Table S5: Biodistribution of the corresponding (SiFA)SeFe sstR2-rh-compounds in selected organs [%ID/g] at 1 h, p.i. in AR42J tumor-bearing female CD1-nu/nu mice.

Organ	[^{18}F](SiFA)SeFe-rhTATE1 1h (n=3)	[^{18}F](SiFA)SeFe-rhTATE3 1h (n=3)
Blood	0.67 ± 0.28	1.17 ± 0.22
Heart	0.42 ± 0.12	0.67 ± 0.17
Lung	6.42 ± 1.73	11.21 ± 2.24
Liver	1.66 ± 0.56	4.47 ± 0.47
Spleen	0.79 ± 0.11	1.92 ± 0.84
Pancreas	23.12 ± 5.77	23.78 ± 5.94
Stomach	16.52 ± 6.22	16.76 ± 4.63
Intestine	2.70 ± 0.67	3.40 ± 0.35
Kidney	22.90 ± 6.91	98.69 ± 7.62
Adrenal gland	2.96 ± 1.81	4.55 ± 1.44
Muscle	0.10 ± 0.03	0.35 ± 0.30
Bone	2.94 ± 1.33	2.08 ± 0.33
Tumor	25.08 ± 7.84	27.32 ± 8.86

Table S6: T/B ratio of the corresponding ^{18}F-labeled (SiFA)SeFe sstR2-compounds in selected organs [%ID/g] at 1 h p.i. in AR42J tumor-bearing female CD1-nu/nu mice.

Organ	[^{18}F](SiFA)SeFe-rhTATE1 1h (n=3)	[^{18}F](SiFA)SeFe-rhTATE3 1h (n=3)
Blood	46.42 ± 22.14	23.56 ± 8.01
Heart	67.92 ± 28.11	40.86 ± 12.58
Lung	3.88 ± 0.58	2.38 ± 0.51
Liver	15.56 ± 3.84	5.97 ± 1.48
Spleen	31.95 ± 8.40	15.63 ± 6.27
Pancreas	1.09 ± 0.24	1.19 ± 0.45
Stomach	1.60 ± 0.46	1.61 ± 0.19
Intestine	9.68 ± 3.06	7.85 ± 1.96
Kidney	1.14 ± 0.34	0.28 ± 0.09
Adrenal gland	16.65 ± 13.73	6.00± 0.22
Muscle	265.68 ± 92.79	125.38 ± 71.86
Bone	9.76 ± 4.10	13.45 ± 5.21

[1] M. Schottelius, J. Šimeček, F. Hoffmann, M. Willibald, M. Schwaiger, H. J. Wester, Twins in spirit - episode I: comparative preclinical evaluation of [(68)Ga]DOTATATE and [(68)Ga]HA-DOTATATE, *EJNMMI Res*, **2015**, *5*, 22.

[2] S. Niedermoser, J. Chin, C. Wängler, A. Kostikov, V. Bernard-Gauthier, N. Vogler, J. P. Soucy, A. J. McEwan, R. Schirrmacher, B. Wängler, In Vivo Evaluation of ^{18}F-SiFAlin-Modified TATE: A Potential Challenge for ^{68}Ga-DOTATATE, the Clinical Gold Standard for Somatostatin Receptor Imaging with PET, *J. Nucl. Med.*, **2015**, *56*, 1100-1105.
